# An improved parameterization procedure for NDDO-descendant semi-empirical methods

**DOI:** 10.1007/s00894-023-05499-3

**Published:** 2023-03-28

**Authors:** Adrian Wee Wen Ong, Steve Yueran Cao, Leong Chuan Kwek

**Affiliations:** 1 NUS High School of Mathematics and Science, 20 Clementi Avenue 1, 129957 Singapore, Singapore; 2 Centre for Quantum Technologies, National University of Singapore, 117543 Singapore, Singapore; 3 MajuLab, CNRS-UNS-NUS-NTU International Joint Research Unit, UMI 3654, 117543 Singapore, Singapore; 4 National Institute of Education, Nanyang Technological University, 1 Nanyang Walk, 637616 Singapore, Singapore; 5 School of Electrical and Electronic Engineering Block S2.1, 50 Nanyang Avenue, 639798 Singapore, Singapore

**Keywords:** NDDO, MNDO, Quantum chemistry

## Abstract

**Concept:**

MNDO-based semi-empirical methods in quantum chemistry have found widespread application in the modelling of large and complex systems. A method for the analytic evaluation of first and second derivatives of molecular properties against semi-empirical parameters in MNDO-based NDDO-descendant models is presented, and the resultant parameter Hessian is compared against the approximant currently used in parameterization for the PMx models.

**Methods:**

As a proof of concept, the exact parameter Hessian is employed in a limited reparameterization of MNDO for the elements C, H, N, O and F using 1206 molecules for reference data (heats of formation, ionization energies, dipole moments and reference geometries). The correctness of our MNDO implementation was verified by comparing the calculated molecular properties with the MOPAC program.

**Supplementary Information:**

The online version contains supplementary material available at 10.1007/s00894-023-05499-3.

## Introduction 

In modern computational chemistry, semi-empirical methods based on the neglect of diatomic differential overlap (NDDO) [1–12] have found widespread applications in studies where more computationally intensive ab initio methods are unfeasible. While semi-empirical model Hamiltonians developed via machine-learning have gained significant attention [13, 14], recent work on the use of machine-learning techniques to construct correction terms for molecular properties calculated at lower levels of theory [15, 16] or develop semi-empirical molecular Hamiltonians [17] suggests that semi-empirical methods can achieve chemical accuracy. Essential to the success of a developed semi-empirical model is a robust parameterization procedure that allows the model to best reproduce experimental data; as such, parameter optimization is an important area of study for the development of effective and accurate semi-empirical models.

While alternative formulations based on the NDDO approximation [18–22] have been proposed in recent years, most NDDO-descendant semi-empirical models [1, 2, 5, 9] use an identical formalism for the construction of the one-electron matrix $\mathbf{H}$ and two-electron matrix $\mathbf{G}$; with the exception of minor corrections to the asymptotic behaviour of two-electron integrals in PM7 [10], these methods also employ the same approximations and semi-empirical expressions [23, 24] for the evaluation of relevant molecular integrals. The difference between most NDDO-descendant models hence lies only in the parameter values chosen as well as the empirical expressions for core-core repulsion terms, which have been modified significantly between models [2, 9–11]; as such, any parameterization scheme developed for one NDDO-descendant model can be readily applied with few modifications to other NDDO-descendant models.

As noted by Stewart when developing PM7 [10], the parameter Hessian can be used to determine the nature of stationary points detected during parameterization; likewise, accurate second-derivative information can be used to great effect in searching for minima on surfaces. Construction of the parameter Hessian via direct differentiation of the error function appears straightforward; in differentiating the error function $\mathcal{S}$ with respect to parameters ${}^{{\mathrm Z}_{\mathrm A}}\mathrm p_{\mathrm i}$ and ${}^{{\mathrm Z}_{\mathrm B}}\mathrm p_{\mathrm i}$, we find that

1 $$\mathcal{S}=\sum_{\alpha }{\mathcal{C}}_{\alpha }^{2}{\left({\xi }_{\alpha }^{\mathrm{ref}}-{\xi }_{\alpha }\right)}^{2}$$

2 $$\frac{{\partial }^{2}\mathcal{S}}{\partial {}{}^{{Z}_{A}}{p}_{i}\partial {}{}^{{Z}_{B}}{p}_{j}}=2\sum_{\alpha }{\mathcal{C}}_{\alpha }^{2}\left[\left({\xi }_{\alpha }-{\xi }_{\alpha }^{\mathrm{ref}}\right)\frac{{\partial }^{2}{\xi }_{\alpha }}{\partial {}{}^{{Z}_{A}}{p}_{i}\partial {}{}^{{Z}_{B}}{p}_{j}}+\frac{\partial {\xi }_{\alpha }}{\partial {}{}^{{Z}_{A}}{p}_{i}}\frac{\partial {\xi }_{\alpha }}{\partial {}{}^{{Z}_{B}}{p}_{j}}\right]$$

It should be noted, however, that the parameter Hessian constructed in the development of PM7 appears to neglect second derivatives of the reference functions; as given in [10], the expression for $\frac{{\partial }^{2}\mathcal{S}}{\partial {}{}^{{Z}_{A}}{p}_{i}\partial {}{}^{{Z}_{B}}{p}_{j}}$ appears to be

3 $$\frac{{\partial }^{2}{\mathcal{S}}^{\mathrm{PM}7}}{\partial {}{}^{{Z}_{A}}{p}_{i}\partial {}{}^{{Z}_{B}}{p}_{j}}=2\sum_{\alpha }{\mathcal{C}}_{\alpha }^{2}\frac{\partial {\xi }_{\alpha }}{\partial {}{}^{{Z}_{A}}{p}_{i}}\frac{\partial {\xi }_{\alpha }}{\partial {}{}^{{Z}_{B}}{p}_{j}}$$

The neglect of the second-derivative term is expected to significantly affect the nature of the constructed Hessian matrix and its eigenvalues, impacting the quality of parameter optimization.

Evaluation of parameter derivatives of molecular properties via finite difference, as in standard procedure, results in numerical instability and may lead to an undesirable irreproducibility in results; thus, an analytical method for evaluation of parameter first and second derivatives is sought. This requires the evaluation of second derivatives of the density matrix as the idempotency condition [25] does not apply for derivatives of the ionization energy or dipole moment; an efficient method for solution of the second order coupled-perturbed Hartree–Fock (CPHF) equations is hence necessary for analytical derivative evaluation. NDDO methods formally operate in the Lowdin basis, where the overlap matrix between basis functions is substituted for the identity matrix; thus, the relevant equations for CPHF equation solution are greatly simplified and may be easily implemented.

### The second-order CPHF equations under the NDDO approximation

The form of the first-order CPHF equations in the Lowdin basis for both closed- and open-shell species under the NDDO approximation has been well-documented [26] due to their implementation in geometry optimization routines [26, 27]; the second-order CPHF equations have been presented only in the MO basis [28] and are hence given in analogous form for the UHF case in the Lowdin basis.

All equations presented in this section are based on the Unrestricted Hartree–Fock (UHF) formalism; equations for the restricted case are obtained as a special case of these results. Matrices for a specific spin (e.g. the Fock matrices for alpha- and beta-spin) are denoted as ${}^{\sigma }\mathbf{M}$, where $\sigma \in \left\{\alpha ,\beta \right\}$ is an arbitrary spin. $\mathbf{P}={}{}^{\alpha }\mathbf{P}+{}{}^{\beta }\mathbf{P}$ represents the density matrix in the Lowdin basis, which is the sum of the alpha- and beta-spin density matrices.

We first define the generalized Coulomb and exchange matrices $\mathbf{J}\left({\varvec{\Delta}}\right)$ and $\mathbf{K}\left({\varvec{\Lambda}}\right)$ as well as their associated static derivatives as the contraction of the relevant 2-electron integrals with arbitrary matrices ${\varvec{\Delta}}$ and ${\varvec{\Lambda}}$:

4 $${J}_{\mu \nu }\left({\varvec{\Delta}}\right)=\left\{\begin{array}{c}\sum_{\lambda \in A}{\Delta }_{\lambda \lambda }\left(\mu \nu |\lambda \lambda \right)+\sum_{\lambda ,\sigma \in B\ne A}{\Delta }_{\lambda \sigma }\left(\mu \nu |\lambda \sigma \right), \mu =\nu , \mu ,\nu \in A\\ 2{\Delta }_{\mu \nu }\left(\mu \nu |\mu \nu \right)+\sum_{\lambda ,\sigma \in B\ne A}{\Delta }_{\lambda \sigma }\left(\mu \nu |\lambda \sigma \right), \mu ,\nu \in A\\ 0, \mu \in A, \nu \in B\ne A\end{array}\right.$$

5 $${J}_{\mu \nu }^{{q}_{1}}\left({\varvec{\Delta}}\right)=\left\{\begin{array}{c}\sum_{\lambda \in A}{\Delta }_{\lambda \lambda }\frac{d\left(\mu \nu |\lambda \lambda \right)}{d{q}_{1}}+\sum_{\lambda ,\sigma \in B\ne A}{\Delta }_{\lambda \sigma }\frac{d\left(\mu \nu |\lambda \sigma \right)}{d{q}_{1}}, \mu =\nu , \mu ,\nu \in A\\ 2{\Delta }_{\mu \nu }\frac{d\left(\mu \nu |\mu \nu \right)}{d{q}_{1}}+\sum_{\lambda ,\sigma \in B\ne A}{\Delta }_{\lambda \sigma }\frac{d\left(\mu \nu |\lambda \sigma \right)}{d{q}_{1}}, \mu ,\nu \in A\\ 0, \mu \in A, \nu \in B\ne A\end{array}\right.$$

6 $${J}_{\mu \nu }^{{q}_{1}{q}_{2}}\left({\varvec{\Delta}}\right)=\left\{\begin{array}{c}\sum_{\lambda \in A}{\Delta }_{\lambda \lambda }\frac{{d}^{2}\left(\mu \nu |\lambda \lambda \right)}{d{q}_{1}d{q}_{2}}+\sum_{\lambda ,\sigma \in B\ne A}{\Delta }_{\lambda \sigma }\frac{{d}^{2}\left(\mu \nu |\lambda \sigma \right)}{d{q}_{1}d{q}_{2}}, \mu =\nu , \mu ,\nu \in A\\ 2{\Delta }_{\mu \nu }\frac{{d}^{2}\left(\mu \nu |\mu \nu \right)}{d{q}_{1}d{q}_{2}}+\sum_{\lambda ,\sigma \in B\ne A}{\Delta }_{\lambda \sigma }\frac{{d}^{2}\left(\mu \nu |\lambda \sigma \right)}{d{q}_{1}d{q}_{2}}, \mu ,\nu \in A\\ 0, \mu \in A, \nu \in B\ne A\end{array}\right.$$

7 $${K}_{\mu \nu }\left({\varvec{\Lambda}}\right)=\left\{\begin{array}{c}\sum_{\lambda \in A}{\Lambda }_{\lambda \lambda }\left(\mu \lambda |\nu \lambda \right), \mu =\nu , \mu ,\nu \in A\\ {\Lambda }_{\mu \nu }\left[\left(\mu \nu |\mu \nu \right)+\left(\mu \mu |\nu \nu \right)\right], \mu ,\nu \in A\\ \sum_{\begin{array}{c}\lambda \in A\\ \sigma \in B\end{array}}{\Lambda }_{\lambda \sigma }\left(\mu \lambda |\nu \sigma \right), \mu \in A, \nu \in B\ne A\end{array}\right.$$

8 $${K}_{\mu \nu }^{{q}_{1}}\left({\varvec{\Lambda}}\right)=\left\{\begin{array}{c}\sum_{\lambda \in A}{\Lambda }_{\lambda \lambda }\frac{d\left(\mu \lambda |\nu \lambda \right)}{d{q}_{1}}, \mu =\nu , \mu ,\nu \in A\\ {\Lambda }_{\mu \nu }\left[\frac{d\left(\mu \nu |\mu \nu \right)}{d{q}_{1}}+\frac{d\left(\mu \mu |\nu \nu \right)}{d{q}_{1}}\right], \mu ,\nu \in A\\ \sum_{\begin{array}{c}\lambda \in A\\ \sigma \in B\end{array}}{\Lambda }_{\lambda \sigma }\frac{d\left(\mu \lambda |\nu \sigma \right)}{d{q}_{1}}, \mu \in A, \nu \in B\ne A\end{array}\right.$$

9 $${K}_{\mu \nu }^{{q}_{1}{q}_{2}}\left({\varvec{\Lambda}}\right)=\left\{\begin{array}{c}\sum_{\lambda \in A}{\Lambda }_{\lambda \lambda }\frac{{d}^{2}\left(\mu \lambda |\nu \lambda \right)}{d{q}_{1}d{q}_{2}}, \mu =\nu , \mu ,\nu \in A\\ {\Lambda }_{\mu \nu }\left[\frac{{d}^{2}\left(\mu \nu |\mu \nu \right)}{d{q}_{1}d{q}_{2}}+\frac{{d}^{2}\left(\mu \mu |\nu \nu \right)}{d{q}_{1}d{q}_{2}}\right], \mu ,\nu \in A\\ \sum_{\begin{array}{c}\lambda \in A\\ \sigma \in B\end{array}}{\Lambda }_{\lambda \sigma }\frac{{d}^{2}\left(\mu \lambda |\nu \sigma \right)}{d{q}_{1}d{q}_{2}}, \mu \in A, \nu \in B\ne A\end{array}\right.$$

In conventional NDDO methods [1–11], the core matrix is given by

10 $${\mathbf{H}}_{\mu \nu }=\left\{\begin{array}{c}{}{}^{{Z}_{A}}{U}_{\mu \mu }+\sum_{B\ne A}{V}_{\mu \mu ,B}, \mu =\nu , \mu ,\nu \in A\\ \sum_{B\ne A}{V}_{\mu \nu ,B}, \mu ,\nu \in A\\ {\beta }_{\mu \nu }, \mu \in A, \nu \in B\ne A\end{array}\right.$$

In the above, ${V}_{\mu \nu ,B}$ represents the two-centre nuclear-electron attraction integral between basis functions $\mu ,\nu$ and atom $B$; ${\beta }_{\mu \nu }=\frac{{\beta }_{\mu }+{\beta }_{\nu }}{2}{S}_{\mu \nu }$ represents the resonance integral between two basis functions on different atoms.

The Fock matrix in the NDDO formalism can hence be given as follows:

11 $${}^{\sigma }\mathbf{F}=\mathbf{H}+\mathbf{J}\left(\mathbf{P}\right)-\mathbf{K}\left({}{}^{\sigma }\mathbf{P}\right)$$

In matrix form, the first-order orbital coefficients ${}^{\sigma }{\mathbf{x}}^{{q}_{1}}$ are related to the direct derivative of the (AO) coefficient matrix ${}^{\sigma }\mathbf{C}$ as follows:

12 $$\frac{d{}{}^{\sigma }\mathbf{C}}{d{q}_{1}}={}{}^{\sigma }\mathbf{C}{}{}^{\sigma }{\mathbf{x}}^{{q}_{1}}$$

Analogously, the second-order orbital coefficients can be obtained via differentiation of the first-order orbital coefficients:

13 $$\frac{d{}{}^{\sigma }{\mathbf{x}}^{{q}_{1}}}{d{q}_{2}}={}{}^{\sigma }{{\varvec{\upgamma}}}^{{q}_{1}{q}_{2}}-{}{}^{\sigma }{\mathbf{x}}^{{q}_{2}} {}{}^{\sigma }{\mathbf{x}}^{{q}_{1}}$$

Application of the orthonormality condition ${{}^{\sigma }\mathbf{C}}^{\mathrm{T}}{}{}^{\sigma }\mathbf{C}=1$ yields the relevant commutator relations for the first- and second-order orbital coefficients:

14 $${}^{\sigma }{x}_{ij}^{{q}_{1}}+{}{}^{\sigma }{x}_{ji}^{{q}_{1}}=0$$

15 $${}^{\sigma }{\gamma }_{ij}^{{q}_{1}{q}_{2}}+{}{}^{\sigma }{\gamma }_{ji}^{{q}_{1}{q}_{2}}={\sum }_{k}\left({}{}^{\sigma }{x}_{ik}^{{q}_{1}}{}{}^{\sigma }{x}_{kj}^{{q}_{2}}+{}{}^{\sigma }{x}_{ik}^{{q}_{2}}{}{}^{\sigma }{x}_{kj}^{{q}_{1}}\right)$$

The derivatives of the off-diagonal elements of the MO-basis Fock matrix must be zero due to the variational condition:

16 $${}^{\sigma }\mathfrak{F}={{}{}^{\sigma }\mathbf{C}}^{\mathrm{T}}{}{}^{\sigma }\mathbf{F}{}{}^{\sigma }\mathbf{C}$$

17 $${\left(\frac{d{}{}^{\sigma }\mathfrak{F}}{d{q}_{1}}\right)}_{ij}=0, {\left(\frac{{d}^{2}{}{}^{\sigma }\mathfrak{F}}{d{q}_{1}d{q}_{2}}\right)}_{ij}=0, i\ne j$$

The first-order CPHF equations are well-documented in the literature and are hence only presented for completeness.

Evaluation of the first derivative $\frac{d{}{}^{\sigma }\mathfrak{F}}{d{q}_{1}}$ yields the following:

18 $$\frac{d{}{}^{\sigma }\mathfrak{F}}{d{q}_{1}}={\left({}{}^{\sigma }{\mathbf{x}}^{{q}_{1}}\right)}^{\mathrm{T}}{}{}^{\sigma }\mathfrak{F}+{{}{}^{\sigma }\mathbf{C}}^{\mathrm{T}}\frac{d{}{}^{\sigma }\mathbf{F}}{d{q}_{1}}{}{}^{\sigma }\mathbf{C}+{}{}^{\sigma }\mathfrak{F}{}{}^{\sigma }{\mathbf{x}}^{{q}_{1}}$$

19 $$\frac{d{}{}^{\sigma }\mathbf{F}}{d{q}_{1}}=\frac{d\mathbf{H}}{d{q}_{1}}+{\mathbf{J}}^{{q}_{1}}\left(\mathbf{P}\right)-{\mathbf{K}}^{{q}_{1}}\left({}{}^{\sigma }\mathbf{P}\right)+\mathbf{J}\left(\frac{d\mathbf{P}}{d{q}_{1}}\right)-\mathbf{K}\left(\frac{d{}{}^{\sigma }\mathbf{P}}{d{q}_{1}}\right)$$

The “static” derivative term $\frac{d\mathbf{H}}{d{q}_{1}}+{\mathbf{J}}^{{q}_{1}}\left(\mathbf{P}\right)-{\mathbf{K}}^{{q}_{1}}\left({}{}^{\sigma }\mathbf{P}\right)$ is referred to as ${}^{\sigma }{\mathbf{F}}^{{q}_{1}}$ and the response term $\mathbf{J}\left(\frac{d\mathbf{P}}{d{q}_{1}}\right)-\mathbf{K}\left(\frac{d{}{}^{\sigma }\mathbf{P}}{d{q}_{1}}\right)$ is referred to as ${}^{\sigma }{\mathbf{R}}^{{q}_{1}}$; collection of terms yields

20 $${{}^{\sigma }\mathfrak{F}}^{{q}_{1}}={{}{}^{\sigma }\mathbf{C}}^{\mathrm{T}}\left(\frac{d\mathbf{H}}{d{q}_{1}}+{\mathbf{J}}^{{q}_{1}}\left(\mathbf{P}\right)-{\mathbf{K}}^{{q}_{1}}\left({}{}^{\sigma }\mathbf{P}\right)\right){}{}^{\sigma }\mathbf{C}$$

21 $${{}^{\sigma }\mathfrak{R}}^{{q}_{1}}={{}{}^{\sigma }\mathbf{C}}^{\mathrm{T}}\left(\mathbf{J}\left(\frac{d\mathbf{P}}{d{q}_{1}}\right)-\mathbf{K}\left(\frac{d{}{}^{\sigma }\mathbf{P}}{d{q}_{1}}\right)\right){}{}^{\sigma }\mathbf{C}$$

22 $$\begin{array}{c}\frac{d{}{}^{\sigma }\mathfrak{F}}{d{q}_{1}}={\left({}{}^{\sigma }{\mathbf{x}}^{{q}_{1}}\right)}^{\mathrm{T}}{}{}^{\sigma }\mathfrak{F}+{{}{}^{\sigma }\mathbf{C}}^{\mathrm{T}}\left({}{}^{\sigma }{\mathbf{F}}^{{q}_{1}}+{}{}^{\sigma }{\mathbf{R}}^{{q}_{1}}\right){}{}^{\sigma }\mathbf{C}+{}{}^{\sigma }\mathfrak{F}{}{}^{\sigma }{\mathbf{x}}^{{q}_{1}}\\ ={\left({}{}^{\sigma }{\mathbf{x}}^{{q}_{1}}\right)}^{\mathrm{T}}{}{}^{\sigma }\mathfrak{F}+{{}{}^{\sigma }\mathfrak{F}}^{{q}_{1}}+{{}{}^{\sigma }\mathfrak{R}}^{{q}_{1}}+{}{}^{\sigma }\mathfrak{F}{}{}^{\sigma }{\mathbf{x}}^{{q}_{1}}\end{array}$$

Thus, the first-order CPHF equations are given as follows, as reported in [26, 27]:

23 $${\left(\frac{d{}{}^{\sigma }\mathfrak{F}}{d{q}_{1}}\right)}_{ij}={}{}^{\sigma }{x}_{ji}^{{q}_{1}}{}{}^{\sigma }{\epsilon }_{j}+{}{}^{\sigma }{x}_{ij}^{{q}_{1}}{}{}^{\sigma }{\epsilon }_{i}+{{}{}^{\sigma }\mathfrak{F}}_{ij}^{{q}_{1}}+{{}{}^{\sigma }\mathfrak{R}}_{ij}^{{q}_{1}}=0, i\ne j$$

24 $$\left({}^{\sigma }{\epsilon }_{j}-{}{}^{\sigma }{\epsilon }_{i}\right){}{}^{\sigma }{x}_{ij}^{{q}_{1}}-{{}{}^{\sigma }\mathfrak{R}}_{ij}^{{q}_{1}}={{}{}^{\sigma }\mathfrak{F}}_{ij}^{{q}_{1}}$$

To evaluate ${{}^{\sigma }\mathfrak{R}}_{ij}^{{q}_{1}}$, $\frac{d {}{}^{\sigma }\mathbf{P}}{d {q}_{1}}$ must be cast in terms of ${}^{\sigma }{\mathbf{x}}^{{q}_{1}}$; since ${}^{\sigma }\mathbf{P}={{}{}^{\sigma }\mathbf{C}}_{occ}^{\mathrm{T}}{}{}^{\sigma }{\mathbf{C}}_{occ}$,

25 $$\begin{array}{c}{\left(\frac{d {}{}^{\sigma }\mathbf{P}}{d {q}_{1}}\right)}_{\mu \nu }={\sum }_{i\in occ.}{\sum }_{j}\left({}{}^{\sigma }{c}_{\mu j}{}{}^{\sigma }{c}_{\nu i}+{}{}^{\sigma }{c}_{\mu i}{}{}^{\sigma }{c}_{\nu j}\right){x}_{ji}^{{q}_{1}}=-{\sum }_{i\in occ.}{\sum }_{j\in virt.}\left({}{}^{\sigma }{c}_{\mu j}{}{}^{\sigma }{c}_{\nu i}+{}{}^{\sigma }{c}_{\mu i}{}{}^{\sigma }{c}_{\nu j}\right){x}_{ij}^{{q}_{1}}\end{array}$$

Lastly, the expression for $\frac{d\mathbf{H}}{d{q}_{1}}$ is of Eq. (10):

26 $${\left(\frac{d\mathbf{H}}{d{q}_{1}}\right)}_{\mu \nu }=\left\{\begin{array}{c}\frac{d{}{}^{{Z}_{A}}{U}_{\mu \mu }}{d{q}_{1}}+\sum_{B\ne A}\frac{d{V}_{\mu \mu ,B}}{d{q}_{1}}, \mu =\nu , \mu ,\nu \in A\\ \sum_{B\ne A}\frac{d{V}_{\mu \nu ,B}}{d{q}_{1}}, \mu ,\nu \in A\\ \frac{d{\beta }_{\mu \nu }}{d{q}_{1}}, \mu \in A, \nu \in B\ne A\end{array}\right.$$

The first-order CPHF equations are hence linear in the first-order coefficients ${x}_{ij}^{{q}_{1}}$.

Since only the occupied-virtual block of ${}^{\sigma }{\mathbf{x}}^{{q}_{1}}$ is necessary to solve for $\frac{d{}{}^{\sigma }\mathbf{P}}{d{q}_{1}}$, the remaining elements of ${}^{\sigma }{\mathbf{x}}^{{q}_{1}}$ are evaluated afterwards via direct substitution:

27 $${}^{\sigma }{x}_{ij}^{{q}_{1}}=\left\{\begin{array}{c}0, i=j\\ \\ \\ \frac{{{}{}^{\sigma }\mathfrak{F}}^{{q}_{1}}+{{}{}^{\sigma }\mathfrak{R}}^{{q}_{1}}}{{}{}^{\sigma }{\epsilon }_{j}-{}{}^{\sigma }{\epsilon }_{i}}, i\ne j\end{array}\right.$$

The full matrix ${}^{\sigma }{\mathbf{x}}^{{q}_{1}}$, not just the occupied-virtual block, is necessary for solution of the second-order CPHF equations. These are obtained via further differentiation of Eq. (18):

28 $$\begin{array}{c}\frac{{d}^{2}{}{}^{\sigma }\mathfrak{F}}{d{q}_{1}d{q}_{2}}={\left(\frac{d{}{}^{\sigma }{\mathbf{x}}^{{q}_{1}}}{d{q}_{2}}\right)}^{\mathrm{T}}{}{}^{\sigma }\mathfrak{F}+{\left({}{}^{\sigma }{\mathbf{x}}^{{q}_{1}}\right)}^{\mathrm{T}}\frac{d{}{}^{\sigma }\mathfrak{F}}{d{q}_{2}}+{\left({}{}^{\sigma }{\mathbf{x}}^{{q}_{2}}\right)}^{\mathrm{T}}\left({{}{}^{\sigma }\mathfrak{F}}^{{q}_{1}}+{{}{}^{\sigma }\mathfrak{R}}^{{q}_{1}}\right)\\ +{{}{}^{\sigma }\mathbf{C}}^{\mathrm{T}}\frac{{d}^{2}{}{}^{\sigma }\mathbf{F}}{d{q}_{1}d{q}_{2}}{}{}^{\sigma }\mathbf{C}+\left({{}{}^{\sigma }\mathfrak{F}}^{{q}_{1}}+{{}{}^{\sigma }\mathfrak{R}}^{{q}_{1}}\right){}{}^{\sigma }{\mathbf{x}}^{{q}_{2}}+\frac{d{}{}^{\sigma }\mathfrak{F}}{d{q}_{2}}{}{}^{\sigma }{\mathbf{x}}^{{q}_{1}}+{}{}^{\sigma }\mathfrak{F}\frac{d{}{}^{\sigma }{\mathbf{x}}^{{q}_{1}}}{d{q}_{2}}\end{array}$$

Simplification yields:

29 $$\begin{array}{c}{\left(\frac{{d}^{2}{}{}^{\sigma }\mathfrak{F}}{d{q}_{1}d{q}_{2}}\right)}_{ij}={\left({{}{}^{\sigma }\mathbf{C}}^{\mathrm{T}}\frac{{d}^{2}{}{}^{\sigma }\mathbf{F}}{d{q}_{1}d{q}_{2}}{}{}^{\sigma }\mathbf{C}\right)}_{ij}+{}{}^{\sigma }{\gamma }_{ji}^{{q}_{1}{q}_{2}}{}{}^{\sigma }{\epsilon }_{j}\\ +{\left({\left({}{}^{\sigma }{\mathbf{x}}^{{q}_{1}}\right)}^{\mathrm{T}}\left({{}{}^{\sigma }\mathfrak{F}}^{{q}_{2}}+{{}{}^{\sigma }\mathfrak{R}}^{{q}_{2}}\right)+{\left({}{}^{\sigma }{\mathbf{x}}^{{q}_{1}}\right)}^{\mathrm{T}}{}{}^{\sigma }{\varvec{\upepsilon}}{}{}^{\sigma }{\mathbf{x}}^{{q}_{2}}+\left({{}{}^{\sigma }\mathfrak{F}}^{{q}_{2}}+{{}{}^{\sigma }\mathfrak{R}}^{{q}_{2}}\right){}{}^{\sigma }{\mathbf{x}}^{{q}_{1}}\right)}_{ij}+{}{}^{\sigma }{\gamma }_{ij}^{{q}_{1}{q}_{2}}{}{}^{\sigma }{\epsilon }_{i}\\ +{\left({\left({}{}^{\sigma }{\mathbf{x}}^{{q}_{2}}\right)}^{\mathrm{T}}\left({{}{}^{\sigma }\mathfrak{F}}^{{q}_{1}}+{{}{}^{\sigma }\mathfrak{R}}^{{q}_{2}}\right)+{\left({}{}^{\sigma }{\mathbf{x}}^{{q}_{2}}\right)}^{\mathrm{T}}{}{}^{\sigma }{\varvec{\upepsilon}}{}{}^{\sigma }{\mathbf{x}}^{{q}_{1}}+\left({{}{}^{\sigma }\mathfrak{F}}^{{q}_{1}}+{{}{}^{\sigma }\mathfrak{R}}^{{q}_{1}}\right){}{}^{\sigma }{\mathbf{x}}^{{q}_{2}}\right)}_{ij}\end{array}$$

Application of the commutator relation further reduces the expression complexity:

30 $$\begin{array}{c}{\left(\frac{{d}^{2}{}{}^{\sigma }\mathfrak{F}}{d{q}_{1}d{q}_{2}}\right)}_{ij}={\left({{}{}^{\sigma }\mathbf{C}}^{\mathrm{T}}\frac{{d}^{2}{}{}^{\sigma }\mathbf{F}}{d{q}_{1}d{q}_{2}}{}{}^{\sigma }\mathbf{C}\right)}_{ij}-\left({}{}^{\sigma }{\epsilon }_{j}-{}{}^{\sigma }{\epsilon }_{i}\right){}{}^{\sigma }{\gamma }_{ij}^{{q}_{1}{q}_{2}}+{\left(\left({}{}^{\sigma }{\mathbf{x}}^{{q}_{1}}{}{}^{\sigma }{\mathbf{x}}^{{q}_{2}}+{}{}^{\sigma }{\mathbf{x}}^{{q}_{2}}{}{}^{\sigma }{\mathbf{x}}^{{q}_{1}}\right){}{}^{\sigma }{\varvec{\upepsilon}}\right)}_{ij}\\ +{\left({\left({}{}^{\sigma }{\mathbf{x}}^{{q}_{1}}\right)}^{\mathrm{T}}\left({{}{}^{\sigma }\mathfrak{F}}^{{q}_{2}}+{{}{}^{\sigma }\mathfrak{R}}^{{q}_{2}}\right)+{\left({}{}^{\sigma }{\mathbf{x}}^{{q}_{1}}\right)}^{\mathrm{T}}{}{}^{\sigma }{\varvec{\upepsilon}}{}{}^{\sigma }{\mathbf{x}}^{{q}_{2}}+\left({{}{}^{\sigma }\mathfrak{F}}^{{q}_{2}}+{{}{}^{\sigma }\mathfrak{R}}^{{q}_{2}}\right){}{}^{\sigma }{\mathbf{x}}^{{q}_{1}}\right)}_{ij}\\ +{\left({\left({}{}^{\sigma }{\mathbf{x}}^{{q}_{2}}\right)}^{\mathrm{T}}\left({{}{}^{\sigma }\mathfrak{F}}^{{q}_{1}}+{{}{}^{\sigma }\mathfrak{R}}^{{q}_{2}}\right)+{\left({}{}^{\sigma }{\mathbf{x}}^{{q}_{2}}\right)}^{\mathrm{T}}{}{}^{\sigma }{\varvec{\upepsilon}}{}{}^{\sigma }{\mathbf{x}}^{{q}_{1}}+\left({{}{}^{\sigma }\mathfrak{F}}^{{q}_{1}}+{{}{}^{\sigma }\mathfrak{R}}^{{q}_{1}}\right){}{}^{\sigma }{\mathbf{x}}^{{q}_{2}}\right)}_{ij}\end{array}$$

Evaluation of $\frac{{d}^{2}{}{}^{\sigma }\mathbf{F}}{d{q}_{1}d{q}_{2}}$ is performed analogously to Eq. (19), albeit with significantly more terms in the resultant expression:

31 $$\begin{array}{c}\frac{{d}^{2}{}{}^{\sigma }\mathbf{F}}{d{q}_{1}d{q}_{2}}=\frac{{d}^{2}\mathbf{H}}{d{q}_{1}d{q}_{2}}+{\mathbf{J}}^{{q}_{1}{q}_{2}}\left(\mathbf{P}\right)+{\mathbf{J}}^{{q}_{1}}\left(\frac{d\mathbf{P}}{d{q}_{2}}\right)+{\mathbf{J}}^{{q}_{2}}\left(\frac{d\mathbf{P}}{d{q}_{1}}\right)-{\mathbf{K}}^{{q}_{1}{q}_{2}}\left({}{}^{\sigma }\mathbf{P}\right)\\ -{\mathbf{K}}^{{q}_{1}}\left(\frac{d{}{}^{\sigma }\mathbf{P}}{d{q}_{2}}\right)-{\mathbf{K}}^{{q}_{2}}\left(\frac{d{}{}^{\sigma }\mathbf{P}}{d{q}_{1}}\right)+\mathbf{J}\left(\frac{{d}^{2}\mathbf{P}}{d{q}_{1}d{q}_{2}}\right)-\mathbf{K}\left(\frac{{d}^{2}{}{}^{\sigma }\mathbf{P}}{d{q}_{1}d{q}_{2}}\right)\end{array}$$

Lastly, direct differentiation of the density matrix yields

32 $$\begin{array}{c}{\left(\frac{{d}^{2}{}{}^{\sigma }\mathbf{P}}{d{q}_{1}d{q}_{2}}\right)}_{\mu \nu }={\sum }_{i\in occ.}{\sum }_{j,k}\left({}{}^{\sigma }{c}_{\mu k}{}{}^{\sigma }{c}_{\nu i}+{}{}^{\sigma }{c}_{\mu i}{}{}^{\sigma }{c}_{\nu k}\right)\left({}{}^{\sigma }{x}_{kj}^{{q}_{2}}{}{}^{\sigma }{x}_{ji}^{{q}_{1}}+{}{}^{\sigma }{x}_{kj}^{{q}_{1}}{}{}^{\sigma }{x}_{ji}^{{q}_{2}}\right)\\ \begin{array}{c}+{\sum }_{i\in occ.}{\sum }_{j\in occ.}{\sum }_{k}{}{}^{\sigma }{c}_{\mu i}{}{}^{\sigma }{c}_{\nu j}\left({}{}^{\sigma }{x}_{ik}^{{q}_{1}}{}{}^{\sigma }{x}_{kj}^{{q}_{2}}+{}{}^{\sigma }{x}_{ik}^{{q}_{2}}{}{}^{\sigma }{x}_{kj}^{{q}_{1}}\right)\\ +{\sum }_{i\in occ.}{\sum }_{j,k}\left({}{}^{\sigma }{c}_{\mu j}{}{}^{\sigma }{c}_{\nu k}+{}{}^{\sigma }{c}_{\mu k}{}{}^{\sigma }{c}_{\nu j}\right){}{}^{\sigma }{x}_{ki}^{{q}_{1}}{}{}^{\sigma }{x}_{ji}^{{q}_{2}}\\ -{\sum }_{i\in occ.}{\sum }_{j\in virt.}\left({}{}^{\sigma }{c}_{\mu j}{}{}^{\sigma }{c}_{\nu i}+{}{}^{\sigma }{c}_{\mu i}{}{}^{\sigma }{c}_{\nu j}\right){}{}^{\sigma }{\gamma }_{ij}^{{q}_{1}{q}_{2}}\end{array}\end{array}$$

Since the first three terms in ${\left(\frac{{d}^{2}{}{}^{\sigma }\mathbf{P}}{d{q}_{1}d{q}_{2}}\right)}_{\mu \nu }$ are independent of the second-order coefficients, we define

33 $${}^{\sigma }{{\varvec{\uprho}}}_{\mu \nu }^{{q}_{1}{q}_{2}} =-{\sum }_{i\in occ.}{\sum }_{j\in virt.}\left({}{}^{\sigma }{c}_{\mu j}{}{}^{\sigma }{c}_{\nu i}+{}{}^{\sigma }{c}_{\mu i}{}{}^{\sigma }{c}_{\nu j}\right){}{}^{\sigma }{\gamma }_{ij}^{{q}_{1}{q}_{2}}$$

34 $$\begin{array}{c}{}{}^{\sigma }{\mathbf{\varsigma }}_{\mu \nu }^{{q}_{1}{q}_{2}}={\sum }_{i\in occ.}{\sum }_{j,k}\left({}{}^{\sigma }{c}_{\mu k}{}{}^{\sigma }{c}_{\nu i}+{}{}^{\sigma }{c}_{\mu i}{}{}^{\sigma }{c}_{\nu k}\right)\left({}{}^{\sigma }{x}_{kj}^{{q}_{2}}{}{}^{\sigma }{x}_{ji}^{{q}_{1}}+{}{}^{\sigma }{x}_{kj}^{{q}_{1}}{}{}^{\sigma }{x}_{ji}^{{q}_{2}}\right)\\ \begin{array}{c}+{\sum }_{i\in occ.}{\sum }_{j\in occ.}{\sum }_{k}{}{}^{\sigma }{c}_{\mu i}{}{}^{\sigma }{c}_{\nu j}\left({}{}^{\sigma }{x}_{ik}^{{q}_{1}}{}{}^{\sigma }{x}_{kj}^{{q}_{2}}+{}{}^{\sigma }{x}_{ik}^{{q}_{2}}{}{}^{\sigma }{x}_{kj}^{{q}_{1}}\right)\\ +{\sum }_{i\in occ.}{\sum }_{j,k}\left({}{}^{\sigma }{c}_{\mu j}{}{}^{\sigma }{c}_{\nu k}+{}{}^{\sigma }{c}_{\mu k}{}{}^{\sigma }{c}_{\nu j}\right){}{}^{\sigma }{x}_{ki}^{{q}_{1}}{}{}^{\sigma }{x}_{ji}^{{q}_{2}}\end{array}\end{array}$$

35 $$\frac{{d}^{2}{}{}^{\sigma }\mathbf{P}}{d{q}_{1}d{q}_{2}}={}{}^{\sigma }{{\varvec{\uprho}}}^{{q}_{1}{q}_{2}}+{}{}^{\sigma }{\mathbf{\varsigma }}^{{q}_{1}{q}_{2}}$$

The static derivative term in $\frac{{d}^{2}{}{}^{\sigma }\mathbf{F}}{d{q}_{1}d{q}_{2}}$ is hence termed ${{}^{\sigma }\mathbf{F}}^{{q}_{1}{q}_{2}}$, with the response term correspondingly referred to as ${{}^{\sigma }\mathbf{R}}^{{q}_{1}{q}_{2}}$:

36 $$\begin{array}{c}{{}^{\sigma }\mathbf{F}}^{{q}_{1}{q}_{2}}=\frac{{d}^{2}\mathbf{H}}{d{q}_{1}d{q}_{2}}+{\mathbf{J}}^{{q}_{1}{q}_{2}}\left(\mathbf{P}\right)+{\mathbf{J}}^{{q}_{1}}\left(\frac{d\mathbf{P}}{d{q}_{2}}\right)+{\mathbf{J}}^{{q}_{2}}\left(\frac{d\mathbf{P}}{d{q}_{1}}\right)\\ -{\mathbf{K}}^{{q}_{1}{q}_{2}}\left({}{}^{\sigma }\mathbf{P}\right)-{\mathbf{K}}^{{q}_{1}}\left(\frac{d{}{}^{\sigma }\mathbf{P}}{d{q}_{2}}\right)-{\mathbf{K}}^{{q}_{2}}\left(\frac{d{}{}^{\sigma }\mathbf{P}}{d{q}_{1}}\right)+\mathbf{J}\left({\mathbf{\varsigma }}^{{q}_{1}{q}_{2}}\right)-\mathbf{K}\left({}{}^{\sigma }{\mathbf{\varsigma }}^{{q}_{1}{q}_{2}}\right)\end{array}$$

37 $${{}^{\sigma }\mathbf{R}}^{{q}_{1}{q}_{2}}=\mathbf{J}\left({{\varvec{\uprho}}}^{{q}_{1}{q}_{2}}\right)-\mathbf{K}\left({}{}^{\sigma }{{\varvec{\uprho}}}^{{q}_{1}{q}_{2}}\right)$$

Accordingly, defining ${{}^{\sigma }\mathfrak{F}}^{{q}_{1}{q}_{2}}$ and ${{}^{\sigma }\mathfrak{R}}^{{q}_{1}{q}_{2}}$ yields the second-order CPHF equations:

38 $$\begin{array}{c}{{}^{\sigma }\mathfrak{F}}^{{q}_{1}{q}_{2}}={{}{}^{\sigma }\mathbf{C}}^{\mathrm{T}}{}{}^{\sigma }{\mathbf{F}}^{{q}_{1}{q}_{2}}{}{}^{\sigma }\mathbf{C}+\left({}{}^{\sigma }{\mathbf{x}}^{{q}_{1}}{}{}^{\sigma }{\mathbf{x}}^{{q}_{2}}+{}{}^{\sigma }{\mathbf{x}}^{{q}_{2}}{}{}^{\sigma }{\mathbf{x}}^{{q}_{1}}\right){}{}^{\sigma }{\varvec{\upepsilon}}\\ +{\left({}{}^{\sigma }{\mathbf{x}}^{{q}_{1}}\right)}^{\mathrm{T}}\left({{}{}^{\sigma }\mathfrak{F}}^{{q}_{2}}+{{}{}^{\sigma }\mathfrak{R}}^{{q}_{2}}\right)+{\left({}{}^{\sigma }{\mathbf{x}}^{{q}_{1}}\right)}^{\mathrm{T}}{}{}^{\sigma }{\varvec{\upepsilon}}{}{}^{\sigma }{\mathbf{x}}^{{q}_{2}}+\left({{}{}^{\sigma }\mathfrak{F}}^{{q}_{2}}+{{}{}^{\sigma }\mathfrak{R}}^{{q}_{2}}\right){}{}^{\sigma }{\mathbf{x}}^{{q}_{1}}\\ +{\left({}{}^{\sigma }{\mathbf{x}}^{{q}_{2}}\right)}^{\mathrm{T}}\left({{}{}^{\sigma }\mathfrak{F}}^{{q}_{1}}+{{}{}^{\sigma }\mathfrak{R}}^{{q}_{2}}\right)+{\left({}{}^{\sigma }{\mathbf{x}}^{{q}_{2}}\right)}^{\mathrm{T}}{}{}^{\sigma }{\varvec{\upepsilon}}{}{}^{\sigma }{\mathbf{x}}^{{q}_{1}}+\left({{}{}^{\sigma }\mathfrak{F}}^{{q}_{1}}+{{}{}^{\sigma }\mathfrak{R}}^{{q}_{1}}\right){}{}^{\sigma }{\mathbf{x}}^{{q}_{2}}\end{array}$$

39 $${{}^{\sigma }\mathfrak{R}}^{{q}_{1}{q}_{2}}={{}{}^{\sigma }\mathbf{C}}^{\mathrm{T}}\left(\mathbf{J}\left({{\varvec{\uprho}}}^{{q}_{1}{q}_{2}}\right)-\mathbf{K}\left({}{}^{\sigma }{{\varvec{\uprho}}}^{{q}_{1}{q}_{2}}\right)\right){}{}^{\sigma }\mathbf{C}$$

40 $${\left(\frac{{d}^{2}\mathbf{H}}{d{q}_{1}d{q}_{2}}\right)}_{\mu \nu }=\left\{\begin{array}{c}\frac{{d}^{2}{}{}^{{Z}_{A}}{U}_{\mu \mu }}{d{q}_{1}d{q}_{2}}+\sum_{B\ne A}\frac{{d}^{2}{V}_{\mu \mu ,B}}{d{q}_{1}d{q}_{2}}, \mu =\nu , \mu ,\nu \in A\\ \sum_{B\ne A}\frac{{d}^{2}{V}_{\mu \nu ,B}}{d{q}_{1}d{q}_{2}}, \mu ,\nu \in A\\ \frac{{d}^{2}{\beta }_{\mu \nu }}{d{q}_{1}d{q}_{2}}, \mu \in A, \nu \in B\ne A\end{array}\right.$$

41 $$\left({}^{\sigma }{\epsilon }_{j}-{}{}^{\sigma }{\epsilon }_{i}\right){}{}^{\sigma }{\gamma }_{ij}^{{q}_{1}{q}_{2}}-{{}{}^{\sigma }\mathfrak{R}}_{ij}^{{q}_{1}{q}_{2}}={{}{}^{\sigma }\mathfrak{F}}_{ij}^{{q}_{1}{q}_{2}}$$

The form of the second-order CPHF equations has intentionally been cast into a form that resemble the first-order CPHF equations; as such, the same algorithms [26, 29] employed to solve the first-order CPHF equations may be applied for solution of the second-order CPHF equations.

### Derivatives of molecular properties under the MNDO formalism

The first derivatives $\frac{d\left(\Delta {H}_{f}\right)}{d{}{}^{{Z}_{A}}\alpha }$ and $\frac{d\left(\Delta {H}_{f}\right)}{d{}{}^{{Z}_{A}}{E}_{eisol}}$ are easily evaluated:

42 $$\frac{d\left(\Delta {H}_{f}\right)}{d{}{}^{{Z}_{A}}p}={k}_{conv}\left(\frac{d{E}_{el}}{d{}{}^{{Z}_{A}}p}+\frac{d{V}_{core}}{d{}{}^{{Z}_{A}}p}-{\sum }_{A}\frac{d{}{}^{{Z}_{A}}{E}_{eisol}}{d{}{}^{{Z}_{A}}p}\right)$$

43 $$\frac{d\left(\Delta {H}_{f}\right)}{d{}{}^{{Z}_{A}}\alpha }={k}_{conv}{\sum }_{C>B}\frac{d{V}_{BC}^{CRF}}{d{}{}^{{Z}_{A}}\alpha }, \frac{d\left(\Delta {H}_{f}\right)}{d{}{}^{{Z}_{A}}{E}_{eisol}}=-{k}_{conv}{n}_{{Z}_{A}}$$

If another NDDO-based semi-empirical method using the same formalism as MNDO for $\mathbf{H}$ and $\mathbf{G}$ [1–11] is required, the derivatives of $\Delta {H}_{f}$ with respect to the additional core-repulsion function parameters will depend on the expression for ${V}_{BC}^{CRF}$ and can be easily obtained via direct differentiation.

The remaining derivatives (against ${}^{{Z}_{A}}{\beta }_{s},{}{}^{{Z}_{A}}{\beta }_{p}, {}{}^{{Z}_{A}}{U}_{ss},{}{}^{{Z}_{A}}{U}_{pp}, {}{}^{{Z}_{A}}{\zeta }_{s},{}{}^{{Z}_{A}}{\zeta }_{p}$) are identical for [1–11] but will require modification for other methods (e.g. MNDO-F, OMx):

44 $$\frac{d\left({E}_{el}\right)}{d{}{}^{{Z}_{A}}p}={k}_{conv}\left[\mathrm{Tr}\left(\frac{d\mathbf{H}}{d{}{}^{{Z}_{A}}p}\mathbf{P}\right)+\frac{1}{2}\mathrm{Tr}\left({\mathbf{J}}^{{}{}^{{Z}_{A}}p}\left(\mathbf{P}\right)\mathbf{P}-{\mathbf{K}}^{{}{}^{{Z}_{A}}p}\left({}{}^{\alpha }\mathbf{P}\right){}{}^{\alpha }\mathbf{P}-{\mathbf{K}}^{{}{}^{{Z}_{A}}p}\left({}{}^{\beta }\mathbf{P}\right){}{}^{\beta }\mathbf{P}\right)\right]$$

Likewise,

45 $$\frac{{\partial }^{2}\left(\Delta {H}_{f}\right)}{\partial {}{}^{{Z}_{A}}\alpha \partial {}{}^{{Z}_{B}}\alpha }={k}_{conv}{\sum }_{D>C}\frac{{\partial }^{2}{V}_{CD}^{CRF}}{\partial {}{}^{{Z}_{A}}\alpha \partial {}{}^{{Z}_{B}}\alpha }$$

46 $$\frac{{\partial }^{2}\left(\Delta {H}_{f}\right)}{\partial {}{}^{{Z}_{A}}\alpha \partial {}{}^{{Z}_{B}}{p}_{i}}=0 \forall {}{}^{{Z}_{B}}{p}_{i}\ne {}{}^{{Z}_{B}}\alpha$$

47 $$\frac{{\partial }^{2}\left(\Delta {H}_{f}\right)}{\partial {}{}^{{Z}_{A}}{E}_{eisol}\partial {}{}^{{Z}_{B}}{p}_{i}}=0 \forall {}{}^{{Z}_{B}}{p}_{i}$$

48 $$\begin{array}{c}\frac{{\partial }^{2}\left(\Delta {H}_{f}\right)}{\partial {}{}^{{Z}_{A}}{p}_{i}\partial {}{}^{{Z}_{B}}{p}_{j}}={k}_{conv}\mathrm{Tr}\left(\frac{{d}^{2}\mathbf{H}}{d{}{}^{{Z}_{A}}{p}_{i}d{}{}^{{Z}_{B}}{p}_{j}}\mathbf{P}+\frac{d\mathbf{H}}{d{}{}^{{Z}_{A}}{p}_{i}}\frac{d\mathbf{P}}{d{}{}^{{Z}_{B}}{p}_{j}}\right)\\ \begin{array}{c}+\frac{1}{2}{k}_{conv}\mathrm{Tr}\left({\mathbf{J}}^{{}{}^{{Z}_{A}}{p}_{i}{}{}^{{Z}_{B}}{p}_{j}}\left(\mathbf{P}\right)\mathbf{P}+{\mathbf{J}}^{{}{}^{{Z}_{A}}p}\left(\frac{d\mathbf{P}}{d{}{}^{{Z}_{B}}{p}_{j}}\right)\mathbf{P}+{\mathbf{J}}^{{}{}^{{Z}_{A}}p}\left(\mathbf{P}\right)\frac{d\mathbf{P}}{d{}{}^{{Z}_{B}}{p}_{j}}\right)\\ \begin{array}{c}-\frac{1}{2}{k}_{conv}\mathrm{Tr}\left({\mathbf{K}}^{{}{}^{{Z}_{A}}{p}_{i}{}{}^{{Z}_{B}}{p}_{j}}\left({}{}^{\alpha }\mathbf{P}\right){}{}^{\alpha }\mathbf{P}+{\mathbf{K}}^{{}{}^{{Z}_{A}}p}\left(\frac{d{}{}^{\alpha }\mathbf{P}}{d{}{}^{{Z}_{B}}{p}_{j}}\right){}{}^{\alpha }\mathbf{P}+{\mathbf{K}}^{{}{}^{{Z}_{A}}p}\left({}{}^{\alpha }\mathbf{P}\right)\frac{d{}{}^{\alpha }\mathbf{P}}{d{}{}^{{Z}_{B}}{p}_{j}}\right)\\ -\frac{1}{2}{k}_{conv}\mathrm{Tr}\left({\mathbf{K}}^{{}{}^{{Z}_{A}}{p}_{i}{}{}^{{Z}_{B}}{p}_{j}}\left({}{}^{\beta }\mathbf{P}\right){}{}^{\beta }\mathbf{P}+{\mathbf{K}}^{{}{}^{{Z}_{A}}p}\left(\frac{d{}{}^{\beta }\mathbf{P}}{d{}{}^{{Z}_{B}}{p}_{j}}\right){}{}^{\beta }\mathbf{P}+{\mathbf{K}}^{{}{}^{{Z}_{A}}p}\left({}{}^{\beta }\mathbf{P}\right)\frac{d{}{}^{\beta }\mathbf{P}}{d{}{}^{{Z}_{B}}{p}_{j}}\right)\end{array}\end{array}\end{array}$$

The expressions for $\frac{d\mathbf{H}}{d{}{}^{{Z}_{A}}p}$, $\frac{{d}^{2}\mathbf{H}}{d{}{}^{{Z}_{A}}{p}_{i}d{}{}^{{Z}_{B}}{p}_{j}}$, ${\mathbf{J}}^{{}{}^{{Z}_{A}}p}\left({\varvec{\Delta}}\right)$, ${\mathbf{J}}^{{}{}^{{Z}_{A}}{p}_{i}{}{}^{{Z}_{B}}{p}_{j}}\left({\varvec{\Delta}}\right)$, ${\mathbf{K}}^{{}{}^{{Z}_{A}}p}\left({\varvec{\Lambda}}\right)$ and ${\mathbf{K}}^{{}{}^{{Z}_{A}}{p}_{i}{}{}^{{Z}_{B}}{p}_{j}}\left({\varvec{\Lambda}}\right)$ for each of the parameters ${}^{{Z}_{A}}{\beta }_{s},{}{}^{{Z}_{A}}{\beta }_{p}, {}{}^{{Z}_{A}}{U}_{ss},{}{}^{{Z}_{A}}{U}_{pp}, {}{}^{{Z}_{A}}{\zeta }_{s},{}{}^{{Z}_{A}}{\zeta }_{p}$ are detailed in the Supplementary Information.

Direct differentiation yields the first and second derivatives of the (semi-empirical) dipole moment and the energy matrix ${}^{\sigma }{\varvec{\upepsilon}}$ via which the ionization energy can be obtained:

49 $$\frac{d\langle \mu \rangle }{d{}{}^{{Z}_{A}}p}=\frac{{\varvec{\upmu}}\cdot \frac{d{\varvec{\upmu}}}{d{}{}^{{Z}_{A}}p}}{\left|{\varvec{\upmu}}\right|},$$

50 $$\frac{d\mu_\tau}{d{{}^{Z_A}}p}=-2\sum\limits_{B}\left(\frac{d{{}^{Z_B}}D_1}{d{{}^{Z_A}}p}P_{sp_\tau}+{{}^{Z_B}}D_1\frac{dP_{sp_\tau}}{d{{}^{Z_A}}p}\right)+\sum\limits_{B}{\tau_{CM,B}\left(Q_A-\sum\limits_{m\in B}\frac{dP_{mm}}{d{{}^{Z_A}}p}\right)}$$

51 $$\frac{{d}^{2}\langle \mu \rangle }{d{}{}^{{Z}_{A}}{p}_{i}d{}{}^{{Z}_{B}}{p}_{j}}=\frac{{\varvec{\upmu}}\cdot \frac{{d}^{2}{\varvec{\upmu}}}{d{}{}^{{Z}_{A}}{\zeta }_{{x}_{1}}d{}{}^{{Z}_{B}}{\zeta }_{{x}_{2}}}+\frac{d{\varvec{\upmu}}}{d{}{}^{{Z}_{B}}{p}_{j}}\cdot \frac{d{\varvec{\upmu}}}{d{}{}^{{Z}_{A}}{p}_{i}}-\frac{d\langle \mu \rangle }{d{}{}^{{Z}_{A}}{p}_{i}}\frac{d\langle \mu \rangle }{d{}{}^{{Z}_{B}}{p}_{j}}}{\left|{\varvec{\upmu}}\right|}$$

52 $$\begin{array}{c}\frac{{d}^{2}{\mu }_{\tau }}{d{}{}^{{Z}_{A}}{p}_{i}d{}{}^{{Z}_{B}}{p}_{j}}={\sum }_{B}{\tau }_{CM,B}\left({Q}_{A}-{\sum }_{m\in B}\frac{{d}^{2}{P}_{mm}}{d{}{}^{{Z}_{A}}{p}_{i}d{}{}^{{Z}_{B}}{p}_{j}}\right)\\ -2{\sum }_{C}\left(\frac{d{}{}^{{Z}_{C}}{D}_{1}}{d{}{}^{{Z}_{A}}{p}_{i}}\frac{d{P}_{s{p}_{\tau }}}{d{}{}^{{Z}_{B}}{p}_{j}}+\frac{{d}^{2}{}{}^{{Z}_{C}}{D}_{1}}{d{}{}^{{Z}_{A}}{p}_{i}d{}{}^{{Z}_{B}}{p}_{j}}{P}_{s{p}_{\tau }}+\frac{d{}{}^{{Z}_{C}}{D}_{1}}{d{}{}^{{Z}_{B}}{p}_{j}}\frac{d{P}_{s{p}_{\tau }}}{d{}{}^{{Z}_{A}}{p}_{i}}+{}{}^{{Z}_{C}}{D}_{1}\frac{{d}^{2}{P}_{s{p}_{\tau }}}{d{}{}^{{Z}_{A}}{p}_{i}d{}{}^{{Z}_{B}}{p}_{j}}\right)\end{array}$$

53 $$\frac{d{}{}^{\sigma }{\varvec{\upepsilon}}}{d{}{}^{{Z}_{A}}p}=\frac{d{{}{}^{\sigma }\mathbf{C}}^{\mathrm{T}}}{d{}{}^{{Z}_{A}}p}{}{}^{\sigma }\mathbf{F}{}{}^{\sigma }\mathbf{C}+{{}{}^{\sigma }\mathbf{C}}^{\mathrm{T}}\frac{d{}{}^{\sigma }\mathbf{F}}{d{}{}^{{Z}_{A}}p}{}{}^{\sigma }\mathbf{C}+{{}{}^{\sigma }\mathbf{C}}^{\mathrm{T}}{}{}^{\sigma }\mathbf{F}\frac{\partial {}{}^{\sigma }\mathbf{C}}{\partial {}{}^{{Z}_{A}}p}$$

54 $$\begin{array}{c}\frac{{d}^{2}{}{}^{\sigma }{\varvec{\upepsilon}}}{d{}{}^{{Z}_{A}}{p}_{i}d{}{}^{{Z}_{B}}{p}_{j}}=\frac{{d}^{2}{{}{}^{\sigma }\mathbf{C}}^{\mathrm{T}}}{d{}{}^{{Z}_{A}}{p}_{i}d{}{}^{{Z}_{B}}{p}_{j}}{}{}^{\sigma }\mathbf{F}{}{}^{\sigma }\mathbf{C}+\frac{d{{}{}^{\sigma }\mathbf{C}}^{\mathrm{T}}}{d{}{}^{{Z}_{A}}{p}_{i}}\frac{d{}{}^{\sigma }\mathbf{F}}{d{}{}^{{Z}_{B}}{p}_{j}}{}{}^{\sigma }\mathbf{C}+\frac{d{{}{}^{\sigma }\mathbf{C}}^{\mathrm{T}}}{d{}{}^{{Z}_{A}}{p}_{i}}{}{}^{\sigma }\mathbf{F}\frac{d{}{}^{\sigma }\mathbf{C}}{d{}{}^{{Z}_{B}}{p}_{j}}\\ +\frac{d{{}{}^{\sigma }\mathbf{C}}^{\mathrm{T}}}{d{}{}^{{Z}_{B}}{p}_{j}}\frac{d{}{}^{\sigma }\mathbf{F}}{d{}{}^{{Z}_{A}}{p}_{i}}{}{}^{\sigma }\mathbf{C}+{{}{}^{\sigma }\mathbf{C}}^{\mathrm{T}}\frac{d{}{}^{\sigma }\mathbf{F}}{d{}{}^{{Z}_{A}}{p}_{i}d{}{}^{{Z}_{B}}{p}_{j}}{}{}^{\sigma }\mathbf{C}+{{}{}^{\sigma }\mathbf{C}}^{\mathrm{T}}\frac{d{}{}^{\sigma }\mathbf{F}}{d{}{}^{{Z}_{A}}{p}_{i}}\frac{d{}{}^{\sigma }\mathbf{C}}{d{}{}^{{Z}_{B}}{p}_{j}}\\ +\frac{d{{}{}^{\sigma }\mathbf{C}}^{\mathrm{T}}}{d{}{}^{{Z}_{B}}{p}_{j}}{}{}^{\sigma }\mathbf{F}\frac{d{}{}^{\sigma }\mathbf{C}}{d{}{}^{{Z}_{A}}{p}_{i}}+{{}{}^{\sigma }\mathbf{C}}^{\mathrm{T}}\frac{d{}{}^{\sigma }\mathbf{F}}{d{}{}^{{Z}_{B}}{p}_{j}}\frac{d{}{}^{\sigma }\mathbf{C}}{d{}{}^{{Z}_{A}}{p}_{i}}+{{}{}^{\sigma }\mathbf{C}}^{\mathrm{T}}{}{}^{\sigma }\mathbf{F}\frac{{d}^{2}{}{}^{\sigma }\mathbf{C}}{d{}{}^{{Z}_{A}}{p}_{i}d{}{}^{{Z}_{B}}{p}_{j}}\end{array}$$

Derivatives of dipole moments are presented for the Restricted Hartree–Fock case, as no extension to UHF systems is necessary.

Lastly, the elements of $\frac{d\mathbf{g}}{d{}{}^{{Z}_{C}}p}$ (derivative of the gradient vector in Cartesian coordinates against arbitrary parameter ${}^{{Z}_{C}}p$) are given by

55 $$\frac{{d}^{2}E}{d{}{}^{A}\tau d{}{}^{{Z}_{C}}p}=\sum_{B\ne A}\frac{d}{d{}{}^{{Z}_{C}}p}\left(\frac{\partial {E}_{AB}}{\partial {}{}^{A}\tau }\right)+\frac{d}{d{}{}^{{Z}_{C}}p}\left({\sum }_{B\ne A}\frac{d{V}_{AB}^{CRF}}{d{}{}^{A}\tau }\right)$$

56 $$\begin{array}{c}\frac{d}{d{}{}^{{Z}_{C}}p}\left(\frac{\partial {E}_{AB}}{\partial {}{}^{A}\tau }\right)=\sum_{\mu ,\nu \in A}\left(\frac{d{P}_{\mu \nu }}{d{}{}^{{Z}_{C}}p}\frac{d{V}_{\mu \nu ,B}}{d{}{}^{A}\tau }+{P}_{\mu \nu }\frac{{d}^{2}{V}_{\mu \nu ,B}}{d{}{}^{A}\tau d{}{}^{{Z}_{C}}p}\right)\\ +\sum_{\lambda ,\sigma \in B}\left(\frac{d{P}_{\lambda \sigma }}{d{}{}^{{Z}_{C}}p}\frac{d{V}_{\lambda \sigma ,A}}{d{}{}^{A}\tau }+{P}_{\lambda \sigma }\frac{{d}^{2}{V}_{\lambda \sigma ,A}}{d{}{}^{A}\tau d{}{}^{{Z}_{C}}p}\right)+2\sum_{\begin{array}{c}\mu \in A\\ \lambda \in B\end{array}}\left(\frac{d{P}_{\mu \lambda }}{d{}{}^{{Z}_{C}}p}\frac{d{\beta }_{\mu \lambda }}{d{}{}^{A}\tau }+{P}_{\mu \lambda }\frac{{d}^{2}{\beta }_{\mu \lambda }}{d{}{}^{A}\tau d{}{}^{{Z}_{C}}p}\right)\\ \begin{array}{c}+\sum_{\begin{array}{c}\mu ,\nu \in A\\ \lambda ,\sigma \in B\end{array}}\left({P}_{\mu \nu }{P}_{\lambda \sigma }-{}{}^{\alpha }{P}_{\mu \nu }{}{}^{\alpha }{P}_{\lambda \sigma }-{}{}^{\beta }{P}_{\mu \nu }{}{}^{\beta }{P}_{\lambda \sigma }\right)\frac{{d}^{2}\left(\mu \nu |\lambda \sigma \right)}{d{}{}^{A}\tau d{}{}^{{Z}_{C}}p}\\ +\sum_{\begin{array}{c}\mu ,\nu \in A\\ \lambda ,\sigma \in B\end{array}}\left(\frac{d{P}_{\mu \nu }}{d{}{}^{{Z}_{C}}p}{P}_{\lambda \sigma }+{P}_{\mu \nu }\frac{d{P}_{\lambda \sigma }}{d{}{}^{{Z}_{C}}p}\right)\frac{d\left(\mu \nu |\lambda \sigma \right)}{d{}{}^{A}\tau }\\ -\sum_{\begin{array}{c}\mu ,\nu \in A\\ \lambda ,\sigma \in B\end{array}}\frac{d\left(\mu \nu |\lambda \sigma \right)}{d{}{}^{A}\tau }\sum_{\sigma \in \left\{\alpha ,\beta \right\}}\left(\frac{d{}{}^{\alpha }{P}_{\mu \nu }}{d{}{}^{{Z}_{C}}p}{}{}^{\alpha }{P}_{\lambda \sigma }+{}{}^{\alpha }{P}_{\mu \nu }\frac{d{}{}^{\alpha }{P}_{\lambda \sigma }}{d{}{}^{{Z}_{C}}p}\right)\end{array}\end{array}$$

57 $$\frac{d}{d{}{}^{{Z}_{C}}p}\left({\sum }_{B\ne A}\frac{d{V}_{AB}^{CRF}}{d{}{}^{A}\tau }\right)={\sum }_{B\ne A}\frac{{d}^{2}{V}_{AB}^{CRF}}{d{}{}^{A}\tau d{}{}^{{Z}_{C}}p}$$

Elements of $\frac{{d}^{2}\mathbf{g}}{d{}{}^{{Z}_{C}}{p}_{i}d{}{}^{{Z}_{D}}{p}_{j}}$ are likewise obtained by direct differentiation of the above expression and are omitted for brevity.

Further details on the equations in this section, where necessary, are provided in the Supplementary Information.

### Nature of the Hessian approximant in PM7

**Fig. 1:**
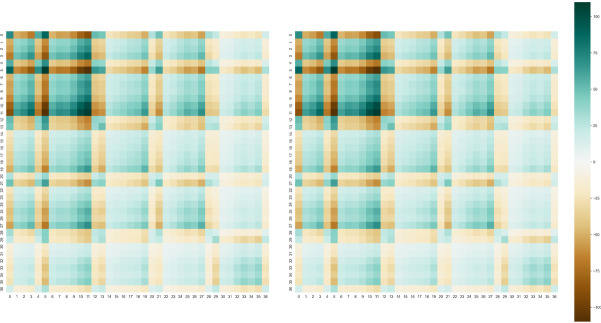
A heatmap of the elements of the exact Hessian $\mathbf{H}$ (left) and Hessian approximant ${}^{\mathrm{PM}7}\mathbf{H}$ (right), raised to the fifth root. The $37\times 37$ Hessian contains second derivatives for the MNDO parameters $\alpha ,{\beta }_{s},{\beta }_{p},{U}_{ss},{U}_{pp},{\zeta }_{s},{\zeta }_{p},{E}_{isol}$ for the elements H, C, N, O and F

To illustrate the differences between the exact Hessian $\mathbf{H}$ and the approximant ${}^{\mathrm{PM}7}\mathbf{H}$, a pictorial representation of the two matrices is given in Fig. 1. The matrices were computed using original MNDO parameters [1] for a chosen training set of 1206 molecules (see Supplementary Information).

Furthermore, the approximant ${}^{\mathrm{PM}7}\mathbf{H}$ is observed to be positive definite while $\mathbf{H}$ reflects that the parameter surface is non-convex (Table 1).

**Table 1:** Eigenvalues obtained from diagonalization of the approximant ${}^{\mathrm{PM}7}\mathbf{H}$ (left) and the exact Hessian $\mathbf{H}$ (right) using the parameters from the MNDO model. The exact reference data and weighting used to construct the error function is detailed in Table 2

No	Eigenvalue (${}^{\mathbf{P}\mathbf{M}7}\mathbf{H}$)	Eigenvalue ($\mathbf{H}$)
1	0.128368	− 512,816
2	0.934416928	− 460,096.7651
3	15.41202367	− 267,302.7955
4	30.05635772	− 216,687.9532
5	61.02869235	− 167,602.8167
6	89.83798894	− 39,421.75011
7	133.3580448	− 7503.560232
8	314.5683375	− 2616.97078
9	333.6432758	− 983.335745
10	1440.455065	− 533.6458032
11	1645.025654	− 211.742801
12	2550.646282	− 101.2912023
13	3835.543251	− 41.93718193
14	5680.70884	248.1694688
15	8639.457771	775.4405619
16	13,231.619	2083.752434
17	20,549.13091	3068.359634
18	38,971.27902	11,361.85088
19	103,579.4856	19,381.78711
20	121,144.8051	68,127.12467
21	182,104.4115	119,463.3803
22	247,015.4439	298,112.1443
23	313,366.6596	894,952.2348
24	536,170.167	1,204,079.117
25	755,156.5518	1,925,370.492
26	968,678.9509	2,107,406.892
27	1,153,754.339	2,604,117.089
28	2,096,012.221	8,556,379.32
29	3,620,532.328	8,932,577.125
30	5,794,531.367	17,840,724.68
31	12,084,613.12	38,505,843.45
32	38,639,528.07	72,107,717.07
33	187,190,499.4	190,046,809.8
34	446,920,022	450,868,384.7
35	509,186,668.9	534,566,784.3
36	854,723,315.4	862,489,769.1
37	47,330,423,728	47,359,707,042

## Methods for parameter optimization

In PM7, parameter optimization is performed via an approximated line search, with the search direction obtained via a direct Hessian descent (HD) step on the approximated Hessian [5]:

58 $$\hat{\mathbf{d}}=-\frac{{{}^{PM7}}\mathbf{H}^{-1}\mathbf{g}\ }{\left|{{}^{PM7}}\mathbf{H}^{-1}\mathbf{g}\ \right|},\ \ \left|\mathbf{d}\right|={\text{argmax}}_k\left(\widetilde{\mathcal{S}}|_{\mathbf{p}=\mathbf{p}_0+k\hat{\mathbf{d}}}\right)$$

59 $$\widetilde{\mathcal{S}}=\sum_{\alpha }{\mathcal{C}}_{\alpha }^{2}{\left({\xi }_{\alpha }^{\mathrm{ref}}-{\xi }_{\alpha }{\left.\right|}_{\mathbf{p}={\mathbf{p}}_{0}}-\sum_{{Z}_{A}}\sum_{{}{}^{{Z}_{A}}{p}_{i}}{\left.\frac{d{\xi }_{\alpha }}{d{}{}^{{Z}_{A}}{p}_{i}}\right|}_{\mathbf{p}={\mathbf{p}}_{0}}\left({}{}^{{Z}_{A}}{p}_{i}{\left.\right|}_{\mathbf{p}}-{}{}^{{Z}_{A}}{p}_{i}{\left.\right|}_{\mathbf{p}={\mathbf{p}}_{0}}\right)\right)}^{2}$$

This method of determining the step size shall be termed the approximated line search (ALS) method; an alternative would be a trust radius (TR), where the step size is constrained by a dynamic trust radius. The modification of the trust radius in our attempt is given by the following computation, with $\mathbf{B}$ representing an arbitrary Hessian or Hessian approximant:

60 $${Q}_{n}={\mathbf{d}}_{n}^{\mathrm{T}}{\mathbf{g}}_{n}+\frac{1}{2}{\mathbf{d}}_{n}^{\mathrm{T}}{\mathbf{B}}_{n}{\mathbf{d}}_{n}, {\rho }_{n}=\frac{\mathcal{S}{\left.\boldsymbol{}\right|}_{\mathbf{p}={\mathbf{p}}_{n}+{\mathbf{d}}_{n}}-\mathcal{S}{\left.\boldsymbol{}\right|}_{\mathbf{p}={\mathbf{p}}_{n}}}{{Q}_{n}}$$

61 $$\left|{\mathbf{d}}_{n}\right|={R}_{n}, {R}_{n+1}=\left\{\begin{array}{c}\frac{5}{4}{R}_{n}, {\rho }_{n}>0.8 and S{\left.\boldsymbol{}\right|}_{\mathbf{p}={\mathbf{p}}_{n}+{\mathbf{d}}_{n}}<S{\left.\boldsymbol{}\right|}_{\mathbf{p}={\mathbf{p}}_{n}}\\ \frac{1}{2}{R}_{n}, {\rho }_{n}<0.25 or S{\left.\boldsymbol{}\right|}_{\mathbf{p}={\mathbf{p}}_{n}+{\mathbf{d}}_{n}}\ge S{\left.\boldsymbol{}\right|}_{\mathbf{p}={\mathbf{p}}_{n}}\\ {R}_{n}, otherwise\end{array}\right.$$

In addition to determination of $\widehat{\mathbf{d}}$ via direct Hessian descent, a trust region optimization (TRO) was also attempted, where $\mathbf{d}$ is computed via:

62 $$\mathbf{d}={\left(\mathbf{B}-\lambda \mathbf{I}\right)}^{-1}\mathbf{g},\left|\mathbf{d}\right|=R$$

The shift parameter $\lambda$ is computed iteratively, with the chosen numerical method detailed in the Supplementary Information.

Lastly, three different choices for the second-derivative matrix $\mathbf{B}$ were investigated. In addition to the exact Hessian $\mathbf{H}$ and the approximant ${}^{\mathrm{PM}7}\mathbf{H}$, a modified Hessian ${\mathbb{H}}$ was found to yield promising results:

63 $${\mathbb{H}}=\mathbf{P}{\mathbf{D}}^{\mathbf{^{\prime}}}{\mathbf{P}}^{-1},\boldsymbol{ }\boldsymbol{ }{\mathbf{D}}_{ii}^{\mathbf{^{\prime}}}=\left|{\mathbf{D}}_{ii}\right|,\mathbf{H}=\mathbf{P}\mathbf{D}{\mathbf{P}}^{-1}$$

The modified Hessian ${\mathbb{H}}$ preserves the eigenvectors of the exact Hessian while converting it to be positive (semi)definite, and ensures that a direct Hessian descent step will not traverse in the uphill direction given a surface of the wrong concavity.

**Fig. 2:**
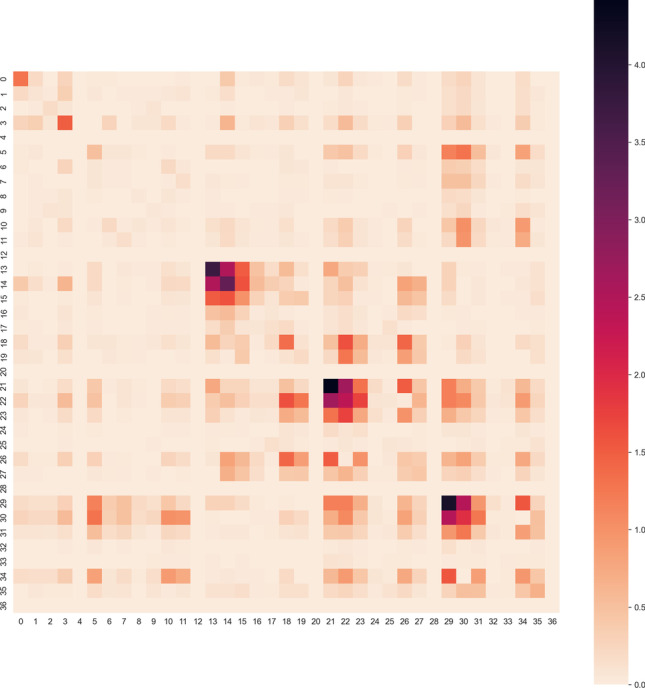
A heatmap of percentage errors of each Hessian element, calculated as $\left|\frac{{{}^{\mathrm{PM}7}\mathbf{H}}_{ij}-{\mathbf{H}}_{ij}}{{\mathbf{H}}_{ij}}\right|\times 100\%$

It should be noted that ${}^{\mathrm{PM}7}\mathbf{H}$ is constructed without any second derivatives of molecular properties, and hence, optimization with ${}^{\mathrm{PM}7}\mathbf{H}$ is comparable to other methods using approximate Hessians that do not evaluate second derivative information (e.g. the BFGS or DFP schemes); however, ${}^{\mathrm{PM}7}\mathbf{H}$ appears to provide a reasonably good estimate for $\mathbf{H}$ despite having incorrect eigenvalue information (as seen in Fig. 2, the percentage errors for specific elements in the Hessian matrix are at most 4–5%) and is thus expected to perform better than a regular quasi-Newton optimizer. We note in passing that PM3 and PM6 were optimized with the method outlined in [5] using a DFP update scheme while the more recent PM7 [10] was optimized using ${}^{\mathrm{PM}7}\mathbf{H}$, suggesting that ${}^{\mathrm{PM}7}\mathbf{H}$ better approximates the exact Hessian $\mathbf{H}$.

The computation of the gradient $\mathbf{g}$ is straightforward and may be obtained easily by both finite difference and analytic differentiation; this suggests that gradient-free optimization methods would not be necessary in optimizing parameters for NDDO-based methods. Such local optimizers can, however, be combined with other methods to identify global minima, e.g. genetic algorithms [30, 31], and may be indicative of possible avenues for further work on this subject. We defer a discussion of the nature of local and global minima on the parameter surface to the “Conclusion”.

## Results of limited parameterisation

As a proof of concept, a limited parameterization of 1206 molecules consisting of the atom types C, H, N, O, F was performed using the MNDO formalism; the relevant routines for the semi-empirical evaluation of molecular properties using MNDO were correspondingly implemented and compared against MOPAC [32] for accuracy (see the Supplementary Information). Geometrical data, e.g., bond angles or bond lengths, are accounted for in our parameterization procedure by using the norm of the gradient vector $\left|\mathbf{g}\right|$ at a reference geometry (either experimental or the result of high-level calculations), with the corresponding reference function set to zero to simulate a perfect correspondence between the semi-empirical and reference geometries; this was chosen to facilitate the ease of preparing the relevant inputs. The weighting functions ${\mathcal{C}}_{i}$ for the error function were chosen as specified in Table 2.

**Table 2:** Weighting factors ${\mathcal{C}}_{i}$ for the reference properties used in parameterization. The weighting factors for $\Delta {H}_{f}$, $I.E.$ and $\langle \mu \rangle$ are the same as in the PMx methods

Property	Units	${\mathcal{C}}_{{\varvec{i}}}$
${\varvec{\Delta}}{{\varvec{H}}}_{{\varvec{f}}}$	$\mathrm{kcal}/\mathrm{mol}$	$1\mathrm{ mol}/\mathrm{kcal}$
${\varvec{I}}.{\varvec{E}}.$	$\mathrm{eV}$	$10 {\mathrm{eV}}^{-1}$
$\langle {\varvec{\mu}}\rangle$	$\mathrm{D}$	$20 {\mathrm{D}}^{-1}$
$\left|\mathbf{g}\right|$	$\mathrm{kcal}/(\mathrm{mol}\cdot \mathrm{bohr})$	$0.5\mathrm{ mol}\cdot \mathrm{bohr}/\mathrm{kcal}$

While additional properties such as reaction barriers were also viable choices for inclusion as reference data in our training set, such information was not used in our limited parameterization; nonetheless, the parameter derivatives of the molecular energy used in calculating derivatives of $\Delta {H}_{f}$ can be applied to calculate the relevant parameter derivatives for the energies of transition states, allowing for an easy extension if necessary.

For all optimization methods, the graphs shown below are terminated once the decrease in $\mathcal{S}$ is no longer appreciable; the optimization runs discussed in this section hence do not represent the identification of minima on the parameter surface.

**Fig. 3:**
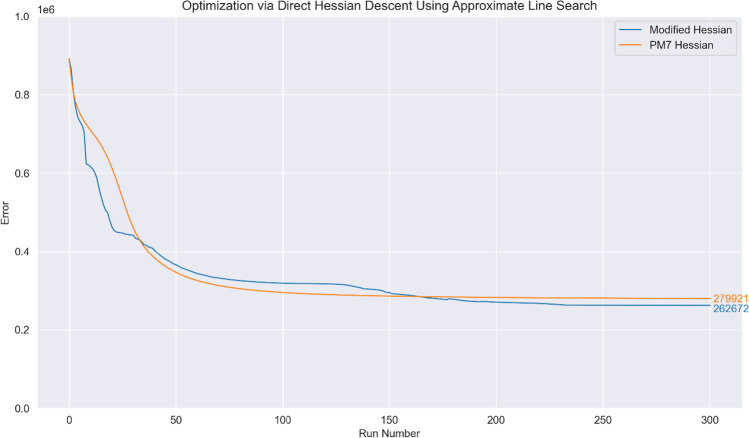
Optimization curve obtained when using the parameterization procedure detailed in [5] and [10]

**Fig. 4:**
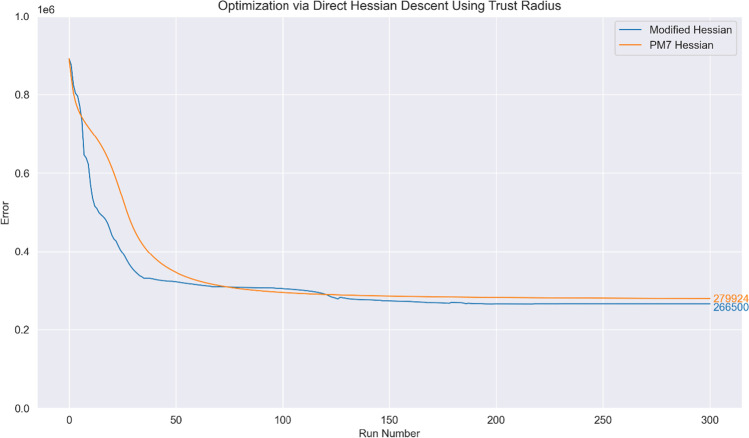
Optimization curve obtained when using a trust radius with the direct Hessian descent (Newton–Raphson) step. The initial step size at run no. 0 is set at 2.0 for ${\mathbb{H}}$ and ${}^{\mathrm{PM}7}\mathbf{H}$

**Fig. 5:**
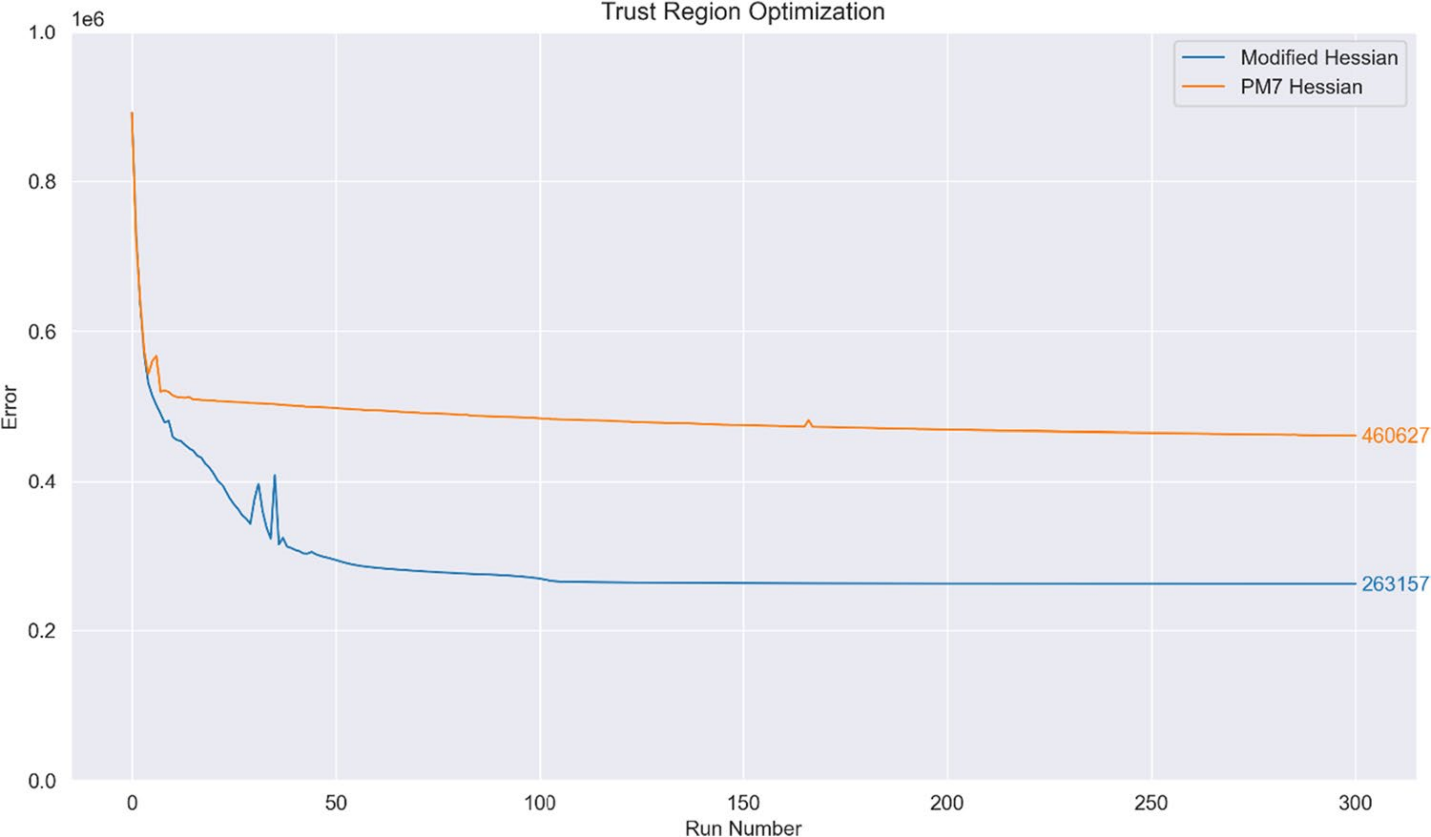
Optimization curve obtained when using a trust region optimizer. The initial step size at run no. 0 is set at 0.1 for both curves

First, application of the optimization method used in the PMx models (Hessian descent with step size determined by the approximate line search) leads to surprisingly similar behaviour when both ${\mathbb{H}}$ and ${}^{\mathrm{PM}7}\mathbf{H}$ are used; nonetheless, there is an appreciable difference in the final parameter values for the two methods. Direct employment of the Hessian matrix $\mathbf{H}$ results in a poor optimisation procedure as the evaluated step size shrinks significantly around $\mathcal{S}=575000$; this is expected as the line-search procedure seeks to minimise $\mathcal{S}$ even as the Newton–Raphson step traverses uphill for nonconvex regions. The optimization curves for $\mathbf{H}$ are hence omitted in Figs. 3, 4 and 5.

To compare the line-search method used in the PMx models with a trust radius method, optimization using direct Hessian descent but with the step size determined via a dynamic trust radius was attempted. We note in passing that the resultant optimization curve strongly resembles that of the according line-search method (cf. Figure 3); once again, direct employment of $\mathbf{H}$ leads to very poor optimisation, and the resultant curve has been omitted:

Lastly, optimization was attempted with a trust region optimizer; this led to similar performance for ${\mathbb{H}}$ while ${}^{\mathrm{PM}7}\mathbf{H}$ performed markedly poorer. Optimization with $\mathbf{H}$ led to suboptimal parameters that encountered problems with SCF convergence and geometry optimization and was hence abandoned.

Optimization with ${}^{\mathrm{PM}7}\mathbf{H}$ appears to result in the identification of a saddle point, which is incorrectly identified as a minimum due to the positive-definite nature of ${}^{\mathrm{PM}7}\mathbf{H}$; the Hessian eigenvalues (from the exact Hessian $\mathbf{H}$) for the resultant parameters obtained via optimization with ${}^{\mathrm{PM}7}\mathbf{H}$ are given in Table 3.

**Table 3:** Eigenvalues taken from diagonalizing the exact Hessian using parameters from run no. 300 of optimization with ${}^{\mathrm{PM}7}\mathbf{H}$ (corresponding to the end point of the green curve in Fig. 5)

No	Eigenvalue	No	Eigenvalue	No	Eigenvalue
1	− 41.234	14	6818.55467	27	1,512,211.907
2	− 3.823761	15	8866.28568	28	2,277,598.426
3	− 2.9794242	16	19,648.5226	29	4,749,192.867
4	7.18597596	17	34,039.3551	30	11,509,664.69
5	42.2832925	18	42,849.9713	31	20,200,473.71
6	53.6581437	19	122,256.195	32	37,260,138.2
7	114.412963	20	166,404.131	33	146,715,815.1
8	351.125533	21	219,075.729	34	359,303,765.9
9	778.324332	22	233,887.679	35	404,915,300.3
10	923.225124	23	328,171.073	36	771,116,378.5
11	1804.53062	24	499,506.976	37	39,386,113,276
12	3531.45311	25	878,679.923		
13	4324.88892	26	1,287,823.88		

**Fig. 6:**
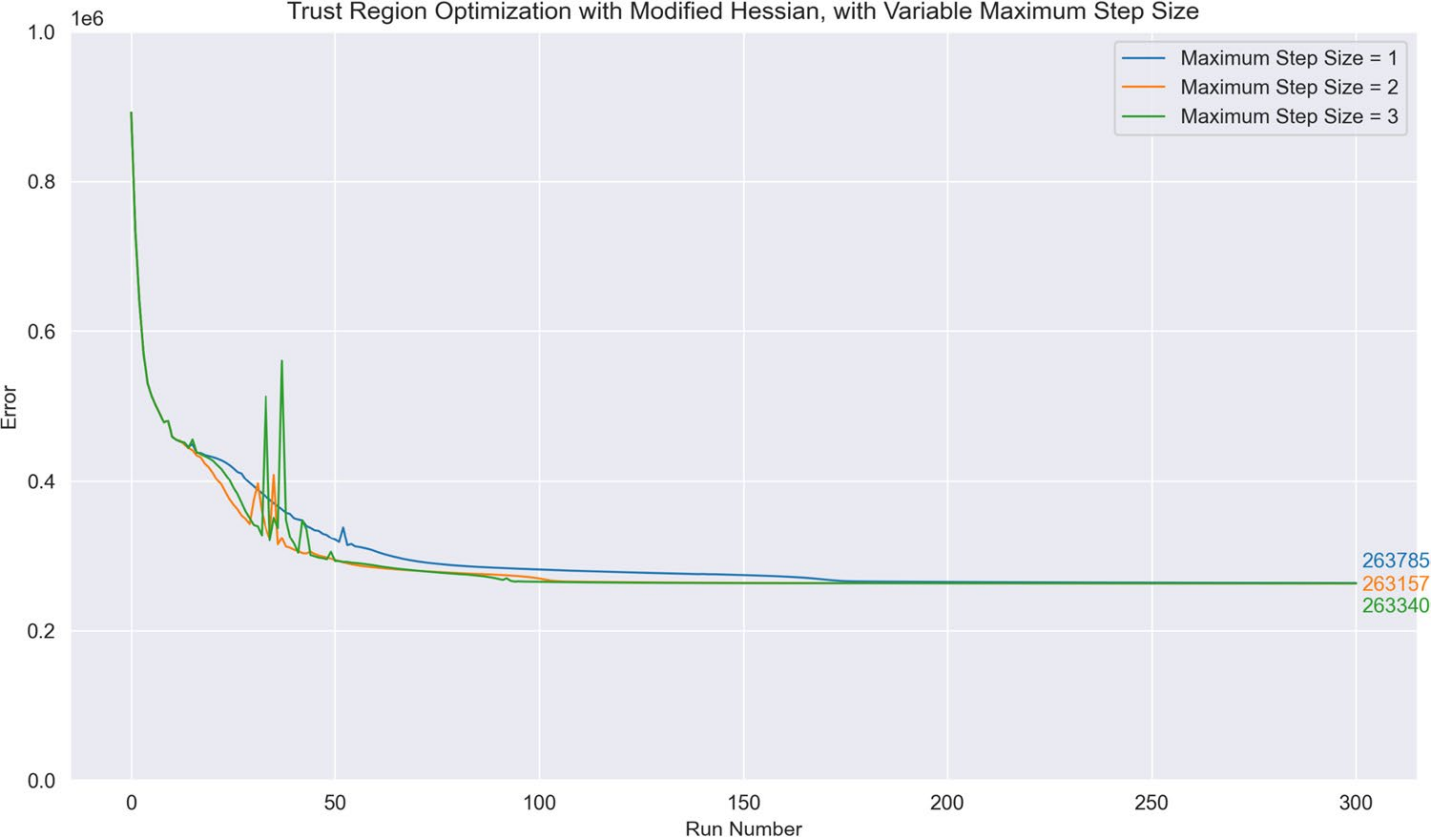
Optimization curves obtained when using a trust region optimizer and the modified Hessian ${\mathbb{H}}$. The initial step size at run no. 0 is set at 0.1 for all three curves, but the maximum step size ${\left|\mathbf{d}\right|}_{max}$ was varied

In all methods (Hessian descent with both line search and trust radius determination of step size as well as the trust-region optimizer), the step size is constrained to a maximum of ${\left|\mathbf{d}\right|}_{max}=2$ to ensure that there are no significant and unexpected increases in the error function. We note in passing that the choice of ${\left|\mathbf{d}\right|}_{max}=2$, while arbitrary, does not play a significant role in the optimization of parameters; choices of ${\left|\mathbf{d}\right|}_{max}=1$ and ${\left|\mathbf{d}\right|}_{max}=3$ lead to very similar optimization curves (Fig. 6).

In the course of parameterization, it was realized that identification of a true local minimum would be difficult and require manual intervention in the parameterization procedure; thus, any set of parameters with a reasonably small gradient vector magnitude, a positive definite (exact) Hessian matrix and a marked resistance to further reduction in the error function should be accepted as a reasonable result from parameter optimization.

In this work, we report the identification of a local minimum from our limited parameterization, obtained with the imposition of no constraints on the parameters. The parameters, as well as the values of the associated gradient element, for an identified minimum with $\mathcal{S}=261996$ are given in Table 4; the associated Hessian eigenvalues are presented in Table 5.

**Table 4:** Top and bottom: parameter values and their associated elements of the gradient vector used in parameter optimization for the parameters obtained at the end of our limited optimization

Parameter	Element				
	C	H	N	O	F
${}^{{{\varvec{Z}}}_{{\varvec{A}}}}\boldsymbol{\alpha }$	2.660499	2.993408	3.021163	3.334554	3.590480
${}^{{{\varvec{Z}}}_{{\varvec{A}}}}{{\varvec{\beta}}}_{{\varvec{s}}}$	− 13.719865	− 7.334656	− 17.522551	− 73.616234	− 1753.340685
${}^{{{\varvec{Z}}}_{{\varvec{A}}}}{{\varvec{\beta}}}_{{\varvec{p}}}$	− 6.970837	NIL	− 15.550446	− 22.327739	− 22.382738
${}^{{{\varvec{Z}}}_{{\varvec{A}}}}{{\varvec{U}}}_{{\varvec{s}}{\varvec{s}}}$	− 47.292652	− 10.314472	− 64.905273	− 94.106196	− 107.380890
${}^{{{\varvec{Z}}}_{{\varvec{A}}}}{{\varvec{U}}}_{{\varvec{p}}{\varvec{p}}}$	− 40.221274	NIL	− 57.638287	− 78.565181	− 106.952003
${}^{{{\varvec{Z}}}_{{\varvec{A}}}}{{\varvec{\zeta}}}_{{\varvec{s}}}$	2.326858	1.124161	2.914488	5.594470	50.167806
${}^{{{\varvec{Z}}}_{{\varvec{A}}}}{{\varvec{\zeta}}}_{{\varvec{p}}}$	1.637021	NIL	2.126141	2.425014	2.463961
${}^{{{\varvec{Z}}}_{{\varvec{A}}}}{{\varvec{E}}}_{{\varvec{i}}{\varvec{s}}{\varvec{o}}{\varvec{l}}}$	− 112.533538	− 11.423363	− 189.262258	− 309.024988	− 434.411284

Gradient	Element				
	C	H	N	O	F
${}^{{{\varvec{Z}}}_{{\varvec{A}}}}\boldsymbol{\alpha }$	− 5631.2989	− 122.511277	− 265.68094	− 170.18825	− 1319.4913
${}^{{{\varvec{Z}}}_{{\varvec{A}}}}{{\varvec{\beta}}}_{{\varvec{s}}}$	122.967088	39.777392	4.0270245	0.92212293	1.65447933
${}^{{{\varvec{Z}}}_{{\varvec{A}}}}{{\varvec{\beta}}}_{{\varvec{p}}}$	284.128795	NIL	14.8445277	14.6824465	110.38728
${}^{{{\varvec{Z}}}_{{\varvec{A}}}}{{\varvec{U}}}_{{\varvec{s}}{\varvec{s}}}$	264.36961	51.0183803	29.0786636	40.8640734	655.636142
${}^{{{\varvec{Z}}}_{{\varvec{A}}}}{{\varvec{U}}}_{{\varvec{p}}{\varvec{p}}}$	655.367166	NIL	55.1677978	94.2737931	2154.95912
${}^{{{\varvec{Z}}}_{{\varvec{A}}}}{{\varvec{\zeta}}}_{{\varvec{s}}}$	1724.84129	97.0883125	57.8930721	36.7178843	86.2475113
${}^{{{\varvec{Z}}}_{{\varvec{A}}}}{{\varvec{\zeta}}}_{{\varvec{p}}}$	4822.82283	NIL	297.807752	334.865333	1932.25505
${}^{{{\varvec{Z}}}_{{\varvec{A}}}}{{\varvec{E}}}_{{\varvec{i}}{\varvec{s}}{\varvec{o}}{\varvec{l}}}$	− 245.1164	− 66.13715	− 17.695251	− 21.930875	− 391.28695

**Table 5:** Eigenvalues taken from diagonalizing the exact Hessian using the parameters obtained at the end of our limited parameterization

No	Eigenvalue	No	Eigenvalue	No	Eigenvalue
1	0.000307191	14	7097.672134	27	1,169,583.719
2	2.110409103	15	11,248.65204	28	4,374,828.93
3	9.894574497	16	13,983.01	29	4,455,736.981
4	39.09862218	17	27,423.51093	30	9,638,553.272
5	88.84495043	18	31,607.2681	31	21,918,237.20
6	120.8695565	19	87,137.37687	32	32,920,532.04
7	418.3575145	20	180,143.5721	33	104,814,193.86
8	460.8171646	21	209,812.6169	34	241,208,268.54
9	755.3529097	22	324,571.6458	35	294,810,061.12
10	1256.25836	23	416,778.2783	36	555,086,343.83
11	2304.566797	24	555,533.5715	37	33,782,858,461.66
12	3297.414171	25	688,452.7501		
13	3864.018903	26	1,078,765.379		

While clearly unphysical, these parameters indicate a position close to a local minimum on the parameter surface and are an indication of the significant deficiencies of the MNDO model in modelling charged or radical species.

Notably, ammonia is predicted to be planar using these parameters; as the non-planarity of ammonia was not a constraint imposed upon the system for parameterization, this is not an erroneous result. A detailed tabulation of the predicted molecular properties at the given parameters is provided in the Supplementary Information.

**Fig. 7:**
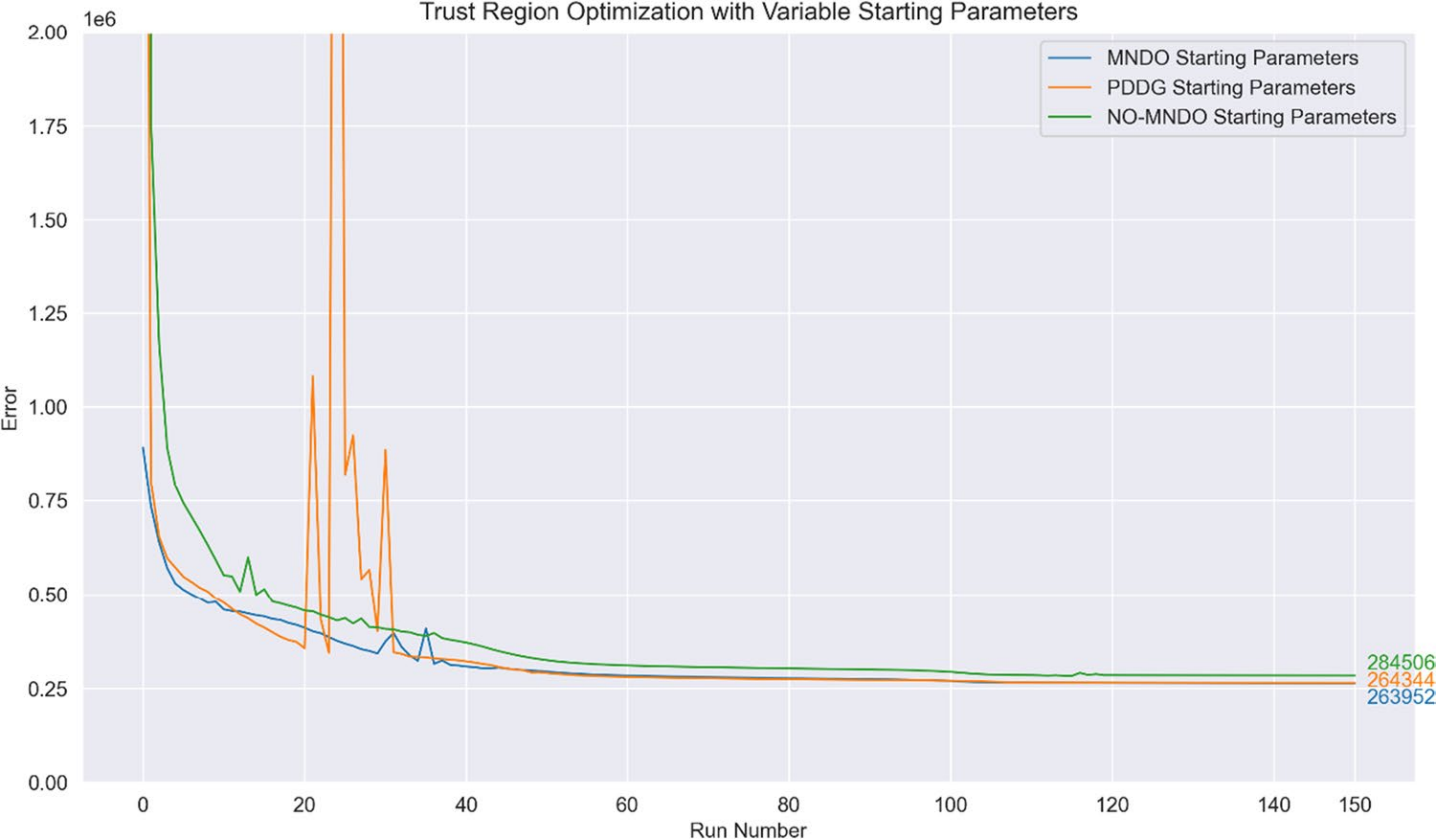
Optimization curves obtained when using a trust region optimizer and the modified Hessian ${\mathbb{H}}$. The initial step size at run no. 0 is set at 0.1 for all three curves and ${\left|\mathbf{d}\right|}_{max}=2$, but the initial parameters used were varied (see Tables 6 and 7)

In Fig. 7, optimization using ${\mathbb{H}}$ with initial step size 0.1 and ${\left|\mathbf{d}\right|}_{max}=2$ is compared for three sets of starting parameters: the original MNDO parameters, the MNDO parameters reported for PDDG/MNDO [11] and the parameters for the elements C, H, N and O reported in NO-MNDO [22] alongside original MNDO parameters for fluorine (see Tables 6 and 7). Since the formalism used remains that of MNDO and not PDDG/MNDO or NO-MNDO, these additional sets of parameters are expected to perform worse than the original MNDO parameters; however, they provide an interesting case study for how our local optimizer might behave when starting from different initial parameter values. The graph is terminated after 150 optimization runs, and the final error function values obtained are hence not reflective of the identification of a local minimum; nonetheless, the very similar error function values despite the significant differences in final parameters (as reported in Table 8) seem to indicate that there may be numerous local minima with similar error function values.

**Table 6:** Initial parameter values obtained from PDDG/MNDO (see Fig. 7)

Parameter	Element				
	C	H	N	O	F
${}^{{{\varvec{Z}}}_{{\varvec{A}}}}\boldsymbol{\alpha }$	2.555522	2.491813	2.843678	3.238842	3.322382
${}^{{{\varvec{Z}}}_{{\varvec{A}}}}{{\varvec{\beta}}}_{{\varvec{s}}}$	− 18.841334	− 7.493504	− 20.375774	− 33.606336	− 67.827612
${}^{{{\varvec{Z}}}_{{\varvec{A}}}}{{\varvec{\beta}}}_{{\varvec{p}}}$	− 7.922234	NIL	− 21.085373	− 27.984442	− 40.924818
${}^{{{\varvec{Z}}}_{{\varvec{A}}}}{{\varvec{U}}}_{{\varvec{s}}{\varvec{s}}}$	− 53.837582	− 11.724114	− 71.871894	− 97.88497	− 134.22038
${}^{{{\varvec{Z}}}_{{\varvec{A}}}}{{\varvec{U}}}_{{\varvec{p}}{\varvec{p}}}$	− 39.936409	NIL	− 58.216617	− 77.342674	− 107.15596
${}^{{{\varvec{Z}}}_{{\varvec{A}}}}{{\varvec{\zeta}}}_{{\varvec{s}}}$	1.809817	1.322431	2.231424	2.569172	4.328519
${}^{{{\varvec{Z}}}_{{\varvec{A}}}}{{\varvec{\zeta}}}_{{\varvec{p}}}$	1.825008	NIL	2.25346	2.697152	2.905042
${}^{{{\varvec{Z}}}_{{\varvec{A}}}}{{\varvec{E}}}_{{\varvec{i}}{\varvec{s}}{\varvec{o}}{\varvec{l}}}$	− 123.86441	− 12.015956	− 206.46663	− 310.87975	− 488.70324

**Table 7:** Initial parameter values obtained from NO-MNDO (see Fig. 7)

Parameter	Element				
	C	H	N	O	F
${}^{{{\varvec{Z}}}_{{\varvec{A}}}}\boldsymbol{\alpha }$	2.48446	2.687705	2.658599	2.946645	3.4196606
${}^{{{\varvec{Z}}}_{{\varvec{A}}}}{{\varvec{\beta}}}_{{\varvec{s}}}$	− 16.208034	− 9.364858	− 24.90552	− 35.477596	− 48.290466
${}^{{{\varvec{Z}}}_{{\varvec{A}}}}{{\varvec{\beta}}}_{{\varvec{p}}}$	− 10.637421	NIL	− 21.291958	− 28.881783	− 36.50854
${}^{{{\varvec{Z}}}_{{\varvec{A}}}}{{\varvec{U}}}_{{\varvec{s}}{\varvec{s}}}$	− 50.189763	− 10.880363	− 69.782951	− 96.705658	− 131.07155
${}^{{{\varvec{Z}}}_{{\varvec{A}}}}{{\varvec{U}}}_{{\varvec{p}}{\varvec{p}}}$	− 39.547267	NIL	− 56.981889	− 76.391762	− 105.78214
${}^{{{\varvec{Z}}}_{{\varvec{A}}}}{{\varvec{\zeta}}}_{{\varvec{s}}}$	1.925428	1.061597	2.351138	2.455548	2.848487
${}^{{{\varvec{Z}}}_{{\varvec{A}}}}{{\varvec{\zeta}}}_{{\varvec{p}}}$	1.727933	NIL	1.951819	2.537964	2.848487
${}^{{{\varvec{Z}}}_{{\varvec{A}}}}{{\varvec{E}}}_{{\varvec{i}}{\varvec{s}}{\varvec{o}}{\varvec{l}}}$	− 119.5944	− 13.160122	− 202.2436	− 304.34129	− 476.68378

**Table 8:** Top, middle and bottom: parameter values after 150 optimization runs starting from MNDO parameters, PDDG/MNDO parameters and NO-MNDO parameters (see Tables 6 and 7)

MNDO parameter	Element				
	C	H	N	O	F
${}^{{{\varvec{Z}}}_{{\varvec{A}}}}\boldsymbol{\alpha }$	2.65893028	2.98616999	3.01789735	3.33094136	3.59661896
${}^{{{\varvec{Z}}}_{{\varvec{A}}}}{{\varvec{\beta}}}_{{\varvec{s}}}$	− 13.896133	− 7.3703509	− 17.576889	− 72.876223	− 219.98053
${}^{{{\varvec{Z}}}_{{\varvec{A}}}}{{\varvec{\beta}}}_{{\varvec{p}}}$	− 6.9485023	NIL	− 15.611441	− 22.291261	− 20.757317
${}^{{{\varvec{Z}}}_{{\varvec{A}}}}{{\varvec{U}}}_{{\varvec{s}}{\varvec{s}}}$	− 47.280029	− 10.315317	− 64.95198	− 94.806035	− 107.24866
${}^{{{\varvec{Z}}}_{{\varvec{A}}}}{{\varvec{U}}}_{{\varvec{p}}{\varvec{p}}}$	− 40.191348	NIL	− 57.601922	− 78.551712	− 106.91534
${}^{{{\varvec{Z}}}_{{\varvec{A}}}}{{\varvec{\zeta}}}_{{\varvec{s}}}$	2.33907785	1.12519887	2.90566782	5.48159077	12.7783341
${}^{{{\varvec{Z}}}_{{\varvec{A}}}}{{\varvec{\zeta}}}_{{\varvec{p}}}$	1.62963659	NIL	2.12324781	2.42135448	2.39933061
${}^{{{\varvec{Z}}}_{{\varvec{A}}}}{{\varvec{E}}}_{{\varvec{i}}{\varvec{s}}{\varvec{o}}{\varvec{l}}}$	− 112.50774	− 11.42606	− 189.30868	− 310.3944	− 434.01522

PDDG/MNDO parameter	Element				
	C	H	N	O	F
${}^{{{\varvec{Z}}}_{{\varvec{A}}}}\boldsymbol{\alpha }$	2.66899203	2.92239613	3.02052461	3.33649311	3.58643208
${}^{{{\varvec{Z}}}_{{\varvec{A}}}}{{\varvec{\beta}}}_{{\varvec{s}}}$	− 13.972814	− 7.364351	− 16.728601	− 72.368252	− 230.56023
${}^{{{\varvec{Z}}}_{{\varvec{A}}}}{{\varvec{\beta}}}_{{\varvec{p}}}$	− 6.8267709	NIL	− 15.800279	− 22.29721	− 21.980605
${}^{{{\varvec{Z}}}_{{\varvec{A}}}}{{\varvec{U}}}_{{\varvec{s}}{\varvec{s}}}$	− 47.306366	− 10.336626	− 64.273955	− 95.398437	− 107.30999
${}^{{{\varvec{Z}}}_{{\varvec{A}}}}{{\varvec{U}}}_{{\varvec{p}}{\varvec{p}}}$	− 40.14483	NIL	− 57.523819	− 78.507984	− 106.77862
${}^{{{\varvec{Z}}}_{{\varvec{A}}}}{{\varvec{\zeta}}}_{{\varvec{s}}}$	2.35713045	1.18520195	2.94159025	5.44939528	13.2748539
${}^{{{\varvec{Z}}}_{{\varvec{A}}}}{{\varvec{\zeta}}}_{{\varvec{p}}}$	1.62332729	NIL	2.12477097	2.42716075	2.46038129
${}^{{{\varvec{Z}}}_{{\varvec{A}}}}{{\varvec{E}}}_{{\varvec{i}}{\varvec{s}}{\varvec{o}}{\varvec{l}}}$	− 112.36726	− 11.193921	− 187.80396	− 311.39522	− 433.37951

NO-MNDO parameter	Element				
	C	H	N	O	F
${}^{{{\varvec{Z}}}_{{\varvec{A}}}}\boldsymbol{\alpha }$	2.66382164	2.98279164	3.03799145	3.35980247	3.71454355
${}^{{{\varvec{Z}}}_{{\varvec{A}}}}{{\varvec{\beta}}}_{{\varvec{s}}}$	− 13.386239	− 7.3096246	− 18.540266	− 82.349631	− 185.91367
${}^{{{\varvec{Z}}}_{{\varvec{A}}}}{{\varvec{\beta}}}_{{\varvec{p}}}$	− 6.999786	NIL	− 15.011623	− 21.616282	− 16.258881
${}^{{{\varvec{Z}}}_{{\varvec{A}}}}{{\varvec{U}}}_{{\varvec{s}}{\varvec{s}}}$	− 47.243051	− 10.247595	− 65.176426	− 92.97953	− 105.84392
${}^{{{\varvec{Z}}}_{{\varvec{A}}}}{{\varvec{U}}}_{{\varvec{p}}{\varvec{p}}}$	− 40.187542	NIL	− 57.741407	− 78.760165	− 107.38397
${}^{{{\varvec{Z}}}_{{\varvec{A}}}}{{\varvec{\zeta}}}_{{\varvec{s}}}$	2.30677182	1.14028611	3.00201012	6.13082666	12.0426399
${}^{{{\varvec{Z}}}_{{\varvec{A}}}}{{\varvec{\zeta}}}_{{\varvec{p}}}$	1.64820504	NIL	2.09568357	2.38054219	2.14285707
${}^{{{\varvec{Z}}}_{{\varvec{A}}}}{{\varvec{E}}}_{{\varvec{i}}{\varvec{s}}{\varvec{o}}{\varvec{l}}}$	− 112.2377	− 11.304432	− 190.16538	− 307.79559	− 434.22304

## Conclusion

In this paper, we report a fully analytic differentiation routine for the evaluation of parameter derivatives for construction of the parameter gradient and Hessian in MNDO-based semi-empirical methods and have applied these equations for a proof-of-concept optimization of the MNDO parameters for the elements C, H, N, O and F. While the PM7 Hessian ${}^{\mathrm{PM}7}\mathbf{H}$ appears to work remarkably well in parameterization schemes, it does not guarantee that the identified stationary point on the parameter surface will be a local minimum; furthermore, ${}^{\mathrm{PM}7}\mathbf{H}$ does appear to perform worse when the optimization nears a local minimum or stationary point. The full Hessian $\mathbf{H}$ may be necessary for optimization near the stationary point, and the accurate eigenvalue information may also be helpful in ensuring that a local minimum is attained at the termination of parameterization.

We have placed significant emphasis on the identification of local minima on the parameter surface. While it is conceded that identification of global minima is of greater concern in parameterization, we note that parameter surfaces encountered in the optimization of neural networks often show multiple local minima, all with similar loss function values [33] and posit that a similar situation may be observed in NDDO-based methods.

## Supplementary Information

Below is the link to the electronic supplementary material.

Supplementary file1 (DOCX 377 KB)

Supplementary file2 (TXT 1435 KB)

## Data Availability

The relevant data in this paper shall be provided upon request.
