# Supplementary material for: An improved parameterization procedure for NDDO-descendant semi-empirical methods
Source: J Mol Model. 2023 Mar 28;29(4):118. doi: 10.1007/s00894-023-05499-3 (PMC10050048; doi:10.1007/s00894-023-05499-3)
Supplement: Supplementary file 1 — Supplementary file1 (DOCX 377 KB) [file 894_2023_5499_MOESM1_ESM.docx]

An Improved Parameterization Procedure for NDDO-Descendant Semiempirical Methods: Supplementary Information

**First Derivatives of Heats of Formation**

First derivatives of heats of formation do not require any first-order response terms; differentiation of the expression for $\Delta H_{f}$ yields the compact equation that

$$\begin{aligned} \frac{d\left( \Delta H_{f} \right)}{d{}^{Z_{A}}p}=k_{conv}\left( \frac{dE_{el}}{d{}^{Z_{A}}p}+\frac{dV_{core}}{d{}^{Z_{A}}p}-\sum_{A} \frac{d{}^{Z_{A}}{E_{eisol}}}{d{}^{Z_{A}}p} \right)\#\left( S1 \right) \end{aligned}$$

The value $k_{conv}$ represents the conversion factor between the various terms (evaluated in $\mathrm{eV}$) and the value $\Delta H_{f}$ (in $kcal/mol$).

Thus, the derivatives $\frac{d\left( \Delta H_{f} \right)}{d{}^{Z_{A}}\alpha}$ and $\frac{d\left( \Delta H_{f} \right)}{d{}^{Z_{A}}{E_{eisol}}}$ are easily evaluated:

$$\begin{aligned} \frac{d\left( \Delta H_{f} \right)}{d{}^{Z_{A}}\alpha}=k_{conv}\sum_{C>B} \frac{dV_{BC}^{CRF}}{d{}^{Z_{A}}\alpha}, \frac{d\left( \Delta H_{f} \right)}{d{}^{Z_{A}}{E_{eisol}}}=-k_{conv}n_{Z_{A}}\#\left( S2 \right) \end{aligned}$$

In the above expression, $n_{Z_{A}}$ represents the stoichiometric ratio for the element in question (e.g. $n_{Z_{6}}=6$ in cyclohexane, $C_{6}H_{12}$).

The remaining derivatives (against ${}^{Z_{A}}{\beta_{s}},{}^{Z_{A}}{\beta_{p}}, {}^{Z_{A}}{U_{ss}},{}^{Z_{A}}{U_{pp}}, {}^{Z_{A}}{\zeta_{s}},{}^{Z_{A}}{\zeta_{p}}$) are significantly more complex, and requires particular attention:

$$\begin{aligned} \frac{d\left( \Delta H_{f} \right)}{d{}^{Z_{A}}{\beta_{x}}}=2k_{conv}\sum_{\begin{aligned} B \\ C>B \end{aligned}} \sum_{\begin{aligned} \mu\in B \\ \nu\in C \end{aligned}} P_{\mu\nu}\frac{d\beta_{\mu\nu}}{d{}^{Z_{A}}{\beta_{x}}}, \frac{d\beta_{\mu\nu}}{d{}^{Z_{A}}{\beta_{x}}}=\frac{\left( \delta_{\beta_{\mu}{}^{Z_{A}}{\beta_{x}}}+\delta_{\beta_{\nu}{}^{Z_{A}}{\beta_{x}}} \right)S_{\mu\nu}}{2}\#\left( S3a,b \right) \end{aligned}$$

$$\begin{aligned} \frac{d\left( \Delta H_{f} \right)}{d{}^{Z_{A}}{U_{xx}}}=k_{conv}\sum_{\mu} P_{\mu\mu}\delta_{U_{\mu\mu}{}^{Z_{A}}{U_{xx}}}\#\left( S4 \right) \end{aligned}$$

Alternatively, one may employ the matrices $\frac{\partial\mathbf{H}}{\partial{}^{Z_{A}}p}$, $\mathbf{J}^{{}^{Z_{A}}p}\left( \mathbf{P} \right)$ and $\mathbf{K}^{{}^{Z_{A}}p}\left( {}^{\sigma}\mathbf{P} \right)$ to evaluate $\frac{\partial E_{el}}{\partial{}^{Z_{A}}p}$ accordingly, with the final expression given by:

$$\begin{aligned} \frac{d\left( E_{el} \right)}{d{}^{Z_{A}}p}=k_{conv}\left[ \mathrm{Tr}\left( \frac{d\mathbf{H}}{d{}^{Z_{A}}p}\mathbf{P} \right)+\frac{1}{2}\mathrm{Tr}\left( \mathbf{J}^{{}^{Z_{A}}p}\left( \mathbf{P} \right)\mathbf{P-}\mathbf{K}^{{}^{Z_{A}}p}\left( {}^{\alpha}\mathbf{P} \right){}^{\alpha}\mathbf{P}-\mathbf{K}^{{}^{Z_{A}}p}\left( {}^{\beta}\mathbf{P} \right){}^{\beta}\mathbf{P} \right) \right]\#\left( S5 \right) \end{aligned}$$

The expressions for such an approach are given by:

$$\begin{aligned} \frac{dH_{\mu\nu}}{d{}^{Z_{A}}{\beta_{x}}}=\left\{ \begin{aligned} \frac{d\beta_{\mu\nu}}{d{}^{Z_{A}}{\beta_{x}}}, \mu\in C,\nu\in B\neq C \\ \\ 0, \mathrm{otherwise} \end{aligned} \right.\#\left( S6 \right) \end{aligned}$$

$$\begin{aligned} \frac{dH_{\mu\nu}}{d{}^{Z_{A}}{U_{xx}}}=\left\{ \begin{aligned} \delta_{U_{\mu\mu}{}^{Z_{A}}{U_{xx}}}, \mu=\nu\\ \\ 0, \mathrm{otherwise} \end{aligned} \right.\#\left( S7 \right) \end{aligned}$$

$$\begin{aligned} \mathbf{J}^{{}^{Z_{A}}{\beta_{x}}}\left( \mathbf{P} \right)\boldsymbol{=}\mathbf{J}^{{}^{Z_{A}}{U_{xx}}}\left( \mathbf{P} \right)\boldsymbol{=}\mathbf{K}^{{}^{Z_{A}}{\beta_{x}}}\left( {}^{\sigma}\mathbf{P} \right)\boldsymbol{=}\mathbf{K}^{{}^{Z_{A}}{U_{xx}}}\left( {}^{\sigma}\mathbf{P} \right)\boldsymbol{=0\#}\left( S8 \right) \end{aligned}$$

$$\begin{aligned} \frac{dH_{\mu\nu}}{d{}^{Z_{A}}{\zeta_{x}}}=\left\{ \begin{aligned} \sum_{C\neq B} \frac{dV_{\mu\nu,C}}{d{}^{Z_{A}}{\zeta_{x}}}, \mu,\nu\in B \\ \\ \\ \frac{d\beta_{\mu\nu}}{d{}^{Z_{A}}{\zeta_{x}}}, \mu\in C,\nu\in B\neq C \end{aligned} \right.\#\left( S9 \right) \end{aligned}$$

In the above equations,

$$\begin{aligned} \frac{dV_{\mu\nu,C}}{d{}^{Z_{A}}{\zeta_{x}}}=-Q_{A}Q_{B}\frac{d\left( \mu\nu| s^{C}s^{C} \right)}{d{}^{Z_{A}}{\zeta_{x}}}, \frac{d\beta_{\mu\nu}}{d{}^{Z_{A}}{\zeta_{x}}}=\frac{\beta_{\mu}+\beta_{\nu}}{2}\frac{dS_{\mu\nu}}{d{}^{Z_{A}}{\zeta_{x}}}\#\left( S10a, b \right) \end{aligned}$$

$$\begin{aligned} J_{\mu\nu}^{{}^{Z_{A}}{\zeta_{x}}}\left( \mathbf{P} \right)=\left\{ \begin{aligned} \sum_{\lambda, \sigma} \sum_{\in C} P_{\lambda\sigma}\frac{d\left( \mu\nu| \lambda\sigma\right)}{d{}^{Z_{A}}{\zeta_{x}}}, \mu,\nu\in B \\ \\ \\ 0, \mu\in B,\nu\in C\neq B \end{aligned} \right.\#\left( S11 \right) \end{aligned}$$

$$\begin{aligned} K_{\mu\nu}^{{}^{Z_{A}}{\zeta_{x}}}\left( {}^{\sigma}\mathbf{P} \right)=\left\{ \begin{aligned} 0, \mu,\nu\in B \\ \\ \\ \sum_{\lambda\in B} \sum_{\sigma\in C} {{}^{\sigma}P}_{\lambda\sigma}\frac{d\left( \mu\lambda| \nu\sigma\right)}{d{}^{Z_{A}}{\zeta_{x}}}, \mu\in B,\nu\in C\neq B \end{aligned} \right.\#\left( S12 \right) \end{aligned}$$

**Second Derivatives of Heats of Formation**

Further differentiation of the expression for $\Delta H_{f}$ yields

$$\begin{aligned} \frac{\partial^{2}}{\partial{{}^{Z_{A}}p}_{i}\partial{{}^{Z_{B}}p}_{j}}\left( \Delta H_{f} \right)=k_{conv}\left( \frac{\partial^{2}E_{el}}{\partial{{}^{Z_{A}}p}_{i}\partial{{}^{Z_{B}}p}_{j}}+\frac{\partial^{2}V_{core}}{\partial{{}^{Z_{A}}p}_{i}\partial{{}^{Z_{B}}p}_{j}}-\sum_{A} \frac{\partial^{2}{}^{Z_{A}}{E_{eisol}}}{\partial{{}^{Z_{A}}p}_{i}\partial{{}^{Z_{B}}p}_{j}} \right)\#\left( S13 \right) \end{aligned}$$

Thus, the derivative $\frac{\partial^{2}\left( \Delta H_{f} \right)}{\partial{}^{Z_{A}}\alpha\partial{}^{Z_{B}}\alpha}$ is easily evaluated:

$$\begin{aligned} \frac{\partial^{2}\left( \Delta H_{f} \right)}{\partial{}^{Z_{A}}\alpha\partial{}^{Z_{B}}\alpha}=k_{conv}\sum_{D>C} \frac{\partial^{2}V_{CD}^{CRF}}{\partial{}^{Z_{A}}\alpha\partial{}^{Z_{B}}\alpha}\#\left( S14 \right) \end{aligned}$$

Furthermore,

$$\begin{aligned} \frac{\partial^{2}\left( \Delta H_{f} \right)}{\partial{}^{Z_{A}}\alpha\partial{}^{Z_{B}}{p_{i}}}=0 \forall{}^{Z_{B}}{p_{i}}\neq{}^{Z_{B}}\alpha\#\left( S15 \right) \end{aligned}$$

$$\begin{aligned} \frac{\partial^{2}\left( \Delta H_{f} \right)}{\partial{}^{Z_{A}}{E_{eisol}}\partial{}^{Z_{B}}{p_{i}}}=0 \forall{}^{Z_{B}}{p_{i}}\#\left( S16 \right) \end{aligned}$$

Most static second derivatives of the heats of formation are zero. The non-zero static second derivative matrices are given below:

$$\begin{aligned} \frac{\partial^{2}H_{\mu\nu}}{\partial{}^{Z_{A}}{\beta_{x}}\partial{}^{Z_{B}}{\zeta_{x}}}=\left\{ \begin{aligned} \frac{\partial^{2}\beta_{\mu\nu}}{\partial{}^{Z_{A}}{\beta_{x}}\partial{}^{Z_{B}}{\zeta_{x}}} , \mu\in C,\nu\in D\neq C \\ \\ 0, \mathrm{otherwise} \end{aligned} \right.\#\left( S17 \right) \end{aligned}$$

$$\begin{aligned} \frac{\partial^{2}H_{\mu\nu}}{\partial{}^{Z_{A}}{\zeta_{x_{1}}}\partial{}^{Z_{B}}{\zeta_{x_{2}}}}=\left\{ \begin{aligned} \sum_{D\neq C} \frac{\partial^{2}V_{\mu\nu,D}}{\partial{}^{Z_{A}}{\zeta_{x}}\partial{}^{Z_{B}}{\zeta_{x}}}, \mu,\nu\in C \\ \\ \\ \frac{\partial^{2}\beta_{\mu\nu}}{\partial{}^{Z_{A}}{\zeta_{x_{1}}}\partial{}^{Z_{B}}{\zeta_{x_{2}}}}, \mu\in C,\nu\in D\neq C \end{aligned} \right.\#\left( S18 \right) \end{aligned}$$

In the above,

$$\begin{aligned} \frac{\partial^{2}\beta_{\mu\nu}}{\partial{}^{Z_{A}}{\beta_{x}}\partial{}^{Z_{B}}{\zeta_{x}}}=\frac{\left( \delta_{\beta_{\mu}{}^{Z_{A}}{\beta_{x}}}+\delta_{\beta_{\nu}{}^{Z_{A}}{\beta_{x}}} \right)}{2}\frac{\partial S_{\mu\nu}}{\partial{}^{Z_{B}}{\zeta_{x}}} \#\left( S19a \right) \end{aligned}$$

$$\begin{aligned} \frac{\partial^{2}V_{\mu\nu,D}}{\partial{}^{Z_{A}}{\zeta_{x}}\partial{}^{Z_{B}}{\zeta_{x}}}=-Q_{C}Q_{D}\frac{\partial^{2}\left( \mu\nu| s^{D}s^{D} \right)}{\partial{}^{Z_{A}}{\zeta_{x}}\partial{}^{Z_{B}}{\zeta_{x}}} \#\left( S19b \right) \end{aligned}$$

Lastly, the nonzero derivatives of the two-electron matrices are given by

$$\begin{aligned} J_{\mu\nu}^{{}^{Z_{A}}{\zeta_{x_{1}}}{}^{Z_{B}}{\zeta_{x_{2}}}}\left( \mathbf{P} \right)=\left\{ \begin{aligned} \sum_{\lambda, \sigma} \sum_{\in D} P_{\lambda\sigma}\frac{d^{2}\left( \mu\nu| \lambda\sigma\right)}{d{}^{Z_{A}}{\zeta_{x_{1}}}\partial{}^{Z_{B}}{\zeta_{x_{2}}}}, \mu,\nu\in C \\ \\ \\ 0, \mu\in C,\nu\in D\neq C \end{aligned} \right.\#\left( S20 \right) \end{aligned}$$

$$\begin{aligned} K_{\mu\nu}^{{}^{Z_{A}}{\zeta_{x_{1}}}{}^{Z_{B}}{\zeta_{x_{2}}}}\left( {}^{\sigma}\mathbf{P} \right)=\left\{ \begin{aligned} 0, \mu,\nu\in C \\ \\ \\ \sum_{\lambda\in C} \sum_{\sigma\in D} {{}^{\sigma}P}_{\lambda\sigma}\frac{d^{2}\left( \mu\lambda| \nu\sigma\right)}{d{}^{Z_{A}}{\zeta_{x_{1}}}\partial{}^{Z_{B}}{\zeta_{x_{2}}}}, \mu\in C,\nu\in D\neq C \end{aligned} \right.\#\left( S21 \right) \end{aligned}$$

The second derivatives of the heat of formation are evaluated using the expression:

$$\begin{aligned} \frac{\partial^{2}\left( \Delta H_{f} \right)}{\partial{}^{Z_{A}}{p_{i}}\partial{}^{Z_{B}}{p_{j}}}=k_{conv}\mathrm{Tr}\left( \frac{d^{2}\mathbf{H}}{d{}^{Z_{A}}{p_{i}}d{}^{Z_{B}}{p_{j}}}\mathbf{P+}\frac{d\mathbf{H}}{d{}^{Z_{A}}{p_{i}}}\frac{d\mathbf{P}}{d{}^{Z_{B}}{p_{j}}} \right) \\ +\frac{1}{2}k_{conv}\mathrm{Tr}\left( \mathbf{J}^{{}^{Z_{A}}{p_{i}}{}^{Z_{B}}{p_{j}}}\left( \mathbf{P} \right)\mathbf{P+}\mathbf{J}^{{}^{Z_{A}}p}\left( \frac{d\mathbf{P}}{d{}^{Z_{B}}{p_{j}}} \right)\mathbf{P+}\mathbf{J}^{{}^{Z_{A}}p}\left( \mathbf{P} \right)\frac{d\mathbf{P}}{d{}^{Z_{B}}{p_{j}}} \right) \\ -\frac{1}{2}k_{conv}\mathrm{Tr}\left( \mathbf{K}^{{}^{Z_{A}}{p_{i}}{}^{Z_{B}}{p_{j}}}\left( {}^{\alpha}\mathbf{P} \right){}^{\alpha}\mathbf{P}\mathbf{+}\mathbf{K}^{{}^{Z_{A}}p}\left( \frac{d{}^{\alpha}\mathbf{P}}{d{}^{Z_{B}}{p_{j}}} \right){}^{\alpha}\mathbf{P}\mathbf{+}\mathbf{K}^{{}^{Z_{A}}p}\left( {}^{\alpha}\mathbf{P} \right)\frac{d{}^{\alpha}\mathbf{P}}{d{}^{Z_{B}}{p_{j}}} \right) \\ -\frac{1}{2}k_{conv}\mathrm{Tr}\left( \mathbf{K}^{{}^{Z_{A}}{p_{i}}{}^{Z_{B}}{p_{j}}}\left( {}^{\beta}\mathbf{P} \right){}^{\beta}\mathbf{P}\mathbf{+}\mathbf{K}^{{}^{Z_{A}}p}\left( \frac{d{}^{\beta}\mathbf{P}}{d{}^{Z_{B}}{p_{j}}} \right){}^{\beta}\mathbf{P}\mathbf{+}\mathbf{K}^{{}^{Z_{A}}p}\left( {}^{\beta}\mathbf{P} \right)\frac{d{}^{\beta}\mathbf{P}}{d{}^{Z_{B}}{p_{j}}} \right) \#\left( S22 \right) \end{aligned}$$

**First and Second Derivatives of Dipole Moments**

The semiempirical dipole moment is formally evaluated as the magnitude of a 3-element dipole moment vector:

$$\begin{aligned} \left\langle\mu\right\rangle=\left| \boldsymbol{\mu} \right|\#\left( S23 \right) \end{aligned}$$

$$\begin{aligned} \mu_{\tau}=-2\sum_{B} {}^{Z_{B}}{D_{1}}P_{sp_{\tau}}+\sum_{B} \tau_{CM,B}\left( Q_{A}-\sum_{m\in B} P_{mm} \right)\#\left( S24 \right) \end{aligned}$$

The distance $\tau_{CM,B}$ denotes the projection of the vector $\mathbf{r}_{B}\boldsymbol{-}\mathbf{r}_{CM}$ onto the axis $\tau(=x,y,z)$; the centre-of-mass position vector $\mathbf{r}_{CM}$ is evaluated using standard empirical measurements for atomic masses.

Direct differentiation yields the first and second derivatives of the dipole moment, which are presented in the main text and hence omitted from the Supplementary Information.

**First and Second Derivatives of Reference Geometry Gradients**

The derivative $\frac{d\left| \mathbf{g} \right|}{d{}^{Z_{C}}p}$ can be easily cast in terms of the derivative vector $\frac{d\mathbf{g}}{d{}^{Z_{C}}p}$:

$$\begin{aligned} \frac{d\left| \mathbf{g} \right|}{d{}^{Z_{C}}p}=\frac{\boldsymbol{g\cdot}\frac{d\mathbf{g}}{d{}^{Z_{C}}p}}{\left| \mathbf{g} \right|}\#\left( S25 \right) \end{aligned}$$

Likewise,

$$\begin{aligned} \frac{d^{2}\left| \mathbf{g} \right|}{d{}^{Z_{C}}{p_{i}}d{}^{Z_{D}}{p_{j}}}=\frac{\frac{d\mathbf{g}}{d{}^{Z_{C}}{p_{i}}}\boldsymbol{\cdot}\frac{d\mathbf{g}}{d{}^{Z_{D}}{p_{j}}}\boldsymbol{+g\cdot}\frac{d^{2}\mathbf{g}}{d{}^{Z_{C}}{p_{i}}d{}^{Z_{D}}{p_{j}}}-\frac{d\left| \mathbf{g} \right|}{d{}^{Z_{C}}{p_{i}}}\frac{d\left| \mathbf{g} \right|}{d{}^{Z_{D}}{p_{j}}}}{\left| \mathbf{g} \right|}\#\left( S26 \right) \end{aligned}$$

The elements of the reference geometry gradient $\mathbf{g}$ are evaluated as follows:

$$\begin{aligned} \frac{dE}{d{}^{A}\tau}=\sum_{B\neq A} \frac{\partial E_{AB}}{\partial{}^{A}\tau}+\sum_{B\neq A} \frac{dV_{AB}^{CRF}}{d{}^{A}\tau}\#\left( S27 \right) \end{aligned}$$

$$\begin{aligned} \frac{\partial E_{AB}}{\partial{}^{A}\tau}=\sum_{\mu,\nu\in A} P_{\mu\nu}\frac{dV_{\mu\nu,B}}{d{}^{A}\tau}+\sum_{\lambda,\sigma\in B} P_{\lambda\sigma}\frac{dV_{\lambda\sigma,A}}{d{}^{A}\tau}+2\sum_{\begin{aligned} \mu\in A \\ \lambda\in B \end{aligned}} P_{\mu\lambda}\frac{d\beta_{\mu\lambda}}{d{}^{A}\tau} \\ +\sum_{\begin{aligned} \mu,\nu\in A \\ \lambda,\sigma\in B \end{aligned}} \left( P_{\mu\nu}P_{\lambda\sigma}-{}^{\alpha}{P_{\mu\nu}}{}^{\alpha}{P_{\lambda\sigma}}-{}^{\beta}{P_{\mu\nu}}{}^{\beta}{P_{\lambda\sigma}} \right)\frac{d\left( \mu\nu| \lambda\sigma\right)}{d{}^{A}\tau}\#\left( S28 \right) \end{aligned}$$

$$\begin{aligned} \frac{d\beta_{\mu\lambda}}{d{}^{A}\tau}=\frac{\beta_{\mu}+\beta_{\lambda}}{2}\frac{dS_{\mu\lambda}}{d{}^{A}\tau}\#\left( S29 \right) \end{aligned}$$

Elements of $\frac{d\mathbf{g}}{d{}^{Z_{C}}p}$ and $\frac{d^{2}\mathbf{g}}{d{}^{Z_{C}}{p_{i}}d{}^{Z_{D}}{p_{j}}}$ are obtained via direct differentiation of the above expression.

**The Trust Region Optimizer**

In a quadratic trust region optimiser, we seek to optimise the Lagrangian function

$$\begin{aligned} \mathcal{L=}\mathbf{g}^{T}\mathbf{d+}\frac{1}{2}\mathbf{d}^{T}\mathbf{Bd+}\kappa\left( \mathbf{d}^{T}\mathbf{d}-R^{2} \right) \#\left( S30 \right) \end{aligned}$$

Differentiation hence yields an expression for the Hessian shift parameter $\lambda$ as a function of the Lagrange multiplier $\kappa$:

$$\begin{aligned} \mathbf{d=-}\left( \mathbf{B+}2\kappa\mathbf{I} \right)^{-1}\mathbf{g=-}\left( \mathbf{B+}\lambda\mathbf{I} \right)^{-1}\mathbf{g}\boldsymbol{\#}\left( S31 \right) \end{aligned}$$

By constraining the step length, we hence obtain an iterative algorithm for $\lambda$:

$$\begin{aligned} \Phi=1-\frac{\sqrt{\mathbf{d}^{T}\mathbf{d}}}{R}=0\#\left( S32 \right) \end{aligned}$$

$$\begin{aligned} \lambda_{n+1}=\lambda_{n}+\frac{\Phi}{\frac{1}{2R\sqrt{\mathbf{d}^{T}\mathbf{d}}}\frac{d\left( \mathbf{d}^{T}\mathbf{d} \right)}{d\lambda}}=\lambda_{n}+\frac{2\left( R\sqrt{\mathbf{d}^{T}\mathbf{d}}-\mathbf{d}^{T}\mathbf{d} \right)}{\frac{d\left( \mathbf{d}^{T}\mathbf{d} \right)}{d\lambda}}\#\left( S33a \right) \end{aligned}$$

$$\begin{aligned} \frac{d\left( \mathbf{d}^{T}\mathbf{d} \right)}{d\lambda}=-2\sum_{i} \frac{\left( \mathbf{u}_{i}^{T}\mathbf{g} \right)^{2}}{\left( B_{ii}\boldsymbol{+}\lambda\right)^{3}}\#\left( S33b \right) \end{aligned}$$

In the above, $\mathbf{u}_{i}$ represents the eigenvectors of the Hessian $\mathbf{B}$.

**Training Set for Limited Parameterization**

The training set employed for our limited parameterization is provided as *training_set.txt*.

**Comparison of Our Program to MOPAC**

Calculated Values for $\Delta H_{f}$ Using the MNDO Formalism (given in $kcal/mol$)

| **Molecular Formula** | **Molecule Name** | **MOPAC** | **Our Program** |
| --- | --- | --- | --- |
| H | Hydrogen, cation | 326.7 | 326.7 |
| H | Hydrogen, atom | 52.1 | 52.1 |
| H2 | Hydrogen | 0.7 | 0.7 |
| C | Carbon, cation | 389.4 | 389.4 |
| C | Carbon, atom | 170.9 | 170.9 |
| CH | Methylidyne | 143.3 | 143.3 |
| CH2 | Methylene, singlet | 107.4 | 107.4 |
| CH2 | Methylene, triplet | 73.9 | 73.9 |
| CH3 | Methyl, cation | 243.9 | 243.9 |
| CH4 | Methane | -12 | -12.0 |
| C2H2 | Acetylene | 57.9 | 57.9 |
| C2H3 | Vinyl, cation | 265.7 | 265.7 |
| C2H3 | Vinyl | 59 | 59.0 |
| C2H4 | Ethylene, cation | 237.7 | 237.7 |
| C2H4 | Ethylene | 15.4 | 15.4 |
| C2H4 | Methylmethylene | 88.3 | 88.3 |
| C2H5 | Ethyl, cation | 219.6 | 219.6 |
| C2H5 | Ethyl radical | 10.5 | 10.5 |
| C2H6 | Ethane | -19.7 | -19.8 |
| C3 | Carbon, trimer | 220.3 | 220.3 |
| C3H3 | Cyclopropenyl, cation | 272.5 | 272.5 |
| C3H3 | Propynyl, cation | 265.4 | 265.4 |
| C3H4 | Allene | 43.9 | 43.9 |
| C3H4 | Cyclopropene | 68.3 | 68.3 |
| C3H4 | Propyne | 41.4 | 41.4 |
| C3H5 | Allyl, cation | 221.4 | 221.4 |
| C3H5 | Cyclopropyl, cation | 258.1 | 258.1 |
| C3H5 | Propenyl, cation | 240.1 | 240.1 |
| C3H5 | Allyl | 25.3 | 25.3 |
| C3H6 | Cyclopropane | 11.2 | 11.2 |
| C3H6 | Propene | 4.9 | 4.9 |
| C3H7 | i-Propyl radical | -1.6 | -1.6 |
| C3H8 | Propane | -25 | -25.0 |
| C4 | Carbon, tetramer | 271.3 | 271.3 |
| C4H2 | Diacetylene | 103.2 | 103.2 |
| C4H4 | Vinylacetylene | 65.5 | 65.5 |
| C4H4 | Butatriene | 71.2 | 71.2 |
| C4H6 | 1,2-Butadiene | 33.5 | 33.5 |
| C4H6 | 1,3-Butadiene | 28.9 | 28.9 |
| C4H6 | 1-Butyne | 36.1 | 36.1 |
| C4H6 | 2-Butyne | 24.8 | 24.9 |
| C4H6 | Bicyclobutane | 64 | 64.0 |
| C4H6 | Cyclobutene | 31 | 31.0 |
| C4H6 | Methyl cyclopropene | 53.7 | 53.7 |
| C4H6 | Methylenecyclopropane | 37.8 | 37.8 |
| C4H7 | 2-Butenyl, cation | 206.9 | 206.9 |
| C4H7 | Cyclobutyl, cation | 221.4 | 221.3 |
| C4H8 | 1-Butene | 0.3 | -0.3 |
| C4H8 | cis-2-Butene | -4.4 | -4.4 |
| C4H8 | Cyclobutane | -11.9 | -12.0 |
| C4H8 | Isobutene | -2 | -2.0 |
| C4H8 | trans-2-Butene | -5.1 | -5.1 |
| C4H9 | Isobutyl, cation | 187.9 | 187.3 |
| C4H9 | Isobutyl | -10.1 | -10.1 |
| C4H10 | Isobutane | -26.8 | -26.8 |
| C4H10 | n-Butane, trans | -29.8 | -29.8 |
| C5H5 | Cyclopentadienyl, anion | 18.9 | 18.9 |
| C5H6 | Cyclopentadiene | 32 | 32.0 |
| C5H8 | 1,2-Dimethyl cyclopropene | 39.2 | 39.3 |
| C5H8 | 1,4-Pentadiene | 24.5 | 24.5 |
| C5H8 | 1,cis-3-Pentadiene | 20 | 20.0 |
| C5H8 | 1,trans-3-Pentadiene | 19.1 | 19.1 |
| C5H8 | Bicyclo(2.1.0)-pentane | 30.1 | 30.1 |
| C5H8 | Cyclopentene | -0.4 | -0.4 |
| C5H8 | Isoprene | 23.3 | 23.3 |
| C5H8 | Methylene cyclobutane | 10.8 | 10.8 |
| C5H8 | Spiropentane | 33.6 | 33.6 |
| C5H9 | Cyclopentyl, cation | 194.1 | 194.1 |
| C5H10 | 2-Methyl-2-butene | -10.2 | -10.2 |
| C5H10 | cis-2-Pentene | -8.9 | -8.9 |
| C5H10 | cis-Dimethylcyclopropane | -2.2 | -2.2 |
| C5H10 | Cyclopentane | -30.5 | -30.6 |
| C5H10 | trans-2-Pentene | -10.3 | -10.4 |
| C5H12 | n-Pentane | -34.5 | -34.5 |
| C5H12 | Neopentane | -24.7 | -24.7 |
| C6H6 | Benzene | 21.2 | 21.3 |
| C6H6 | Fulvene | 53.6 | 53.6 |
| C6H8 | (E)-1,3,5-Hexatriene | 42.5 | 42.5 |
| C6H8 | (Z)-1,3,5-Hexatriene | 43.8 | 43.8 |
| C6H8 | 1,3-Cyclohexadiene | 14.4 | 14.4 |
| C6H8 | 1,4-Cyclohexadiene | 14.3 | 14.3 |
| C6H10 | 1,2-Dimethylcyclobutene | 6.3 | 6.3 |
| C6H10 | 1,5-Hexadiene | 19.6 | 19.5 |
| C6H10 | 1-Methyl cyclopentene | -10.3 | -10.3 |
| C6H10 | 3-Methyl cyclopentene | -3.7 | -3.7 |
| C6H10 | 4-Methyl cyclopentene | -3.1 | -3.1 |
| C6H10 | Bicyclopropyl | 28.5 | 28.5 |
| C6H10 | Cyclohexene | -10 | -10.0 |
| C6H11 | Cyclohexyl, cation | 186.8 | 186.8 |
| C6H12 | 1-Hexene | -9.1 | -9.1 |
| C6H12 | 2,3-Dimethyl-1-butene | -7.3 | -7.3 |
| C6H12 | 2,3-Dimethyl-2-butene | -13.3 | -13.2 |
| C6H12 | (Z)-3-Methyl-2-pentene | -12.5 | -12.4 |
| C6H12 | 4-Methyl-1-pentene | -5.8 | -5.9 |
| C6H12 | Cyclohexane | -34.8 | -34.8 |
| C6H14 | 2,2-Dimethyl butane | -26.1 | -26.1 |
| C6H14 | 2,3-Dimethyl butane | -27.8 | -27.8 |
| C6H14 | 2-Methyl pentane | -34.6 | -34.7 |
| C6H14 | 3-Methyl pentane | -33 | -33.1 |
| C6H14 | n-Hexane | -39.2 | -39.2 |
| C7H7 | Benzyl, cation | 218 | 218.0 |
| C7H7 | Tropylium cation | 207.6 | 207.6 |
| C7H8 | Cycloheptatriene | 33.7 | 33.8 |
| C7H8 | Norbornadiene | 62.8 | 62.8 |
| C7H8 | Toluene | 13.5 | 13.5 |
| C7H12 | 1,2-Dimethyl cyclopentene | -18.5 | -18.7 |
| C7H12 | 1-Ethyl cyclopentene | -14 | -14.0 |
| C7H12 | 1-Methyl cyclohexene | -17.1 | -17.1 |
| C7H12 | Norbornane | -10.5 | -10.6 |
| C7H14 | 1,1-Dimethyl cyclopentane | -30.1 | -30.1 |
| C7H14 | 1,2-cis-Dimethyl cyclopentane | -32.4 | -32.4 |
| C7H14 | 1,2-trans-Dimethyl cyclopentane | -34.6 | -35.3 |
| C7H14 | 1,3-cis-Dimethyl cyclopentane | -35.3 | -34.6 |
| C7H14 | 1-Heptene | -13.9 | -13.9 |
| C7H14 | Ethyl cyclopentane | -36.7 | -36.7 |
| C7H14 | Methyl-cyclohexane | -36.3 | -36.3 |
| C7H16 | 2,2,3-Trimethyl butane | -22.2 | -22.2 |
| C7H16 | 2,2-Dimethyl pentane | -30.4 | -30.4 |
| C7H16 | 2,3-Dimethyl pentane | -32 | -32.0 |
| C7H16 | 2,4-Dimethyl pentane | -34.6 | -34.6 |
| C7H16 | 2-Methyl hexane | -39.3 | -39.4 |
| C7H16 | 3,3-Dimethyl pentane | -27 | -27.0 |
| C7H16 | 3-Ethyl pentane | -35.9 | -36.0 |
| C7H16 | 3-Methyl hexane | -37.6 | -37.6 |
| C7H16 | n-Heptane | -43.9 | -43.9 |
| C8H8 | Cubane | 98.9 | 98.9 |
| C8H8 | Cyclooctatetraene | 56.1 | 56.1 |
| C8H8 | Styrene | 37.5 | 37.6 |
| C8H10 | Ethylbenzene | 8.7 | 8.7 |
| C8H10 | m-Xylene | 5.8 | 5.9 |
| C8H10 | o-Xylene | 8.2 | 8.3 |
| C8H10 | p-Xylene | 5.6 | 5.7 |
| C8H12 | 1,5-Cyclooctadiene | 11.3 | 11.3 |
| C8H12 | 4-Vinyl cyclohexene | 13 | 13.1 |
| C8H14 | 1-Octyne | 17 | 17.0 |
| C8H14 | 2,5-Dimethyl 2,4-hexadiene | -2.6 | -2.4 |
| C8H14 | 2-Octyne | 5.3 | 5.3 |
| C8H14 | 3,4-Dimethyl-(E,E)-2,4-hexadiene | -2.4 | -2.4 |
| C8H14 | 3,4-Dimethyl-(E,Z)-2,4-hexadiene | -2.2 | -1.9 |
| C8H14 | 3,4-Dimethyl-(Z,Z)-2,4-hexadiene | -2 | -2.0 |
| C8H14 | 3-Octyne | 4.7 | 4.7 |
| C8H14 | 4-Octyne | 4.6 | 4.6 |
| C8H14 | Bicyclo(2.2.2)-octane | -26.5 | -26.5 |
| C8H16 | 1-Octene | -19.3 | -19.3 |
| C8H16 | Ethylcyclohexane | -39.5 | -39.5 |
| C8H18 | 2,2,3,3-Tetramethyl butane | -12.8 | -12.8 |
| C8H18 | 2,2,3-Trimethyl pentane | -24.6 | -24.6 |
| C8H18 | 2,2,4-Trimethyl pentane | -29.3 | -29.2 |
| C8H18 | 2,2-Dimethyl hexane | -35.1 | -35.1 |
| C8H18 | 2,3,3-Trimethyl pentane | -24.5 | -24.5 |
| C8H18 | 2,3,4-Trimethyl pentane | -28.8 | -28.8 |
| C8H18 | 2,3-Dimethyl hexane | -36.6 | -36.6 |
| C8H18 | 2,4-Dimethyl hexane | -37.5 | -37.5 |
| C8H18 | 2,5-Dimethyl hexane | -39.4 | -39.5 |
| C8H18 | 2-Methyl heptane | -44.1 | -44.1 |
| C8H18 | 3,3-Dimethyl hexane | -31.2 | -31.2 |
| C8H18 | 3,4-Dimethyl hexane | -31.7 | -31.6 |
| C8H18 | 3-Ethyl hexane | -39.8 | -39.8 |
| C8H18 | 3-Ethyl-2-methyl pentane | -31.6 | -31.6 |
| C8H18 | 3-Ethyl-3-methyl pentane | -27.8 | -27.8 |
| C8H18 | 3-Methyl heptane | -42.3 | -42.3 |
| C8H18 | 4-Methyl heptane | -42.1 | -42.1 |
| C8H18 | n-Octane | -48.7 | -48.7 |
| C9H10 | alpha-Methyl styrene | 30.4 | 30.5 |
| C9H10 | Cyclopropyl benzene | 37.5 | 37.6 |
| C9H18 | 1,3,5-Trimethyl cyclohexane | -39 | -39.0 |
| C9H18 | cis-cis-trans-1,3,5-Trimethyl cyclohexane | -37.8 | -37.8 |
| C9H20 | 3,3-Diethylpentane | -27.9 | -27.9 |
| C9H20 | n-Nonane | -53.4 | -53.4 |
| C10H8 | Azulene | 72 | 72.1 |
| C10H8 | Naphthalene | 38.2 | 38.3 |
| C10H10 | 1,4-Dicyclopropylbuta-1,3-diyne | 117.7 | 117.7 |
| C10H10 | 1-Butynl benzene | 52 | 52.0 |
| C10H10 | 2a,4a,6a,6b-Tetrahydrocyclopentapentalene | 47.6 | 47.6 |
| C10H10 | Bulvalene | 63.4 | 63.4 |
| C10H10 | Diisopropenyldiacetylene | 103 | 103.0 |
| C10H10 | Tricyclo[6.2.0.0]deca-1(8),2,6-triene | 54.1 | 54.1 |
| C10H12 | 1,2,6,7-Cyclodecatetraene | 67.8 | 67.8 |
| C10H12 | Dispiro[2.2.2.2]deca-4,9-diene | 65.1 | 65.1 |
| C10H12 | Tetralin | 1.3 | 1.3 |
| C10H14 | (1-Methylpropyl) benzene | 4.7 | 4.5 |
| C10H14 | (2-Methylpropyl) benzene | 3.6 | 3.7 |
| C10H14 | 1,2,3,4-Tetramethyl benzene | 0.9 | 1.0 |
| C10H14 | 1,2,3,4-Tetramethylfulvene | 20 | 20.1 |
| C10H14 | 1,2,3,5-Tetramethyl benzene | -3.1 | -3.0 |
| C10H14 | 1,2,4,5-Tetramethyl benzene | -4.7 | -4.6 |
| C10H14 | tert-Butyl benzene | 15.4 | 15.6 |
| C10H14 | Tetrahydrotriquinacene | -10.8 | -10.8 |
| C10H16 | 1,2,3,4,5-Pentamethyl-1,3-cyclopentadiene | -6.5 | -6.5 |
| C10H16 | Adamantane | -26.5 | -26.5 |
| C10H16 | Camphene | 14.6 | 14.6 |
| C10H16 | Perhydrotriquinacene | -38.9 | -38.9 |
| C10H18 | 1-Methyl-4-(1-methylethyl)-cyclohexene | -20.4 | -20.5 |
| C10H18 | 4-Methyl-1-(1-methylethyl)-cyclohexene | -23.8 | -23.8 |
| C10H18 | cis-Decalin | -37.2 | -37.3 |
| C10H18 | Spiro(4-5)decane | -37.3 | -37.3 |
| C10H18 | trans-Decalin | -41.9 | -41.9 |
| C10H20 | (E)-2,2,5,5-Tetramethyl-3-hexene | -10.9 | -10.9 |
| C10H20 | (Z)-2,2,5,5-Tetramethyl-3-hexene | -0.3 | -0.3 |
| C10H20 | 1-Decene | -28 | -28.0 |
| C10H20 | Butyl cyclohexane | -48.8 | -48.8 |
| C10H20 | Pentyl cyclopentane | -50.7 | -50.7 |
| C10H22 | 2,2,5,5-Tetramethylhexane | -30.7 | -11.8 |
| C10H22 | 3,3,4,4-Tetramethylhexane | -11.8 | -30.7 |
| C10H22 | n-Decane | -58.1 | -58.1 |
| C11H16 | Pentamethylbenzene | -2.6 | -2.5 |
| C11H22 | 1,1,4-Trimethylcycloheptane | -26.5 | -26.5 |
| C11H22 | Hexyl cyclopentane | -55.5 | -55.5 |
| C11H22 | Pentyl cyclohexane | -53.4 | -53.5 |
| C11H24 | Undecane | -62.8 | -62.8 |
| C12H8 | Acenaphthylene | 67 | 67.1 |
| C12H8 | Biphenylene | 94.6 | 94.7 |
| C12H10 | Acenaphthene | 33 | 33.1 |
| C12H10 | Biphenyl | 45.9 | 46.0 |
| C12H18 | Hexamethylbenzene | 0.1 | 0.1 |
| C12H24 | Hexylcyclohexane | -58.2 | -58.2 |
| C12H26 | n-Duodecane | -67.6 | -67.6 |
| C13H10 | Fluorene | 44.8 | 44.9 |
| C13H28 | Tri-t-butylmethane | 32.4 | 32.4 |
| C13H28 | Tridecane | -72.3 | -72.3 |
| C14H10 | Anthracene | 58.7 | 58.8 |
| C14H10 | Diphenylethyne | 89.4 | 89.5 |
| C14H10 | Phenanthrene | 55.5 | 55.6 |
| C14H12 | 9,10-Dihydro-phenanthrene | 38.3 | 38.3 |
| C14H12 | 9-Methyl-9H-fluorene | 42.2 | 42.3 |
| C14H12 | Octalene | 106.1 | 106.2 |
| C14H12 | Stilbene | 63.3 | 63.4 |
| C14H14 | 1,2,3,4-Tetrahydrophenanthrene | 21.8 | 21.9 |
| C14H14 | 4,4'-Dimethylbiphenyl | 30.3 | 30.4 |
| C14H14 | Bibenzyl | 37.5 | 37.6 |
| C14H16 | 1,4,5,8-Tetramethynaphthalene | 31.3 | 31.4 |
| C14H18 | 1,2,3,4,5,6,7,8-Octahydro-anthracene | -18.6 | -18.6 |
| C14H20 | Diadamantane | -23.6 | -23.6 |
| C14H24 | 1,3,5,7-Tetramethyladamantane | -22.8 | -22.8 |
| C14H28 | (E)-3,4-Di-tert-butyl-3-hexene | 19.2 | 19.2 |
| C14H28 | Cyclotetradecane | -53.2 | -53.2 |
| C14H28 | n-Nonylcyclopentane | -69.6 | -69.6 |
| C14H28 | Octylcyclohexane | -67.7 | -67.7 |
| C14H30 | 3,3,4,4-Tetraethylhexane | 6.2 | 6.2 |
| C14H30 | Octamethylhexane | 46.7 | 46.7 |
| C14H30 | Tetradecane | -77 | -77.0 |
| C15H12 | 4-Methylphenanthrene | 56.8 | 56.9 |
| C15H22 | 1-Methyldiadamantane | -18.1 | -18.0 |
| C15H22 | 3-Methyladamantane | -22.7 | -22.7 |
| C15H22 | 4-Methyldiadamantane | -23 | -23.0 |
| C15H22 | 6-(1,1-dimethylethyl)-2,3-dihydro-1,1-dimethyl-1H-Indene | 0.5 | 0.6 |
| C15H30 | n-Nonylcyclohexane | -72.3 | -72.3 |
| C15H32 | Pentadecane | -81.7 | -81.7 |
| C16H10 | Fluoranthene | 72.6 | 72.7 |
| C16H10 | Pyrene | 60.5 | 60.7 |
| C16H14 | 2,7-Dimethylphenanthrene | 39.9 | 40.0 |
| C16H14 | 4,5,9,10-Tetrahydropyrene | 25.4 | 25.5 |
| C16H14 | 9,10-Dimethylphenanthrene | 52.7 | 52.9 |
| C16H16 | (2.2)Metaparacyclophane | 62.6 | 62.7 |
| C16H16 | [2.2]Metacyclophane | 58.6 | 58.7 |
| C16H16 | [2.2]Paracyclophane | 66.5 | 66.6 |
| C16H18 | 1,2,3,6,7,8-Hexahydropyrene | 9.2 | 9.3 |
| C16H28 | Tricyclo[8.2.2.2]-hexadecane | -15.8 | -15.8 |
| C16H32 | 1-Hexadecene | -56.4 | -56.4 |
| C16H32 | Decylcyclohexane | -77.1 | -77.1 |
| C16H32 | n-Undecylcyclopentane | -79 | -79.1 |
| C17H34 | n-Dodecylcyclopentane | -83.8 | -83.8 |
| C17H34 | Undecylcyclohexane | -81.8 | -81.8 |
| C17H36 | Heptadecane | -91.2 | -91.2 |
| C18H14 | p-Terphenyl | 70.6 | 70.7 |
| C18H18 | 2,5-Diphenyl-1,5-hexadiene | 72.5 | 72.6 |
| C18H18 | 3,4,5,6-Tetramethylphenanthrene | 48.9 | 49.0 |
| C18H20 | [3.3]Paracyclophane | 36.9 | 37.0 |
| C18H22 | 1,1'-(1,1,2,2-Tetramethyl-1,2-ethanediyl)bis-benzene | 70.2 | 70.3 |
| C18H36 | Dodecylcyclohexane | -86.5 | -86.5 |
| C18H36 | n-Tridecylcyclopentane | -88.5 | -88.5 |
| C18H38 | 1,1,2,2-Tetra-t-butylethane | 70.2 | 70.2 |
| C18H38 | Octadecane | -95.9 | -95.9 |
| C19H20 | 2,6-Diphenyl-1,6-heptadiene | 67.8 | 67.9 |
| C19H38 | n-Tetradecylcyclopentane | -93.2 | -93.2 |
| C19H38 | n-Tridecylcyclohexane | -91.2 | -91.3 |
| C19H40 | Nonadecane | -100.6 | -100.6 |
| C20H14 | 9,10-Dihydro-9,10[1',2']benzanthracene | 87.1 | 87.3 |
| C20H16 | 3,9-Dimethylbenz[a]anthracene | 59.1 | 59.3 |
| C20H16 | 5,6-Dimethyl chrysene | 76.2 | 76.4 |
| C20H16 | 9,10-Dimethyl-1,2-benzanthracene | 79.6 | 79.8 |
| C20H30 | 1,3,5-Tri-tert-butyl pentalene | 54.6 | 54.7 |
| C20H36 | Tetra-tert-butyltetrahedrane | 80.9 | 80.9 |
| C20H38 | Meso-3,4-dicyclohexyl-2,5-dimethylhexane | -36 | -36.0 |
| C20H40 | Tetradecylcyclohexane | -96 | -96.0 |
| C20H42 | Eicosane | -105.3 | -105.4 |
| N | Nitrogen, cation | 417.5 | 417.5 |
| N | Nitrogen, atom | 113 | 113.0 |
| NH2 | Amidogen | 36.4 | 36.4 |
| NH3 | Ammonia | -6.4 | -6.4 |
| NH4 | Ammonium, cation | 164.6 | 164.6 |
| CN | Cyanide | 126.3 | 126.3 |
| CNH | Hydrogen cyanide | 35.3 | 35.3 |
| CNH4 | CH2-NH2, cation | 186.8 | 186.8 |
| CNH4 | CH3-NH. | 29.3 | 29.3 |
| CNH4 | CH3NH, anion | 23.5 | 23.5 |
| CNH5 | Methylamine | -7.6 | -7.6 |
| C2NH3 | Acetonitrile | 19.2 | 19.2 |
| C2NH3 | Methyl isocyanide | 60.3 | 60.3 |
| C2NH5 | Ethyleneimine (Azirane) | 25 | 25.0 |
| C2NH6 | Dimethyl nitrogen, anion | 8.5 | 8.5 |
| C2NH7 | Dimethylamine | -6.7 | -6.7 |
| C2NH7 | Ethylamine | -13.3 | -13.3 |
| C3NH3 | Acrylonitrile | 43.8 | 43.8 |
| C3NH5 | Ethyl cyanide | 13.7 | 13.7 |
| C3NH7 | Cyclopropylamine | 15.3 | 15.3 |
| C3NH9 | Isopropylamine | -16.4 | -16.4 |
| C3NH9 | n-Propylamine | -18.3 | -18.3 |
| C3NH9 | Trimethylamine | -2.8 | -2.9 |
| C4NH5 | (E)-2-Butenenitrile | 33.8 | 33.8 |
| C4NH5 | (Z)-2-Butenenitrile | 34.1 | 34.1 |
| C4NH5 | 3-Butenenitrile | 39.2 | 39.2 |
| C4NH5 | Pyrrole | 32.4 | 32.4 |
| C4NH7 | Butanenitrile | 8.9 | 8.9 |
| C4NH7 | Isobutane nitrile | 11.2 | 11.2 |
| C4NH9 | Pyrrolidine | -15.9 | -15.9 |
| C4NH11 | 2-Butylamine | -20.4 | -20.4 |
| C4NH11 | 2-Methyl-1-propylamine | -19.3 | -19.3 |
| C4NH11 | N-Butylamine | -23.1 | -23.1 |
| C4NH11 | t-Butylamine | -15.5 | -15.5 |
| C5NH5 | Pyridine | 28.7 | 28.8 |
| C5NH7 | N-Methyl pyrrole | 32.2 | 32.2 |
| C5NH9 | 1,2,3,6-Tetrahydropyridine | 6.1 | 6.1 |
| C5NH9 | 2-Cyanobutane | 7.7 | 7.8 |
| C5NH9 | Butyl cyanide | 4.1 | 4.1 |
| C5NH9 | t-Butylnitrile | 12.5 | 12.5 |
| C5NH11 | Cyclopentylamine | -22.6 | -22.6 |
| C5NH11 | Piperidine | -18.6 | -18.6 |
| C5NH13 | N-Methyl-n-butylamine | -21.7 | -21.8 |
| C6NH7 | 1-Cyclopentenecarbonitrile | 28.5 | 28.6 |
| C6NH7 | 2-Cyclopentenecarbonitrile | 34.9 | 34.9 |
| C6NH7 | 2-Methyl pyridine | 19.7 | 19.7 |
| C6NH7 | 3-Methyl pyridine | 20.4 | 20.4 |
| C6NH7 | 4-Methyl pyridine | 20.8 | 20.8 |
| C6NH7 | Aniline | 21.6 | 21.7 |
| C6NH9 | 2,5-Dimethyl-1H-pyrrole | 10.1 | 10.1 |
| C6NH9 | Cyclopentanecarbonitrile | 5.4 | 5.4 |
| C6NH13 | 2-Methylpiperidine | -21.6 | -21.6 |
| C6NH13 | Cyclohexamethylenimine | -17.7 | -17.7 |
| C6NH13 | Cyclohexanamine | -25.8 | -25.8 |
| C6NH15 | Di-n-propylamine | -27.3 | -27.3 |
| C6NH15 | Diisopropylamine | -20.3 | -20.3 |
| C6NH15 | Triethylamine | -14.8 | -14.8 |
| C7NH5 | Phenyl cyanide | 51.9 | 51.9 |
| C7NH9 | 1-Cyclohexenecarbonitrile | 21 | 21.0 |
| C7NH9 | 2,6-Dimethylpyridine | 10.6 | 10.7 |
| C7NH9 | 2-Cyclohexenecarbonitrile | 26.5 | 26.6 |
| C7NH9 | Benzylamine | 19.4 | 19.5 |
| C7NH9 | m-Toluidine | 14.1 | 14.1 |
| C7NH9 | N-Methylaniline | 24.2 | 24.2 |
| C7NH9 | o-Toluidine | 16.1 | 16.1 |
| C7NH9 | p-Toluidine | 13.7 | 13.8 |
| C7NH11 | Cyclohexanecarbonitrile | 1.7 | 1.7 |
| C7NH13 | Hexahydro-1H-pyrrolizine | -20.1 | -20.1 |
| C7NH13 | n-Heptanenitrile | -5.3 | -5.3 |
| C7NH17 | Isopropylbutylamine | -28.9 | -29.0 |
| C8NH11 | 1-Norbornylcyanide | 24.2 | 24.3 |
| C8NH11 | 1-Norbornylisocyanide | 63.1 | 63.0 |
| C8NH11 | 5-Ethyl-2-methyl-pyridine | 6.4 | 6.4 |
| C8NH11 | N,N-Dimethyl aniline | 33.4 | 28.8 |
| C8NH11 | N-Ethyl aniline | 19.2 | 19.3 |
| C8NH15 | 3-Azabicyclo[3.2.2]nonane | -13.9 | -13.9 |
| C8NH15 | n-Heptyl cyanide | -10 | -10.0 |
| C8NH17 | N-(2-Methylpropylidene)-butylamine | -15.2 | -15.2 |
| C8NH19 | 2-Methyl-N-(2-methylpropyl)-1-propanamine | -28.5 | -28.5 |
| C8NH19 | Di-sec-butylamine | -26.6 | -26.7 |
| C8NH19 | Dibutylamine | -36.6 | -36.7 |
| C8NH19 | N-(2-Methylpropyl)-1-butanamine | -32.9 | -32.9 |
| C8NH19 | n-Octylamine | -42 | -42.0 |
| C9NH7 | Isoquinoline | 45.2 | 45.2 |
| C9NH7 | Quinoline | 44.6 | 44.7 |
| C9NH9 | 2,6-Dimethylbenzonitrile | 39.9 | 40.0 |
| C9NH11 | (1a,2a,4a)-Bicyclo[2.2.2]oct-5-ene-2-carbonitrile | 39.7 | 39.7 |
| C9NH11 | (1a,2b,4a)-Bicyclo[2.2.2]oct-5-ene-2-carbonitrile | 40.1 | 40.1 |
| C9NH11 | 1,2,3,4-Tetrahydroquinoline | 14 | 14.1 |
| C9NH11 | 5,6,7,8-Tetrahydroquinoline | 6.9 | 7.0 |
| C9NH13 | N,N-Dimethyl m-toluidine | 26.1 | 26.2 |
| C9NH13 | N,N-Dimethyl p-toluidine | 25.3 | 25.4 |
| C9NH13 | N-Ethyl m-toluidine | 11.7 | 11.7 |
| C9NH17 | cis-3,7a-H-cis-5,8-H-3,5-Dimethylpyrrolizidine | -25.9 | -25.9 |
| C9NH17 | Decahydro trans-quinoline | -26.9 | -26.9 |
| C9NH19 | 2,2,6,6-Tetramethyl piperidine | -14 | -14.0 |
| N2 | Nitrogen | 8.3 | 8.3 |
| N2H2 | Diazene | 31.8 | 31.8 |
| N2H4 | Hydrazine | 14.1 | 14.1 |
| CN2H2 | Diazomethane | 67.2 | 67.2 |
| CN2H2 | N=N-CH2- | 72.4 | 72.4 |
| CN2H6 | Methylhydrazine | 14.3 | 14.3 |
| C2N2 | Cyanogen | 66.6 | 66.6 |
| C2N2H8 | 1,1-Dimethylhydrazine | 18.1 | 18.0 |
| C2N2H8 | 1,2-Dimethylhydrazine | 15 | 14.9 |
| C3N2H4 | 1H-Pyrazole | 45.3 | 45.3 |
| C3N2H4 | Imidazole | 33.2 | 33.2 |
| C3N2H10 | 1,2-Propanediamine | -10.6 | -10.7 |
| C4N2 | Dicyanoacetylene | 111.3 | 111.4 |
| C4N2H2 | Fumaronitrile | 74.7 | 74.7 |
| C4N2H4 | 1,3-Diazine | 34.9 | 34.9 |
| C4N2H4 | Pyrazine | 37.7 | 37.7 |
| C4N2H4 | Pyridazine | 43.5 | 43.5 |
| C4N2H4 | Succinonitrile | 48.8 | 48.8 |
| C4N2H6 | 2-Methyl-1H-imidazole | 21.5 | 21.5 |
| C4N2H8 | (Dimethylamino) acetonitrile | 31.5 | 31.5 |
| C4N2H8 | 1,4,5,6-Tetrahydropyrimidine | 8.9 | 8.9 |
| C4N2H10 | Piperazine | -2.9 | -2.9 |
| C5N2H6 | 2-Aminopyridine | 25.7 | 25.8 |
| C5N2H6 | 3-Aminopyridine | 29.2 | 29.3 |
| C5N2H6 | 4-Aminopyridine | 28.3 | 28.3 |
| C5N2H6 | Dimethyl propanedinitrile | 52.8 | 52.8 |
| C5N2H8 | 2-Ethyl-1H-imidazole | 16.5 | 16.5 |
| C5N2H10 | Diethylcyanamide | 29.3 | 29.4 |
| C5N2H12 | Butylmethyldiazene | 5.7 | 5.6 |
| C5N2H14 | N,N-Dimethyl-1,3-propanediamine | -8.8 | -8.8 |
| C6N2H4 | 2-Cyanopyridine | 60.2 | 60.3 |
| C6N2H4 | 3-Cyanopyridine | 59.6 | 59.7 |
| C6N2H4 | 4-Cyanopyridine | 60.8 | 60.9 |
| C6N2H8 | 2,3-Dimethyl pyrazine | 20.2 | 20.2 |
| C6N2H8 | Hexanedinitrile | 38.5 | 38.5 |
| C6N2H8 | Phenylhydrazine | 45.2 | 45.2 |
| C6N2H12 | 3(Dimethylamino) propanenitrile | 22.1 | 22.1 |
| C6N2H12 | Tetramethyldiazetine | 33.6 | 33.6 |
| C6N2H12 | Triethylenediamine | 19.3 | 19.3 |
| C6N2H14 | 1,2-Diisopropyldiazene | 3.7 | 3.7 |
| C6N2H14 | Dipropyldiazene | -0.9 | -0.9 |
| C7N2H6 | 1H-Benzimidazole | 45.7 | 45.8 |
| C7N2H6 | 1H-Indazole | 58 | 58.0 |
| C7N2H10 | 1-Methyl-1-phenylhydrazine | 24.9 | 24.9 |
| C7N2H10 | t-Butylmalononitrile | 52.5 | 52.5 |
| C7N2H10 | Trimethyl pyrazine | 10.5 | 10.6 |
| C7N2H12 | 1-Piperidineacetonitrile | 19.6 | 19.6 |
| C7N2H14 | 3,3,5,5-Tetramethyl-1-pyrazoline | 5.2 | 5.2 |
| C8N2H4 | m-Dicyanobenzene | 83.8 | 83.8 |
| C8N2H4 | o-Dicyanobenzene | 85.3 | 85.3 |
| C8N2H4 | p-Dicyanobenzene | 83.9 | 83.9 |
| C8N2H6 | Phthalazine | 59 | 59.0 |
| C8N2H6 | Quinazoline | 50.2 | 50.3 |
| C8N2H6 | Quinoxaline | 52.6 | 52.7 |
| C8N2H12 | n-Pentylmalonodinitrile | 34.2 | 34.2 |
| C8N2H12 | Tetramethylbutanedinitrile | 56.8 | 56.8 |
| C8N2H12 | Tetramethylpyrazine | 2.6 | 2.7 |
| C8N2H14 | 1,4-Dimethyl-2,3-diaza-bicyclo[2.2.2]oct-2-ene | 13.7 | 13.7 |
| C8N2H16 | 3,4,5,6-Tetrahydro-3,3,6,6-tetramethylpyridazine | 6.7 | 6.7 |
| C8N2H18 | Di-n-butyldiazene | -10.4 | -10.4 |
| C8N2H18 | Di-tert-butyldiazene | 4.9 | 4.9 |
| C8N2H20 | 1,2-Dibutylhydrazine | -14.4 | -14.4 |
| N3 | Azide radical | 97.1 | 97.1 |
| N3H | Hydrazoic acid | 73 | 73.1 |
| C3N3H3 | 1,3,5-Triazine | 39.9 | 40.0 |
| C5N3H | Ethylenetricarbonitrile | 110.4 | 110.4 |
| C5N3H3 | 1,1,1-Ethanetricarbonitrile | 96.1 | 96.2 |
| CN4H2 | 1-H Tetrazole | 53.8 | 53.7 |
| CN4H2 | 2-H-Tetrazole | 58 | 58.0 |
| C6N4 | Tetracyanoethylene | 147.9 | 148.0 |
| C6N4H12 | 1,3,5,7-Tetraazaadamantane | 51.4 | 51.5 |
| C3N6H6 | Melamine | 21.4 | 21.7 |
| C10NH9 | 2-Methyl-quinoline | 35.7 | 35.8 |
| C10NH9 | 4-Methyl-quinoline | 38.9 | 39.0 |
| C10NH9 | 6-Methyl-quinoline | 36.9 | 37.0 |
| C10NH9 | 8-Methyl-quinoline | 38.1 | 38.1 |
| C10NH11 | 2,4,6-Trimethyl-benzonitrile | 32.4 | 32.5 |
| C10NH11 | 2,4,6-Trimethylphenyl isocyanide | 70.8 | 70.9 |
| C10NH15 | N,N-Diethyl aniline | 26.9 | 26.9 |
| C10NH19 | n-Nonyl cyanide | -19.5 | -19.5 |
| C11NH11 | 2,6-Dimethyl quinoline | 28 | 28.1 |
| C11NH11 | 2,7-Dimethyl quinoline | 28 | 28.1 |
| C11NH15 | 1-Adamantyl cyanide | 11.4 | 11.4 |
| C11NH15 | 1-Adamantyl isocyanide | 50 | 50.0 |
| C11NH17 | 2-Methyl-6-t-butylaniline | 19.7 | 19.7 |
| C11NH21 | n-Undecanenitrile | -24.2 | -24.2 |
| C12NH9 | Carbazole | 53.4 | 53.5 |
| C12NH11 | 2-Biphenylamine | 47.8 | 47.9 |
| C12NH11 | Biphenylamine | 56 | 55.7 |
| C12NH23 | 2-n-Butyl-2-methylhexanenitrile | -7.3 | -7.3 |
| C13NH9 | 6,7-Benzoquinoline | 60.5 | 60.6 |
| C13NH9 | a-Benzoquinoline | 59.8 | 59.9 |
| C13NH9 | Acridine | 64.2 | 64.3 |
| C13NH9 | Benzo[f]quinoline | 61.4 | 61.5 |
| C13NH9 | Phenanthridine | 60.5 | 60.6 |
| C13NH11 | N-Methylcarbazole | 56 | 56.1 |
| C13NH15 | 1,2,3,4-Tetrahydro-N-methylcarbazole | 22.8 | 22.9 |
| C14NH13 | 9-Ethyl-9H-carbazole | 51 | 51.1 |
| C14NH27 | Tetradecanenitrile | -38.4 | -38.4 |
| C9N2H8 | 3-Quinolinamine | 45.1 | 45.2 |
| C9N2H8 | 5-Quinolinamine | 47.2 | 47.2 |
| C9N2H8 | 6-Quinolinamine | 45.2 | 45.2 |
| C9N2H8 | 8-Quinolinamine | 45.2 | 45.3 |
| C9N2H18 | 2-(Diethylamino)-pentanenitrile | 14 | 14.0 |
| C10N2H8 | 2,2-Bipyridyl | 58.7 | 58.8 |
| C10N2H8 | 2,4-Bipyridyl | 59.9 | 60.0 |
| C10N2H8 | 4,4'-Bipyridine | 61.1 | 61.1 |
| C10N2H10 | 2,3-Dimethyl quinoxaline | 36.3 | 36.4 |
| C10N2H12 | alpha N,N-dimethylamino phenylacetonitrile | 58.8 | 58.9 |
| C10N2H16 | Ethyl(1,1-dimethylpropyl)malonodinitrile | 56.5 | 56.6 |
| C10N2H16 | Meso-2,3-diethyl-2,3-dimethylsuccinodinitrile | 55 | 55.0 |
| C10N2H16 | Methyl(1,1,2-trimethylpropyl)malonodinitrile | 66.2 | 66.3 |
| C12N2H8 | Phenazine | 71.6 | 71.7 |
| C12N2H8 | Phenazone | 72.5 | 72.6 |
| C12N2H10 | cis-Azobenzene | 83.4 | 83.5 |
| C12N2H12 | 4,4'-Dimethyl-2,2'-bipyridine | 42.9 | 43.0 |
| C12N2H20 | 1,(1-Piperidinyl) cyclohexanecarbonitrile | 17.6 | 17.6 |
| C13N2H16 | a-Phenyl-1-piperidineacetonitrile | 53.5 | 53.5 |
| C9N3H3 | 1,3,5-Tricyanobenzene | 116.8 | 116.8 |
| C11N3H7 | 1,1,1-Tricyano-2-phenyl ethane | 126.7 | 126.7 |
| C16NH35 | Dioctylamine | -74.5 | -74.5 |
| C18NH15 | Triphenylamine | 93.7 | 93.9 |
| C18N2H12 | 2,2'-Biquinoline | 90.9 | 91.1 |
| C15N3H11 | 2,2',6',2'-Terpyridine | 88.5 | 88.6 |
| O | Oxygen, atom | 59.6 | 59.6 |
| HO | Hydroxyl radical | 0.2 | 0.2 |
| HO | Hydroxide, anion | -5.8 | -5.8 |
| H2O | Water | -60.9 | -61.0 |
| H3O | Hydronium, cation | 134.1 | 134.2 |
| CO | Carbon monoxide | -5.9 | -6.0 |
| CHO | HCO, cation | 184.9 | 184.8 |
| CHO | HCO | -1.4 | -1.4 |
| CH2O | Formaldehyde | -32.9 | -32.9 |
| CH3O | CH2OH, cation | 155.5 | 155.5 |
| CH3O | Methoxy, anion | -39.8 | -39.8 |
| CH4O | Methanol | -57.4 | -57.4 |
| C2H2O | Ketene | -6.8 | -6.9 |
| C2H4O | Acetaldehyde | -42.3 | -42.3 |
| C2H4O | Ethylene oxide | -15.6 | -15.5 |
| C2H5O | Ethoxy, anion | -45.3 | -45.3 |
| C2H6O | Dimethyl ether | -51.3 | -51.2 |
| C2H6O | Ethanol | -63 | -63.0 |
| C3H6O | Acetone | -49.4 | -49.5 |
| C3H6O | Propanal | -47.4 | -47.4 |
| C3H6O | Trimethylene oxide | -37.2 | -37.2 |
| C3H8O | Isopropanol | -65.1 | -65.2 |
| C3H8O | Methyl ethyl ether | -56.7 | -56.7 |
| C3H8O | Propanol | -67.6 | -67.6 |
| C4H4O | Acetyl acetylene | 12.3 | 12.3 |
| C4H4O | Furan | -8.7 | -8.7 |
| C4H6O | 2,3-Dihydrofuran | -29.9 | -29.9 |
| C4H6O | Crotonaldehyde | -27.9 | -27.9 |
| C4H6O | Divinyl ether | -2.1 | -2.0 |
| C4H8O | Butanal | -52.2 | -52.9 |
| C4H8O | Isobutanal | -50.6 | -50.6 |
| C4H8O | Methyl ethyl ketone | -54.1 | -54.1 |
| C4H8O | Tetrahydrofuran | -59.3 | -59.3 |
| C4H10O | Diethyl ether | -62.1 | -62.0 |
| C4H10O | t-Butanol | -64.3 | -64.4 |
| C5H8O | 2,3-Dihydro-5-methyl-furan | -39.9 | -39.9 |
| C5H8O | 2-Ethylacrolein | -28 | -30.7 |
| C5H8O | 3,4-Dihydro-2H-pyran | -39.4 | -39.4 |
| C5H8O | 3-Penten-2-one | -34.9 | -34.9 |
| C5H8O | Cyclopentanone | -57.1 | -57.1 |
| C5H10O | Diethyl ketone | -59.5 | -59.5 |
| C5H10O | Tetrahydropyran | -62.1 | -62.1 |
| C5H12O | t-Butyl methyl ether | -54.6 | -54.6 |
| C6H5O | Phenoxy, anion | -42.3 | -42.3 |
| C6H6O | Phenol | -26.8 | -26.7 |
| C6H10O | 4-Methyl-3-penten-2-one | -40.9 | -40.9 |
| C6H10O | Cyclohexanone | -60.2 | -60.2 |
| C6H12O | Methyl neopentyl ketone | -50.9 | -50.9 |
| C6H14O | Di-isopropyl ether | -62.5 | -62.5 |
| C7H6O | Benzaldehyde | -9.7 | -9.6 |
| C7H8O | Anisole | -17.8 | -17.7 |
| C7H8O | m-Cresol | -34.3 | -34.2 |
| C7H8O | o-Cresol | -33.3 | -33.3 |
| C7H8O | p-Cresol | -34.7 | -34.6 |
| C7H10O | 2-Methyl-5-hexen-3-yn-2-ol | 5.2 | 5.3 |
| C7H10O | 2-Norbornanone | -37.4 | -37.4 |
| C7H10O | cis-2,3-Epoxybicyclo[2.2.1]heptane | 2.8 | 2.8 |
| C7H10O | Norbornan-7-one | -37.7 | -37.7 |
| C7H12O | 1-Methoxy cyclohexene | -49.1 | -49.1 |
| C7H12O | Bicyclo[2.2.1]heptan-7-ol | -51.2 | -51.2 |
| C7H12O | cis-1,2-Epoxycycloheptane | -35.7 | -35.6 |
| C7H12O | Cycloheptanone | -59.5 | -59.6 |
| C7H14O | 2,4-Dimethyl 3-pentanone | -63.1 | -63.1 |
| C7H14O | 2-Methyl cis-cyclohexanol | -73.8 | -73.8 |
| C7H14O | 3,3-Dimethyl-2-pentanone | -52.3 | -52.3 |
| C7H14O | 4-Heptanone | -67.8 | -67.8 |
| C7H14O | Heptanal | -66.4 | -67.1 |
| C7H14O | t-Butyl ethyl ketone | -55.4 | -55.4 |
| C7H16O | n-Heptanol | -86.7 | -86.7 |
| C7H16O | t-Butyl isopropyl ether | -60.5 | -60.5 |
| C8H6O | Benzofuran | 3.8 | 3.8 |
| C8H8O | 1,3-Dihydro isobenzofuran | -20.5 | -20.4 |
| C8H8O | 1-Phenylethenol | -9.5 | -9.5 |
| C8H8O | 2,3-Dihydro-benzofuran | -22.1 | -22.1 |
| C8H8O | Acetophenone | -17.1 | -17.1 |
| C8H10O | 2,3-Dimethyl phenol | -36.1 | -36.0 |
| C8H10O | 2,4-Dimethyl phenol | -40.3 | -40.2 |
| C8H10O | 2,5-Dimethyl phenol | -40.1 | -40.1 |
| C8H10O | 2,6-Dimethyl phenol | -38.6 | -38.5 |
| C8H10O | 2-Ethyl phenol | -35.3 | -35.2 |
| C8H10O | 3,4-Dimethyl phenol | -39.6 | -39.5 |
| C8H10O | 3,5-Dimethyl phenol | -41.7 | -41.6 |
| C8H10O | 3-Ethyl phenol | -39.1 | -39.1 |
| C8H10O | 4-Ethyl phenol | -39.5 | -39.4 |
| C8H10O | 2-phenylethanol | -34.1 | -34.0 |
| C8H10O | Ethoxybenzene | -23 | -23.0 |
| C8H10O | Phenetole | -21.9 | -22.6 |
| C8H12O | 1-Methylnorcamphor | -40.8 | -40.9 |
| C8H12O | Bicyclo[2.2.2]octanone | -53.4 | -53.4 |
| C8H12O | Bicyclo[3.2.1]octan-2-one | -52.1 | -52.1 |
| C8H12O | Bicyclo[3.2.1]octan-3-one | -52 | -52.1 |
| C8H12O | Bicyclo[3.2.1]octan-8-one | -52.6 | -52.6 |
| C8H12O | cis-Bicyclo[3.3.0]-octan-2-one | -64 | -64.0 |
| C8H12O | trans-Bicyclo[3.3.0]-octan-2-one | -48.1 | -48.2 |
| C8H14O | 3-Oxabicyclo[3,2,2]nonane | -58.1 | -58.0 |
| C8H14O | 6-Methyl-5-hepten-2-one | -48.3 | -48.3 |
| C8H14O | 8-Oxatricyclo[3,2,1,0(1,5)]octane | 50.3 | 50.3 |
| C8H14O | Bicyclo(2.2.2)octan-2-ol | -66 | -66.0 |
| C8H14O | cis-1,2-Epoxycyclooctane | -34.3 | -34.3 |
| C8H14O | Cyclooctanone | -61.5 | -61.5 |
| C8H16O | 2,2,4-Trimethyl-3-pentanone | -56.6 | -56.6 |
| C8H16O | 2-Octanone | -72.9 | -72.9 |
| C8H16O | 3,3,4-Trimethyl pentan-2-one | -48.5 | -48.5 |
| C8H16O | 3-Octanone | -72.9 | -74.0 |
| C8H16O | 4-Octanone | -72.5 | -74.0 |
| C8H16O | Octanal | -71.1 | -71.1 |
| C8H18O | 1-Octanol | -91.4 | -91.4 |
| C8H18O | 1-Tert-butoxybutane | -69.2 | -69.2 |
| C8H18O | 2-(1,1-Dimethylethoxy)-butane | -63.2 | -63.2 |
| C8H18O | Di-n-butyl ether | -81 | -81.0 |
| C8H18O | Di-sec-butyl ether | -69 | -69.1 |
| C8H18O | tert-Butyl ether | -51.8 | -51.8 |
| C8H18O | tert-Butyl isobutyl ether | -65.3 | -65.3 |
| C9H10O | 3,4-Dihydro-1H-2-benzopyran | -26.8 | -26.7 |
| C9H10O | 3,4-Dihydro-2H-1-benzopyran | -28.8 | -28.8 |
| C9H10O | Benzyl methyl ketone | -20.8 | -20.8 |
| C9H12O | 2(-1-Methylethyl)-phenol | -37.2 | -37.2 |
| C9H12O | 2,4,6-Trimethyl phenol | -46.3 | -46.2 |
| C9H12O | 3(-1-Methylethyl)-phenol | -39.6 | -39.6 |
| C9H12O | 4-(-1-Methylethyl)-phenol | -40 | -40.0 |
| C9H14O | 2,6,6-Trimethyl-2-cyclohexen-1-one | -39.9 | -39.9 |
| C9H14O | Bicycle[3.3.1]nonan-9-one -check -3-one | -58.4 | -58.4 |
| C9H14O | cis Octahydro-2H-inden-2-one | -64.2 | -64.3 |
| C9H14O | trans Octahydro-2H-inden-2-one | -62.1 | -62.1 |
| C9H16O | Cyclononanone | -61.7 | -61.7 |
| C9H18O | 2,6-Dimethyl-4-heptanone | -69.7 | -69.7 |
| C9H18O | 2-Nonanone | -78.3 | -78.3 |
| C9H18O | 3,3,4,4-Tetramethyl-2-pentanone | -37.6 | -37.6 |
| C9H18O | 5-Nonanone | -77.3 | -77.3 |
| C9H18O | Di-tert-butyl ketone | -43.6 | -43.6 |
| C9H20O | 1-Nonanol | -96.1 | -96.1 |
| C9H20O | Amyl-t-butyl ether | -73.9 | -73.9 |
| C9H20O | Butyl 1,1-dimethylpropyl ether | -71.5 | -71.5 |
| NO | Nitric oxide, cation | 230.6 | 230.6 |
| NO | Nitric oxide | -0.5 | -0.5 |
| CNO | NCO | 31.6 | 31.6 |
| CNHO | Hydrogen isocyanate | -10.8 | -10.8 |
| CNH3O | Formamide | -39.4 | -40.2 |
| C2NH5O | Acetaldoxime | -18.3 | -18.3 |
| C2NH5O | Acetamide | -47.2 | -48.2 |
| C3NH3O | Isoxazole | 19.2 | 19.2 |
| C3NH3O | Oxalone (oxazole) | -8.3 | -8.3 |
| C3NH5O | Acrylamine | -22.4 | -24.4 |
| C3NH5O | Methoxyacetonitrile | -15.7 | -15.6 |
| C3NH7O | Dimethylformamide | -37 | -37.0 |
| C3NH7O | N-Methyl acetamide | -46.9 | -47.0 |
| C3NH7O | Propanamide | -51.7 | -53.5 |
| C3NH9O | Dimethylaminomethanol | -50.2 | -50.2 |
| C4NH5O | 3-Methyl isoxazole | 7.4 | 7.4 |
| C4NH5O | 5-Methyl isoxazole | 8.1 | 8.1 |
| C4NH7O | 2-Pyrrolidinone | -51.8 | -51.9 |
| C4NH7O | 4,5-Dihydro-2-methyl oxazole | -39.7 | -39.6 |
| C4NH7O | Methacrylamide | -30.9 | -32.0 |
| C4NH9O | 2-Methyl propanamide | -54.1 | -55.2 |
| C4NH9O | Butanamide | -56.2 | -58.2 |
| C4NH9O | Isobutylamide | -54.1 | -55.2 |
| C4NH11O | N,N-Diethyl-hydroxylamine | -28.2 | -28.3 |
| C5NH5O | 2-Pyridinol | -25.1 | -25.0 |
| C5NH5O | 3-Pyridinol | -18.7 | -18.7 |
| C5NH5O | 4-Pyridinol | -20 | -20.0 |
| C5NH5O | Pyridine 1 oxide | 44.4 | 44.4 |
| C5NH7O | 3,5-Dimethyl isoxazole | -3.6 | -3.6 |
| C5NH9O | 1-Methyl-2-pyrrolidinone | -51.1 | -51.0 |
| C5NH9O | 2-Ethyl-4,5-dihydro-oxazole | -44.1 | -44.1 |
| C5NH9O | N,N-Dimethylamino-2-propen-3-al | -14.6 | -14.6 |
| C5NH11O | 1-(Dimethylamino)-2-propanone | -37.3 | -37.3 |
| C5NH11O | 2,2-Dimethyl-propanamide | -49 | -50.1 |
| C5NH11O | N,N-Dimethyl propanamide | -45.6 | -45.6 |
| C6NH7O | 2-Hydroxy-6-methylpyridine | -34 | -33.9 |
| C6NH7O | 3-Hydroxy-2-methylpyridine | -26.7 | -26.6 |
| C6NH7O | 3-Hydroxy-6-methylpyridine | -27.8 | -27.8 |
| C6NH7O | 4-Hydroxy-2-methylpyridine | -28.9 | -28.9 |
| C6NH7O | 6-Methyl-2(1H)-pyridinone | -23.1 | -23.0 |
| C6NH7O | m-Amino phenol | -26.5 | -26.4 |
| C6NH7O | o-Amino phenol | -26.2 | -26.2 |
| C6NH7O | p-Amino phenol | -25.9 | -25.9 |
| C6NH9O | Trimethyl isoxazole | -13.3 | -13.3 |
| C6NH11O | Caprolactam | -52.9 | -52.9 |
| C6NH11O | Cyclohexanone oxime | -35.3 | -35.3 |
| C6NH13O | N,N-Diethyl acetamide | -48.9 | -48.9 |
| C6NH13O | N,N-Dimethylbutyramine | -50.1 | -50.1 |
| C7NH5O | Benzoxazole | 5.1 | 5.2 |
| C7NH5O | Isocyanatobenzene | 14 | 14.0 |
| C7NH7O | Benzamide | -15.2 | -16.2 |
| C7NH11O | N,N-Dimethylamino-2,4-pentadiene-5-al | -0.1 | -0.1 |
| C7NH13O | 2-Methoxy-3,3-dimethylbutanenitrile | -18.9 | -18.9 |
| C7NH15O | N,N-Diethylaminoacetone | -43.9 | -44.0 |
| C7NH15O | N,N-Dimethyl-tert-butylcarboxamide | -35 | -41.3 |
| C8NH5O | alpha-oxo Benzeneacetonitrile | 25.4 | 25.4 |
| C8NH9O | 1,3-dimethyl-2-nitroso-benzene | 16.7 | 16.8 |
| C8NH9O | N-methyl-N-phenyl formamide | -6.8 | -6.7 |
| C8NH17O | Octanone-1-oxime | -46.4 | -46.5 |
| C8NH17O | Octanone-2-oxime | -47.5 | -47.5 |
| C8NH17O | Octanone-3-oxime | -49 | -49.0 |
| C8NH17O | Octanone-4-oxime | -48.3 | -48.3 |
| N2O | Nitrous oxide | 31 | 31.0 |
| CN2H4O | Urea | -42.8 | -44.8 |
| C2N2H6O | N-Methyl urea | -41.7 | -43.9 |
| C4N2H6O | Dimethyl furazan | 20.9 | 20.9 |
| C4N2H10O | Isopropylurea | -50.7 | -51.3 |
| C5N2H8O | 5-Amino-3,4-dimethylisoxazole | -4.8 | -4.7 |
| C5N2H12O | (1-Methylpropyl) urea | -54.7 | -55.3 |
| C5N2H12O | N,N-diethylurea | -43.6 | -45.5 |
| C5N2H12O | Tetramethylurea | -30.8 | -30.7 |
| C4N3H5O | 4-Amino-2(1H)-pyrimidinone | -23 | -22.9 |
| O2 | Oxygen (Singlet) | 12.1 | 12.1 |
| O2 | Oxygen (Triplet) | -16 | -16.1 |
| H2O2 | Hydrogen peroxide | -38.3 | -38.2 |
| CO2 | Carbon dioxide | -75.1 | -75.1 |
| CHO2 | Formate, anion | -112.9 | -101.6 |
| CH2O2 | Formic acid | -92.6 | -92.6 |
| C2H2O2 | trans Glyoxal | -61.4 | -61.4 |
| C2H3O2 | Acetate, anion | -126.7 | -110.0 |
| C2H4O2 | Acetic acid | -101.2 | -101.1 |
| C2H4O2 | Methyl formate | -85.6 | -85.5 |
| C2H6O2 | Dimethyl peroxide | -28.4 | -28.3 |
| C2H6O2 | Ethylene glycol | -106 | -106.0 |
| C3O2 | Carbon suboxide | -23.5 | -23.6 |
| C3H4O2 | 2-Oxo-propanal | -70.9 | -70.9 |
| C3H4O2 | 2-Propenoic acid | -76.2 | -76.2 |
| C3H4O2 | beta-Propiolactone | -68.9 | -68.9 |
| C3H6O2 | 1,3-Dioxalane | -93.1 | -93.0 |
| C3H6O2 | Ethyl formate | -90.2 | -90.2 |
| C3H6O2 | Methyl acetate | -93.7 | -93.7 |
| C3H6O2 | Propionic acid | -105.7 | -105.7 |
| C3H8O2 | 1,3-Propanediol | -110.2 | -110.2 |
| C3H8O2 | 2-Methoxyethanol | -99.8 | -99.8 |
| C3H8O2 | Dimethoxymethane | -94.4 | -94.4 |
| C3H8O2 | Propylene glycol | -107.6 | -107.6 |
| C4H6O2 | 2-Butenoic acid | -85.9 | -85.9 |
| C4H6O2 | 2-Methyl-2-propenic acid | -83.8 | -83.7 |
| C4H6O2 | Diacetyl | -78.8 | -78.9 |
| C4H6O2 | gamma Butyrolactone | -94 | -94.0 |
| C4H6O2 | Methyl 2-propenoate | -68.6 | -68.6 |
| C4H8O2 | 1,1 Dimethoxy ethene | -71 | -70.9 |
| C4H8O2 | 1,3 Dioxan | -94.3 | -94.3 |
| C4H8O2 | 1,4-Dioxane | -89.3 | -89.3 |
| C4H8O2 | Ethyl acetate | -99 | -99.0 |
| C4H10O2 | 1,2-Dimethoxyethane | -93.4 | -93.4 |
| C4H10O2 | 1,4 Butandiol | -115.3 | -115.3 |
| C4H10O2 | Diethyl peroxide | -38.3 | -38.5 |
| C4H10O2 | Dimethyl acetal | -95.2 | -95.2 |
| C5H8O2 | Acetylacetone | -84.3 | -84.4 |
| C5H10O2 | Ethyl propionate | -103.5 | -103.5 |
| C5H10O2 | Isopropyl acetate | -100.5 | -100.6 |
| C5H12O2 | 1,5 Pentandiol | -119.9 | -119.9 |
| C6H4O2 | p-Benzoquinone | -32.9 | -33.0 |
| C6H6O2 | 1,2-Benzenediol | -72.8 | -72.7 |
| C6H6O2 | Hydroquinone | -74 | -74.0 |
| C6H6O2 | Resorcinol | -75 | -75.0 |
| C6H8O2 | 1,3-Cyclohexanedione | -83.6 | -83.7 |
| C6H8O2 | 1,4-Cyclohexanedione | -84.6 | -84.7 |
| C6H10O2 | 2,4-Hexanedione | -88.9 | -89.0 |
| C6H10O2 | 2-Oxepanone | -94.4 | -94.4 |
| C6H10O2 | 3-Methyl-2,4-pentandione | -84.4 | -84.5 |
| C6H10O2 | Ethyl-(E)-2-butenoate | -83.8 | -83.7 |
| C6H12O2 | 1,1-Dimethoxy-2-butene | -79.8 | -79.7 |
| C6H12O2 | 4-Hydroxy-4-methylpentan-2-one | -96.4 | -96.4 |
| C6H12O2 | 5,5-Dimethyl-1,3-dioxane | -93.7 | -93.6 |
| C6H12O2 | cis-2,4-Dimethyl-1,3-dioxane | -100.8 | -100.7 |
| C6H12O2 | Ethyl butanoate | -108.1 | -108.1 |
| C6H12O2 | Hexanoic acid | -119.8 | -119.7 |
| C6H12O2 | Methyl 2-methylbutanoate | -104 | -103.9 |
| C6H12O2 | Methyl 2,2-dimethyl-propanoate | -96.3 | -96.2 |
| C6H12O2 | Methyl 3-methylbutanoate | -104 | -103.9 |
| C6H12O2 | Methyl pentanoate | -107.6 | -107.5 |
| C6H12O2 | t-Butyl acetate | -96.6 | -96.5 |
| C6H12O2 | trans 4,5-Dimethyl-1,3-dioxane | -98 | -97.9 |
| C6H14O2 | 1,1-Diethoxy ethane | -105.8 | -106.5 |
| C6H14O2 | 1,1-Dimethoxy-butane | -104 | -104.0 |
| C6H14O2 | 1,2-Diethoxy ethane | -104.2 | -104.1 |
| C6H14O2 | 1,6-Hexanediol | -124.7 | -124.7 |
| C6H14O2 | 2,3-Dimethyl-2,3-butanediol | -99.2 | -99.2 |
| C7H6O2 | 3-(2-Furanyl)-2-propenal | -27.8 | -27.8 |
| C7H6O2 | Benzoic acid | -65.9 | -67.7 |
| C7H6O2 | Phenyl formate | -50.1 | -50.0 |
| C7H6O2 | Tropolone | -40.1 | -40.1 |
| C7H8O2 | 3-Methyl-1,2-benzenediol | -80.3 | -80.3 |
| C7H8O2 | 4-Methyl 1,2-Benzenediol | -80.4 | -80.3 |
| C7H10O2 | Ethyl 2-methylene-3-butenoate | -57.6 | -57.6 |
| C7H10O2 | Ethyl 2-pentynoate | -56.2 | -56.2 |
| C7H10O2 | Ethyl 3-pentynoate | -58.3 | -58.2 |
| C7H10O2 | Ethyl 4-pentynoate | -47.1 | -47.0 |
| C7H12O2 | 3,5-Heptanedione | -93.5 | -93.6 |
| C7H12O2 | 3-Ethyl-2,4-pentanedione | -87.8 | -87.8 |
| C7H12O2 | 5-Methyl-2,4-hexanedione | -90.9 | -91.0 |
| C7H12O2 | Butyl 2-propenoate | -83.4 | -83.3 |
| C7H12O2 | Ethyl (Z)-2-pentenoate | -88.4 | -88.4 |
| C7H12O2 | Ethyl (Z)-3-pentenoate | -89.6 | -89.5 |
| C7H12O2 | Ethyl 4-pentenoate | -82.8 | -82.7 |
| C7H12O2 | Ethyl trans-2-pentenoate | -88.4 | -88.4 |
| C7H12O2 | Heptanolactone | -92.4 | -92.4 |
| C7H12O2 | Isopropyl 2-butenoate | -85.4 | -85.3 |
| C7H12O2 | Propyl (E)-2-butenoate | -88.6 | -88.5 |
| C7H14O2 | (2a,4a,6b)-2,4,6-Trimethyl-1,3-dioxane | -103.4 | -103.4 |
| C7H14O2 | 1,1-Dimethoxycyclopentane | -97.2 | -97.3 |
| C7H14O2 | 1,1-Dimethylpropyl acetate | -97.4 | -97.3 |
| C7H14O2 | Ethyl 2-methylbutanoate | -108.5 | -108.5 |
| C7H14O2 | Ethyl 3-methylbutanoate | -108.5 | -108.4 |
| C7H14O2 | Ethyl pentanoate | -112.8 | -112.8 |
| C7H14O2 | Methyl 3,3-dimethylbutanoate | -99.7 | -99.6 |
| C7H14O2 | Methyl hexanoate | -112.3 | -112.2 |
| C7H16O2 | 1,3-Diethoxypropane | -108.3 | -108.2 |
| C7H16O2 | 1,7-Heptanediol | -129.4 | -129.4 |
| C8H8O2 | m-Methylbenzoic acid | -75.5 | -75.3 |
| C8H8O2 | Methyl benzoate | -60.4 | -60.3 |
| C8H8O2 | o-Methylbenzoic acid | -73.4 | -73.3 |
| C8H8O2 | p-Methylbenzoic acid | -75.5 | -75.4 |
| NO2 | Nitrogen dioxide, cation | 240.6 | 240.7 |
| NO2 | Nitrogen dioxide | -6.1 | -6.0 |
| NHO2 | Nitrous acid, trans | -40.7 | -40.7 |
| CNH3O2 | Methyl nitrite | -36.7 | -36.7 |
| CNH3O2 | Nitromethane | 3.3 | 3.3 |
| C2NH5O2 | Ethyl nitrite | -42 | -42.0 |
| C2NH5O2 | Glycine | -95.7 | -95.7 |
| C2NH5O2 | Methyl carbamate | -87.3 | -89.0 |
| C2NH5O2 | Nitroethane | -2.1 | -2.1 |
| C3NH7O2 | Alanine | -98.8 | -98.7 |
| C3NH7O2 | beta-Alanine | -99.1 | -99.0 |
| C3NH7O2 | Isopropylnitrite | -44.8 | -44.7 |
| C3NH7O2 | N-Methyl glycine | -94.1 | -94.0 |
| C3NH7O2 | Propyl nitrite | -47 | -47.0 |
| C3NH7O2 | Urethane | -92.6 | -94.3 |
| C4NH5O2 | Methyl cyanoacetate | -57.1 | -57.1 |
| C4NH5O2 | Succinimide | -87.8 | -87.7 |
| C4NH9O2 | 2-Nitrobutane | -10.2 | -10.2 |
| C4NH9O2 | 2-Nitroisobutane | -3.7 | -3.7 |
| C4NH9O2 | 4-Aminobutanoic acid | -104.3 | -104.3 |
| C4NH9O2 | Isobutyl nitrite | -48 | -47.9 |
| C4NH9O2 | n-Butyl nitrite | -51.6 | -51.6 |
| C4NH9O2 | Sec-butyl nitrite | -48.3 | -48.2 |
| C4NH9O2 | t-Butyl nitrite | -42.6 | -42.6 |
| C4NH11O2 | Diethanolamine | -103.4 | -103.4 |
| C5NH5O2 | N-Methylmaleimide | -51.2 | -51.1 |
| C5NH7O2 | Glutarimide | -92.1 | -92.0 |
| C5NH7O2 | N-Methylsuccinimide | -86.1 | -86.1 |
| C5NH9O2 | Proline | -101 | -101.0 |
| C5NH11O2 | 5-Aminovaleric acid | -108.4 | -109.1 |
| C5NH11O2 | N,N-Dimethylglycine methyl ester | -81.9 | -81.9 |
| C5NH11O2 | tert-Pentyl nitrite | -44.2 | -44.2 |
| C5NH11O2 | Valine | -101.1 | -101.1 |
| C5NH13O2 | 1,1-Dimethoxy-trimethylamine | -84.5 | -84.4 |
| C6NH5O2 | Niacin | -60.1 | -60.1 |
| C6NH5O2 | Nitrobenzene | 37.5 | 35.8 |
| C6NH9O2 | Ethyl 2-cyanopropionate | -64.7 | -64.6 |
| C6NH13O2 | Ethyl N,N-dimethylglycinate | -86.5 | -86.4 |
| C6NH13O2 | Hexanoic acid, 6-amino- | -112.9 | -112.9 |
| C6NH13O2 | Isoleucine | -103.3 | -103.2 |
| C6NH13O2 | Leucine | -106.8 | -106.7 |
| C6NH13O2 | Methyl N,N-dimethylalaninate | -81.7 | -81.6 |
| C6NH15O2 | N,N-Dimethylacetamide dimethyl acetal | -78.5 | -78.4 |
| C7NH7O2 | m-Aminobenzoic acid | -67 | -67.0 |
| C7NH7O2 | o-Aminobenzoic acid | -66.4 | -66.3 |
| C7NH7O2 | p-Aminobenzoic acid | -67.8 | -67.7 |
| C7NH7O2 | p-Nitrotoluene | 28 | 28.1 |
| C7NH7O2 | Phenylnitromethane | 29 | 29.0 |
| C7NH15O2 | Methyl N,N-,a,a-tetramethylglycinate | -73.1 | -73.0 |
| C2N2H4O2 | Oxalamide | -73.3 | -76.3 |
| C2N2H6O2 | N-Nitrodimethylamine | 22.3 | 22.3 |
| C3N2H6O2 | Acetyl-urea | -80.1 | -82.4 |
| C3N2H6O2 | Propanediamide | -80 | -82.2 |
| C4N2H4O2 | Pyrazine-1,4-dioxide | 71.7 | 71.7 |
| C4N2H4O2 | Uracil | -64.8 | -64.7 |
| C5N2H6O2 | Thymine | -72.7 | -72.5 |
| C6N2H6O2 | m-Nitroaniline | 36.4 | 36.5 |
| C6N2H6O2 | p-Nitroaniline | 35.5 | 35.6 |
| C6N2H14O2 | Lysine | -104.4 | -104.4 |
| C2N3H5O2 | Imidodicarbonic diamide | -76.3 | -77.8 |
| O3 | Ozone | 48.5 | 48.5 |
| C3H6O3 | 1,3,5-Trioxane | -130.3 | -130.2 |
| C3H6O3 | Methyl hydroxyacetate | -137.1 | -137.0 |
| C3H8O3 | Glycerol | -150.8 | -150.8 |
| C4H2O3 | Malaic anhydride | -88.6 | -88.5 |
| C4H6O3 | Acetic anhydride | -132.7 | -132.6 |
| C4H10O3 | Trimethoxymethane | -136 | -135.9 |
| C6H14O3 | 2,5,8-Trioxanonane | -135.5 | -135.4 |
| C7H6O3 | m-Salicylic acid | -115.2 | -115.1 |
| C7H6O3 | o-Salicylic acid | -114.2 | -114.1 |
| C7H6O3 | p-Salicylic acid | -114.6 | -116.0 |
| C7H14O3 | 2,3-Butanediol, 2,3-dimethyl-, monoformate | -121.8 | -121.8 |
| NO3 | Nitrate anion | -67.1 | -66.9 |
| NHO3 | Nitric acid | -17.6 | -17.4 |
| CNH3O3 | Methyl nitrate | -12.5 | -12.3 |
| C2NH3O3 | Oxamic acid | -126.5 | -127.3 |
| C2NH5O3 | Ethyl nitrate | -18 | -17.8 |
| C3NH7O3 | Serine | -141.3 | -141.3 |
| C4NH3O3 | 2-Nitrofuran | 7.8 | 7.9 |
| C4NH9O3 | Threonine | -142.6 | -142.5 |
| C6NH5O3 | m-Nitrophenol | -11.5 | -11.4 |
| C6NH5O3 | o-Nitrophenol | -10.3 | -10.2 |
| C6NH5O3 | p-Nitrophenol | -12.6 | -12.5 |
| N2O3 | Dinitrogen trioxide | 13.6 | 12.5 |
| C2H2O4 | Oxalic acid | -170.6 | -170.5 |
| C2H6O4 | Dioxybismethanol | -129.2 | -129.1 |
| C4H4O4 | 1,4-Dioxan-2,5-dione | -154.4 | -154.3 |
| C4H6O4 | Dimethyl oxalate | -164.1 | -164.0 |
| C4H8O4 | 1,3,5,7-Tetroxane | -168.5 | -168.3 |
| C5H8O4 | Dimethyl malonate | -170.4 | -170.4 |
| C5H8O4 | Ethylmalonic acid | -190.3 | -190.2 |
| C5H8O4 | Methylene diacetate | -178 | -178.2 |
| C5H12O4 | Tetramethoxymethane | -181.3 | -181.1 |
| C6H10O4 | 1,1-Diacetoxyethane | -180.6 | -180.5 |
| C6H10O4 | Dimethyl methylmalonate | -172.2 | -172.1 |
| C4NH7O4 | Aspartic acid | -182.8 | -182.7 |
| N2O4 | Dinitrogen tetroxide | 29.9 | 30.2 |
| CN2H2O4 | Dinitromethane | 27.8 | 27.9 |
| CN3H3O4 | Methyldinitramine | 53 | 53.2 |
| C3H4O5 | Tartronic acid | -226.5 | -226.4 |
| C5H10O5 | 1,3,5,7,9-pentaoxecane | -209.1 | -208.8 |
| N2O5 | Dinitrogen pentoxide | 34.1 | 34.4 |
| C10H10O | 4-Phenyl-3-buten-2-one | -2.9 | -2.8 |
| C10H14O | 2-Adamantone | -53.7 | -53.7 |
| C10H14O | 2-Isopropyl-4-methylphenol | -42.6 | -42.5 |
| C10H14O | 2-Isopropyl-5-methylphenol | -44.8 | -44.8 |
| C10H14O | 2-Isopropyl-6-methylphenol | -40.6 | -40.5 |
| C10H14O | 2-Methyl-5-isopropylphenol | -45.5 | -45.5 |
| C10H14O | 3-Isopropyl-2-methylphenol | -39.1 | -39.1 |
| C10H14O | 3-Methyl-2-isopropylphenol | -36.5 | -38.8 |
| C10H14O | 3-Methyl-5-isopropylphenol | -47 | -46.9 |
| C10H14O | 4-Isopropyl-2-methylphenol | -45.6 | -45.6 |
| C10H14O | 4-Isopropyl-3-methylphenol | -40.4 | -40.3 |
| C10H14O | 4-Methyl-3-isopropylphenol | -40.3 | -40.3 |
| C10H14O | m-tert-Butylphenol | -32.1 | -32.1 |
| C10H14O | o-sec-Butylphenol | -38.2 | -38.1 |
| C10H14O | o-tert-Butylphenol | -26.8 | -26.7 |
| C10H14O | p-sec-Butylphenol | -43.4 | -43.4 |
| C10H14O | p-tert-Butyl phenol | -32.7 | -32.6 |
| C10H16O | 1-Adamantol | -65.9 | -65.9 |
| C10H16O | 2-Adamantol | -65.2 | -65.2 |
| C10H16O | Camphor | -33.9 | -33.9 |
| C10H16O | Octahydro-3a-methyl-cis-2H-inden-2-one | -60.7 | -60.7 |
| C10H16O | Octahydro-3a-methyl-trans-2H-inden-2-one | -54.2 | -54.2 |
| C10H18O | Beta-caran-3-ol | -45.3 | -46.8 |
| C10H18O | Cyclodecanone | -63.2 | -63.3 |
| C10H20O | 2,2,5,5-Tetramethyl-3-hexanone | -54.6 | -54.6 |
| C10H22O | Decanol | -100.8 | -100.8 |
| C10H22O | Dipentyl ether | -89.4 | -89.4 |
| C11H14O | 2,4,5-Trimethyl-acetophenone | -35.4 | -35.4 |
| C11H14O | 2,4,6-Trimethyl-acetophenone | -34.7 | -34.7 |
| C11H16O | 2-tert-Butyl-p-cresol | -34.3 | -34.3 |
| C11H16O | 3-Methyl-2-phenylbutane-2-ol | -25.7 | -25.6 |
| C11H20O | Cycloundecanone | -66.3 | -66.4 |
| C11H22O | 2,2,6,6-Tetramethyl-4-heptanone | -60.4 | -60.4 |
| C11H22O | Dipentyl ketone | -86.7 | -87.8 |
| C11H24O | Decyl methyl ether | -94.4 | -94.5 |
| C12H8O | Dibenzofuran | 14.2 | 14.4 |
| C12H10O | m-Hydroxybiphenyl | -1.8 | -1.7 |
| C12H10O | o-Hydroxybiphenyl | -0.8 | -0.7 |
| C12H10O | p-Hydroxybiphenyl | -2.1 | -2.1 |
| C12H16O | Isobutyl phenyl ketone | -31.9 | -31.9 |
| C12H18O | 2,6-Diisopropylphenol | -44 | -44.7 |
| C13H10O | Benzophenone | 15.9 | 16.0 |
| C14H10O | Anthone | 12.2 | 12.3 |
| C9NH7O | 2(1H)-Quinolinone | -3.4 | -3.4 |
| C9NH7O | 3-Phenyl isoxazole | 40.5 | 40.6 |
| C9NH7O | 4-Quinolinol | -3.1 | -3.1 |
| C9NH7O | 5-Phenyl isoxazole | 41.5 | 41.6 |
| C9NH7O | 8-Quinolinol | -4.7 | -4.6 |
| C9NH7O | a-Cyanoacetophenone | 17.8 | 17.9 |
| C9NH11O | 2,4,6-Trimethylnitrosobenzene | 9.2 | 9.3 |
| C9NH11O | N,N-Dimethyl benzamide | -9.3 | -9.3 |
| C9NH13O | N,N-Dimethyamino-2,4,6-heptatriene-7-al | 13.7 | 13.8 |
| C9NH17O | 2,2,6,6-Tetramethyl-4-piperidinone | -39.6 | -39.7 |
| C10NH9O | 2-Methyl-4-hydroxyquinoline | -11.8 | -11.8 |
| C10NH9O | 2-Methyl-8-quinolinol | -13.6 | -13.6 |
| C10NH9O | 3-Methyl-5-phenyl isoxazole | 29.7 | 29.9 |
| C10NH9O | 4-Methyl-2-hydroxyquinoline | -14.8 | -14.7 |
| C10NH9O | 5-Methyl-3-phenyl isoxazole | 29.5 | 29.6 |
| C10NH9O | beta-Cyanopropiophenone | 12.3 | 12.4 |
| C10NH11O | 2,4,6-Trimethylbenzonitrile, N-oxide | 48.5 | 48.6 |
| C10NH13O | 2-(Dimethylamino)-acetophenone | -4.7 | -4.7 |
| C10NH13O | N,N,4-Trimethyl benzamide | -17.1 | -17.1 |
| C11NH13O | (E)-3-(Methylamino)-1-phenyl-but-2-enone | 2.8 | 2.9 |
| C11NH15O | 1-Propanone, 2-(dimethylamino)-1-phenyl- | -4.8 | -4.9 |
| C11NH15O | 2-Propanamine, 2-methyl-N-(phenylmethylene)-, N-oxide | 50.3 | 50.4 |
| C11NH17O | 1-Adamantanecarboxamide | -51.2 | -52.4 |
| C11NH23O | N,N-Dimethylnonamide | -74 | -74.1 |
| C12NH9O | Phenoxazine | 16.6 | 16.8 |
| C12NH17O | 2-(Diethylamino)-1-phenylethanone | -12 | -12.0 |
| C13NH9O | 9,10-Dihydro-9-oxoacridine | 17.6 | 17.7 |
| C13NH11O | Benzenamine, N-(phenylmethylene)-, N-oxide | 85.9 | 86.1 |
| C13NH19O | 1-Propanone, 2-(diethylamino)-1-phenyl- | -11.7 | -11.8 |
| C13NH21O | N,N-Dimethyl-1-adamantylcarboxamide | -43.8 | -43.8 |
| C9H10O2 | 1,3-Dioxolane-2-phenyl | -61.3 | -61.2 |
| C9H10O2 | 2,3-Dimethylbenzoic acid | -77.4 | -77.4 |
| C9H10O2 | 2,4-Dimethylbenzoic acid | -81 | -80.9 |
| C9H10O2 | 2,5-Dimethylbenzoic acid | -81.1 | -81.0 |
| C9H10O2 | 2,6-Dimethylbenzoic acid | -78.2 | -78.1 |
| C9H10O2 | 3,4-Dimethylbenzoic acid | -80.6 | -80.6 |
| C9H10O2 | 3,5-Dimethylbenzoic acid | -83 | -82.9 |
| C9H10O2 | 3-Ethylbenzoic acid | -80.2 | -80.2 |
| C9H10O2 | 4-Ethylbenzoic acid | -80.3 | -80.3 |
| C9H10O2 | Methyl 4-methylbenzoate | -65.9 | -65.8 |
| C10H8O2 | 1,2-Naphthalenediol | -54.7 | -54.7 |
| C10H8O2 | 1,3-Naphthalenediol | -56.5 | -56.5 |
| C10H8O2 | 1,4-Naphthalenediol | -53.9 | -53.9 |
| C10H8O2 | 2,3-Naphthalenediol | -55.8 | -55.7 |
| C10H10O2 | 1-Phenyl-1,3-butanedione | -51.2 | -51.9 |
| C10H12O2 | 2,3,4-Trimethylbenzoic acid | -80.9 | -80.9 |
| C10H12O2 | 2,3,5-Trimethylbenzoic acid | -85 | -85.0 |
| C10H12O2 | 2,3,6-Trimethylbenzoic acid | -82 | -81.9 |
| C10H12O2 | 2,4,5-Trimethylbenzoic acid | -86.2 | -86.1 |
| C10H12O2 | 2,4,6-Trimethylbenzoic acid | -85.7 | -85.6 |
| C10H12O2 | 2-Isopropyl benzoic acid | -77.5 | -77.5 |
| C10H12O2 | 2-Methyl-2-phenyl-1,3-dioxolane | -62.2 | -62.2 |
| C10H12O2 | 3,4,5-Trimethylbenzoic acid | -82.1 | -82.1 |
| C10H12O2 | 3-Isopropyl benzoic acid | -80.8 | -80.7 |
| C10H12O2 | 4-Isopropyl benzoic acid | -80.9 | -80.9 |
| C10H14O2 | 2-Isopropyl-6-methyl-pyrocatechol | -89.7 | -89.7 |
| C10H18O2 | Cyclohexyl butanoate | -119.1 | -119.1 |
| C10H20O2 | Ethyl octanoate | -127 | -127.0 |
| C10H22O2 | 1,10-Decanediol | -143.5 | -143.6 |
| C11H8O2 | 1-Naphthoic acid | -48.4 | -48.3 |
| C11H8O2 | Isonaphthoic acid | -50.6 | -50.5 |
| C11H14O2 | 2,3,4,5-Tetramethylbenzoic acid | -84.5 | -84.4 |
| C11H14O2 | 2,3,4,6-Tetramethylbenzoic acid | -85.5 | -85.4 |
| C11H14O2 | 2,3,5,6-Tetramethylbenzoic acid | -85.8 | -85.8 |
| C11H14O2 | p-tert-Butyl benzoic acid | -73.6 | -73.6 |
| C11H20O2 | Oxacyclododecan-2-one | -111.8 | -111.8 |
| C11H22O2 | Ethyl nonanoate | -131.7 | -131.7 |
| C11H24O2 | 1,1-Dibutoxypropane | -128.5 | -128.5 |
| C12H16O2 | 5,5-Dimethyl-2-phenyl-1,3-dioxane | -62 | -61.9 |
| C12H16O2 | Pentamethylbenzoic acid | -83.2 | -83.2 |
| C12H22O2 | 2,2,6,6-Tetramethyl-3,5-heptanedione | -89.9 | -90.0 |
| C12H24O2 | Ethyl decanoate | -136.4 | -136.4 |
| C13H8O2 | Xanthone | -22.3 | -22.3 |
| C13H10O2 | Phenyl benzoate | -27 | -26.9 |
| C8NH9O2 | 2,6-Dimethylnitrobenzene | 24.6 | 24.8 |
| C8NH9O2 | 2-Amino-2-phenylacetic acid | -63.8 | -63.7 |
| C8NH9O2 | 2-Nitro-m-xylene | 24.6 | 24.8 |
| C8NH9O2 | N-Phenylglycine | -62.6 | -62.6 |
| C8NH17O2 | 8-Aminocaprylic acid | -122.5 | -123.3 |
| C9NH7O2 | 2-Methyl-1H-isoindole-1,3(2H)-dione | -45.9 | -45.9 |
| C9NH11O2 | Nitromesitylene | 17.2 | 17.3 |
| C9NH11O2 | Phenylalanine | -68.8 | -68.8 |
| C10NH7O2 | 1-Nitroso-2-naphthalenol | -0.4 | -0.4 |
| C10NH13O2 | N,N-Dimethyl 4-methoxybenzamide | -49 | -49.0 |
| C10NH15O2 | 1-Nitroadamantane | -4.6 | -4.6 |
| C10NH15O2 | 2-Nitroadamantane | -3.8 | -3.8 |
| C8N2H8O2 | Isophthalamide | -51.4 | -53.3 |
| C8N2H8O2 | Teraphthalamide | -51.2 | -53.0 |
| C8N2H10O2 | N,N-Dimethyl-m-nitroaniline | 43.4 | 43.6 |
| C8N2H10O2 | N,N-Dimethyl-p-nitroaniline | 43.3 | 43.4 |
| C11N2H12O2 | Tryptophan | -48.3 | -48.2 |
| C6N3H9O2 | Histidine | -60.4 | -61.3 |
| C6N4H14O2 | Argenine | -75.7 | -75.6 |
| C10H14O3 | Trimethoxymethyl benzene | -98.2 | -98.1 |
| C10H22O3 | 1-tert-Butoxy-3-propoxy-2-propanol | -151.7 | -151.7 |
| C11H24O3 | 1-Butoxy-3-tert-butyl-2-propanol | -157.2 | -157.2 |
| C9NH11O3 | Tyrosine | -117 | -117.0 |
| C4N2H4O3 | Barbituric acid | -118.8 | -118.7 |
| C4N2H8O3 | Asparagine | -129 | -130.4 |
| C4N2H8O3 | GLY-GLY | -128.9 | -129.2 |
| C5N2H10O3 | Glutamine | -132.8 | -134.4 |
| C6N2H12O3 | ALA-ALA | -133.9 | -134.2 |
| C9N2H6O3 | 8-Hydroxy-5-nitroquinoline | 11.6 | 11.8 |
| C10N2H16O3 | PRO-PRO | -136.1 | -136.1 |
| C10N2H20O3 | VAL-VAL | -140.1 | -140.2 |
| C3N3H3O3 | 1,3,5-Triazine-2,4,6(1H,3H,5H)-trione | -119 | -118.8 |
| C6N3H9O3 | 1,3,5-Trimethyl-s-triazine-2,4,6-trione | -103.7 | -103.5 |
| C6N3H9O3 | 2,4,6-Trimethoxy-s-triazine | -100 | -99.7 |
| C7H12O4 | 2,2-Diacetoxypropane | -171 | -171.0 |
| C7H12O4 | Diethyl malonate | -181.1 | -181.0 |
| C7H12O4 | Dimethyl dimethylmalonate | -166.6 | -166.5 |
| C7H16O4 | 3,5,7,9-Tetraoxyundecane | -184.3 | -189.8 |
| C8H16O4 | 12-Crown-4 | -163.9 | -164.0 |
| C10H6O4 | 5,8-Dihydroxy-1,4-naphthalenedione | -113.6 | -113.6 |
| C10H10O4 | Dimethyl isophthalate | -141.1 | -141.0 |
| C10H10O4 | Dimethyl phthalate | -138.3 | -138.2 |
| C10H22O4 | 1-(tert-Butyldioxy)-3-propoxy-2-propanol | -130 | -130.0 |
| C11H12O4 | Benzal diacetate | -146 | -145.8 |
| C11H24O4 | 1-Butoxy-1-tert-butyldioxy-2-propanol | -139 | -138.9 |
| C6NH5O4 | 4-Nitrocatechol | -59.3 | -59.2 |
| C7NH5O4 | p-Nitrobenzoic acid | -51.2 | -51.2 |
| C6N2H4O4 | m-Dinitrobenzene | 54.6 | 54.8 |
| C6N2H4O4 | o-Dinitrobenzene | 54.6 | 54.8 |
| C6N2H4O4 | p-Dinitrobenzene | 54.4 | 54.6 |
| C7N2H6O4 | 2,4-Dinitrotoluene | 48.7 | 48.9 |
| C7N2H6O4 | Dinitrophenylmethane | 56 | 56.2 |
| C8H18O5 | 3,5,7,9,11-Pentaoxa-tridecane | -230.1 | -235.4 |
| C10H20O5 | 15-Crown-5 | -208.6 | -208.6 |
| C6N2H12O5 | SER-SER | -220.2 | -220.9 |
| C8N2H16O5 | THR-THR | -217.4 | -217.6 |
| C7H10O6 | Trimethyl methanetricarboxylate | -242.6 | -242.5 |
| C2N3H3O6 | 1,1,1-Trinitroethane | 56.2 | 56.4 |
| CN4O8 | Tetranitromethane | 94.8 | 95.0 |
| C3N3H5O9 | Glycerol trinitrate | -6.7 | -6.3 |
| C14NH21O | 4-Isopropylbenzylidene t-butylamine N-oxide | 37.1 | 37.2 |
| C14H8O2 | 9,10-Anthroquinone | -10.7 | -13.2 |
| C14H8O2 | 9,10-Phenanthroquinone | -10.7 | -10.7 |
| C13NH11O2 | Phenol, 2-[(phenylimino)methyl]-, N-oxide | 38.1 | 38.2 |
| C14NH13O2 | Benzenamine, N-[(4-methoxyphenyl)methylene]-, N-oxide | 45.7 | 45.8 |
| C15NH17O2 | N-(3-Phenoxy-2-hydroxypropyl)aniline | -28.4 | -28.4 |
| C14H10O3 | Benzoic acid, anhydride | -66.3 | -66.1 |
| C12N2H24O3 | ILE-ILE | -144.5 | -144.8 |
| C12N2H24O3 | LEU-LEU | -150.2 | -150.6 |
| C12H14O4 | 1,1-Ethanediol, 2-phenyl-, diacetate | -150.8 | -151.0 |
| C12H14O4 | Benzyl diacetate | -151.1 | -151.0 |
| C14H6O4 | 1,4,9,10-Anthracenetetrone | -62.1 | -62.1 |
| C14H8O4 | 1,4-Dihydroxy-9,10-anthracenedione | -105 | -104.9 |
| C14H10O4 | Diphenyl oxalate | -97.2 | -97.0 |
| C14H12O4 | Dimethyl naphthalene-2,6-dicarboxylate | -124.1 | -124.0 |
| C10H16O6 | Triethyl methanetricarboxylate | -258.6 | -258.5 |
| C11H18O6 | Triethyl 1,1,1-ethanetricarboxylate | -253 | -252.9 |
| C7N3H5O6 | 2,4,6-Trinitrotoluene | 74.4 | 74.6 |
| C9NH9O7 | 2-(Diacetoxymethyl)-5-nitrofuran | -161.4 | -161.3 |
| C7N3H5O7 | 2,4,6-Trinitroanisole | 39.7 | 39.9 |
| C8N3H7O7 | 2,4,6-Trinitrophenetole | 36.6 | 36.7 |
| F | Fluorine, atom | 18.9 | 18.9 |
| F | Fluoride, anion | -17.1 | -17.1 |
| HF | Hydrogen fluoride | -59.7 | -59.7 |
| CF | Fluoromethylidyne radical | 38.2 | 38.2 |
| CH2F | Fluoromethyl, cation | 182.8 | 182.8 |
| CH3F | Fluoromethane | -60.9 | -60.9 |
| C2HF | Fluoroacetylene | 15.6 | 15.6 |
| C2H3F | Fluoroethylene | -34.5 | -34.6 |
| C2H4F | CH3CHF, cation | 164.7 | 164.7 |
| C2H5F | Fluoroethane | -65.1 | -65.1 |
| C3H7F | 2-Fluoropropane | -66.6 | -66.7 |
| C6H5F | Fluorobenzene | -25.3 | -25.3 |
| C6H11F | Fluorocyclohexane | -76.5 | -76.5 |
| C9H19F | 1-Fluorononane | -98.5 | -98.5 |
| CNF | Cyanogen fluoride | -2.4 | -2.3 |
| OF | Fluorine oxide | 20.4 | 20.4 |
| HOF | Hypofluorous acid | -18.6 | -18.6 |
| CHOF | HCOF | -88.8 | -88.8 |
| C2H3OF | Acetyl fluoride | -96.5 | -96.5 |
| NOF | Nitrosyl fluoride | -24.8 | -24.7 |
| O2F | Fluorine dioxide | 22.7 | 22.8 |
| C7H5O2F | m-fluorobenzoic acid | -113.3 | -113.2 |
| NO2F | Fluorine nitrite | 0.6 | 0.8 |
| NO3F | Fluorine nitrate | 27.9 | 28.2 |
| CN2HO4F | Fluorodinitromethane | -18.3 | -18.2 |
| F2 | Fluorine molecule | 7.3 | 7.4 |
| CF2 | Difluoromethylene | -65.2 | -65.2 |
| CHF2 | Difluoromethyl, cation | 132.4 | 132.4 |
| CH2F2 | Difluoromethane, cation | 177.6 | 177.6 |
| CH2F2 | Difluoromethane | -111.8 | -111.7 |
| C2F2 | Difluoroacetylene | -21 | -21.0 |
| C2H2F2 | gem-Difluoroethylene | -83.7 | -83.7 |
| C2H3F2 | CH3CF2, cation | 116.5 | 116.6 |
| C2H4F2 | 1,1-Difluoroethane | -113.5 | -113.4 |
| C6H4F2 | 1,2-Difluorobenzene | -70.7 | -70.6 |
| C6H4F2 | 1,3-Difluorobenzene | -71 | -71.0 |
| C6H4F2 | 1,4-Difluorobenzene | -71.1 | -71.1 |
| C4NH9F2 | t-Butyldifluoroamine | -21.2 | -21.1 |
| C7NH7F2 | N,N'-Difluorobenzylamine | 11.3 | 11.3 |
| N2F2 | cis-Difluorodiazene | -2.3 | -2.3 |
| N2F2 | trans-Difluorodiazene | 2.3 | 2.3 |
| OF2 | Difluorine oxide | 18.2 | 18.3 |
| COF2 | Carbonyl fluoride | -138.6 | -138.5 |
| CF3 | Trifluoromethyl, cation | 100.9 | 101.0 |
| CF3 | Trifluoromethyl | -138.7 | -138.6 |
| CF3 | Trifluoromethyl, anion | -178.8 | -178.7 |
| CHF3 | Trifluoromethane | -163.8 | -163.7 |
| C2HF3 | Trifluoroethylene | -131.1 | -131.1 |
| C2H2F3 | CF3CH2, cation | 121.2 | 121.3 |
| C2H2F3 | CH2F.CF2, radical cation | 82.4 | 82.4 |
| C2H2F3 | CF3CH2 radical | -131.2 | -131.1 |
| C2H3F3 | 1,1,1-Trifluoroethane | -164.4 | -164.3 |
| C7H5F3 | Trifluoromethylbenzene | -127.7 | -127.5 |
| NF3 | Nitrogen trifluoride | -34.3 | -34.1 |
| C2NF3 | Trifluoroacetonitrile | -113.3 | -113.1 |
| C2NF3 | Trifluoromethylisocyanide | -90.5 | -90.3 |
| NOF3 | F3NO | 22.7 | 23.0 |
| C2HO2F3 | Trifluoroacetic acid | -238.2 | -238.0 |
| CF4 | Carbon tetrafluoride | -214.2 | -214.0 |
| C2F4 | Tetrafluoroethylene | -175.7 | -175.6 |
| C6H2F4 | 1,2,4,5-Tetrafluorobenzene | -159.3 | -159.2 |
| N2F4 | Tetrafluorohydrazine | -16.2 | -19.6 |
| COF4 | Perfluoromethanol | -163.4 | -163.1 |
| CO2F4 | Bis(fluoroxy)perfluoromethane | -112.5 | -112.1 |
| CNF5 | Pentafluoromethylamine | -163.4 | -163.1 |
| CN3F5 | Pentafluoroguanidine | 6.1 | 6.2 |
| C2F6 | Hexafluoroethane | -299.7 | -299.4 |
| C4F6 | Perfluorobutadiene | -250.1 | -250.0 |
| CN2F6 | Hexafluorodimethylamine | -111.7 | -111.9 |
| C2OF6 | Dimethyl perfluoroether | -357.5 | -357.3 |
| C3OF6 | Perfluoroacetone | -322 | -322.2 |
| C7N2H5O4F | Fluorodinitrophenylmethane | 14.5 | 14.6 |
| C7H4F4 | 1-Fluoro-3-(trifluoro-methyl)benzene | -172.5 | -172.3 |
| C6N2H10F4 | N,N,N',N'-tetrafluoro-1,1-cyclohexanediamine | -24.8 | -24.7 |
| C6N2H12F4 | N,N,N',N'-Tetrafluoro-4-methyl-1,2-pentane | -26.6 | -26.5 |
| C7N2H14F4 | 1,1-Bis(difluoroamine)heptane | -42.5 | -42.5 |
| C6HF5 | Pentafluorobenzene | -201.8 | -201.7 |
| C7H3F5 | 2,3,4,5,6-Pentafluorotoluene | -206.6 | -206.6 |
| C6HOF5 | Pentafluorophenol | -247.9 | -247.9 |
| C6F6 | Hexafluorobenzene | -243.6 | -243.5 |
| C3F8 | Perfluoropropane | -384.6 | -384.3 |
| C4F8 | Perfluorobut-2-ene | -366.2 | -365.9 |
| C4F8 | Perfluorocyclobutane | -363.8 | -363.7 |
| C7F8 | Octafluorotoluene | -340.6 | -340.4 |
| CN4F8 | Octafluoromethanetetramine | -4 | -3.8 |
| C3O2F8 | Perfluorodimethoxymethane | -502.1 | -501.3 |
| C4F10 | n-Perfluorobutane | -469.4 | -469.0 |
| C7H6O4F6 | Hexafluoropentanedioic acid, dimethyl ester | -420 | -419.7 |
| C6F10 | Decafluorocyclohexene | -423.3 | -423.1 |
| C5NF11 | Undecafluoropiperidine | -437.2 | -436.9 |
| C2N5F11 | Tetrakis(difluoroamine)-N-1,1-trifluorodimethylamine | -85.7 | -85.4 |
| C6F12 | Dodecafluorocyclohexane | -522.8 | -522.5 |

Calculated Values for $I.E.$ Using the MNDO Formalism (given in $\mathrm{eV}$)

| **Molecular Formula** | **Molecule Name** | **MOPAC** | **Our Program** |
| --- | --- | --- | --- |
| H2 | Hydrogen | 15.75 | 15.75 |
| CH4 | Methane | 13.86 | 13.87 |
| C2H2 | Acetylene | 11.01 | 11.01 |
| C2H4 | Ethylene | 10.18 | 10.18 |
| C2H6 | Ethane | 12.7 | 12.70 |
| C3 | Carbon, trimer | 11.04 | 11.05 |
| C3H4 | Allene | 10.02 | 10.02 |
| C3H4 | Cyclopropene | 9.88 | 9.88 |
| C3H4 | Propyne | 10.72 | 10.72 |
| C3H6 | Cyclopropane | 11.43 | 11.43 |
| C3H6 | Propene | 9.96 | 9.96 |
| C3H8 | Propane | 12.34 | 12.34 |
| C4H2 | Diacetylene | 9.99 | 9.99 |
| C4H4 | Vinylacetylene | 9.5 | 9.50 |
| C4H4 | Butatriene | 9.01 | 9.01 |
| C4H6 | 1,2-Butadiene | 9.84 | 9.84 |
| C4H6 | 1,3-Butadiene | 9.14 | 9.14 |
| C4H6 | 1-Butyne | 10.68 | 10.68 |
| C4H6 | 2-Butyne | 10.47 | 10.47 |
| C4H6 | Cyclobutene | 9.77 | 9.77 |
| C4H8 | 1-Butene | 9.95 | 9.97 |
| C4H8 | Cyclobutane | 11.8 | 11.81 |
| C4H10 | Isobutane | 12.12 | 12.12 |
| C4H10 | n-Butane, trans | 12.21 | 12.21 |
| C5H6 | Cyclopentadiene | 9.04 | 9.04 |
| C5H8 | Cyclopentene | 9.72 | 9.72 |
| C5H10 | Cyclopentane | 12.06 | 12.06 |
| C5H12 | n-Pentane | 12.16 | 12.16 |
| C5H12 | Neopentane | 12.11 | 12.12 |
| C6H6 | Benzene | 9.39 | 9.39 |
| C6H10 | Cyclohexene | 9.75 | 9.75 |
| C6H12 | Cyclohexane | 11.74 | 11.74 |
| C7H8 | Cycloheptatriene | 8.58 | 8.72 |
| C7H8 | Toluene | 9.28 | 9.28 |
| C8H10 | Ethylbenzene | 9.28 | 9.28 |
| C8H14 | Bicyclo(2.2.2)-octane | 11.4 | 11.41 |
| C10H8 | Naphthalene | 8.58 | 8.57 |
| C10H16 | Adamantane | 11.27 | 11.27 |
| C14H10 | Anthracene | 8.05 | 8.05 |
| NH3 | Ammonia | 11.19 | 11.19 |
| CNH | Hydrogen cyanide | 13.41 | 13.41 |
| CNH5 | Methylamine | 10.56 | 10.56 |
| C2NH3 | Acetonitrile | 12.79 | 12.79 |
| C2NH3 | Methyl isocyanide | 12.24 | 12.24 |
| C2NH5 | Ethyleneimine (Azirane) | 10.68 | 10.68 |
| C2NH7 | Dimethylamine | 10.04 | 10.04 |
| C2NH7 | Ethylamine | 10.5 | 10.50 |
| C3NH3 | Acrylonitrile | 10.61 | 10.61 |
| C3NH5 | Ethyl cyanide | 12.59 | 12.59 |
| C3NH9 | Trimethylamine | 9.59 | 9.59 |
| C4NH5 | Pyrrole | 8.56 | 8.56 |
| C5NH5 | Pyridine | 9.69 | 9.69 |
| C6NH7 | Aniline | 8.75 | 8.75 |
| C7NH5 | Phenyl cyanide | 9.81 | 9.81 |
| N2 | Nitrogen | 14.87 | 14.87 |
| CN2H2 | Diazomethane | 8.66 | 8.67 |
| CN2H6 | Methylhydrazine | 9.66 | 9.66 |
| C2N2 | Cyanogen | 13.2 | 13.20 |
| H2O | Water | 12.19 | 12.19 |
| CO | Carbon monoxide | 13.43 | 13.43 |
| CH2O | Formaldehyde | 11.04 | 11.04 |
| CH4O | Methanol | 11.41 | 11.41 |
| C2H2O | Ketene | 9.29 | 9.29 |
| C2H4O | Acetaldehyde | 10.88 | 10.88 |
| C2H4O | Ethylene oxide | 11.49 | 11.49 |
| C2H6O | Dimethyl ether | 11.04 | 11.04 |
| C2H6O | Ethanol | 11.3 | 11.30 |
| C3H6O | Acetone | 10.75 | 10.75 |
| C3H6O | Propanal | 10.82 | 10.82 |
| C4H4O | Furan | 9.14 | 9.14 |
| C4H8O | Butanal | 10.81 | 10.80 |
| C4H10O | Diethyl ether | 10.91 | 10.91 |
| C7H6O | Benzaldehyde | 9.74 | 9.73 |
| C7H8O | Anisole | 8.84 | 8.84 |
| CNHO | Hydrogen isocyanate | 11.1 | 11.10 |
| CH2O2 | Formic acid | 11.74 | 11.74 |
| C2H2O2 | trans Glyoxal | 10.75 | 10.75 |
| C2H4O2 | Acetic acid | 11.57 | 11.57 |
| C2H4O2 | Methyl formate | 11.61 | 11.61 |
| C2H6O2 | Dimethyl peroxide | 10.57 | 10.57 |
| C3O2 | Carbon suboxide | 10.07 | 10.07 |
| C3H4O2 | beta-Propiolactone | 11.4 | 11.40 |
| C3H6O2 | Methyl acetate | 11.46 | 11.46 |
| C3H6O2 | Propionic acid | 11.52 | 11.51 |
| C3H8O2 | 2-Methoxyethanol | 11.02 | 11.02 |
| C5H8O2 | Acetylacetone | 10.79 | 10.78 |
| C7H6O2 | Benzoic acid | 9.76 | 9.77 |
| CNH3O2 | Methyl nitrite | 11.42 | 11.41 |
| C2NH5O2 | Ethyl nitrite | 11.36 | 11.36 |
| C3NH7O2 | Alanine | 10.81 | 10.81 |
| C6NH5O2 | Nitrobenzene | 10.31 | 10.31 |
| O3 | Ozone | 12.71 | 12.71 |
| C4H2O3 | Malaic anhydride | 11.7 | 11.70 |
| C7H6O3 | o-Salicylic acid | 9.26 | 9.26 |
| C2H2O4 | Oxalic acid | 11.67 | 11.66 |
| N2O4 | Dinitrogen tetroxide | 12.05 | 12.05 |
| N2O5 | Dinitrogen pentoxide | 13.18 | 13.18 |
| HF | Hydrogen fluoride | 14.82 | 14.82 |
| CH3F | Fluoromethane | 13.05 | 13.05 |
| C2HF | Fluoroacetylene | 11.06 | 11.06 |
| C2H3F | Fluoroethylene | 10.17 | 10.17 |
| C2H5F | Fluoroethane | 12.61 | 12.61 |
| C3H7F | 2-Fluoropropane | 12.33 | 12.33 |
| C6H5F | Fluorobenzene | 9.47 | 9.47 |
| NOF | Nitrosyl fluoride | 12.93 | 12.93 |
| C7H5O2F | m-fluorobenzoic acid | 9.83 | 9.83 |
| NO2F | Fluorine nitrite | 12.99 | 12.99 |
| CH2F2 | Difluoromethane | 13.09 | 13.09 |
| C2F2 | Difluoroacetylene | 11.17 | 11.17 |
| C2H2F2 | gem-Difluoroethylene | 10.18 | 10.18 |
| C2H4F2 | 1,1-Difluoroethane | 12.73 | 12.73 |
| N2F2 | trans-Difluorodiazene | 13 | 13.00 |
| OF2 | Difluorine oxide | 13.52 | 13.52 |
| CHF3 | Trifluoromethane | 14.57 | 14.57 |
| C2HF3 | Trifluoroethylene | 10.46 | 10.46 |
| C2H3F3 | 1,1,1-Trifluoroethane | 14.01 | 14.01 |
| C7H5F3 | Trifluoromethylbenzene | 10.07 | 10.07 |
| NF3 | Nitrogen trifluoride | 13.93 | 13.93 |
| C2HO2F3 | Trifluoroacetic acid | 12.73 | 12.72 |
| CF4 | Carbon tetrafluoride | 16.81 | 16.81 |
| C2F4 | Tetrafluoroethylene | 10.74 | 10.74 |
| N2F4 | Tetrafluorohydrazine | 13.19 | 13.04 |
| C2F6 | Hexafluoroethane | 14.5 | 14.49 |
| C3OF6 | Perfluoroacetone | 13 | 13.00 |
| C6HF5 | Pentafluorobenzene | 10.4 | 10.40 |
| C6F6 | Hexafluorobenzene | 10.78 | 10.77 |

Calculated Values for $\left\langle\mu\right\rangle$ Using the MNDO Formalism (given in $\mathrm{Debyes}$)

| **Molecular Formula** | **Molecule Name** | **MOPAC** | **Our Program** |
| --- | --- | --- | --- |
| C3H4 | Cyclopropene | 0.48 | 0.48 |
| C3H4 | Propyne | 0.12 | 0.12 |
| C3H6 | Propene | 0.04 | 0.04 |
| C3H8 | Propane | 0.00 | 0.00 |
| C4H6 | Bicyclobutane | 0.41 | 0.41 |
| C4H6 | Cyclobutene | 0.08 | 0.08 |
| C5H6 | Cyclopentadiene | 0.18 | 0.18 |
| C5H8 | Cyclopentene | 0.06 | 0.05 |
| C6H6 | Fulvene | 0.70 | 0.69 |
| C7H8 | Toluene | 0.05 | 0.05 |
| NH3 | Ammonia | 1.75 | 1.75 |
| CNH | Hydrogen cyanide | 2.50 | 2.50 |
| CNH5 | Methylamine | 1.48 | 1.48 |
| C2NH3 | Acetonitrile | 2.63 | 2.63 |
| C2NH3 | Methyl isocyanide | 2.17 | 2.17 |
| C2NH5 | Ethyleneimine (Azirane) | 1.75 | 1.75 |
| C2NH7 | Dimethylamine | 1.17 | 1.17 |
| C2NH7 | Ethylamine | 1.52 | 1.52 |
| C3NH3 | Acrylonitrile | 2.97 | 2.97 |
| C3NH9 | Trimethylamine | 0.75 | 0.75 |
| C4NH5 | Pyrrole | 1.81 | 1.81 |
| C5NH5 | Pyridine | 1.96 | 1.96 |
| C6NH7 | Aniline | 1.46 | 1.46 |
| CN2H2 | Diazomethane | 1.25 | 1.25 |
| CN2H2 | N=N-CH2- | 1.55 | 1.55 |
| CN2H6 | Methylhydrazine | 0.24 | 0.24 |
| H2O | Water | 1.78 | 1.78 |
| CO | Carbon monoxide | 0.20 | 0.20 |
| CH2O | Formaldehyde | 2.17 | 2.16 |
| CH4O | Methanol | 1.48 | 1.48 |
| C2H2O | Ketene | 1.04 | 1.04 |
| C2H4O | Acetaldehyde | 2.38 | 2.38 |
| C2H4O | Ethylene oxide | 1.92 | 1.92 |
| C2H6O | Dimethyl ether | 1.27 | 1.27 |
| C2H6O | Ethanol | 1.40 | 1.40 |
| C3H6O | Acetone | 2.50 | 2.51 |
| C4H4O | Furan | 0.42 | 0.42 |
| C4H10O | Diethyl ether | 1.09 | 1.10 |
| C6H6O | Phenol | 1.17 | 1.16 |
| C7H8O | Anisole | 1.07 | 1.07 |
| C3NH7O | Dimethylformamide | 3.06 | 3.06 |
| N2O | Nitrous oxide | 0.77 | 0.76 |
| CH2O2 | Formic acid | 1.49 | 1.49 |
| C2H4O2 | Acetic acid | 1.68 | 1.68 |
| C2H4O2 | Methyl formate | 1.63 | 1.63 |
| C3H6O2 | Methyl acetate | 1.75 | 1.75 |
| C3H6O2 | Propionic acid | 1.64 | 1.64 |
| NHO2 | Nitrous acid, trans | 2.28 | 2.28 |
| O3 | Ozone | 1.18 | 1.18 |
| NHO3 | Nitric acid | 2.78 | 2.78 |
| HF | Hydrogen fluoride | 1.99 | 1.99 |
| CH3F | Fluoromethane | 1.76 | 1.76 |
| C2HF | Fluoroacetylene | 1.57 | 1.58 |
| C2H3F | Fluoroethylene | 1.70 | 1.70 |
| C2H5F | Fluoroethane | 1.87 | 1.87 |
| C6H5F | Fluorobenzene | 1.96 | 1.96 |
| CNF | Cyanogen fluoride | 0.89 | 0.89 |
| HOF | Hypofluorous acid | 1.81 | 1.81 |
| CHOF | HCOF | 2.50 | 2.50 |
| NOF | Nitrosyl fluoride | 0.51 | 0.51 |
| NO2F | Fluorine nitrite | 0.66 | 0.66 |
| CH2F2 | Difluoromethane | 2.21 | 2.22 |
| C2H4F2 | 1,1-Difluoroethane | 2.50 | 2.50 |
| N2F2 | cis-Difluorodiazene | 0.02 | 0.02 |
| OF2 | Difluorine oxide | 0.32 | 0.32 |
| COF2 | Carbonyl fluoride | 0.81 | 0.81 |
| CHF3 | Trifluoromethane | 2.23 | 2.23 |
| C2HF3 | Trifluoroethylene | 1.82 | 1.82 |
| C2H3F3 | 1,1,1-Trifluoroethane | 2.87 | 2.87 |
| NF3 | Nitrogen trifluoride | 0.20 | 0.20 |
| C2NF3 | Trifluoroacetonitrile | 0.36 | 0.36 |
| C2HO2F3 | Trifluoroacetic acid | 2.45 | 2.45 |

**Computed Molecular Properties Using Optimized Parameters**

Values for $\Delta H_{f}$ (given in $kcal/mol$)

| **Molecular Formula** | **Molecule Name** | **Ref.** | **Initial** | **Final** |
| --- | --- | --- | --- | --- |
| H | Hydrogen, cation | 365.7 | 326.7 | 315.5 |
| H | Hydrogen, atom | 52.1 | 52.1 | 77.7 |
| H2 | Hydrogen | 0 | 0.7 | -7.9 |
| C | Carbon, cation | 430.6 | 389.4 | 412.3 |
| C | Carbon, atom | 170.9 | 170.9 | 170.3 |
| CH | Methylidyne | 142.4 | 143.3 | 150.1 |
| CH2 | Methylene, singlet | 99.8 | 107.4 | 115.0 |
| CH2 | Methylene, triplet | 99.8 | 73.9 | 65.0 |
| CH3 | Methyl, cation | 261 | 243.9 | 262.8 |
| CH4 | Methane | -17.9 | -12.0 | -7.6 |
| C2H2 | Acetylene | 54.3 | 57.9 | 43.0 |
| C2H3 | Vinyl, cation | 266 | 265.7 | 249.8 |
| C2H3 | Vinyl | 59.6 | 59.0 | 49.6 |
| C2H4 | Ethylene, cation | 257 | 237.7 | 242.9 |
| C2H4 | Ethylene | 12.5 | 15.4 | 17.2 |
| C2H4 | Methylmethylene | 90.3 | 88.3 | 84.7 |
| C2H5 | Ethyl, cation | 216 | 219.6 | 211.1 |
| C2H5 | Ethyl radical | 25 | 10.5 | 13.6 |
| C2H6 | Ethane | -20 | -19.8 | -14.0 |
| C3 | Carbon, trimer | 196 | 220.3 | 215.9 |
| C3H3 | Cyclopropenyl, cation | 257 | 272.5 | 273.7 |
| C3H3 | Propynyl, cation | 281 | 265.4 | 274.5 |
| C3H4 | Allene | 45.6 | 43.9 | 38.4 |
| C3H4 | Cyclopropene | 66.2 | 68.3 | 62.2 |
| C3H4 | Propyne | 44.4 | 41.4 | 31.0 |
| C3H5 | Allyl, cation | 226 | 221.4 | 234.8 |
| C3H5 | Cyclopropyl, cation | 235 | 258.1 | 249.1 |
| C3H5 | Propenyl, cation | 237 | 240.1 | 218.9 |
| C3H5 | Allyl | 40 | 25.3 | 28.5 |
| C3H6 | Cyclopropane | 12.7 | 11.2 | 10.9 |
| C3H6 | Propene | 4.9 | 4.9 | 6.5 |
| C3H7 | i-Propyl radical | 16.8 | -1.6 | 0.7 |
| C3H8 | Propane | -24.8 | -25.0 | -19.8 |
| C4 | Carbon, tetramer | 232 | 271.3 | 208.2 |
| C4H2 | Diacetylene | 113 | 103.2 | 85.0 |
| C4H4 | Vinylacetylene | 72.8 | 65.5 | 56.3 |
| C4H4 | Butatriene | 83 | 71.2 | 65.8 |
| C4H6 | 1,2-Butadiene | 38.8 | 33.5 | 30.6 |
| C4H6 | 1,3-Butadiene | 26 | 28.9 | 29.6 |
| C4H6 | 1-Butyne | 39.5 | 36.1 | 26.8 |
| C4H6 | 2-Butyne | 34.7 | 24.9 | 20.1 |
| C4H6 | Bicyclobutane | 51.9 | 64.0 | 54.2 |
| C4H6 | Cyclobutene | 37.5 | 31.0 | 25.7 |
| C4H6 | Methyl cyclopropene | 58.2 | 53.7 | 50.9 |
| C4H6 | Methylenecyclopropane | 47.9 | 37.8 | 35.4 |
| C4H7 | 2-Butenyl, cation | 200 | 206.9 | 206.9 |
| C4H7 | Cyclobutyl, cation | 213 | 221.3 | 203.6 |
| C4H8 | 1-Butene | -0.2 | -0.3 | 1.5 |
| C4H8 | cis-2-Butene | -1.9 | -4.4 | -3.5 |
| C4H8 | Cyclobutane | 6.8 | -12.0 | -8.7 |
| C4H8 | Isobutene | -4.3 | -2.0 | -1.4 |
| C4H8 | trans-2-Butene | -3 | -5.1 | -3.2 |
| C4H9 | Isobutyl, cation | 176 | 187.3 | 161.3 |
| C4H9 | Isobutyl | 4.5 | -10.1 | -8.3 |
| C4H10 | Isobutane | -32.4 | -26.8 | -24.3 |
| C4H10 | n-Butane, trans | -30.4 | -29.8 | -25.4 |
| C5H5 | Cyclopentadienyl, anion | 21.3 | 18.9 | 4.1 |
| C5H6 | Cyclopentadiene | 32.1 | 32.0 | 23.9 |
| C5H8 | 1,2-Dimethyl cyclopropene | 46.4 | 39.3 | 40.3 |
| C5H8 | 1,4-Pentadiene | 25.3 | 24.5 | 23.3 |
| C5H8 | 1,cis-3-Pentadiene | 19.1 | 20.0 | 19.2 |
| C5H8 | 1,trans-3-Pentadiene | 18.1 | 19.1 | 19.9 |
| C5H8 | Bicyclo(2.1.0)-pentane | 37.3 | 30.1 | 23.3 |
| C5H8 | Cyclopentene | 8.3 | -0.4 | -1.1 |
| C5H8 | Isoprene | 18 | 23.3 | 22.6 |
| C5H8 | Methylene cyclobutane | 29.1 | 10.8 | 10.6 |
| C5H8 | Spiropentane | 44.3 | 33.6 | 32.1 |
| C5H9 | Cyclopentyl, cation | 188 | 194.1 | 174.7 |
| C5H10 | 2-Methyl-2-butene | -9.9 | -10.2 | -10.7 |
| C5H10 | cis-2-Pentene | -6.1 | -8.9 | -8.4 |
| C5H10 | cis-Dimethylcyclopropane | 1.3 | -2.2 | -3.1 |
| C5H10 | Cyclopentane | -18.3 | -30.6 | -25.4 |
| C5H10 | trans-2-Pentene | -7.9 | -10.4 | -8.0 |
| C5H12 | n-Pentane | -35.1 | -34.5 | -30.9 |
| C5H12 | Neopentane | -40.3 | -24.7 | -26.7 |
| C6H6 | Benzene | 19.8 | 21.3 | 21.3 |
| C6H6 | Fulvene | 47.5 | 53.6 | 50.4 |
| C6H8 | (E)-1,3,5-Hexatriene | 40.1 | 42.5 | 42.3 |
| C6H8 | (Z)-1,3,5-Hexatriene | 41.1 | 43.8 | 42.3 |
| C6H8 | 1,3-Cyclohexadiene | 25.4 | 14.4 | 15.3 |
| C6H8 | 1,4-Cyclohexadiene | 25 | 14.3 | 13.5 |
| C6H10 | 1,2-Dimethylcyclobutene | 19.8 | 6.3 | 6.8 |
| C6H10 | 1,5-Hexadiene | 20.1 | 19.5 | 17.6 |
| C6H10 | 1-Methyl cyclopentene | -1.1 | -10.3 | -8.8 |
| C6H10 | 3-Methyl cyclopentene | 2.3 | -3.7 | -5.4 |
| C6H10 | 4-Methyl cyclopentene | 3.5 | -3.1 | -6.3 |
| C6H10 | Bicyclopropyl | 30.9 | 28.5 | 23.9 |
| C6H10 | Cyclohexene | -1.1 | -10.0 | -8.7 |
| C6H11 | Cyclohexyl, cation | 177 | 186.8 | 165.0 |
| C6H12 | 1-Hexene | -10.1 | -9.1 | -9.5 |
| C6H12 | 2,3-Dimethyl-1-butene | -15.7 | -7.3 | -8.5 |
| C6H12 | 2,3-Dimethyl-2-butene | -16.8 | -13.2 | -17.6 |
| C6H12 | (Z)-3-Methyl-2-pentene | -14.8 | -12.4 | -15.5 |
| C6H12 | 4-Methyl-1-pentene | -11.8 | -5.9 | -8.3 |
| C6H12 | Cyclohexane | -29.5 | -34.8 | -31.8 |
| C6H14 | 2,2-Dimethyl butane | -44.4 | -26.1 | -31.7 |
| C6H14 | 2,3-Dimethyl butane | -42.5 | -27.8 | -34.2 |
| C6H14 | 2-Methyl pentane | -41.7 | -34.7 | -35.5 |
| C6H14 | 3-Methyl pentane | -41.1 | -33.1 | -34.9 |
| C6H14 | n-Hexane | -39.9 | -39.2 | -36.5 |
| C7H7 | Benzyl, cation | 212 | 218.0 | 227.9 |
| C7H7 | Tropylium cation | 209 | 207.6 | 213.9 |
| C7H8 | Cycloheptatriene | 43.2 | 33.8 | 35.0 |
| C7H8 | Norbornadiene | 59.7 | 62.8 | 45.6 |
| C7H8 | Toluene | 12 | 13.5 | 13.9 |
| C7H12 | 1,2-Dimethyl cyclopentene | -9.9 | -18.7 | -16.2 |
| C7H12 | 1-Ethyl cyclopentene | -6 | -14.0 | -13.1 |
| C7H12 | 1-Methyl cyclohexene | -19.4 | -17.1 | -15.8 |
| C7H12 | Norbornane | -12.4 | -10.6 | -19.1 |
| C7H14 | 1,1-Dimethyl cyclopentane | -33.1 | -30.1 | -33.0 |
| C7H14 | 1,2-cis-Dimethyl cyclopentane | -31 | -32.4 | -34.4 |
| C7H14 | 1,2-trans-Dimethyl cyclopentane | -32.7 | -35.3 | -35.3 |
| C7H14 | 1,3-cis-Dimethyl cyclopentane | -31.9 | -34.6 | -35.1 |
| C7H14 | 1-Heptene | -14.9 | -13.9 | -15.0 |
| C7H14 | Ethyl cyclopentane | -30.4 | -36.7 | -36.1 |
| C7H14 | Methyl-cyclohexane | -37 | -36.3 | -36.3 |
| C7H16 | 2,2,3-Trimethyl butane | -48.7 | -22.2 | -35.2 |
| C7H16 | 2,2-Dimethyl pentane | -49.3 | -30.4 | -37.5 |
| C7H16 | 2,3-Dimethyl pentane | -47.6 | -32.0 | -38.6 |
| C7H16 | 2,4-Dimethyl pentane | -48.3 | -34.6 | -39.9 |
| C7H16 | 2-Methyl hexane | -46.6 | -39.4 | -40.9 |
| C7H16 | 3,3-Dimethyl pentane | -48.2 | -27.0 | -36.1 |
| C7H16 | 3-Ethyl pentane | -45.3 | -36.0 | -40.9 |
| C7H16 | 3-Methyl hexane | -46 | -37.6 | -40.5 |
| C7H16 | n-Heptane | -44.9 | -43.9 | -42.0 |
| C8H8 | Cubane | 148.7 | 98.9 | 74.5 |
| C8H8 | Cyclooctatetraene | 70.7 | 56.1 | 57.6 |
| C8H8 | Styrene | 35.3 | 37.6 | 37.4 |
| C8H10 | Ethylbenzene | 7.2 | 8.7 | 9.6 |
| C8H10 | m-Xylene | 4.1 | 5.9 | 6.7 |
| C8H10 | o-Xylene | 4.6 | 8.3 | 6.6 |
| C8H10 | p-Xylene | 4.3 | 5.7 | 6.8 |
| C8H12 | 1,5-Cyclooctadiene | 13.7 | 11.3 | 9.1 |
| C8H12 | 4-Vinyl cyclohexene | 16.6 | 13.1 | 9.3 |
| C8H14 | 1-Octyne | 19.3 | 17.0 | 4.7 |
| C8H14 | 2,5-Dimethyl 2,4-hexadiene | -4.6 | -2.4 | -6.0 |
| C8H14 | 2-Octyne | 15.2 | 5.3 | -0.9 |
| C8H14 | 3,4-Dimethyl-(E,E)-2,4-hexadiene | -0.5 | -2.4 | -3.8 |
| C8H14 | 3,4-Dimethyl-(E,Z)-2,4-hexadiene | 0.7 | -1.9 | -2.1 |
| C8H14 | 3,4-Dimethyl-(Z,Z)-2,4-hexadiene | -0.9 | -2.0 | -4.1 |
| C8H14 | 3-Octyne | 14.9 | 4.7 | 0.3 |
| C8H14 | 4-Octyne | 14.4 | 4.6 | -0.1 |
| C8H14 | Bicyclo(2.2.2)-octane | -24.1 | -26.5 | -30.0 |
| C8H16 | 1-Octene | -19.8 | -19.3 | -20.5 |
| C8H16 | Ethylcyclohexane | -41.1 | -39.5 | -41.5 |
| C8H18 | 2,2,3,3-Tetramethyl butane | -54 | -12.8 | -35.8 |
| C8H18 | 2,2,3-Trimethyl pentane | -52.6 | -24.6 | -40.7 |
| C8H18 | 2,2,4-Trimethyl pentane | -53.6 | -29.2 | -43.4 |
| C8H18 | 2,2-Dimethyl hexane | -53.7 | -35.1 | -43.0 |
| C8H18 | 2,3,3-Trimethyl pentane | -51.7 | -24.5 | -40.7 |
| C8H18 | 2,3,4-Trimethyl pentane | -52 | -28.8 | -42.3 |
| C8H18 | 2,3-Dimethyl hexane | -51.1 | -36.6 | -44.3 |
| C8H18 | 2,4-Dimethyl hexane | -52.4 | -37.5 | -45.2 |
| C8H18 | 2,5-Dimethyl hexane | -53.2 | -39.5 | -45.6 |
| C8H18 | 2-Methyl heptane | -51.5 | -44.1 | -46.5 |
| C8H18 | 3,3-Dimethyl hexane | -52.6 | -31.2 | -41.9 |
| C8H18 | 3,4-Dimethyl hexane | -50.9 | -31.6 | -44.9 |
| C8H18 | 3-Ethyl hexane | -50.4 | -39.8 | -47.5 |
| C8H18 | 3-Ethyl-2-methyl pentane | -50.5 | -31.6 | -46.3 |
| C8H18 | 3-Ethyl-3-methyl pentane | -51.4 | -27.8 | -42.3 |
| C8H18 | 3-Methyl heptane | -50.8 | -42.3 | -46.0 |
| C8H18 | 4-Methyl heptane | -50.7 | -42.1 | -46.1 |
| C8H18 | n-Octane | -49.9 | -48.7 | -47.5 |
| C9H10 | alpha-Methyl styrene | 28.3 | 30.5 | 31.0 |
| C9H10 | Cyclopropyl benzene | 36 | 37.6 | 35.1 |
| C9H18 | 1,3,5-Trimethyl cyclohexane | -51.5 | -39.0 | -44.8 |
| C9H18 | cis-cis-trans-1,3,5-Trimethyl cyclohexane | -49.4 | -37.8 | -44.3 |
| C9H20 | 3,3-Diethylpentane | -55.4 | -27.9 | -46.2 |
| C9H20 | n-Nonane | -54.7 | -53.4 | -53.0 |
| C10H8 | Azulene | 73.5 | 72.1 | 72.0 |
| C10H8 | Naphthalene | 36.1 | 38.3 | 39.3 |
| C10H10 | 1,4-Dicyclopropylbuta-1,3-diyne | 134.3 | 117.7 | 104.8 |
| C10H10 | 1-Butynl benzene | 59.4 | 52.0 | 48.5 |
| C10H10 | 2a,4a,6a,6b-Tetrahydrocyclopentapentalene | 53.1 | 47.6 | 37.7 |
| C10H10 | Bulvalene | 79.9 | 63.4 | 59.2 |
| C10H10 | Diisopropenyldiacetylene | 118.1 | 103.0 | 96.7 |
| C10H10 | Tricyclo[6.2.0.0]deca-1(8),2,6-triene | 74 | 54.1 | 48.6 |
| C10H12 | 1,2,6,7-Cyclodecatetraene | 85.1 | 67.8 | 59.4 |
| C10H12 | Dispiro[2.2.2.2]deca-4,9-diene | 72.3 | 65.1 | 58.3 |
| C10H12 | Tetralin | 6.2 | 1.3 | 1.5 |
| C10H14 | (1-Methylpropyl) benzene | -4.2 | 4.5 | 0.7 |
| C10H14 | (2-Methylpropyl) benzene | -5.2 | 3.7 | -0.2 |
| C10H14 | 1,2,3,4-Tetramethyl benzene | -8.6 | 1.0 | -6.6 |
| C10H14 | 1,2,3,4-Tetramethylfulvene | 19.9 | 20.1 | 19.6 |
| C10H14 | 1,2,3,5-Tetramethyl benzene | -10.3 | -3.0 | -6.8 |
| C10H14 | 1,2,4,5-Tetramethyl benzene | -11.3 | -4.6 | -7.5 |
| C10H14 | tert-Butyl benzene | -5.4 | 15.6 | 4.5 |
| C10H14 | Tetrahydrotriquinacene | 3 | -10.8 | -15.9 |
| C10H16 | 1,2,3,4,5-Pentamethyl-1,3-cyclopentadiene | -5.9 | -6.5 | -8.3 |
| C10H16 | Adamantane | -31.9 | -26.5 | -36.4 |
| C10H16 | Camphene | -6.8 | 14.6 | -4.3 |
| C10H16 | Perhydrotriquinacene | -24.5 | -38.9 | -42.2 |
| C10H18 | 1-Methyl-4-(1-methylethyl)-cyclohexene | -26.5 | -20.5 | -29.4 |
| C10H18 | 4-Methyl-1-(1-methylethyl)-cyclohexene | -26.6 | -23.8 | -27.7 |
| C10H18 | cis-Decalin | -40.5 | -37.3 | -41.3 |
| C10H18 | Spiro(4-5)decane | -34.7 | -37.3 | -44.1 |
| C10H18 | trans-Decalin | -43.5 | -41.9 | -46.8 |
| C10H20 | (E)-2,2,5,5-Tetramethyl-3-hexene | -39.9 | -10.9 | -23.1 |
| C10H20 | (Z)-2,2,5,5-Tetramethyl-3-hexene | -30.3 | -0.3 | -22.6 |
| C10H20 | 1-Decene | -29.8 | -28.0 | -31.6 |
| C10H20 | Butyl cyclohexane | -51 | -48.8 | -52.6 |
| C10H20 | Pentyl cyclopentane | -45.2 | -50.7 | -52.3 |
| C10H22 | 2,2,5,5-Tetramethylhexane | -68 | -11.8 | -45.6 |
| C10H22 | 3,3,4,4-Tetramethylhexane | -63.5 | -30.7 | -49.4 |
| C10H22 | n-Decane | -59.7 | -58.1 | -58.5 |
| C11H16 | Pentamethylbenzene | -16.1 | -2.5 | -13.1 |
| C11H22 | 1,1,4-Trimethylcycloheptane | -50.3 | -26.5 | -50.8 |
| C11H22 | Hexyl cyclopentane | -50.1 | -55.5 | -57.8 |
| C11H22 | Pentyl cyclohexane | -55.9 | -53.5 | -58.1 |
| C11H24 | Undecane | -64.6 | -62.8 | -64.0 |
| C12H8 | Acenaphthylene | 61.6 | 67.1 | 68.2 |
| C12H8 | Biphenylene | 100.5 | 94.7 | 89.4 |
| C12H10 | Acenaphthene | 37.4 | 33.1 | 35.2 |
| C12H10 | Biphenyl | 43.5 | 46.0 | 46.2 |
| C12H18 | Hexamethylbenzene | -18.5 | 0.1 | -18.9 |
| C12H24 | Hexylcyclohexane | -60.8 | -58.2 | -63.6 |
| C12H26 | n-Duodecane | -69.2 | -67.6 | -69.5 |
| C13H10 | Fluorene | 41.8 | 44.9 | 45.1 |
| C13H28 | Tri-t-butylmethane | -56.2 | 32.4 | -48.7 |
| C13H28 | Tridecane | -74.5 | -72.3 | -75.1 |
| C14H10 | Anthracene | 55.2 | 58.8 | 61.2 |
| C14H10 | Diphenylethyne | 92 | 89.5 | 86.3 |
| C14H10 | Phenanthrene | 49.5 | 55.6 | 54.9 |
| C14H12 | 9,10-Dihydro-phenanthrene | 37.1 | 38.3 | 36.3 |
| C14H12 | 9-Methyl-9H-fluorene | 35.4 | 42.3 | 42.5 |
| C14H12 | Octalene | 131.8 | 106.2 | 106.8 |
| C14H12 | Stilbene | 53.4 | 63.4 | 58.1 |
| C14H14 | 1,2,3,4-Tetrahydrophenanthrene | 22.1 | 21.9 | 20.4 |
| C14H14 | 4,4'-Dimethylbiphenyl | 26.6 | 30.4 | 31.6 |
| C14H14 | Bibenzyl | 32.4 | 37.6 | 33.4 |
| C14H16 | 1,4,5,8-Tetramethynaphthalene | 19.5 | 31.4 | 18.1 |
| C14H18 | 1,2,3,4,5,6,7,8-Octahydro-anthracene | -8.9 | -18.6 | -17.4 |
| C14H20 | Diadamantane | -34.9 | -23.6 | -42.2 |
| C14H24 | 1,3,5,7-Tetramethyladamantane | -68 | -22.8 | -47.4 |
| C14H28 | (E)-3,4-Di-tert-butyl-3-hexene | -40.2 | 19.2 | -42.9 |
| C14H28 | Cyclotetradecane | -57.2 | -53.2 | -80.5 |
| C14H28 | n-Nonylcyclopentane | -64.9 | -69.6 | -74.4 |
| C14H28 | Octylcyclohexane | -70.7 | -67.7 | -74.7 |
| C14H30 | 3,3,4,4-Tetraethylhexane | -63.5 | 6.2 | -64.7 |
| C14H30 | Octamethylhexane | -59.4 | 46.7 | -44.1 |
| C14H30 | Tetradecane | -79.4 | -77.0 | -80.6 |
| C15H12 | 4-Methylphenanthrene | 46.8 | 56.9 | 49.9 |
| C15H22 | 1-Methyldiadamantane | -39.9 | -18.0 | -43.8 |
| C15H22 | 3-Methyladamantane | -37.6 | -22.7 | -45.6 |
| C15H22 | 4-Methyldiadamantane | -43.5 | -23.0 | -45.1 |
| C15H22 | 6-(1,1-dimethylethyl)-2,3-dihydro-1,1-dimethyl-1H-Indene | -24.9 | 0.6 | -14.0 |
| C15H30 | n-Nonylcyclohexane | -75.6 | -72.3 | -80.2 |
| C15H32 | Pentadecane | -84.8 | -81.7 | -86.1 |
| C16H10 | Fluoranthene | 69.8 | 72.7 | 76.9 |
| C16H10 | Pyrene | 53.9 | 60.7 | 65.9 |
| C16H14 | 2,7-Dimethylphenanthrene | 34.2 | 40.0 | 40.3 |
| C16H14 | 4,5,9,10-Tetrahydropyrene | 21.6 | 25.5 | 27.1 |
| C16H14 | 9,10-Dimethylphenanthrene | 40 | 52.9 | 41.9 |
| C16H16 | (2.2)Metaparacyclophane | 52.2 | 62.7 | 52.0 |
| C16H16 | [2.2]Metacyclophane | 40.7 | 58.7 | 47.4 |
| C16H16 | [2.2]Paracyclophane | 58.5 | 66.6 | 57.7 |
| C16H18 | 1,2,3,6,7,8-Hexahydropyrene | 9.7 | 9.3 | 4.4 |
| C16H28 | Tricyclo[8.2.2.2]-hexadecane | -36.4 | -15.8 | -63.7 |
| C16H32 | 1-Hexadecene | -59.4 | -56.4 | -64.6 |
| C16H32 | Decylcyclohexane | -80.5 | -77.1 | -85.7 |
| C16H32 | n-Undecylcyclopentane | -74.7 | -79.1 | -85.4 |
| C17H34 | n-Dodecylcyclopentane | -79.6 | -83.8 | -90.9 |
| C17H34 | Undecylcyclohexane | -85.4 | -81.8 | -91.2 |
| C17H36 | Heptadecane | -94.2 | -91.2 | -97.1 |
| C18H14 | p-Terphenyl | 66.6 | 70.7 | 71.3 |
| C18H18 | 2,5-Diphenyl-1,5-hexadiene | 68 | 72.6 | 69.0 |
| C18H18 | 3,4,5,6-Tetramethylphenanthrene | 38.3 | 49.0 | 37.7 |
| C18H20 | [3.3]Paracyclophane | 30.9 | 37.0 | 26.5 |
| C18H22 | 1,1'-(1,1,2,2-Tetramethyl-1,2-ethanediyl)bis-benzene | 13.7 | 70.3 | 31.2 |
| C18H36 | Dodecylcyclohexane | -90.4 | -86.5 | -96.7 |
| C18H36 | n-Tridecylcyclopentane | -84.6 | -88.5 | -96.5 |
| C18H38 | 1,1,2,2-Tetra-t-butylethane | -59.9 | 70.2 | -63.3 |
| C18H38 | Octadecane | -99.1 | -95.9 | -102.6 |
| C19H20 | 2,6-Diphenyl-1,6-heptadiene | 61.9 | 67.9 | 59.1 |
| C19H38 | n-Tetradecylcyclopentane | -89.5 | -93.2 | -102.0 |
| C19H38 | n-Tridecylcyclohexane | -95.3 | -91.3 | -102.2 |
| C19H40 | Nonadecane | -104 | -100.6 | -108.1 |
| C20H14 | 9,10-Dihydro-9,10[1',2']benzanthracene | 76.9 | 87.3 | 81.7 |
| C20H16 | 3,9-Dimethylbenz[a]anthracene | 45.1 | 59.3 | 60.7 |
| C20H16 | 5,6-Dimethyl chrysene | 62.7 | 76.4 | 61.8 |
| C20H16 | 9,10-Dimethyl-1,2-benzanthracene | 66.3 | 79.8 | 65.0 |
| C20H30 | 1,3,5-Tri-tert-butyl pentalene | 3.4 | 54.7 | 30.0 |
| C20H36 | Tetra-tert-butyltetrahedrane | 6.2 | 80.9 | 31.7 |
| C20H38 | Meso-3,4-dicyclohexyl-2,5-dimethylhexane | -71.6 | -36.0 | -97.3 |
| C20H40 | Tetradecylcyclohexane | -100.2 | -96.0 | -107.7 |
| C20H42 | Eicosane | -108.9 | -105.4 | -113.6 |
| N | Nitrogen, cation | 448.3 | 417.5 | 413.3 |
| N | Nitrogen, atom | 113 | 113.0 | 98.0 |
| NH2 | Amidogen | 45.5 | 36.4 | 51.6 |
| NH3 | Ammonia | -11 | -6.4 | 9.7 |
| NH4 | Ammonium, cation | 155 | 164.6 | 170.4 |
| CN | Cyanide | 104 | 126.3 | 86.4 |
| CNH | Hydrogen cyanide | 32.3 | 35.3 | 35.1 |
| CNH4 | CH2-NH2, cation | 178 | 186.8 | 193.9 |
| CNH4 | CH3-NH. | 37 | 29.3 | 35.1 |
| CNH4 | CH3NH, anion | 30.5 | 23.5 | 49.0 |
| CNH5 | Methylamine | -5.5 | -7.6 | 5.7 |
| C2NH3 | Acetonitrile | 17.7 | 19.2 | 20.0 |
| C2NH3 | Methyl isocyanide | 39.1 | 60.3 | 59.8 |
| C2NH5 | Ethyleneimine (Azirane) | 30.2 | 25.0 | 33.6 |
| C2NH6 | Dimethyl nitrogen, anion | 24.7 | 8.5 | 35.8 |
| C2NH7 | Dimethylamine | -4.4 | -6.7 | 2.2 |
| C2NH7 | Ethylamine | -11.4 | -13.3 | -3.9 |
| C3NH3 | Acrylonitrile | 44.1 | 43.8 | 46.8 |
| C3NH5 | Ethyl cyanide | 12.1 | 13.7 | 15.7 |
| C3NH7 | Cyclopropylamine | 18.4 | 15.3 | 18.4 |
| C3NH9 | Isopropylamine | -20 | -16.4 | -9.3 |
| C3NH9 | n-Propylamine | -16.8 | -18.3 | -9.2 |
| C3NH9 | Trimethylamine | -5.7 | -2.9 | 0.5 |
| C4NH5 | (E)-2-Butenenitrile | 33.6 | 33.8 | 35.6 |
| C4NH5 | (Z)-2-Butenenitrile | 32 | 34.1 | 35.9 |
| C4NH5 | 3-Butenenitrile | 37.7 | 39.2 | 38.2 |
| C4NH5 | Pyrrole | 25.9 | 32.4 | 21.0 |
| C4NH7 | Butanenitrile | 7.5 | 8.9 | 10.1 |
| C4NH7 | Isobutane nitrile | 5.6 | 11.2 | 12.3 |
| C4NH9 | Pyrrolidine | -0.8 | -15.9 | -9.6 |
| C4NH11 | 2-Butylamine | -25.4 | -20.4 | -14.2 |
| C4NH11 | 2-Methyl-1-propylamine | -23.6 | -19.3 | -13.4 |
| C4NH11 | N-Butylamine | -22.7 | -23.1 | -14.7 |
| C4NH11 | t-Butylamine | -28.9 | -15.5 | -12.5 |
| C5NH5 | Pyridine | 34.6 | 28.8 | 30.4 |
| C5NH7 | N-Methyl pyrrole | 24.6 | 32.2 | 21.1 |
| C5NH9 | 1,2,3,6-Tetrahydropyridine | 7.1 | 6.1 | 6.5 |
| C5NH9 | 2-Cyanobutane | 0.6 | 7.8 | 7.0 |
| C5NH9 | Butyl cyanide | 2.7 | 4.1 | 4.7 |
| C5NH9 | t-Butylnitrile | -0.8 | 12.5 | 10.4 |
| C5NH11 | Cyclopentylamine | -13.1 | -22.6 | -15.9 |
| C5NH11 | Piperidine | -11.3 | -18.6 | -16.9 |
| C5NH13 | N-Methyl-n-butylamine | -25.9 | -21.8 | -17.7 |
| C6NH7 | 1-Cyclopentenecarbonitrile | 37.4 | 28.6 | 31.2 |
| C6NH7 | 2-Cyclopentenecarbonitrile | 33.9 | 34.9 | 32.1 |
| C6NH7 | 2-Methyl pyridine | 23.7 | 19.7 | 22.7 |
| C6NH7 | 3-Methyl pyridine | 24.8 | 20.4 | 22.8 |
| C6NH7 | 4-Methyl pyridine | 24.8 | 20.8 | 22.4 |
| C6NH7 | Aniline | 20.8 | 21.7 | 23.5 |
| C6NH9 | 2,5-Dimethyl-1H-pyrrole | 9.5 | 10.1 | 5.2 |
| C6NH9 | Cyclopentanecarbonitrile | 11.7 | 5.4 | 6.3 |
| C6NH13 | 2-Methylpiperidine | -20.2 | -21.6 | -22.1 |
| C6NH13 | Cyclohexamethylenimine | -10.8 | -17.7 | -20.7 |
| C6NH13 | Cyclohexanamine | -25.1 | -25.8 | -21.0 |
| C6NH15 | Di-n-propylamine | -27.8 | -27.3 | -28.2 |
| C6NH15 | Diisopropylamine | -32.6 | -20.3 | -27.1 |
| C6NH15 | Triethylamine | -22.1 | -14.8 | -26.1 |
| C7NH5 | Phenyl cyanide | 51.5 | 51.9 | 55.0 |
| C7NH9 | 1-Cyclohexenecarbonitrile | 24.3 | 21.0 | 23.2 |
| C7NH9 | 2,6-Dimethylpyridine | 13.4 | 10.7 | 15.2 |
| C7NH9 | 2-Cyclohexenecarbonitrile | 26.2 | 26.6 | 24.8 |
| C7NH9 | Benzylamine | 21 | 19.5 | 25.7 |
| C7NH9 | m-Toluidine | 14.6 | 14.1 | 16.3 |
| C7NH9 | N-Methylaniline | 20.1 | 24.2 | 22.2 |
| C7NH9 | o-Toluidine | 12.7 | 16.1 | 17.4 |
| C7NH9 | p-Toluidine | 10 | 13.8 | 16.9 |
| C7NH11 | Cyclohexanecarbonitrile | -0.9 | 1.7 | 0.3 |
| C7NH13 | Hexahydro-1H-pyrrolizine | -0.9 | -20.1 | -22.5 |
| C7NH13 | n-Heptanenitrile | -7.4 | -5.3 | -6.3 |
| C7NH17 | Isopropylbutylamine | -39.4 | -29.0 | -33.2 |
| C8NH11 | 1-Norbornylcyanide | 18 | 24.3 | 15.1 |
| C8NH11 | 1-Norbornylisocyanide | 39.6 | 63.0 | 48.0 |
| C8NH11 | 5-Ethyl-2-methyl-pyridine | 8.3 | 6.4 | 11.0 |
| C8NH11 | N,N-Dimethyl aniline | 24 | 28.8 | 23.5 |
| C8NH11 | N-Ethyl aniline | 13.4 | 19.3 | 13.8 |
| C8NH15 | 3-Azabicyclo[3.2.2]nonane | -10.4 | -13.9 | -20.2 |
| C8NH15 | n-Heptyl cyanide | -12.1 | -10.0 | -11.8 |
| C8NH17 | N-(2-Methylpropylidene)-butylamine | -21.8 | -15.2 | -13.2 |
| C8NH19 | 2-Methyl-N-(2-methylpropyl)-1-propanamine | -43.2 | -28.5 | -37.8 |
| C8NH19 | Di-sec-butylamine | -43.9 | -26.7 | -37.8 |
| C8NH19 | Dibutylamine | -40.9 | -36.7 | -39.2 |
| C8NH19 | N-(2-Methylpropyl)-1-butanamine | -41.8 | -32.9 | -39.0 |
| C8NH19 | n-Octylamine | -41.5 | -42.0 | -36.7 |
| C9NH7 | Isoquinoline | 48.9 | 45.2 | 48.5 |
| C9NH7 | Quinoline | 47.9 | 44.7 | 49.1 |
| C9NH9 | 2,6-Dimethylbenzonitrile | 34.9 | 40.0 | 41.0 |
| C9NH11 | (1a,2a,4a)-Bicyclo[2.2.2]oct-5-ene-2-carbonitrile | 36.2 | 39.7 | 29.6 |
| C9NH11 | (1a,2b,4a)-Bicyclo[2.2.2]oct-5-ene-2-carbonitrile | 36.6 | 40.1 | 29.9 |
| C9NH11 | 1,2,3,4-Tetrahydroquinoline | 19.6 | 14.1 | 13.1 |
| C9NH11 | 5,6,7,8-Tetrahydroquinoline | 17 | 7.0 | 9.7 |
| C9NH13 | N,N-Dimethyl m-toluidine | 17.4 | 26.2 | 16.3 |
| C9NH13 | N,N-Dimethyl p-toluidine | 16.5 | 25.4 | 16.9 |
| C9NH13 | N-Ethyl m-toluidine | 7.3 | 11.7 | 6.6 |
| C9NH17 | cis-3,7a-H-cis-5,8-H-3,5-Dimethylpyrrolizidine | -15.9 | -25.9 | -31.7 |
| C9NH17 | Decahydro trans-quinoline | -27 | -26.9 | -32.4 |
| C9NH19 | 2,2,6,6-Tetramethyl piperidine | -38.2 | -14.0 | -32.0 |
| N2 | Nitrogen | 0 | 8.3 | 34.5 |
| N2H2 | Diazene | 36 | 31.8 | 51.1 |
| N2H4 | Hydrazine | 22.8 | 14.1 | 30.2 |
| CN2H2 | Diazomethane | 71 | 67.2 | 64.1 |
| CN2H2 | N=N-CH2- | 79 | 72.4 | 86.1 |
| CN2H6 | Methylhydrazine | 22.6 | 14.3 | 24.4 |
| C2N2 | Cyanogen | 73.8 | 66.6 | 71.4 |
| C2N2H8 | 1,1-Dimethylhydrazine | 20 | 18.0 | 22.4 |
| C2N2H8 | 1,2-Dimethylhydrazine | 22 | 14.9 | 20.7 |
| C3N2H4 | 1H-Pyrazole | 42.9 | 45.3 | 34.7 |
| C3N2H4 | Imidazole | 31.8 | 33.2 | 25.3 |
| C3N2H10 | 1,2-Propanediamine | -12.8 | -10.7 | 1.4 |
| C4N2 | Dicyanoacetylene | 126.5 | 111.4 | 114.1 |
| C4N2H2 | Fumaronitrile | 81.3 | 74.7 | 79.6 |
| C4N2H4 | 1,3-Diazine | 47 | 34.9 | 39.2 |
| C4N2H4 | Pyrazine | 46.9 | 37.7 | 41.8 |
| C4N2H4 | Pyridazine | 66.5 | 43.5 | 47.2 |
| C4N2H4 | Succinonitrile | 50.1 | 48.8 | 48.2 |
| C4N2H6 | 2-Methyl-1H-imidazole | 21.5 | 21.5 | 17.1 |
| C4N2H8 | (Dimethylamino) acetonitrile | 27.3 | 31.5 | 31.6 |
| C4N2H8 | 1,4,5,6-Tetrahydropyrimidine | 13.2 | 8.9 | 8.5 |
| C4N2H10 | Piperazine | 6 | -2.9 | -1.0 |
| C5N2H6 | 2-Aminopyridine | 28.2 | 25.8 | 29.9 |
| C5N2H6 | 3-Aminopyridine | 34.5 | 29.3 | 32.8 |
| C5N2H6 | 4-Aminopyridine | 31.1 | 28.3 | 31.1 |
| C5N2H6 | Dimethyl propanedinitrile | 47.1 | 52.8 | 51.6 |
| C5N2H8 | 2-Ethyl-1H-imidazole | 16.3 | 16.5 | 12.4 |
| C5N2H10 | Diethylcyanamide | 15.2 | 29.4 | 14.3 |
| C5N2H12 | Butylmethyldiazene | 18.9 | 5.6 | 13.4 |
| C5N2H14 | N,N-Dimethyl-1,3-propanediamine | -8.3 | -8.8 | -9.3 |
| C6N2H4 | 2-Cyanopyridine | 67.1 | 60.3 | 66.8 |
| C6N2H4 | 3-Cyanopyridine | 66.4 | 59.7 | 65.1 |
| C6N2H4 | 4-Cyanopyridine | 67.8 | 60.9 | 65.7 |
| C6N2H8 | 2,3-Dimethyl pyrazine | 30.1 | 20.2 | 25.7 |
| C6N2H8 | Hexanedinitrile | 35.7 | 38.5 | 35.9 |
| C6N2H8 | Phenylhydrazine | 48.5 | 45.2 | 47.7 |
| C6N2H12 | 3(Dimethylamino) propanenitrile | 21.6 | 22.1 | 19.7 |
| C6N2H12 | Tetramethyldiazetine | 35.9 | 33.6 | 30.0 |
| C6N2H12 | Triethylenediamine | 21.6 | 19.3 | 1.0 |
| C6N2H14 | 1,2-Diisopropyldiazene | 8.6 | 3.7 | 9.1 |
| C6N2H14 | Dipropyldiazene | 12.4 | -0.9 | 7.7 |
| C7N2H6 | 1H-Benzimidazole | 43.4 | 45.8 | 43.4 |
| C7N2H6 | 1H-Indazole | 58.1 | 58.0 | 52.9 |
| C7N2H10 | 1-Methyl-1-phenylhydrazine | 50.4 | 24.9 | 14.3 |
| C7N2H10 | t-Butylmalononitrile | 30.3 | 52.5 | 43.1 |
| C7N2H10 | Trimethyl pyrazine | 17.8 | 10.6 | 18.1 |
| C7N2H12 | 1-Piperidineacetonitrile | 19.8 | 19.6 | 12.1 |
| C7N2H14 | 3,3,5,5-Tetramethyl-1-pyrazoline | 9.4 | 5.2 | 2.8 |
| C8N2H4 | m-Dicyanobenzene | 86.7 | 83.8 | 90.3 |
| C8N2H4 | o-Dicyanobenzene | 87.8 | 85.3 | 91.6 |
| C8N2H4 | p-Dicyanobenzene | 85.6 | 83.9 | 90.3 |
| C8N2H6 | Phthalazine | 78.9 | 59.0 | 65.0 |
| C8N2H6 | Quinazoline | 58.1 | 50.3 | 57.6 |
| C8N2H6 | Quinoxaline | 57.4 | 52.7 | 60.9 |
| C8N2H12 | n-Pentylmalonodinitrile | 32.5 | 34.2 | 31.3 |
| C8N2H12 | Tetramethylbutanedinitrile | 24.1 | 56.8 | 39.9 |
| C8N2H12 | Tetramethylpyrazine | 13.1 | 2.7 | 10.5 |
| C8N2H14 | 1,4-Dimethyl-2,3-diaza-bicyclo[2.2.2]oct-2-ene | 22.1 | 13.7 | 8.8 |
| C8N2H16 | 3,4,5,6-Tetrahydro-3,3,6,6-tetramethylpyridazine | 10 | 6.7 | 0.2 |
| C8N2H18 | Di-n-butyldiazene | 2.2 | -10.4 | -3.1 |
| C8N2H18 | Di-tert-butyldiazene | -8.7 | 4.9 | 5.0 |
| C8N2H20 | 1,2-Dibutylhydrazine | -14.2 | -14.4 | -18.6 |
| N3 | Azide radical | 99 | 97.1 | 98.7 |
| N3H | Hydrazoic acid | 70.3 | 73.1 | 77.7 |
| C3N3H3 | 1,3,5-Triazine | 54 | 40.0 | 48.3 |
| C5N3H | Ethylenetricarbonitrile | 124.4 | 110.4 | 118.5 |
| C5N3H3 | 1,1,1-Ethanetricarbonitrile | 101 | 96.2 | 96.4 |
| CN4H2 | 1-H Tetrazole | 76.6 | 53.7 | 54.6 |
| CN4H2 | 2-H-Tetrazole | 80 | 58.0 | 56.2 |
| C6N4 | Tetracyanoethylene | 168.5 | 148.0 | 159.4 |
| C6N4H12 | 1,3,5,7-Tetraazaadamantane | 47.6 | 51.5 | 18.7 |
| C3N6H6 | Melamine | 12.4 | 21.7 | 34.8 |
| C10NH9 | 2-Methyl-quinoline | 38 | 35.8 | 41.3 |
| C10NH9 | 4-Methyl-quinoline | 38.7 | 39.0 | 41.3 |
| C10NH9 | 6-Methyl-quinoline | 38.5 | 37.0 | 41.6 |
| C10NH9 | 8-Methyl-quinoline | 40.1 | 38.1 | 42.7 |
| C10NH11 | 2,4,6-Trimethyl-benzonitrile | 25.4 | 32.5 | 33.4 |
| C10NH11 | 2,4,6-Trimethylphenyl isocyanide | 56.6 | 70.9 | 67.4 |
| C10NH15 | N,N-Diethyl aniline | 14.8 | 26.9 | 7.0 |
| C10NH19 | n-Nonyl cyanide | -21.9 | -19.5 | -22.8 |
| C11NH11 | 2,6-Dimethyl quinoline | 28.9 | 28.1 | 33.9 |
| C11NH11 | 2,7-Dimethyl quinoline | 29.1 | 28.1 | 33.9 |
| C11NH15 | 1-Adamantyl cyanide | -1.8 | 11.4 | -2.1 |
| C11NH15 | 1-Adamantyl isocyanide | 17.5 | 50.0 | 30.4 |
| C11NH17 | 2-Methyl-6-t-butylaniline | -10.7 | 19.7 | 3.1 |
| C11NH21 | n-Undecanenitrile | -27.1 | -24.2 | -28.3 |
| C12NH9 | Carbazole | 50.1 | 53.5 | 51.7 |
| C12NH11 | 2-Biphenylamine | 44.1 | 47.9 | 48.9 |
| C12NH11 | Biphenylamine | 48.2 | 55.7 | 45.8 |
| C12NH23 | 2-n-Butyl-2-methylhexanenitrile | -31.8 | -7.3 | -27.7 |
| C13NH9 | 6,7-Benzoquinoline | 58.2 | 60.6 | 64.5 |
| C13NH9 | a-Benzoquinoline | 59.9 | 59.9 | 65.3 |
| C13NH9 | Acridine | 65.5 | 64.3 | 71.6 |
| C13NH9 | Benzo[f]quinoline | 55.9 | 61.5 | 64.7 |
| C13NH9 | Phenanthridine | 57.5 | 60.6 | 64.5 |
| C13NH11 | N-Methylcarbazole | 47.6 | 56.1 | 52.7 |
| C13NH15 | 1,2,3,4-Tetrahydro-N-methylcarbazole | 22.3 | 22.9 | 20.1 |
| C14NH13 | 9-Ethyl-9H-carbazole | 40.6 | 51.1 | 44.8 |
| C14NH27 | Tetradecanenitrile | -41.8 | -38.4 | -44.8 |
| C9N2H8 | 3-Quinolinamine | 49.8 | 45.2 | 51.7 |
| C9N2H8 | 5-Quinolinamine | 50.3 | 47.2 | 51.8 |
| C9N2H8 | 6-Quinolinamine | 49.3 | 45.2 | 51.3 |
| C9N2H8 | 8-Quinolinamine | 44.8 | 45.3 | 50.5 |
| C9N2H18 | 2-(Diethylamino)-pentanenitrile | -1.1 | 14.0 | -0.6 |
| C10N2H8 | 2,2-Bipyridyl | 69.1 | 58.8 | 66.2 |
| C10N2H8 | 2,4-Bipyridyl | 67.9 | 60.0 | 65.9 |
| C10N2H8 | 4,4'-Bipyridine | 70.1 | 61.1 | 65.0 |
| C10N2H10 | 2,3-Dimethyl quinoxaline | 41.3 | 36.4 | 44.6 |
| C10N2H12 | alpha N,N-dimethylamino phenylacetonitrile | 52.7 | 58.9 | 48.4 |
| C10N2H16 | Ethyl(1,1-dimethylpropyl)malonodinitrile | 14.6 | 56.6 | 33.1 |
| C10N2H16 | Meso-2,3-diethyl-2,3-dimethylsuccinodinitrile | 15.3 | 55.0 | 30.4 |
| C10N2H16 | Methyl(1,1,2-trimethylpropyl)malonodinitrile | 19 | 66.3 | 36.2 |
| C12N2H8 | Phenazine | 80.9 | 71.7 | 84.0 |
| C12N2H8 | Phenazone | 89.9 | 72.6 | 80.7 |
| C12N2H10 | cis-Azobenzene | 107.7 | 83.5 | 86.8 |
| C12N2H12 | 4,4'-Dimethyl-2,2'-bipyridine | 50 | 43.0 | 50.6 |
| C12N2H20 | 1,(1-Piperidinyl) cyclohexanecarbonitrile | 0.8 | 17.6 | -3.2 |
| C13N2H16 | a-Phenyl-1-piperidineacetonitrile | 45.4 | 53.5 | 41.9 |
| C9N3H3 | 1,3,5-Tricyanobenzene | 121.8 | 116.8 | 126.9 |
| C11N3H7 | 1,1,1-Tricyano-2-phenyl ethane | 129.2 | 126.7 | 123.5 |
| C16NH35 | Dioctylamine | -76.5 | -74.5 | -83.3 |
| C18NH15 | Triphenylamine | 78.1 | 93.9 | 81.2 |
| C18N2H12 | 2,2'-Biquinoline | 101.8 | 91.1 | 104.3 |
| C15N3H11 | 2,2',6',2'-Terpyridine | 94.3 | 88.6 | 102.2 |
| O | Oxygen, atom | 59.6 | 59.6 | 40.2 |
| HO | Hydroxyl radical | 9.5 | 0.2 | -1.5 |
| HO | Hydroxide, anion | -33.2 | -5.8 | -14.2 |
| H2O | Water | -57.8 | -61.0 | -48.4 |
| H3O | Hydronium, cation | 138.9 | 134.2 | 144.3 |
| CO | Carbon monoxide | -26.4 | -6.0 | 5.5 |
| CHO | HCO, cation | 199 | 184.8 | 193.7 |
| CHO | HCO | 10.4 | -1.4 | -11.2 |
| CH2O | Formaldehyde | -26 | -32.9 | -26.5 |
| CH3O | CH2OH, cation | 168 | 155.5 | 170.7 |
| CH3O | Methoxy, anion | -36 | -39.8 | -26.5 |
| CH4O | Methanol | -48.1 | -57.4 | -46.0 |
| C2H2O | Ketene | -11.4 | -6.9 | -21.9 |
| C2H4O | Acetaldehyde | -39.7 | -42.3 | -43.2 |
| C2H4O | Ethylene oxide | -12.6 | -15.5 | -15.1 |
| C2H5O | Ethoxy, anion | -47.5 | -45.3 | -39.9 |
| C2H6O | Dimethyl ether | -44 | -51.2 | -42.4 |
| C2H6O | Ethanol | -56.2 | -63.0 | -55.1 |
| C3H6O | Acetone | -52 | -49.5 | -55.8 |
| C3H6O | Propanal | -45.5 | -47.4 | -48.7 |
| C3H6O | Trimethylene oxide | -19.3 | -37.2 | -32.2 |
| C3H8O | Isopropanol | -65.1 | -65.2 | -61.8 |
| C3H8O | Methyl ethyl ether | -51.7 | -56.7 | -50.0 |
| C3H8O | Propanol | -61.2 | -67.6 | -60.9 |
| C4H4O | Acetyl acetylene | 15.6 | 12.3 | -1.0 |
| C4H4O | Furan | -8.3 | -8.7 | -13.2 |
| C4H6O | 2,3-Dihydrofuran | -17.3 | -29.9 | -30.8 |
| C4H6O | Crotonaldehyde | -24 | -27.9 | -28.2 |
| C4H6O | Divinyl ether | -3.3 | -2.0 | -6.6 |
| C4H8O | Butanal | -48.9 | -52.9 | -54.1 |
| C4H8O | Isobutanal | -51.6 | -50.6 | -51.4 |
| C4H8O | Methyl ethyl ketone | -57.1 | -54.1 | -60.8 |
| C4H8O | Tetrahydrofuran | -44 | -59.3 | -53.4 |
| C4H10O | Diethyl ether | -60.3 | -62.0 | -58.5 |
| C4H10O | t-Butanol | -74.7 | -64.4 | -63.2 |
| C5H8O | 2,3-Dihydro-5-methyl-furan | -31.1 | -39.9 | -37.5 |
| C5H8O | 2-Ethylacrolein | -31.4 | -30.7 | -29.9 |
| C5H8O | 3,4-Dihydro-2H-pyran | -27 | -39.4 | -38.5 |
| C5H8O | 3-Penten-2-one | -32.6 | -34.9 | -40.6 |
| C5H8O | Cyclopentanone | -46 | -57.1 | -63.2 |
| C5H10O | Diethyl ketone | -61.6 | -59.5 | -64.5 |
| C5H10O | Tetrahydropyran | -53.4 | -62.1 | -58.7 |
| C5H12O | t-Butyl methyl ether | -67.8 | -54.6 | -56.9 |
| C6H5O | Phenoxy, anion | -40.5 | -42.3 | -54.1 |
| C6H6O | Phenol | -23 | -26.7 | -22.0 |
| C6H10O | 4-Methyl-3-penten-2-one | -42.6 | -40.9 | -47.5 |
| C6H10O | Cyclohexanone | -54 | -60.2 | -67.0 |
| C6H12O | Methyl neopentyl ketone | -76.6 | -50.9 | -64.1 |
| C6H14O | Di-isopropyl ether | -76.3 | -62.5 | -68.2 |
| C7H6O | Benzaldehyde | -8.8 | -9.6 | -9.7 |
| C7H8O | Anisole | -17.3 | -17.7 | -15.0 |
| C7H8O | m-Cresol | -31.9 | -34.2 | -29.4 |
| C7H8O | o-Cresol | -30.7 | -33.3 | -27.7 |
| C7H8O | p-Cresol | -29.9 | -34.6 | -28.9 |
| C7H10O | 2-Methyl-5-hexen-3-yn-2-ol | 11 | 5.3 | 0.7 |
| C7H10O | 2-Norbornanone | -40.8 | -37.4 | -54.5 |
| C7H10O | cis-2,3-Epoxybicyclo[2.2.1]heptane | -12.9 | 2.8 | -14.8 |
| C7H10O | Norbornan-7-one | -32 | -37.7 | -52.1 |
| C7H12O | 1-Methoxy cyclohexene | -38.5 | -49.1 | -45.0 |
| C7H12O | Bicyclo[2.2.1]heptan-7-ol | -52 | -51.2 | -60.8 |
| C7H12O | cis-1,2-Epoxycycloheptane | -36.4 | -35.6 | -41.5 |
| C7H12O | Cycloheptanone | -59.3 | -59.6 | -68.4 |
| C7H14O | 2,4-Dimethyl 3-pentanone | -74.4 | -63.1 | -68.7 |
| C7H14O | 2-Methyl cis-cyclohexanol | -71.9 | -73.8 | -76.8 |
| C7H14O | 3,3-Dimethyl-2-pentanone | -72.6 | -52.3 | -69.1 |
| C7H14O | 4-Heptanone | -71.3 | -67.8 | -74.7 |
| C7H14O | Heptanal | -63.1 | -67.1 | -70.6 |
| C7H14O | t-Butyl ethyl ketone | -75 | -55.4 | -68.3 |
| C7H16O | n-Heptanol | -81.2 | -86.7 | -82.3 |
| C7H16O | t-Butyl isopropyl ether | -85.5 | -60.5 | -69.4 |
| C8H6O | Benzofuran | 3.3 | 3.8 | 4.4 |
| C8H8O | 1,3-Dihydro isobenzofuran | -7.2 | -20.4 | -15.7 |
| C8H8O | 1-Phenylethenol | -11 | -9.5 | -4.9 |
| C8H8O | 2,3-Dihydro-benzofuran | -11.1 | -22.1 | -18.2 |
| C8H8O | Acetophenone | -20.7 | -17.1 | -21.7 |
| C8H10O | 2,3-Dimethyl phenol | -37.6 | -36.0 | -36.0 |
| C8H10O | 2,4-Dimethyl phenol | -39 | -40.2 | -35.9 |
| C8H10O | 2,5-Dimethyl phenol | -38.7 | -40.1 | -36.3 |
| C8H10O | 2,6-Dimethyl phenol | -38.7 | -38.5 | -34.7 |
| C8H10O | 2-Ethyl phenol | -34.7 | -35.2 | -32.3 |
| C8H10O | 3,4-Dimethyl phenol | -37.4 | -39.5 | -36.3 |
| C8H10O | 3,5-Dimethyl phenol | -38.6 | -41.6 | -36.6 |
| C8H10O | 3-Ethyl phenol | -34.9 | -39.1 | -33.6 |
| C8H10O | 4-Ethyl phenol | -34.5 | -39.4 | -33.3 |
| C8H10O | 2-phenylethanol | -22.6 | -34.0 | -30.6 |
| C8H10O | Ethoxybenzene | -24.3 | -23.0 | -22.5 |
| C8H10O | Phenetole | -26.3 | -22.6 | -22.5 |
| C8H12O | 1-Methylnorcamphor | -48.9 | -40.9 | -57.7 |
| C8H12O | Bicyclo[2.2.2]octanone | -52.2 | -53.4 | -66.2 |
| C8H12O | Bicyclo[3.2.1]octan-2-one | -52 | -52.1 | -65.0 |
| C8H12O | Bicyclo[3.2.1]octan-3-one | -52.9 | -52.1 | -67.7 |
| C8H12O | Bicyclo[3.2.1]octan-8-one | -46.2 | -52.6 | -63.9 |
| C8H12O | cis-Bicyclo[3.3.0]-octan-2-one | -55 | -64.0 | -72.2 |
| C8H12O | trans-Bicyclo[3.3.0]-octan-2-one | -49.4 | -48.2 | -60.6 |
| C8H14O | 3-Oxabicyclo[3,2,2]nonane | -53.2 | -58.0 | -61.1 |
| C8H14O | 6-Methyl-5-hepten-2-one | -60.1 | -48.3 | -61.3 |
| C8H14O | 8-Oxatricyclo[3,2,1,0(1,5)]octane | 6.4 | 50.3 | 22.7 |
| C8H14O | Bicyclo(2.2.2)octan-2-ol | -68 | -66.0 | -72.6 |
| C8H14O | cis-1,2-Epoxycyclooctane | -39.5 | -34.3 | -45.8 |
| C8H14O | Cyclooctanone | -65.1 | -61.5 | -75.0 |
| C8H16O | 2,2,4-Trimethyl-3-pentanone | -80.9 | -56.6 | -72.1 |
| C8H16O | 2-Octanone | -82.5 | -72.9 | -81.6 |
| C8H16O | 3,3,4-Trimethyl pentan-2-one | -78.5 | -48.5 | -71.9 |
| C8H16O | 3-Octanone | -80.9 | -74.0 | -80.9 |
| C8H16O | 4-Octanone | -83.5 | -74.0 | -81.3 |
| C8H16O | Octanal | -69.8 | -71.1 | -76.1 |
| C8H18O | 1-Octanol | -85.3 | -91.4 | -87.8 |
| C8H18O | 1-Tert-butoxybutane | -86.3 | -69.2 | -76.6 |
| C8H18O | 2-(1,1-Dimethylethoxy)-butane | -90.8 | -63.2 | -75.0 |
| C8H18O | Di-n-butyl ether | -79.8 | -81.0 | -80.5 |
| C8H18O | Di-sec-butyl ether | -86.3 | -69.1 | -78.7 |
| C8H18O | tert-Butyl ether | -86.3 | -51.8 | -70.1 |
| C8H18O | tert-Butyl isobutyl ether | -88 | -65.3 | -75.8 |
| C9H10O | 3,4-Dihydro-1H-2-benzopyran | -15.1 | -26.7 | -24.1 |
| C9H10O | 3,4-Dihydro-2H-1-benzopyran | -19.7 | -28.8 | -25.4 |
| C9H10O | Benzyl methyl ketone | -22.6 | -20.8 | -29.8 |
| C9H12O | 2(-1-Methylethyl)-phenol | -41.9 | -37.2 | -34.9 |
| C9H12O | 2,4,6-Trimethyl phenol | -42.3 | -46.2 | -41.4 |
| C9H12O | 3(-1-Methylethyl)-phenol | -41.9 | -39.6 | -37.0 |
| C9H12O | 4-(-1-Methylethyl)-phenol | -41.9 | -40.0 | -36.8 |
| C9H14O | 2,6,6-Trimethyl-2-cyclohexen-1-one | -55.6 | -39.9 | -49.5 |
| C9H14O | Bicycle[3.3.1]nonan-9-one -check -3-one | -57.3 | -58.4 | -70.2 |
| C9H14O | cis Octahydro-2H-inden-2-one | -59.7 | -64.3 | -77.0 |
| C9H14O | trans Octahydro-2H-inden-2-one | -59.6 | -62.1 | -76.0 |
| C9H16O | Cyclononanone | -66.9 | -61.7 | -80.8 |
| C9H18O | 2,6-Dimethyl-4-heptanone | -85.5 | -69.7 | -83.0 |
| C9H18O | 2-Nonanone | -81.5 | -78.3 | -87.1 |
| C9H18O | 3,3,4,4-Tetramethyl-2-pentanone | -83.1 | -37.6 | -72.9 |
| C9H18O | 5-Nonanone | -82.4 | -77.3 | -85.4 |
| C9H18O | Di-tert-butyl ketone | -82.7 | -43.6 | -70.9 |
| C9H20O | 1-Nonanol | -89.8 | -96.1 | -93.3 |
| C9H20O | Amyl-t-butyl ether | -91 | -73.9 | -82.2 |
| C9H20O | Butyl 1,1-dimethylpropyl ether | -91.4 | -71.5 | -81.4 |
| NO | Nitric oxide, cation | 237 | 230.6 | 268.1 |
| NO | Nitric oxide | 21.6 | -0.5 | 17.1 |
| CNO | NCO | 38.1 | 31.6 | 19.0 |
| CNHO | Hydrogen isocyanate | -24.3 | -10.8 | -29.6 |
| CNH3O | Formamide | -44.5 | -40.2 | -43.2 |
| C2NH5O | Acetaldoxime | -5.4 | -18.3 | -9.2 |
| C2NH5O | Acetamide | -57 | -48.2 | -55.2 |
| C3NH3O | Isoxazole | 19.6 | 19.2 | 17.6 |
| C3NH3O | Oxalone (oxazole) | -3.7 | -8.3 | -9.8 |
| C3NH5O | Acrylamine | -31.1 | -24.4 | -28.7 |
| C3NH5O | Methoxyacetonitrile | -8.5 | -15.6 | -11.0 |
| C3NH7O | Dimethylformamide | -45.8 | -37.0 | -43.8 |
| C3NH7O | N-Methyl acetamide | -59.3 | -47.0 | -57.1 |
| C3NH7O | Propanamide | -61.9 | -53.5 | -59.7 |
| C3NH9O | Dimethylaminomethanol | -48.6 | -50.2 | -46.1 |
| C4NH5O | 3-Methyl isoxazole | 8.5 | 7.4 | 9.4 |
| C4NH5O | 5-Methyl isoxazole | 8.1 | 8.1 | 9.6 |
| C4NH7O | 2-Pyrrolidinone | -47.2 | -51.9 | -60.0 |
| C4NH7O | 4,5-Dihydro-2-methyl oxazole | -31.2 | -39.6 | -35.1 |
| C4NH7O | Methacrylamide | -37.7 | -32.0 | -36.3 |
| C4NH9O | 2-Methyl propanamide | -67.5 | -55.2 | -61.7 |
| C4NH9O | Butanamide | -66.7 | -58.2 | -64.5 |
| C4NH9O | Isobutylamide | -67.5 | -55.2 | -61.7 |
| C4NH11O | N,N-Diethyl-hydroxylamine | -29.1 | -28.3 | -29.1 |
| C5NH5O | 2-Pyridinol | -19 | -25.0 | -16.9 |
| C5NH5O | 3-Pyridinol | -10.4 | -18.7 | -11.9 |
| C5NH5O | 4-Pyridinol | -7.2 | -20.0 | -13.3 |
| C5NH5O | Pyridine 1 oxide | 21 | 44.4 | 25.4 |
| C5NH7O | 3,5-Dimethyl isoxazole | -4.3 | -3.6 | 1.5 |
| C5NH9O | 1-Methyl-2-pyrrolidinone | -50.4 | -51.0 | -58.6 |
| C5NH9O | 2-Ethyl-4,5-dihydro-oxazole | -35.6 | -44.1 | -39.1 |
| C5NH9O | N,N-Dimethylamino-2-propen-3-al | -24.9 | -14.6 | -22.2 |
| C5NH11O | 1-(Dimethylamino)-2-propanone | -43 | -37.3 | -44.8 |
| C5NH11O | 2,2-Dimethyl-propanamide | -74.8 | -50.1 | -63.0 |
| C5NH11O | N,N-Dimethyl propanamide | -59.8 | -45.6 | -57.9 |
| C6NH7O | 2-Hydroxy-6-methylpyridine | -28.8 | -33.9 | -24.7 |
| C6NH7O | 3-Hydroxy-2-methylpyridine | -20.2 | -26.6 | -18.3 |
| C6NH7O | 3-Hydroxy-6-methylpyridine | -16.7 | -27.8 | -19.1 |
| C6NH7O | 4-Hydroxy-2-methylpyridine | -17.1 | -28.9 | -21.1 |
| C6NH7O | 6-Methyl-2(1H)-pyridinone | -28.8 | -23.0 | -34.2 |
| C6NH7O | m-Amino phenol | -23.6 | -26.4 | -20.1 |
| C6NH7O | o-Amino phenol | -25 | -26.2 | -17.8 |
| C6NH7O | p-Amino phenol | -21.6 | -25.9 | -18.2 |
| C6NH9O | Trimethyl isoxazole | -4.8 | -13.3 | -5.2 |
| C6NH11O | Caprolactam | -57.3 | -52.9 | -65.9 |
| C6NH11O | Cyclohexanone oxime | -17.9 | -35.3 | -29.2 |
| C6NH13O | N,N-Diethyl acetamide | -68.6 | -48.9 | -70.4 |
| C6NH13O | N,N-Dimethylbutyramine | -64.7 | -50.1 | -63.6 |
| C7NH5O | Benzoxazole | 10.8 | 5.2 | 9.2 |
| C7NH5O | Isocyanatobenzene | -3.5 | 14.0 | -4.1 |
| C7NH7O | Benzamide | -24.1 | -16.2 | -20.4 |
| C7NH11O | N,N-Dimethylamino-2,4-pentadiene-5-al | -7 | -0.1 | -8.1 |
| C7NH13O | 2-Methoxy-3,3-dimethylbutanenitrile | -39.4 | -18.9 | -22.2 |
| C7NH15O | N,N-Diethylaminoacetone | -55.8 | -44.0 | -64.1 |
| C7NH15O | N,N-Dimethyl-tert-butylcarboxamide | -68.4 | -41.3 | -61.0 |
| C8NH5O | alpha-oxo Benzeneacetonitrile | 28.1 | 25.4 | 27.6 |
| C8NH9O | 1,3-dimethyl-2-nitroso-benzene | 33.4 | 16.8 | 22.7 |
| C8NH9O | N-methyl-N-phenyl formamide | -18.1 | -6.7 | -18.2 |
| C8NH17O | Octanone-1-oxime | -35.7 | -46.5 | -41.7 |
| C8NH17O | Octanone-2-oxime | -43.2 | -47.5 | -45.5 |
| C8NH17O | Octanone-3-oxime | -41.3 | -49.0 | -43.5 |
| C8NH17O | Octanone-4-oxime | -43.3 | -48.3 | -44.6 |
| N2O | Nitrous oxide | 19.6 | 31.0 | 26.2 |
| CN2H4O | Urea | -58.7 | -44.8 | -52.0 |
| C2N2H6O | N-Methyl urea | -56.3 | -43.9 | -52.0 |
| C4N2H6O | Dimethyl furazan | 25.6 | 20.9 | 29.1 |
| C4N2H10O | Isopropylurea | -69.3 | -51.3 | -66.4 |
| C5N2H8O | 5-Amino-3,4-dimethylisoxazole | 1.2 | -4.7 | 4.6 |
| C5N2H12O | (1-Methylpropyl) urea | -73.4 | -55.3 | -71.3 |
| C5N2H12O | N,N-diethylurea | -65.1 | -45.5 | -66.9 |
| C5N2H12O | Tetramethylurea | -49.1 | -30.7 | -45.2 |
| C4N3H5O | 4-Amino-2(1H)-pyrimidinone | -14.2 | -22.9 | -9.9 |
| O2 | Oxygen (Singlet) | 22 | 12.1 | 19.7 |
| O2 | Oxygen (Triplet) | 0 | -16.1 | -8.2 |
| H2O2 | Hydrogen peroxide | -32.5 | -38.2 | -43.4 |
| CO2 | Carbon dioxide | -94.1 | -75.1 | -101.9 |
| CHO2 | Formate, anion | -106.6 | -101.6 | -117.6 |
| CH2O2 | Formic acid | -90.5 | -92.6 | -94.6 |
| C2H2O2 | trans Glyoxal | -50.7 | -61.4 | -60.8 |
| C2H3O2 | Acetate, anion | -122.5 | -110.0 | -131.0 |
| C2H4O2 | Acetic acid | -103.3 | -101.1 | -104.5 |
| C2H4O2 | Methyl formate | -83.6 | -85.5 | -86.7 |
| C2H6O2 | Dimethyl peroxide | -30.1 | -28.3 | -35.5 |
| C2H6O2 | Ethylene glycol | -93.9 | -106.0 | -95.6 |
| C3O2 | Carbon suboxide | -22.4 | -23.6 | -41.5 |
| C3H4O2 | 2-Oxo-propanal | -64.8 | -70.9 | -74.3 |
| C3H4O2 | 2-Propenoic acid | -79 | -76.2 | -76.5 |
| C3H4O2 | beta-Propiolactone | -67.6 | -68.9 | -77.2 |
| C3H6O2 | 1,3-Dioxalane | -72.1 | -93.0 | -85.5 |
| C3H6O2 | Ethyl formate | -95.2 | -90.2 | -94.0 |
| C3H6O2 | Methyl acetate | -97.9 | -93.7 | -96.1 |
| C3H6O2 | Propionic acid | -108.4 | -105.7 | -108.5 |
| C3H8O2 | 1,3-Propanediol | -97.6 | -110.2 | -100.7 |
| C3H8O2 | 2-Methoxyethanol | -90.1 | -99.8 | -91.2 |
| C3H8O2 | Dimethoxymethane | -83.2 | -94.4 | -85.3 |
| C3H8O2 | Propylene glycol | -102.7 | -107.6 | -102.1 |
| C4H6O2 | 2-Butenoic acid | -88.1 | -85.9 | -87.8 |
| C4H6O2 | 2-Methyl-2-propenic acid | -87.8 | -83.7 | -84.0 |
| C4H6O2 | Diacetyl | -78.2 | -78.9 | -87.5 |
| C4H6O2 | gamma Butyrolactone | -87 | -94.0 | -99.6 |
| C4H6O2 | Methyl 2-propenoate | -79.6 | -68.6 | -68.5 |
| C4H8O2 | 1,1 Dimethoxy ethene | -67.1 | -70.9 | -62.7 |
| C4H8O2 | 1,3 Dioxan | -80.9 | -94.3 | -89.8 |
| C4H8O2 | 1,4-Dioxane | -75.5 | -89.3 | -84.9 |
| C4H8O2 | Ethyl acetate | -106.5 | -99.0 | -103.4 |
| C4H10O2 | 1,2-Dimethoxyethane | -81.9 | -93.4 | -84.8 |
| C4H10O2 | 1,4 Butandiol | -101.8 | -115.3 | -105.6 |
| C4H10O2 | Diethyl peroxide | -46.1 | -38.5 | -49.6 |
| C4H10O2 | Dimethyl acetal | -93.1 | -95.2 | -87.8 |
| C5H8O2 | Acetylacetone | -91.9 | -84.4 | -98.9 |
| C5H10O2 | Ethyl propionate | -111.5 | -103.5 | -107.2 |
| C5H10O2 | Isopropyl acetate | -115.1 | -100.6 | -107.7 |
| C5H12O2 | 1,5 Pentandiol | -105.6 | -119.9 | -111.3 |
| C6H4O2 | p-Benzoquinone | -29.3 | -33.0 | -37.4 |
| C6H6O2 | 1,2-Benzenediol | -65.7 | -72.7 | -61.2 |
| C6H6O2 | Hydroquinone | -66.2 | -74.0 | -63.8 |
| C6H6O2 | Resorcinol | -68 | -75.0 | -65.4 |
| C6H8O2 | 1,3-Cyclohexanedione | -80.2 | -83.7 | -100.9 |
| C6H8O2 | 1,4-Cyclohexanedione | -79.5 | -84.7 | -99.6 |
| C6H10O2 | 2,4-Hexanedione | -105.1 | -89.0 | -102.9 |
| C6H10O2 | 2-Oxepanone | -94.7 | -94.4 | -107.2 |
| C6H10O2 | 3-Methyl-2,4-pentandione | -102.5 | -84.5 | -101.2 |
| C6H10O2 | Ethyl-(E)-2-butenoate | -89.8 | -83.7 | -86.5 |
| C6H12O2 | 1,1-Dimethoxy-2-butene | -72.4 | -79.7 | -72.3 |
| C6H12O2 | 4-Hydroxy-4-methylpentan-2-one | -116.2 | -96.4 | -107.9 |
| C6H12O2 | 5,5-Dimethyl-1,3-dioxane | -100.7 | -93.6 | -96.2 |
| C6H12O2 | cis-2,4-Dimethyl-1,3-dioxane | -102.3 | -100.7 | -97.0 |
| C6H12O2 | Ethyl butanoate | -115.9 | -108.1 | -112.8 |
| C6H12O2 | Hexanoic acid | -122.5 | -119.7 | -124.9 |
| C6H12O2 | Methyl 2-methylbutanoate | -117.7 | -103.9 | -108.2 |
| C6H12O2 | Methyl 2,2-dimethyl-propanoate | -118.2 | -96.2 | -102.4 |
| C6H12O2 | Methyl 3-methylbutanoate | -119 | -103.9 | -109.7 |
| C6H12O2 | Methyl pentanoate | -112.7 | -107.5 | -110.9 |
| C6H12O2 | t-Butyl acetate | -123.4 | -96.5 | -109.2 |
| C6H12O2 | trans 4,5-Dimethyl-1,3-dioxane | -98.2 | -97.9 | -98.3 |
| C6H14O2 | 1,1-Diethoxy ethane | -108.4 | -106.5 | -104.5 |
| C6H14O2 | 1,1-Dimethoxy-butane | -101.7 | -104.0 | -98.3 |
| C6H14O2 | 1,2-Diethoxy ethane | -97.6 | -104.1 | -102.5 |
| C6H14O2 | 1,6-Hexanediol | -109.8 | -124.7 | -116.9 |
| C6H14O2 | 2,3-Dimethyl-2,3-butanediol | -129.2 | -99.2 | -108.2 |
| C7H6O2 | 3-(2-Furanyl)-2-propenal | -25.3 | -27.8 | -28.8 |
| C7H6O2 | Benzoic acid | -70.1 | -67.7 | -67.9 |
| C7H6O2 | Phenyl formate | -51.6 | -50.0 | -52.3 |
| C7H6O2 | Tropolone | -37.2 | -40.1 | -36.4 |
| C7H8O2 | 3-Methyl-1,2-benzenediol | -71.5 | -80.3 | -69.8 |
| C7H8O2 | 4-Methyl 1,2-Benzenediol | -71.3 | -80.3 | -68.2 |
| C7H10O2 | Ethyl 2-methylene-3-butenoate | -69.2 | -57.6 | -58.4 |
| C7H10O2 | Ethyl 2-pentynoate | -59.8 | -56.2 | -62.9 |
| C7H10O2 | Ethyl 3-pentynoate | -56.8 | -58.2 | -69.7 |
| C7H10O2 | Ethyl 4-pentynoate | -55.7 | -47.0 | -65.4 |
| C7H12O2 | 3,5-Heptanedione | -104.9 | -93.6 | -106.9 |
| C7H12O2 | 3-Ethyl-2,4-pentanedione | -105.1 | -87.8 | -106.9 |
| C7H12O2 | 5-Methyl-2,4-hexanedione | -108.2 | -91.0 | -105.5 |
| C7H12O2 | Butyl 2-propenoate | -89.7 | -83.3 | -86.2 |
| C7H12O2 | Ethyl (Z)-2-pentenoate | -94.3 | -88.4 | -91.4 |
| C7H12O2 | Ethyl (Z)-3-pentenoate | -92.6 | -89.5 | -94.7 |
| C7H12O2 | Ethyl 4-pentenoate | -92.1 | -82.7 | -90.9 |
| C7H12O2 | Ethyl trans-2-pentenoate | -94.2 | -88.4 | -91.4 |
| C7H12O2 | Heptanolactone | -98.3 | -92.4 | -108.0 |
| C7H12O2 | Isopropyl 2-butenoate | -98.2 | -85.3 | -90.9 |
| C7H12O2 | Propyl (E)-2-butenoate | -94.4 | -88.5 | -91.6 |
| C7H14O2 | (2a,4a,6b)-2,4,6-Trimethyl-1,3-dioxane | -106.8 | -103.4 | -100.9 |
| C7H14O2 | 1,1-Dimethoxycyclopentane | -95 | -97.3 | -96.3 |
| C7H14O2 | 1,1-Dimethylpropyl acetate | -128.8 | -97.3 | -114.1 |
| C7H14O2 | Ethyl 2-methylbutanoate | -123.4 | -108.5 | -115.4 |
| C7H14O2 | Ethyl 3-methylbutanoate | -126 | -108.4 | -116.9 |
| C7H14O2 | Ethyl pentanoate | -121.2 | -112.8 | -118.1 |
| C7H14O2 | Methyl 3,3-dimethylbutanoate | -122.3 | -99.6 | -110.4 |
| C7H14O2 | Methyl hexanoate | -118 | -112.2 | -116.4 |
| C7H16O2 | 1,3-Diethoxypropane | -104.3 | -108.2 | -107.9 |
| C7H16O2 | 1,7-Heptanediol | -114.1 | -129.4 | -122.5 |
| C8H8O2 | m-Methylbenzoic acid | -78.4 | -75.3 | -75.2 |
| C8H8O2 | Methyl benzoate | -66.8 | -60.3 | -59.5 |
| C8H8O2 | o-Methylbenzoic acid | -76.6 | -73.3 | -74.1 |
| C8H8O2 | p-Methylbenzoic acid | -79 | -75.4 | -75.8 |
| NO2 | Nitrogen dioxide, cation | 233 | 240.7 | 238.7 |
| NO2 | Nitrogen dioxide | 7.9 | -6.0 | -14.4 |
| NHO2 | Nitrous acid, trans | -18.8 | -40.7 | -27.3 |
| CNH3O2 | Methyl nitrite | -15.8 | -36.7 | -23.1 |
| CNH3O2 | Nitromethane | -17.9 | 3.3 | -14.5 |
| C2NH5O2 | Ethyl nitrite | -25.9 | -42.0 | -30.9 |
| C2NH5O2 | Glycine | -93.7 | -95.7 | -92.9 |
| C2NH5O2 | Methyl carbamate | -101.6 | -89.0 | -94.3 |
| C2NH5O2 | Nitroethane | -23.5 | -2.1 | -21.2 |
| C3NH7O2 | Alanine | -99.1 | -98.7 | -96.9 |
| C3NH7O2 | beta-Alanine | -101 | -99.0 | -97.7 |
| C3NH7O2 | Isopropylnitrite | -31.9 | -44.7 | -35.7 |
| C3NH7O2 | N-Methyl glycine | -87.8 | -94.0 | -95.9 |
| C3NH7O2 | Propyl nitrite | -28.4 | -47.0 | -36.2 |
| C3NH7O2 | Urethane | -106.7 | -94.3 | -101.7 |
| C4NH5O2 | Methyl cyanoacetate | -58.2 | -57.1 | -60.8 |
| C4NH5O2 | Succinimide | -89.8 | -87.7 | -105.9 |
| C4NH9O2 | 2-Nitrobutane | -39.1 | -10.2 | -32.4 |
| C4NH9O2 | 2-Nitroisobutane | -42.2 | -3.7 | -28.7 |
| C4NH9O2 | 4-Aminobutanoic acid | -105 | -104.3 | -103.0 |
| C4NH9O2 | Isobutyl nitrite | -36.1 | -47.9 | -40.5 |
| C4NH9O2 | n-Butyl nitrite | -34.8 | -51.6 | -41.4 |
| C4NH9O2 | Sec-butyl nitrite | -36.5 | -48.2 | -40.8 |
| C4NH9O2 | t-Butyl nitrite | -41 | -42.6 | -37.9 |
| C4NH11O2 | Diethanolamine | -94.9 | -103.4 | -96.8 |
| C5NH5O2 | N-Methylmaleimide | -61.2 | -51.1 | -64.7 |
| C5NH7O2 | Glutarimide | -94.1 | -92.0 | -111.0 |
| C5NH7O2 | N-Methylsuccinimide | -93.1 | -86.1 | -103.5 |
| C5NH9O2 | Proline | -87.5 | -101.0 | -100.8 |
| C5NH11O2 | 5-Aminovaleric acid | -110 | -109.1 | -108.5 |
| C5NH11O2 | N,N-Dimethylglycine methyl ester | -88.5 | -81.9 | -84.7 |
| C5NH11O2 | tert-Pentyl nitrite | -45.8 | -44.2 | -42.7 |
| C5NH11O2 | Valine | -108.2 | -101.1 | -103.5 |
| C5NH13O2 | 1,1-Dimethoxy-trimethylamine | -85 | -84.4 | -76.5 |
| C6NH5O2 | Niacin | -52.9 | -60.1 | -58.4 |
| C6NH5O2 | Nitrobenzene | 15.4 | 35.8 | 19.3 |
| C6NH9O2 | Ethyl 2-cyanopropionate | -74.6 | -64.6 | -70.4 |
| C6NH13O2 | Ethyl N,N-dimethylglycinate | -97.3 | -86.4 | -91.9 |
| C6NH13O2 | Hexanoic acid, 6-amino- | -115.3 | -112.9 | -114.1 |
| C6NH13O2 | Isoleucine | -110 | -103.2 | -109.0 |
| C6NH13O2 | Leucine | -113.1 | -106.7 | -111.7 |
| C6NH13O2 | Methyl N,N-dimethylalaninate | -94.3 | -81.6 | -89.4 |
| C6NH15O2 | N,N-Dimethylacetamide dimethyl acetal | -92.5 | -78.4 | -79.8 |
| C7NH7O2 | m-Aminobenzoic acid | -69.2 | -67.0 | -65.2 |
| C7NH7O2 | o-Aminobenzoic acid | -70.8 | -66.3 | -66.9 |
| C7NH7O2 | p-Aminobenzoic acid | -70.2 | -67.7 | -67.2 |
| C7NH7O2 | p-Nitrotoluene | 7.4 | 28.1 | 11.0 |
| C7NH7O2 | Phenylnitromethane | 7.3 | 29.0 | 10.6 |
| C7NH15O2 | Methyl N,N-,a,a-tetramethylglycinate | -108.1 | -73.0 | -89.1 |
| C2N2H4O2 | Oxalamide | -92.5 | -76.3 | -87.2 |
| C2N2H6O2 | N-Nitrodimethylamine | -3.2 | 22.3 | -4.8 |
| C3N2H6O2 | Acetyl-urea | -105.6 | -82.4 | -104.4 |
| C3N2H6O2 | Propanediamide | -99.5 | -82.2 | -98.7 |
| C4N2H4O2 | Pyrazine-1,4-dioxide | 36.3 | 71.7 | 34.8 |
| C4N2H4O2 | Uracil | -72.4 | -64.7 | -87.0 |
| C5N2H6O2 | Thymine | -78.6 | -72.5 | -92.8 |
| C6N2H6O2 | m-Nitroaniline | 14.9 | 36.5 | 21.6 |
| C6N2H6O2 | p-Nitroaniline | 13.2 | 35.6 | 18.9 |
| C6N2H14O2 | Lysine | -102.5 | -104.4 | -101.8 |
| C2N3H5O2 | Imidodicarbonic diamide | -104.4 | -77.8 | -104.1 |
| O3 | Ozone | 34.1 | 48.5 | 45.7 |
| C3H6O3 | 1,3,5-Trioxane | -111.3 | -130.2 | -123.1 |
| C3H6O3 | Methyl hydroxyacetate | -133.1 | -137.0 | -133.7 |
| C3H8O3 | Glycerol | -138.1 | -150.8 | -141.4 |
| C4H2O3 | Malaic anhydride | -95.2 | -88.5 | -100.9 |
| C4H6O3 | Acetic anhydride | -137.1 | -132.6 | -145.0 |
| C4H10O3 | Trimethoxymethane | -127.1 | -135.9 | -128.3 |
| C6H14O3 | 2,5,8-Trioxanonane | -124.6 | -135.4 | -128.2 |
| C7H6O3 | m-Salicylic acid | -112.1 | -115.1 | -110.5 |
| C7H6O3 | o-Salicylic acid | -118.5 | -114.1 | -112.7 |
| C7H6O3 | p-Salicylic acid | -117.7 | -116.0 | -111.6 |
| C7H14O3 | 2,3-Butanediol, 2,3-dimethyl-, monoformate | -161.7 | -121.8 | -141.6 |
| NO3 | Nitrate anion | -74.7 | -66.9 | -98.2 |
| NHO3 | Nitric acid | -32.1 | -17.4 | -37.0 |
| CNH3O3 | Methyl nitrate | -29.1 | -12.3 | -33.1 |
| C2NH3O3 | Oxamic acid | -132 | -127.3 | -132.8 |
| C2NH5O3 | Ethyl nitrate | -36.8 | -17.8 | -40.9 |
| C3NH7O3 | Serine | -133.5 | -141.3 | -135.9 |
| C4NH3O3 | 2-Nitrofuran | -6.9 | 7.9 | -9.8 |
| C4NH9O3 | Threonine | -140.5 | -142.5 | -138.5 |
| C6NH5O3 | m-Nitrophenol | -25.2 | -11.4 | -22.2 |
| C6NH5O3 | o-Nitrophenol | -30.8 | -10.2 | -21.5 |
| C6NH5O3 | p-Nitrophenol | -27.4 | -12.5 | -24.4 |
| N2O3 | Dinitrogen trioxide | 19.8 | 12.5 | 11.7 |
| C2H2O4 | Oxalic acid | -175 | -170.5 | -174.3 |
| C2H6O4 | Dioxybismethanol | -124.5 | -129.1 | -133.3 |
| C4H4O4 | 1,4-Dioxan-2,5-dione | -146.3 | -154.3 | -164.9 |
| C4H6O4 | Dimethyl oxalate | -169.5 | -164.0 | -164.4 |
| C4H8O4 | 1,3,5,7-Tetroxane | -148.2 | -168.3 | -166.1 |
| C5H8O4 | Dimethyl malonate | -176.4 | -170.4 | -177.2 |
| C5H8O4 | Ethylmalonic acid | -203.1 | -190.2 | -199.6 |
| C5H8O4 | Methylene diacetate | -186.6 | -178.2 | -190.4 |
| C5H12O4 | Tetramethoxymethane | -173.8 | -181.1 | -161.4 |
| C6H10O4 | 1,1-Diacetoxyethane | -194.2 | -180.5 | -193.0 |
| C6H10O4 | Dimethyl methylmalonate | -183.7 | -172.1 | -177.9 |
| C4NH7O4 | Aspartic acid | -185.9 | -182.7 | -189.1 |
| N2O4 | Dinitrogen tetroxide | 2.2 | 30.2 | 2.5 |
| CN2H2O4 | Dinitromethane | -13.3 | 27.9 | -8.6 |
| CN3H3O4 | Methyldinitramine | 10.3 | 53.2 | 8.8 |
| C3H4O5 | Tartronic acid | -227.5 | -226.4 | -228.9 |
| C5H10O5 | 1,3,5,7,9-pentaoxecane | -186.4 | -208.8 | -206.1 |
| N2O5 | Dinitrogen pentoxide | 2.7 | 34.4 | -8.8 |
| C10H10O | 4-Phenyl-3-buten-2-one | -11.5 | -2.8 | -8.8 |
| C10H14O | 2-Adamantone | -55.1 | -53.7 | -70.4 |
| C10H14O | 2-Isopropyl-4-methylphenol | -47.4 | -42.5 | -40.2 |
| C10H14O | 2-Isopropyl-5-methylphenol | -44.1 | -44.8 | -42.2 |
| C10H14O | 2-Isopropyl-6-methylphenol | -45.4 | -40.5 | -40.3 |
| C10H14O | 2-Methyl-5-isopropylphenol | -46.4 | -45.5 | -44.0 |
| C10H14O | 3-Isopropyl-2-methylphenol | -43.6 | -39.1 | -43.7 |
| C10H14O | 3-Methyl-2-isopropylphenol | -41 | -38.8 | -40.4 |
| C10H14O | 3-Methyl-5-isopropylphenol | -50.2 | -46.9 | -44.2 |
| C10H14O | 4-Isopropyl-2-methylphenol | -49.4 | -45.6 | -43.8 |
| C10H14O | 4-Isopropyl-3-methylphenol | -44 | -40.3 | -45.1 |
| C10H14O | 4-Methyl-3-isopropylphenol | -44.2 | -40.3 | -44.8 |
| C10H14O | m-tert-Butylphenol | -44.3 | -32.1 | -38.7 |
| C10H14O | o-sec-Butylphenol | -45.8 | -38.1 | -38.9 |
| C10H14O | o-tert-Butylphenol | -47.6 | -26.7 | -33.7 |
| C10H14O | p-sec-Butylphenol | -45.6 | -43.4 | -42.5 |
| C10H14O | p-tert-Butyl phenol | -44.5 | -32.6 | -38.4 |
| C10H16O | 1-Adamantol | -74.3 | -65.9 | -75.8 |
| C10H16O | 2-Adamantol | -71.5 | -65.2 | -78.0 |
| C10H16O | Camphor | -63.9 | -33.9 | -61.6 |
| C10H16O | Octahydro-3a-methyl-cis-2H-inden-2-one | -68.6 | -60.7 | -80.0 |
| C10H16O | Octahydro-3a-methyl-trans-2H-inden-2-one | -65.8 | -54.2 | -75.9 |
| C10H18O | Beta-caran-3-ol | -61.9 | -46.8 | -62.1 |
| C10H18O | Cyclodecanone | -72.9 | -63.3 | -85.1 |
| C10H20O | 2,2,5,5-Tetramethyl-3-hexanone | -94.1 | -54.6 | -78.1 |
| C10H22O | Decanol | -94.5 | -100.8 | -98.8 |
| C10H22O | Dipentyl ether | -93.1 | -89.4 | -88.0 |
| C11H14O | 2,4,5-Trimethyl-acetophenone | -45.2 | -35.4 | -43.7 |
| C11H14O | 2,4,6-Trimethyl-acetophenone | -49 | -34.7 | -42.3 |
| C11H16O | 2-tert-Butyl-p-cresol | -49.5 | -34.3 | -40.4 |
| C11H16O | 3-Methyl-2-phenylbutane-2-ol | -50.5 | -25.6 | -37.1 |
| C11H20O | Cycloundecanone | -77 | -66.4 | -93.1 |
| C11H22O | 2,2,6,6-Tetramethyl-4-heptanone | -100.7 | -60.4 | -88.8 |
| C11H22O | Dipentyl ketone | -92.6 | -87.8 | -96.4 |
| C11H24O | Decyl methyl ether | -91.1 | -94.5 | -94.1 |
| C12H8O | Dibenzofuran | 11.3 | 14.4 | 19.8 |
| C12H10O | m-Hydroxybiphenyl | 5.1 | -1.7 | 3.2 |
| C12H10O | o-Hydroxybiphenyl | 4 | -0.7 | 5.7 |
| C12H10O | p-Hydroxybiphenyl | 0 | -2.1 | 3.2 |
| C12H16O | Isobutyl phenyl ketone | -38.4 | -31.9 | -43.0 |
| C12H18O | 2,6-Diisopropylphenol | -60.7 | -44.7 | -48.2 |
| C13H10O | Benzophenone | 11.9 | 16.0 | 14.8 |
| C14H10O | Anthone | 5.6 | 12.3 | 8.5 |
| C9NH7O | 2(1H)-Quinolinone | -6.1 | -3.4 | -11.6 |
| C9NH7O | 3-Phenyl isoxazole | 33.3 | 40.6 | 43.3 |
| C9NH7O | 4-Quinolinol | 5 | -3.1 | 6.5 |
| C9NH7O | 5-Phenyl isoxazole | 38.3 | 41.6 | 44.4 |
| C9NH7O | 8-Quinolinol | 1.6 | -4.6 | 4.5 |
| C9NH7O | a-Cyanoacetophenone | 16.8 | 17.9 | 13.6 |
| C9NH11O | 2,4,6-Trimethylnitrosobenzene | 25.7 | 9.3 | 14.8 |
| C9NH11O | N,N-Dimethyl benzamide | -20.6 | -9.3 | -17.8 |
| C9NH13O | N,N-Dimethyamino-2,4,6-heptatriene-7-al | 5.2 | 13.8 | 5.2 |
| C9NH17O | 2,2,6,6-Tetramethyl-4-piperidinone | -65.4 | -39.7 | -68.0 |
| C10NH9O | 2-Methyl-4-hydroxyquinoline | -5.6 | -11.8 | -1.2 |
| C10NH9O | 2-Methyl-8-quinolinol | -9.4 | -13.6 | -3.4 |
| C10NH9O | 3-Methyl-5-phenyl isoxazole | 24.5 | 29.9 | 36.2 |
| C10NH9O | 4-Methyl-2-hydroxyquinoline | -14.6 | -14.7 | -6.1 |
| C10NH9O | 5-Methyl-3-phenyl isoxazole | 23.6 | 29.6 | 35.3 |
| C10NH9O | beta-Cyanopropiophenone | 7.2 | 12.4 | 6.2 |
| C10NH11O | 2,4,6-Trimethylbenzonitrile, N-oxide | 32.7 | 48.6 | 30.4 |
| C10NH13O | 2-(Dimethylamino)-acetophenone | -11.1 | -4.7 | -9.0 |
| C10NH13O | N,N,4-Trimethyl benzamide | -26.9 | -17.1 | -25.3 |
| C11NH13O | (E)-3-(Methylamino)-1-phenyl-but-2-enone | -12.8 | 2.9 | -8.4 |
| C11NH15O | 1-Propanone, 2-(dimethylamino)-1-phenyl- | -16.7 | -4.9 | -13.8 |
| C11NH15O | 2-Propanamine, 2-methyl-N-(phenylmethylene)-, N-oxide | 7.4 | 50.4 | 21.5 |
| C11NH17O | 1-Adamantanecarboxamide | -76.2 | -52.4 | -77.4 |
| C11NH23O | N,N-Dimethylnonamide | -89.4 | -74.1 | -91.0 |
| C12NH9O | Phenoxazine | 22.5 | 16.8 | 18.9 |
| C12NH17O | 2-(Diethylamino)-1-phenylethanone | -23 | -12.0 | -26.0 |
| C13NH9O | 9,10-Dihydro-9-oxoacridine | 8.3 | 17.7 | 11.8 |
| C13NH11O | Benzenamine, N-(phenylmethylene)-, N-oxide | 62.9 | 86.1 | 68.8 |
| C13NH19O | 1-Propanone, 2-(diethylamino)-1-phenyl- | -29.7 | -11.8 | -32.1 |
| C13NH21O | N,N-Dimethyl-1-adamantylcarboxamide | -68.4 | -43.8 | -74.4 |
| C9H10O2 | 1,3-Dioxolane-2-phenyl | -49.1 | -61.2 | -58.5 |
| C9H10O2 | 2,3-Dimethylbenzoic acid | -82.7 | -77.4 | -81.4 |
| C9H10O2 | 2,4-Dimethylbenzoic acid | -84.9 | -80.9 | -81.7 |
| C9H10O2 | 2,5-Dimethylbenzoic acid | -83.9 | -81.0 | -81.3 |
| C9H10O2 | 2,6-Dimethylbenzoic acid | -81.6 | -78.1 | -80.5 |
| C9H10O2 | 3,4-Dimethylbenzoic acid | -86.6 | -80.6 | -83.0 |
| C9H10O2 | 3,5-Dimethylbenzoic acid | -87.1 | -82.9 | -82.4 |
| C9H10O2 | 3-Ethylbenzoic acid | -82.9 | -80.2 | -79.7 |
| C9H10O2 | 4-Ethylbenzoic acid | -85 | -80.3 | -80.4 |
| C9H10O2 | Methyl 4-methylbenzoate | -74.7 | -65.8 | -67.2 |
| C10H8O2 | 1,2-Naphthalenediol | -47.9 | -54.7 | -45.2 |
| C10H8O2 | 1,3-Naphthalenediol | -50.5 | -56.5 | -45.5 |
| C10H8O2 | 1,4-Naphthalenediol | -47.1 | -53.9 | -42.7 |
| C10H8O2 | 2,3-Naphthalenediol | -46.1 | -55.7 | -43.0 |
| C10H10O2 | 1-Phenyl-1,3-butanedione | -58.3 | -51.9 | -64.4 |
| C10H12O2 | 2,3,4-Trimethylbenzoic acid | -90.2 | -80.9 | -88.1 |
| C10H12O2 | 2,3,5-Trimethylbenzoic acid | -91.4 | -85.0 | -88.4 |
| C10H12O2 | 2,3,6-Trimethylbenzoic acid | -88.7 | -81.9 | -87.5 |
| C10H12O2 | 2,4,5-Trimethylbenzoic acid | -92.3 | -86.1 | -88.8 |
| C10H12O2 | 2,4,6-Trimethylbenzoic acid | -89.4 | -85.6 | -87.9 |
| C10H12O2 | 2-Isopropyl benzoic acid | -85.9 | -77.5 | -81.2 |
| C10H12O2 | 2-Methyl-2-phenyl-1,3-dioxolane | -62.6 | -62.2 | -59.9 |
| C10H12O2 | 3,4,5-Trimethylbenzoic acid | -93.2 | -82.1 | -89.4 |
| C10H12O2 | 3-Isopropyl benzoic acid | -89.8 | -80.7 | -83.3 |
| C10H12O2 | 4-Isopropyl benzoic acid | -91.5 | -80.9 | -83.5 |
| C10H14O2 | 2-Isopropyl-6-methyl-pyrocatechol | -90.6 | -89.7 | -84.8 |
| C10H18O2 | Cyclohexyl butanoate | -130.4 | -119.1 | -128.7 |
| C10H20O2 | Ethyl octanoate | -136.2 | -127.0 | -134.7 |
| C10H22O2 | 1,10-Decanediol | -125 | -143.6 | -139.1 |
| C11H8O2 | 1-Naphthoic acid | -53.3 | -48.3 | -47.4 |
| C11H8O2 | Isonaphthoic acid | -55.6 | -50.5 | -49.5 |
| C11H14O2 | 2,3,4,5-Tetramethylbenzoic acid | -95.3 | -84.4 | -94.7 |
| C11H14O2 | 2,3,4,6-Tetramethylbenzoic acid | -95.2 | -85.4 | -94.1 |
| C11H14O2 | 2,3,5,6-Tetramethylbenzoic acid | -95.6 | -85.8 | -94.4 |
| C11H14O2 | p-tert-Butyl benzoic acid | -95.2 | -73.6 | -85.3 |
| C11H20O2 | Oxacyclododecan-2-one | -123.6 | -111.8 | -136.8 |
| C11H22O2 | Ethyl nonanoate | -141.1 | -131.7 | -140.2 |
| C11H24O2 | 1,1-Dibutoxypropane | -132.1 | -128.5 | -130.0 |
| C12H16O2 | 5,5-Dimethyl-2-phenyl-1,3-dioxane | -74.4 | -61.9 | -65.0 |
| C12H16O2 | Pentamethylbenzoic acid | -101.1 | -83.2 | -100.1 |
| C12H22O2 | 2,2,6,6-Tetramethyl-3,5-heptanedione | -126.3 | -90.0 | -123.5 |
| C12H24O2 | Ethyl decanoate | -146.3 | -136.4 | -145.7 |
| C13H8O2 | Xanthone | -23.5 | -22.3 | -18.7 |
| C13H10O2 | Phenyl benzoate | -34 | -26.9 | -28.3 |
| C8NH9O2 | 2,6-Dimethylnitrobenzene | 2.1 | 24.8 | 5.7 |
| C8NH9O2 | 2-Amino-2-phenylacetic acid | -67 | -63.7 | -64.3 |
| C8NH9O2 | 2-Nitro-m-xylene | 2.1 | 24.8 | 5.7 |
| C8NH9O2 | N-Phenylglycine | -64.2 | -62.6 | -73.1 |
| C8NH17O2 | 8-Aminocaprylic acid | -125 | -123.3 | -125.1 |
| C9NH7O2 | 2-Methyl-1H-isoindole-1,3(2H)-dione | -55.9 | -45.9 | -55.8 |
| C9NH11O2 | Nitromesitylene | -6.4 | 17.3 | -2.0 |
| C9NH11O2 | Phenylalanine | -69.3 | -68.8 | -71.9 |
| C10NH7O2 | 1-Nitroso-2-naphthalenol | 8.6 | -0.4 | 9.6 |
| C10NH13O2 | N,N-Dimethyl 4-methoxybenzamide | -53.7 | -49.0 | -54.2 |
| C10NH15O2 | 1-Nitroadamantane | -45.7 | -4.6 | -41.5 |
| C10NH15O2 | 2-Nitroadamantane | -43 | -3.8 | -41.6 |
| C8N2H8O2 | Isophthalamide | -70.3 | -53.3 | -62.1 |
| C8N2H8O2 | Teraphthalamide | -69.7 | -53.0 | -62.0 |
| C8N2H10O2 | N,N-Dimethyl-m-nitroaniline | 17.4 | 43.6 | 20.8 |
| C8N2H10O2 | N,N-Dimethyl-p-nitroaniline | 16.1 | 43.4 | 17.9 |
| C11N2H12O2 | Tryptophan | -51.6 | -48.2 | -54.8 |
| C6N3H9O2 | Histidine | -63.5 | -61.3 | -66.8 |
| C6N4H14O2 | Argenine | -84.3 | -75.6 | -78.9 |
| C10H14O3 | Trimethoxymethyl benzene | -103.6 | -98.1 | -88.1 |
| C10H22O3 | 1-tert-Butoxy-3-propoxy-2-propanol | -169.2 | -151.7 | -160.2 |
| C11H24O3 | 1-Butoxy-3-tert-butyl-2-propanol | -172.8 | -157.2 | -169.3 |
| C9NH11O3 | Tyrosine | -111.6 | -117.0 | -114.7 |
| C4N2H4O3 | Barbituric acid | -121.8 | -118.7 | -150.4 |
| C4N2H8O3 | Asparagine | -137.8 | -130.4 | -139.6 |
| C4N2H8O3 | GLY-GLY | -136.4 | -129.2 | -137.8 |
| C5N2H10O3 | Glutamine | -142.4 | -134.4 | -145.1 |
| C6N2H12O3 | ALA-ALA | -152.4 | -134.2 | -146.8 |
| C9N2H6O3 | 8-Hydroxy-5-nitroquinoline | -0.4 | 11.8 | 5.5 |
| C10N2H16O3 | PRO-PRO | -131.4 | -136.1 | -153.4 |
| C10N2H20O3 | VAL-VAL | -160.6 | -140.2 | -162.2 |
| C3N3H3O3 | 1,3,5-Triazine-2,4,6(1H,3H,5H)-trione | -134.8 | -118.8 | -157.0 |
| C6N3H9O3 | 1,3,5-Trimethyl-s-triazine-2,4,6-trione | -141.1 | -103.5 | -141.8 |
| C6N3H9O3 | 2,4,6-Trimethoxy-s-triazine | -70.1 | -99.7 | -68.6 |
| C7H12O4 | 2,2-Diacetoxypropane | -199.3 | -171.0 | -191.0 |
| C7H12O4 | Diethyl malonate | -190.1 | -181.0 | -191.9 |
| C7H12O4 | Dimethyl dimethylmalonate | -191.9 | -166.5 | -175.3 |
| C7H16O4 | 3,5,7,9-Tetraoxyundecane | -177.1 | -189.8 | -187.3 |
| C8H16O4 | 12-Crown-4 | -150.8 | -164.0 | -169.8 |
| C10H6O4 | 5,8-Dihydroxy-1,4-naphthalenedione | -119.3 | -113.6 | -113.4 |
| C10H10O4 | Dimethyl isophthalate | -150.4 | -141.0 | -139.6 |
| C10H10O4 | Dimethyl phthalate | -144.9 | -138.2 | -137.0 |
| C10H22O4 | 1-(tert-Butyldioxy)-3-propoxy-2-propanol | -157 | -130.0 | -148.4 |
| C11H12O4 | Benzal diacetate | -167.3 | -145.8 | -158.3 |
| C11H24O4 | 1-Butoxy-1-tert-butyldioxy-2-propanol | -161.3 | -138.9 | -159.1 |
| C6NH5O4 | 4-Nitrocatechol | -69.3 | -59.2 | -65.9 |
| C7NH5O4 | p-Nitrobenzoic acid | -68.7 | -51.2 | -67.6 |
| C6N2H4O4 | m-Dinitrobenzene | 11.3 | 54.8 | 22.3 |
| C6N2H4O4 | o-Dinitrobenzene | 20.2 | 54.8 | 22.3 |
| C6N2H4O4 | p-Dinitrobenzene | 13.3 | 54.6 | 22.3 |
| C7N2H6O4 | 2,4-Dinitrotoluene | 7.9 | 48.9 | 14.6 |
| C7N2H6O4 | Dinitrophenylmethane | 8.3 | 56.2 | 19.2 |
| C8H18O5 | 3,5,7,9,11-Pentaoxa-tridecane | -216.5 | -235.4 | -230.3 |
| C10H20O5 | 15-Crown-5 | -191.1 | -208.6 | -214.9 |
| C6N2H12O5 | SER-SER | -211.6 | -220.9 | -225.2 |
| C8N2H16O5 | THR-THR | -230.2 | -217.6 | -233.7 |
| C7H10O6 | Trimethyl methanetricarboxylate | -252.1 | -242.5 | -251.6 |
| C2N3H3O6 | 1,1,1-Trinitroethane | -12.4 | 56.4 | -0.3 |
| CN4O8 | Tetranitromethane | 18.5 | 95.0 | 25.9 |
| C3N3H5O9 | Glycerol trinitrate | -64.7 | -6.3 | -84.0 |
| C14NH21O | 4-Isopropylbenzylidene t-butylamine N-oxide | -12.2 | 37.2 | 6.4 |
| C14H8O2 | 9,10-Anthroquinone | -18.1 | -13.2 | -14.7 |
| C14H8O2 | 9,10-Phenanthroquinone | -11.1 | -10.7 | -12.7 |
| C13NH11O2 | Phenol, 2-[(phenylimino)methyl]-, N-oxide | 12.9 | 38.2 | 20.2 |
| C14NH13O2 | Benzenamine, N-[(4-methoxyphenyl)methylene]-, N-oxide | 26.2 | 45.8 | 31.2 |
| C15NH17O2 | N-(3-Phenoxy-2-hydroxypropyl)aniline | -44.1 | -28.4 | -39.1 |
| C14H10O3 | Benzoic acid, anhydride | -76.3 | -66.1 | -71.0 |
| C12N2H24O3 | ILE-ILE | -176.5 | -144.8 | -173.1 |
| C12N2H24O3 | LEU-LEU | -177.1 | -150.6 | -177.2 |
| C12H14O4 | 1,1-Ethanediol, 2-phenyl-, diacetate | -167.3 | -151.0 | -167.6 |
| C12H14O4 | Benzyl diacetate | -161.8 | -151.0 | -167.6 |
| C14H6O4 | 1,4,9,10-Anthracenetetrone | -50 | -62.1 | -66.4 |
| C14H8O4 | 1,4-Dihydroxy-9,10-anthracenedione | -112.6 | -104.9 | -101.0 |
| C14H10O4 | Diphenyl oxalate | -104.5 | -97.0 | -101.2 |
| C14H12O4 | Dimethyl naphthalene-2,6-dicarboxylate | -132.7 | -124.0 | -121.3 |
| C10H16O6 | Triethyl methanetricarboxylate | -280.4 | -258.5 | -274.0 |
| C11H18O6 | Triethyl 1,1,1-ethanetricarboxylate | -286.2 | -252.9 | -269.2 |
| C7N3H5O6 | 2,4,6-Trinitrotoluene | 12.9 | 74.6 | 23.3 |
| C9NH9O7 | 2-(Diacetoxymethyl)-5-nitrofuran | -184.4 | -161.3 | -186.5 |
| C7N3H5O7 | 2,4,6-Trinitroanisole | -5.8 | 39.9 | -3.4 |
| C8N3H7O7 | 2,4,6-Trinitrophenetole | -20.1 | 36.7 | -10.9 |
| F | Fluorine, atom | 18.9 | 18.9 | 1.8 |
| F | Fluoride, anion | -61 | -17.1 | -61.2 |
| HF | Hydrogen fluoride | -65.1 | -59.7 | -77.9 |
| CF | Fluoromethylidyne radical | 61 | 38.2 | 46.0 |
| CH2F | Fluoromethyl, cation | 200.3 | 182.8 | 198.1 |
| CH3F | Fluoromethane | -56.8 | -60.9 | -60.5 |
| C2HF | Fluoroacetylene | 30 | 15.6 | 5.6 |
| C2H3F | Fluoroethylene | -32.5 | -34.6 | -37.2 |
| C2H4F | CH3CHF, cation | 166 | 164.7 | 159.4 |
| C2H5F | Fluoroethane | -62.9 | -65.1 | -66.9 |
| C3H7F | 2-Fluoropropane | -69.4 | -66.7 | -71.5 |
| C6H5F | Fluorobenzene | -27.8 | -25.3 | -25.7 |
| C6H11F | Fluorocyclohexane | -80.5 | -76.5 | -82.9 |
| C9H19F | 1-Fluorononane | -101.2 | -98.5 | -104.7 |
| CNF | Cyanogen fluoride | 8.6 | -2.3 | -8.4 |
| OF | Fluorine oxide | 26.1 | 20.4 | 10.1 |
| HOF | Hypofluorous acid | -20.9 | -18.6 | -30.7 |
| CHOF | HCOF | -90 | -88.8 | -96.3 |
| C2H3OF | Acetyl fluoride | -106.4 | -96.5 | -107.1 |
| NOF | Nitrosyl fluoride | -15.7 | -24.7 | -15.5 |
| O2F | Fluorine dioxide | 3 | 22.8 | -6.3 |
| C7H5O2F | m-fluorobenzoic acid | -118.4 | -113.2 | -113.5 |
| NO2F | Fluorine nitrite | -26 | 0.8 | -22.5 |
| NO3F | Fluorine nitrate | 2.5 | 28.2 | -7.0 |
| CN2HO4F | Fluorodinitromethane | -56.1 | -18.2 | -53.6 |
| F2 | Fluorine molecule | 0 | 7.4 | -9.1 |
| CF2 | Difluoromethylene | -45 | -65.2 | -61.8 |
| CHF2 | Difluoromethyl, cation | 142.4 | 132.4 | 141.7 |
| CH2F2 | Difluoromethane, cation | 185.2 | 177.6 | 142.3 |
| CH2F2 | Difluoromethane | -108.1 | -111.7 | -118.0 |
| C2F2 | Difluoroacetylene | 5 | -21.0 | -23.0 |
| C2H2F2 | gem-Difluoroethylene | -80.5 | -83.7 | -86.6 |
| C2H3F2 | CH3CF2, cation | 107 | 116.6 | 112.0 |
| C2H4F2 | 1,1-Difluoroethane | -118.8 | -113.4 | -122.9 |
| C6H4F2 | 1,2-Difluorobenzene | -67.7 | -70.6 | -69.7 |
| C6H4F2 | 1,3-Difluorobenzene | -73.9 | -71.0 | -71.2 |
| C6H4F2 | 1,4-Difluorobenzene | -73.3 | -71.1 | -71.3 |
| C4NH9F2 | t-Butyldifluoroamine | -46 | -21.1 | -31.8 |
| C7NH7F2 | N,N'-Difluorobenzylamine | 1.8 | 11.3 | 5.8 |
| N2F2 | cis-Difluorodiazene | 16.4 | -2.3 | 12.6 |
| N2F2 | trans-Difluorodiazene | 19.4 | 2.3 | 25.0 |
| OF2 | Difluorine oxide | 5.9 | 18.3 | -9.9 |
| COF2 | Carbonyl fluoride | -152.7 | -138.5 | -152.0 |
| CF3 | Trifluoromethyl, cation | 99.3 | 101.0 | 106.5 |
| CF3 | Trifluoromethyl | -112.4 | -138.6 | -151.4 |
| CF3 | Trifluoromethyl, anion | -163.4 | -178.7 | -194.7 |
| CHF3 | Trifluoromethane | -166.3 | -163.7 | -175.8 |
| C2HF3 | Trifluoroethylene | -117.3 | -131.1 | -131.6 |
| C2H2F3 | CF3CH2, cation | 114 | 121.3 | 117.4 |
| C2H2F3 | CH2F.CF2, radical cation | 81 | 82.4 | 79.2 |
| C2H2F3 | CF3CH2 radical | -123.6 | -131.1 | -139.7 |
| C2H3F3 | 1,1,1-Trifluoroethane | -178.9 | -164.3 | -176.7 |
| C7H5F3 | Trifluoromethylbenzene | -143.2 | -127.5 | -137.4 |
| NF3 | Nitrogen trifluoride | -31.6 | -34.1 | -32.8 |
| C2NF3 | Trifluoroacetonitrile | -118.4 | -113.1 | -120.9 |
| C2NF3 | Trifluoromethylisocyanide | -99.7 | -90.3 | -98.5 |
| NOF3 | F3NO | -39 | 23.0 | -17.0 |
| C2HO2F3 | Trifluoroacetic acid | -255 | -238.0 | -249.4 |
| CF4 | Carbon tetrafluoride | -223.3 | -214.0 | -223.5 |
| C2F4 | Tetrafluoroethylene | -157.9 | -175.6 | -173.5 |
| C6H2F4 | 1,2,4,5-Tetrafluorobenzene | -154.6 | -159.2 | -155.5 |
| N2F4 | Tetrafluorohydrazine | -2 | -19.6 | -14.7 |
| COF4 | Perfluoromethanol | -182.8 | -163.1 | -184.5 |
| CO2F4 | Bis(fluoroxy)perfluoromethane | -134.9 | -112.1 | -144.6 |
| CNF5 | Pentafluoromethylamine | -169 | -163.1 | -167.6 |
| CN3F5 | Pentafluoroguanidine | 22.9 | 6.2 | 19.1 |
| C2F6 | Hexafluoroethane | -321.2 | -299.4 | -319.0 |
| C4F6 | Perfluorobutadiene | -253.4 | -250.0 | -244.6 |
| CN2F6 | Hexafluorodimethylamine | -108.8 | -111.9 | -110.3 |
| C2OF6 | Dimethyl perfluoroether | -369 | -357.3 | -370.4 |
| C3OF6 | Perfluoroacetone | -342.6 | -322.2 | -343.7 |
| C7N2H5O4F | Fluorodinitrophenylmethane | -16.9 | 14.6 | -19.5 |
| C7H4F4 | 1-Fluoro-3-(trifluoro-methyl)benzene | -189.4 | -172.3 | -182.3 |
| C6N2H10F4 | N,N,N',N'-tetrafluoro-1,1-cyclohexanediamine | -41.6 | -24.7 | -38.4 |
| C6N2H12F4 | N,N,N',N'-Tetrafluoro-4-methyl-1,2-pentane | -52.8 | -26.5 | -53.3 |
| C7N2H14F4 | 1,1-Bis(difluoroamine)heptane | -52.8 | -42.5 | -54.6 |
| C6HF5 | Pentafluorobenzene | -192.5 | -201.7 | -194.8 |
| C7H3F5 | 2,3,4,5,6-Pentafluorotoluene | -201.6 | -206.6 | -199.9 |
| C6HOF5 | Pentafluorophenol | -228.8 | -247.9 | -233.3 |
| C6F6 | Hexafluorobenzene | -242.5 | -243.5 | -233.2 |
| C3F8 | Perfluoropropane | -426.2 | -384.3 | -413.5 |
| C4F8 | Perfluorobut-2-ene | -389.9 | -365.9 | -383.5 |
| C4F8 | Perfluorocyclobutane | -369.5 | -363.7 | -385.8 |
| C7F8 | Octafluorotoluene | -356.8 | -340.4 | -341.9 |
| CN4F8 | Octafluoromethanetetramine | 0.4 | -3.8 | 3.4 |
| C3O2F8 | Perfluorodimethoxymethane | -520.6 | -501.3 | -517.0 |
| C4F10 | n-Perfluorobutane | -515.3 | -469.0 | -507.7 |
| C7H6O4F6 | Hexafluoropentanedioic acid, dimethyl ester | -460.5 | -419.7 | -448.9 |
| C6F10 | Decafluorocyclohexene | -461.9 | -423.1 | -453.6 |
| C5NF11 | Undecafluoropiperidine | -478.9 | -436.9 | -475.2 |
| C2N5F11 | Tetrakis(difluoroamine)-N-1,1-trifluorodimethylamine | -84.4 | -85.4 | -84.9 |
| C6F12 | Dodecafluorocyclohexane | -590.5 | -522.5 | -574.3 |

Values for $I.E.$ (given in $\mathrm{eV}$)

| **Molecular Formula** | **Molecule Name** | **Ref.** | **Initial** | **Final** |
| --- | --- | --- | --- | --- |
| H2 | Hydrogen | 15.4 | 15.75 | 15.01 |
| CH4 | Methane | 13.6 | 13.86 | 12.94 |
| C2H2 | Acetylene | 11.4 | 11.01 | 12.06 |
| C2H4 | Ethylene | 10.51 | 10.18 | 10.87 |
| C2H6 | Ethane | 12 | 12.7 | 11.10 |
| C3 | Carbon, trimer | 11.1 | 11.04 | 10.54 |
| C3H4 | Allene | 10.07 | 10.02 | 10.30 |
| C3H4 | Cyclopropene | 9.86 | 9.88 | 10.01 |
| C3H4 | Propyne | 10.37 | 10.72 | 10.86 |
| C3H6 | Cyclopropane | 11 | 11.43 | 11.38 |
| C3H6 | Propene | 9.88 | 9.96 | 10.02 |
| C3H8 | Propane | 11.5 | 12.34 | 10.53 |
| C4H2 | Diacetylene | 10.17 | 9.99 | 10.99 |
| C4H4 | Vinylacetylene | 9.1 | 9.5 | 10.27 |
| C4H4 | Butatriene | 9.15 | 9.01 | 9.74 |
| C4H6 | 1,2-Butadiene | 9.15 | 9.84 | 9.56 |
| C4H6 | 1,3-Butadiene | 9.08 | 9.14 | 9.70 |
| C4H6 | 1-Butyne | 10.2 | 10.68 | 10.56 |
| C4H6 | 2-Butyne | 9.6 | 10.47 | 9.99 |
| C4H6 | Cyclobutene | 9.43 | 9.77 | 9.76 |
| C4H8 | 1-Butene | 9.7 | 9.95 | 9.92 |
| C4H8 | Cyclobutane | 10.7 | 11.8 | 10.11 |
| C4H10 | Isobutane | 11.4 | 12.12 | 10.53 |
| C4H10 | n-Butane, trans | 11.2 | 12.21 | 10.31 |
| C5H6 | Cyclopentadiene | 8.57 | 9.04 | 9.29 |
| C5H8 | Cyclopentene | 9.18 | 9.72 | 9.15 |
| C5H10 | Cyclopentane | 10.5 | 12.06 | 10.14 |
| C5H12 | n-Pentane | 10.3 | 12.16 | 10.23 |
| C5H12 | Neopentane | 11.3 | 12.11 | 10.96 |
| C6H6 | Benzene | 9.25 | 9.39 | 9.83 |
| C6H10 | Cyclohexene | 10.3 | 9.75 | 9.20 |
| C6H12 | Cyclohexane | 10.3 | 11.74 | 10.17 |
| C7H8 | Cycloheptatriene | 8.5 | 8.58 | 8.81 |
| C7H8 | Toluene | 8.82 | 9.28 | 9.31 |
| C8H10 | Ethylbenzene | 8.8 | 9.28 | 9.40 |
| C8H14 | Bicyclo(2.2.2)-octane | 9.45 | 11.4 | 9.80 |
| C10H8 | Naphthalene | 8.15 | 8.58 | 9.01 |
| C10H16 | Adamantane | 9.6 | 11.27 | 9.64 |
| C14H10 | Anthracene | 8.16 | 8.05 | 8.46 |
| NH3 | Ammonia | 10.85 | 11.19 | 9.77 |
| CNH | Hydrogen cyanide | 13.6 | 13.41 | 12.63 |
| CNH5 | Methylamine | 9.6 | 10.56 | 8.93 |
| C2NH3 | Acetonitrile | 12.21 | 12.79 | 12.25 |
| C2NH3 | Methyl isocyanide | 11.32 | 12.24 | 10.61 |
| C2NH5 | Ethyleneimine (Azirane) | 9.9 | 10.68 | 9.79 |
| C2NH7 | Dimethylamine | 8.93 | 10.04 | 8.63 |
| C2NH7 | Ethylamine | 9.5 | 10.5 | 9.17 |
| C3NH3 | Acrylonitrile | 10.91 | 10.61 | 11.19 |
| C3NH5 | Ethyl cyanide | 11.9 | 12.59 | 11.50 |
| C3NH9 | Trimethylamine | 8.54 | 9.59 | 8.53 |
| C4NH5 | Pyrrole | 8.21 | 8.56 | 9.09 |
| C5NH5 | Pyridine | 9.67 | 9.69 | 10.11 |
| C6NH7 | Aniline | 7.7 | 8.75 | 8.50 |
| C7NH5 | Phenyl cyanide | 9.7 | 9.81 | 10.20 |
| N2 | Nitrogen | 15.6 | 14.87 | 12.54 |
| CN2H2 | Diazomethane | 9 | 8.66 | 9.36 |
| CN2H6 | Methylhydrazine | 9.3 | 9.66 | 8.88 |
| C2N2 | Cyanogen | 13.36 | 13.2 | 13.05 |
| H2O | Water | 12.62 | 12.19 | 12.03 |
| CO | Carbon monoxide | 14.01 | 13.43 | 11.12 |
| CH2O | Formaldehyde | 10.1 | 11.04 | 10.74 |
| CH4O | Methanol | 10.96 | 11.41 | 10.72 |
| C2H2O | Ketene | 9.64 | 9.29 | 10.09 |
| C2H4O | Acetaldehyde | 10.21 | 10.88 | 10.70 |
| C2H4O | Ethylene oxide | 10.57 | 11.49 | 11.23 |
| C2H6O | Dimethyl ether | 10.04 | 11.04 | 10.18 |
| C2H6O | Ethanol | 10.6 | 11.3 | 10.36 |
| C3H6O | Acetone | 9.72 | 10.75 | 10.64 |
| C3H6O | Propanal | 10 | 10.82 | 10.49 |
| C4H4O | Furan | 8.88 | 9.14 | 9.64 |
| C4H8O | Butanal | 9.83 | 10.81 | 10.52 |
| C4H10O | Diethyl ether | 9.6 | 10.91 | 10.28 |
| C7H6O | Benzaldehyde | 9.7 | 9.74 | 10.15 |
| C7H8O | Anisole | 8.4 | 8.84 | 9.07 |
| CNHO | Hydrogen isocyanate | 11.6 | 11.1 | 11.50 |
| CH2O2 | Formic acid | 11.51 | 11.74 | 11.78 |
| C2H2O2 | trans Glyoxal | 10.59 | 10.75 | 10.67 |
| C2H4O2 | Acetic acid | 10.8 | 11.57 | 11.57 |
| C2H4O2 | Methyl formate | 11.02 | 11.61 | 11.37 |
| C2H6O2 | Dimethyl peroxide | 10.6 | 10.57 | 10.40 |
| C3O2 | Carbon suboxide | 10.6 | 10.07 | 10.85 |
| C3H4O2 | beta-Propiolactone | 10.6 | 11.4 | 11.41 |
| C3H6O2 | Methyl acetate | 10.6 | 11.46 | 11.22 |
| C3H6O2 | Propionic acid | 10.5 | 11.52 | 11.26 |
| C3H8O2 | 2-Methoxyethanol | 9.8 | 11.02 | 10.11 |
| C5H8O2 | Acetylacetone | 9.15 | 10.79 | 10.69 |
| C7H6O2 | Benzoic acid | 9.8 | 9.76 | 10.17 |
| CNH3O2 | Methyl nitrite | 11 | 11.42 | 11.05 |
| C2NH5O2 | Ethyl nitrite | 11.3 | 11.36 | 10.87 |
| C3NH7O2 | Alanine | 8.9 | 10.81 | 9.55 |
| C6NH5O2 | Nitrobenzene | 9.9 | 10.31 | 10.76 |
| O3 | Ozone | 12.75 | 12.71 | 12.69 |
| C4H2O3 | Malaic anhydride | 10.84 | 11.7 | 12.21 |
| C7H6O3 | o-Salicylic acid | 9.8 | 9.26 | 9.47 |
| C2H2O4 | Oxalic acid | 11.2 | 11.67 | 11.32 |
| N2O4 | Dinitrogen tetroxide | 11.4 | 12.05 | 12.60 |
| N2O5 | Dinitrogen pentoxide | 12.3 | 13.18 | 13.63 |
| HF | Hydrogen fluoride | 16.06 | 14.82 | 13.82 |
| CH3F | Fluoromethane | 13.31 | 13.05 | 12.59 |
| C2HF | Fluoroacetylene | 11.3 | 11.06 | 12.01 |
| C2H3F | Fluoroethylene | 10.58 | 10.17 | 10.93 |
| C2H5F | Fluoroethane | 12.43 | 12.61 | 11.40 |
| C3H7F | 2-Fluoropropane | 11.08 | 12.33 | 11.35 |
| C6H5F | Fluorobenzene | 9.19 | 9.47 | 9.95 |
| NOF | Nitrosyl fluoride | 12.94 | 12.93 | 13.01 |
| C7H5O2F | m-fluorobenzoic acid | 9.9 | 9.83 | 10.25 |
| NO2F | Fluorine nitrite | 13.51 | 12.99 | 13.96 |
| CH2F2 | Difluoromethane | 13.17 | 13.09 | 12.76 |
| C2F2 | Difluoroacetylene | 11.2 | 11.17 | 11.92 |
| C2H2F2 | gem-Difluoroethylene | 10.72 | 10.18 | 10.88 |
| C2H4F2 | 1,1-Difluoroethane | 12.8 | 12.73 | 12.45 |
| N2F2 | trans-Difluorodiazene | 13.4 | 13 | 12.74 |
| OF2 | Difluorine oxide | 13.26 | 13.52 | 14.33 |
| CHF3 | Trifluoromethane | 14.8 | 14.57 | 14.12 |
| C2HF3 | Trifluoroethylene | 10.54 | 10.46 | 11.02 |
| C2H3F3 | 1,1,1-Trifluoroethane | 13.8 | 14.01 | 13.71 |
| C7H5F3 | Trifluoromethylbenzene | 9.68 | 10.07 | 10.47 |
| NF3 | Nitrogen trifluoride | 13.73 | 13.93 | 14.19 |
| C2HO2F3 | Trifluoroacetic acid | 12 | 12.73 | 12.61 |
| CF4 | Carbon tetrafluoride | 16.23 | 16.81 | 15.73 |
| C2F4 | Tetrafluoroethylene | 10.5 | 10.74 | 11.11 |
| N2F4 | Tetrafluorohydrazine | 12 | 13.19 | 13.62 |
| C2F6 | Hexafluoroethane | 14.6 | 14.5 | 14.11 |
| C3OF6 | Perfluoroacetone | 12.1 | 13 | 12.85 |
| C6HF5 | Pentafluorobenzene | 9.75 | 10.4 | 10.81 |
| C6F6 | Hexafluorobenzene | 10.9 | 10.78 | 11.12 |

Values for $\left\langle\mu\right\rangle$ (given in $\mathrm{Debyes}$)

| **Molecular Formula** | **Molecule Name** | **Expt.** | **Initial** | **Final** |
| --- | --- | --- | --- | --- |
| C3H4 | Cyclopropene | 0.45 | 0.48 | 0.02 |
| C3H4 | Propyne | 0.78 | 0.12 | 1.15 |
| C3H6 | Propene | 0.37 | 0.04 | 0.59 |
| C3H8 | Propane | 0.08 | 0.00 | 0.02 |
| C4H6 | Bicyclobutane | 0.68 | 0.41 | 0.20 |
| C4H6 | Cyclobutene | 0.13 | 0.08 | 0.57 |
| C5H6 | Cyclopentadiene | 0.42 | 0.18 | 0.96 |
| C5H8 | Cyclopentene | 0.2 | 0.06 | 0.51 |
| C6H6 | Fulvene | 0.42 | 0.70 | 0.63 |
| C7H8 | Toluene | 0.36 | 0.05 | 0.74 |
| NH3 | Ammonia | 1.47 | 1.75 | 0.00 |
| CNH | Hydrogen cyanide | 2.98 | 2.50 | 2.11 |
| CNH5 | Methylamine | 1.31 | 1.48 | 0.65 |
| C2NH3 | Acetonitrile | 3.92 | 2.63 | 3.25 |
| C2NH3 | Methyl isocyanide | 3.85 | 2.17 | 3.26 |
| C2NH5 | Ethyleneimine (Azirane) | 1.9 | 1.75 | 1.94 |
| C2NH7 | Dimethylamine | 1.03 | 1.17 | 1.30 |
| C2NH7 | Ethylamine | 1.22 | 1.52 | 1.10 |
| C3NH3 | Acrylonitrile | 3.87 | 2.97 | 3.04 |
| C3NH9 | Trimethylamine | 0.61 | 0.75 | 1.37 |
| C4NH5 | Pyrrole | 1.74 | 1.81 | 1.83 |
| C5NH5 | Pyridine | 2.22 | 1.96 | 2.07 |
| C6NH7 | Aniline | 1.53 | 1.46 | 1.41 |
| CN2H2 | Diazomethane | 1.5 | 1.25 | 1.65 |
| CN2H2 | N=N-CH2- | 1.59 | 1.55 | 1.94 |
| CN2H6 | Methylhydrazine | 1.66 | 0.24 | 2.05 |
| H2O | Water | 1.85 | 1.78 | 2.11 |
| CO | Carbon monoxide | 0.11 | 0.20 | 0.23 |
| CH2O | Formaldehyde | 2.33 | 2.17 | 2.65 |
| CH4O | Methanol | 1.7 | 1.48 | 2.05 |
| C2H2O | Ketene | 1.42 | 1.04 | 1.67 |
| C2H4O | Acetaldehyde | 2.69 | 2.38 | 3.08 |
| C2H4O | Ethylene oxide | 1.89 | 1.92 | 2.26 |
| C2H6O | Dimethyl ether | 1.3 | 1.27 | 1.87 |
| C2H6O | Ethanol | 1.69 | 1.40 | 2.14 |
| C3H6O | Acetone | 2.88 | 2.50 | 3.15 |
| C4H4O | Furan | 0.66 | 0.42 | 0.63 |
| C4H10O | Diethyl ether | 1.15 | 1.09 | 1.87 |
| C6H6O | Phenol | 1.45 | 1.17 | 1.41 |
| C7H8O | Anisole | 1.38 | 1.07 | 1.70 |
| C3NH7O | Dimethylformamide | 3.82 | 3.06 | 4.05 |
| N2O | Nitrous oxide | 0.17 | 0.77 | 0.83 |
| CH2O2 | Formic acid | 1.41 | 1.49 | 1.43 |
| C2H4O2 | Acetic acid | 1.74 | 1.68 | 1.78 |
| C2H4O2 | Methyl formate | 1.77 | 1.63 | 1.72 |
| C3H6O2 | Methyl acetate | 1.72 | 1.75 | 1.73 |
| C3H6O2 | Propionic acid | 1.75 | 1.64 | 1.79 |
| NHO2 | Nitrous acid, trans | 1.86 | 2.28 | 2.64 |
| O3 | Ozone | 0.53 | 1.18 | 1.29 |
| NHO3 | Nitric acid | 2.17 | 2.78 | 2.76 |
| HF | Hydrogen fluoride | 1.83 | 1.99 | 1.62 |
| CH3F | Fluoromethane | 1.86 | 1.76 | 2.10 |
| C2HF | Fluoroacetylene | 0.7 | 1.57 | 1.25 |
| C2H3F | Fluoroethylene | 1.43 | 1.70 | 1.87 |
| C2H5F | Fluoroethane | 1.96 | 1.87 | 2.28 |
| C6H5F | Fluorobenzene | 1.66 | 1.96 | 2.07 |
| CNF | Cyanogen fluoride | 2.17 | 0.89 | 0.75 |
| HOF | Hypofluorous acid | 2.23 | 1.81 | 2.32 |
| CHOF | HCOF | 2.02 | 2.50 | 2.51 |
| NOF | Nitrosyl fluoride | 1.81 | 0.51 | 1.36 |
| NO2F | Fluorine nitrite | 0.47 | 0.66 | 0.77 |
| CH2F2 | Difluoromethane | 1.96 | 2.21 | 2.37 |
| C2H4F2 | 1,1-Difluoroethane | 2.3 | 2.50 | 2.82 |
| N2F2 | cis-Difluorodiazene | 0.16 | 0.02 | 0.62 |
| OF2 | Difluorine oxide | 0.3 | 0.32 | 0.86 |
| COF2 | Carbonyl fluoride | 0.95 | 0.81 | 0.44 |
| CHF3 | Trifluoromethane | 1.65 | 2.23 | 2.18 |
| C2HF3 | Trifluoroethylene | 1.3 | 1.82 | 1.65 |
| C2H3F3 | 1,1,1-Trifluoroethane | 2.32 | 2.87 | 3.07 |
| NF3 | Nitrogen trifluoride | 0.24 | 0.20 | 0.76 |
| C2NF3 | Trifluoroacetonitrile | 1.26 | 0.36 | 0.32 |
| C2HO2F3 | Trifluoroacetic acid | 2.28 | 2.45 | 2.85 |

Values for $\left| \mathbf{g} \right|$ (given in $kcal/(mol\cdot bohr)$)

| **Molecular Formula** | **Molecule Name** | **Initial** | **Final** |
| --- | --- | --- | --- |
| H2 | Hydrogen | 40.249 | 52.187 |
| CH2 | Methylene, singlet | 19.276 | 11.754 |
| CH2 | Methylene, triplet | 26.903 | 15.460 |
| CH3 | Methyl, cation | 3.512 | 5.395 |
| CH4 | Methane | 13.162 | 3.430 |
| C2H2 | Acetylene | 9.043 | 15.677 |
| C2H3 | Vinyl, cation | 44.877 | 29.066 |
| C2H3 | Vinyl | 0.391 | 17.207 |
| C2H4 | Ethylene, cation | 8.075 | 11.443 |
| C2H4 | Ethylene | 7.216 | 7.344 |
| C2H4 | Methylmethylene | 13.604 | 24.882 |
| C2H5 | Ethyl, cation | 47.440 | 20.017 |
| C2H5 | Ethyl radical | 16.467 | 15.797 |
| C2H6 | Ethane | 21.822 | 10.730 |
| C3 | Carbon, trimer | 17.716 | 14.392 |
| C3H3 | Cyclopropenyl, cation | 123.215 | 92.829 |
| C3H3 | Propynyl, cation | 18.987 | 11.366 |
| C3H4 | Allene | 6.419 | 8.729 |
| C3H4 | Propyne | 21.322 | 15.551 |
| C3H5 | Allyl, cation | 16.256 | 15.540 |
| C3H5 | Allyl | 15.268 | 9.568 |
| C3H6 | Cyclopropane | 24.308 | 16.632 |
| C3H6 | Propene | 19.881 | 9.559 |
| C3H7 | i-Propyl radical | 26.969 | 14.921 |
| C3H8 | Propane | 23.422 | 9.306 |
| C4 | Carbon, tetramer | 0.054 | 8.193 |
| C4H2 | Diacetylene | 16.398 | 37.058 |
| C4H4 | Vinylacetylene | 16.331 | 20.697 |
| C4H4 | Butatriene | 10.203 | 24.536 |
| C4H6 | 1,2-Butadiene | 20.758 | 15.980 |
| C4H6 | 1,3-Butadiene | 10.563 | 13.962 |
| C4H6 | 1-Butyne | 26.050 | 17.390 |
| C4H6 | 2-Butyne | 28.664 | 14.399 |
| C4H6 | Bicyclobutane | 71.719 | 63.762 |
| C4H6 | Cyclobutene | 27.983 | 16.017 |
| C4H6 | Methyl cyclopropene | 67.177 | 60.092 |
| C4H6 | Methylenecyclopropane | 26.722 | 30.665 |
| C4H8 | 1-Butene | 24.330 | 11.238 |
| C4H8 | cis-2-Butene | 27.688 | 16.122 |
| C4H8 | Cyclobutane | 21.936 | 17.444 |
| C4H8 | Isobutene | 26.057 | 11.277 |
| C4H8 | trans-2-Butene | 28.551 | 15.413 |
| C4H9 | Isobutyl | 23.183 | 12.969 |
| C4H10 | Isobutane | 24.439 | 7.759 |
| C4H10 | n-Butane, trans | 26.302 | 9.570 |
| C5H5 | Cyclopentadienyl, anion | 16.858 | 23.711 |
| C5H6 | Cyclopentadiene | 30.305 | 14.418 |
| C5H8 | 1,4-Pentadiene | 24.387 | 9.954 |
| C5H8 | 1,cis-3-Pentadiene | 29.799 | 12.709 |
| C5H8 | 1,trans-3-Pentadiene | 20.673 | 15.786 |
| C5H8 | Bicyclo(2.1.0)-pentane | 37.177 | 30.853 |
| C5H8 | Cyclopentene | 30.052 | 16.229 |
| C5H8 | Isoprene | 21.221 | 15.038 |
| C5H8 | Methylene cyclobutane | 23.437 | 18.069 |
| C5H8 | Spiropentane | 28.996 | 24.211 |
| C5H10 | 2-Methyl-2-butene | 32.403 | 18.438 |
| C5H10 | cis-2-Pentene | 30.831 | 16.176 |
| C5H10 | Cyclopentane | 29.826 | 16.038 |
| C5H10 | trans-2-Pentene | 31.253 | 14.941 |
| C5H12 | n-Pentane | 29.531 | 10.259 |
| C5H12 | Neopentane | 24.782 | 8.028 |
| C6H6 | Benzene | 20.254 | 6.026 |
| C6H6 | Fulvene | 28.377 | 17.945 |
| C6H8 | (E)-1,3,5-Hexatriene | 11.884 | 20.958 |
| C6H8 | (Z)-1,3,5-Hexatriene | 12.152 | 18.315 |
| C6H8 | 1,3-Cyclohexadiene | 20.353 | 13.487 |
| C6H8 | 1,4-Cyclohexadiene | 29.609 | 14.660 |
| C6H10 | 1,5-Hexadiene | 26.448 | 10.864 |
| C6H10 | 1-Methyl cyclopentene | 36.114 | 19.957 |
| C6H10 | 3-Methyl cyclopentene | 32.239 | 15.151 |
| C6H10 | 4-Methyl cyclopentene | 31.351 | 14.494 |
| C6H10 | Cyclohexene | 30.643 | 13.430 |
| C6H12 | 1-Hexene | 28.993 | 9.829 |
| C6H12 | 2,3-Dimethyl-1-butene | 33.167 | 16.006 |
| C6H12 | 2,3-Dimethyl-2-butene | 28.397 | 17.338 |
| C6H12 | (Z)-3-Methyl-2-pentene | 36.289 | 19.672 |
| C6H12 | Cyclohexane | 29.769 | 10.258 |
| C6H14 | 2,2-Dimethyl butane | 30.004 | 12.138 |
| C6H14 | 2,3-Dimethyl butane | 30.809 | 11.911 |
| C6H14 | 2-Methyl pentane | 31.084 | 10.973 |
| C6H14 | 3-Methyl pentane | 32.647 | 13.664 |
| C6H14 | n-Hexane | 31.879 | 10.562 |
| C7H7 | Benzyl, cation | 43.009 | 43.100 |
| C7H7 | Tropylium cation | 70.279 | 61.893 |
| C7H8 | Norbornadiene | 45.765 | 20.212 |
| C7H8 | Toluene | 28.952 | 15.622 |
| C7H12 | Norbornane | 28.769 | 15.425 |
| C7H16 | 2,4-Dimethyl pentane | 33.228 | 11.057 |
| C7H16 | 3-Ethyl pentane | 34.650 | 13.695 |
| C7H16 | n-Heptane | 33.964 | 10.744 |
| C8H8 | Cubane | 10.043 | 20.375 |
| C8H8 | Cyclooctatetraene | 32.305 | 11.589 |
| C8H8 | Styrene | 24.666 | 10.270 |
| C8H10 | Ethylbenzene | 31.377 | 14.900 |
| C8H10 | m-Xylene | 35.794 | 21.375 |
| C8H10 | p-Xylene | 42.984 | 24.998 |
| C8H14 | Bicyclo(2.2.2)-octane | 38.031 | 19.785 |
| C8H18 | 2,2,3,3-Tetramethyl butane | 40.281 | 15.966 |
| C8H18 | n-Octane | 35.933 | 10.962 |
| C10H8 | Azulene | 30.682 | 17.700 |
| C10H8 | Naphthalene | 23.013 | 13.449 |
| C10H16 | Camphene | 31.978 | 9.212 |
| C10H22 | n-Decane | 39.694 | 11.480 |
| C12H8 | Acenaphthylene | 41.638 | 28.347 |
| C12H8 | Biphenylene | 36.731 | 30.415 |
| C12H10 | Biphenyl | 31.376 | 12.517 |
| C14H10 | Anthracene | 22.757 | 16.905 |
| C14H10 | Phenanthrene | 22.757 | 16.905 |
| C16H10 | Pyrene | 29.386 | 13.055 |
| NH2 | Amidogen | 22.160 | 13.559 |
| NH3 | Ammonia | 7.739 | 20.484 |
| NH4 | Ammonium, cation | 4.007 | 12.680 |
| CN | Cyanide | 52.121 | 62.391 |
| CNH | Hydrogen cyanide | 14.852 | 7.393 |
| CNH4 | CH2-NH2, cation | 49.919 | 23.295 |
| CNH5 | Methylamine | 24.489 | 17.299 |
| C2NH3 | Acetonitrile | 28.120 | 20.165 |
| C2NH3 | Methyl isocyanide | 29.041 | 10.348 |
| C2NH5 | Ethyleneimine (Azirane) | 25.641 | 17.491 |
| C2NH7 | Dimethylamine | 26.785 | 10.852 |
| C2NH7 | Ethylamine | 22.568 | 23.890 |
| C3NH3 | Acrylonitrile | 28.833 | 34.467 |
| C3NH5 | Ethyl cyanide | 24.752 | 16.005 |
| C3NH7 | Cyclopropylamine | 47.982 | 37.357 |
| C3NH9 | Isopropylamine | 35.110 | 17.257 |
| C3NH9 | n-Propylamine | 20.692 | 28.640 |
| C3NH9 | Trimethylamine | 43.326 | 26.499 |
| C4NH5 | (E)-2-Butenenitrile | 22.960 | 14.547 |
| C4NH5 | (Z)-2-Butenenitrile | 24.208 | 17.381 |
| C4NH5 | Pyrrole | 41.911 | 16.167 |
| C4NH7 | Butanenitrile | 37.375 | 17.034 |
| C4NH7 | Isobutane nitrile | 39.260 | 37.022 |
| C4NH9 | Pyrrolidine | 24.169 | 21.793 |
| C4NH11 | 2-Butylamine | 29.298 | 22.550 |
| C4NH11 | 2-Methyl-1-propylamine | 28.162 | 22.519 |
| C4NH11 | N-Butylamine | 19.993 | 29.097 |
| C4NH11 | t-Butylamine | 34.573 | 19.032 |
| C5NH5 | Pyridine | 28.533 | 13.794 |
| C5NH7 | N-Methyl pyrrole | 48.235 | 22.594 |
| C5NH9 | 1,2,3,6-Tetrahydropyridine | 29.857 | 13.086 |
| C5NH9 | 2-Cyanobutane | 32.884 | 16.028 |
| C5NH9 | Butyl cyanide | 29.213 | 15.217 |
| C5NH9 | t-Butylnitrile | 25.843 | 12.721 |
| C5NH11 | Cyclopentylamine | 34.233 | 22.549 |
| C5NH11 | Piperidine | 27.905 | 13.458 |
| C6NH7 | 2-Methyl pyridine | 30.916 | 18.164 |
| C6NH7 | Aniline | 27.972 | 23.368 |
| C6NH13 | 2-Methylpiperidine | 47.118 | 16.748 |
| C6NH13 | Cyclohexanamine | 30.709 | 25.733 |
| C6NH15 | Triethylamine | 48.195 | 22.261 |
| C7NH5 | Phenyl cyanide | 34.374 | 32.433 |
| N2 | Nitrogen | 12.911 | 53.256 |
| N2H2 | Diazene | 62.752 | 45.406 |
| N2H4 | Hydrazine | 51.307 | 47.591 |
| CN2H2 | Diazomethane | 16.022 | 15.972 |
| CN2H2 | N=N-CH2- | 25.646 | 47.502 |
| C2N2 | Cyanogen | 13.439 | 17.804 |
| C2N2H8 | 1,2-Dimethylhydrazine | 47.844 | 46.011 |
| C3N2H4 | 1H-Pyrazole | 64.670 | 41.402 |
| C3N2H4 | Imidazole | 38.444 | 21.186 |
| C3N2H10 | 1,2-Propanediamine | 32.068 | 30.070 |
| C4N2 | Dicyanoacetylene | 16.131 | 40.175 |
| C4N2H2 | Fumaronitrile | 21.894 | 13.354 |
| C4N2H4 | 1,3-Diazine | 36.420 | 19.129 |
| C4N2H4 | Pyrazine | 27.650 | 17.958 |
| C4N2H4 | Pyridazine | 68.081 | 51.188 |
| C4N2H4 | Succinonitrile | 33.855 | 22.737 |
| C4N2H6 | 2-Methyl-1H-imidazole | 68.113 | 42.656 |
| C6N2H12 | Triethylenediamine | 40.188 | 16.402 |
| N3 | Azide radical | 6.325 | 2.801 |
| N3H | Hydrazoic acid | 14.904 | 22.552 |
| C3N3H3 | 1,3,5-Triazine | 52.462 | 30.879 |
| CN4H2 | 1-H Tetrazole | 90.922 | 62.378 |
| C6N4 | Tetracyanoethylene | 66.329 | 83.534 |
| HO | Hydroxyl radical | 31.279 | 8.823 |
| H2O | Water | 22.001 | 8.813 |
| H3O | Hydronium, cation | 14.123 | 12.051 |
| CHO | HCO | 35.416 | 38.024 |
| CH2O | Formaldehyde | 20.263 | 12.600 |
| CH4O | Methanol | 40.995 | 12.185 |
| C2H2O | Ketene | 47.680 | 26.278 |
| C2H4O | Acetaldehyde | 31.664 | 13.002 |
| C2H6O | Dimethyl ether | 41.952 | 9.502 |
| C2H6O | Ethanol | 44.052 | 10.309 |
| C3H6O | Acetone | 29.050 | 13.580 |
| C3H6O | Propanal | 35.135 | 12.467 |
| C3H8O | Isopropanol | 42.592 | 12.235 |
| C3H8O | Propanol | 40.301 | 13.695 |
| C4H4O | Acetyl acetylene | 50.842 | 60.869 |
| C4H4O | Furan | 46.322 | 21.539 |
| C4H6O | 2,3-Dihydrofuran | 53.967 | 24.513 |
| C4H6O | Crotonaldehyde | 19.547 | 18.366 |
| C4H6O | Divinyl ether | 26.252 | 27.973 |
| C4H8O | Butanal | 26.539 | 13.585 |
| C4H8O | Isobutanal | 26.579 | 19.180 |
| C4H8O | Methyl ethyl ketone | 27.976 | 16.756 |
| C4H10O | Diethyl ether | 122.746 | 65.731 |
| C4H10O | t-Butanol | 40.595 | 15.797 |
| C5H8O | 2,3-Dihydro-5-methyl-furan | 44.539 | 20.200 |
| C5H8O | 3,4-Dihydro-2H-pyran | 38.999 | 10.955 |
| C5H8O | Cyclopentanone | 14.852 | 21.854 |
| C5H10O | Diethyl ketone | 15.856 | 30.893 |
| C5H10O | Tetrahydropyran | 41.446 | 11.000 |
| C6H6O | Phenol | 42.904 | 21.436 |
| C6H10O | Cyclohexanone | 40.023 | 13.202 |
| C7H6O | Benzaldehyde | 23.121 | 18.518 |
| C7H8O | Anisole | 40.437 | 14.445 |
| C8H8O | Acetophenone | 27.322 | 19.921 |
| NO | Nitric oxide, cation | 63.858 | 105.225 |
| NO | Nitric oxide | 76.737 | 0.867 |
| CNO | NCO | 52.221 | 22.084 |
| CNHO | Hydrogen isocyanate | 31.070 | 21.519 |
| CNH3O | Formamide | 44.898 | 21.631 |
| C2NH5O | Acetaldoxime | 113.114 | 49.098 |
| C2NH5O | Acetamide | 59.735 | 23.494 |
| C3NH3O | Isoxazole | 100.540 | 46.398 |
| C3NH3O | Oxalone (oxazole) | 74.534 | 39.594 |
| C3NH5O | Acrylamine | 44.379 | 19.382 |
| C3NH5O | Methoxyacetonitrile | 52.272 | 36.381 |
| C3NH7O | Dimethylformamide | 46.462 | 29.996 |
| C3NH7O | Propanamide | 44.520 | 44.804 |
| C4NH5O | 3-Methyl isoxazole | 102.120 | 50.462 |
| C4NH5O | 5-Methyl isoxazole | 101.461 | 49.339 |
| C4NH7O | 2-Pyrrolidinone | 44.389 | 23.334 |
| C4NH7O | 4,5-Dihydro-2-methyl oxazole | 101.095 | 62.684 |
| C4NH7O | Methacrylamide | 42.331 | 22.758 |
| C4NH9O | 2-Methyl propanamide | 41.477 | 20.699 |
| C4NH9O | Butanamide | 43.063 | 21.544 |
| C4NH11O | N,N-Diethyl-hydroxylamine | 96.665 | 44.518 |
| C5NH5O | 2-Pyridinol | 51.392 | 24.891 |
| C5NH5O | 3-Pyridinol | 39.319 | 19.221 |
| C5NH5O | 4-Pyridinol | 38.034 | 15.619 |
| N2O | Nitrous oxide | 10.055 | 21.044 |
| CN2H4O | Urea | 48.383 | 19.329 |
| C2N2H6O | N-Methyl urea | 44.261 | 22.193 |
| O2 | Oxygen (Singlet) | 177.061 | 9.171 |
| O2 | Oxygen (Triplet) | 179.698 | 11.883 |
| H2O2 | Hydrogen peroxide | 131.677 | 51.097 |
| CO2 | Carbon dioxide | 60.709 | 4.126 |
| CHO2 | Formate, anion | 38.670 | 30.917 |
| CH2O2 | Formic acid | 60.991 | 22.717 |
| C2H2O2 | trans Glyoxal | 9.206 | 29.323 |
| C2H3O2 | Acetate, anion | 24.275 | 24.544 |
| C2H4O2 | Acetic acid | 70.252 | 26.769 |
| C2H4O2 | Methyl formate | 84.411 | 35.178 |
| C2H6O2 | Dimethyl peroxide | 128.050 | 39.281 |
| C2H6O2 | Ethylene glycol | 59.646 | 20.064 |
| C3O2 | Carbon suboxide | 69.482 | 2.243 |
| C3H4O2 | 2-Oxo-propanal | 40.003 | 17.963 |
| C3H4O2 | 2-Propenoic acid | 72.339 | 20.764 |
| C3H4O2 | beta-Propiolactone | 45.404 | 13.018 |
| C3H6O2 | 1,3-Dioxalane | 35.024 | 19.398 |
| C3H6O2 | Ethyl formate | 55.845 | 19.728 |
| C3H6O2 | Methyl acetate | 58.786 | 17.588 |
| C3H6O2 | Propionic acid | 49.264 | 23.786 |
| C3H8O2 | 1,3-Propanediol | 69.176 | 22.604 |
| C3H8O2 | Dimethoxymethane | 51.576 | 12.255 |
| C3H8O2 | Propylene glycol | 62.492 | 23.854 |
| C4H6O2 | Diacetyl | 33.111 | 20.406 |
| C4H6O2 | Methyl 2-propenoate | 59.743 | 20.999 |
| C4H8O2 | 1,1 Dimethoxy ethene | 46.555 | 28.790 |
| C4H8O2 | 1,3 Dioxan | 49.970 | 14.450 |
| C4H8O2 | 1,4-Dioxane | 51.484 | 10.918 |
| C4H8O2 | Ethyl acetate | 55.013 | 17.263 |
| C4H10O2 | 1,2-Dimethoxyethane | 109.426 | 46.306 |
| C4H10O2 | 1,4 Butandiol | 105.161 | 51.670 |
| C4H10O2 | Dimethyl acetal | 40.931 | 35.803 |
| C5H8O2 | Acetylacetone | 23.312 | 34.343 |
| C6H12O2 | Hexanoic acid | 66.356 | 27.592 |
| C7H6O2 | Benzoic acid | 62.148 | 16.946 |
| NO2 | Nitrogen dioxide | 51.862 | 20.159 |
| NHO2 | Nitrous acid, trans | 119.630 | 48.545 |
| CNH3O2 | Methyl nitrite | 124.706 | 48.513 |
| CNH3O2 | Nitromethane | 75.774 | 24.700 |
| C2NH5O2 | Glycine | 68.591 | 29.796 |
| C3NH7O2 | Alanine | 57.661 | 26.591 |
| C3NH7O2 | beta-Alanine | 60.677 | 24.817 |
| C3NH7O2 | Isopropylnitrite | 104.117 | 42.688 |
| C3NH7O2 | Propyl nitrite | 114.786 | 47.167 |
| C3NH7O2 | Urethane | 65.416 | 20.773 |
| C5NH9O2 | Proline | 87.871 | 33.473 |
| C6NH5O2 | Nitrobenzene | 48.802 | 12.490 |
| C2N2H4O2 | Oxalamide | 60.700 | 25.436 |
| C2N2H6O2 | N-Nitrodimethylamine | 62.493 | 25.710 |
| C4N2H4O2 | Uracil | 50.352 | 35.848 |
| O3 | Ozone | 180.153 | 20.589 |
| C3H6O3 | 1,3,5-Trioxane | 55.828 | 13.778 |
| C3H8O3 | Glycerol | 70.627 | 24.147 |
| C4H2O3 | Malaic anhydride | 42.613 | 30.476 |
| NHO3 | Nitric acid | 75.722 | 33.525 |
| CNH3O3 | Methyl nitrate | 45.529 | 29.312 |
| C2NH3O3 | Oxamic acid | 66.760 | 16.641 |
| C2NH5O3 | Ethyl nitrate | 69.365 | 32.820 |
| C3NH7O3 | Serine | 62.759 | 29.878 |
| N2O3 | Dinitrogen trioxide | 90.632 | 50.463 |
| C2H2O4 | Oxalic acid | 76.342 | 24.934 |
| N2O4 | Dinitrogen tetroxide | 58.535 | 27.883 |
| N2O5 | Dinitrogen pentoxide | 81.852 | 48.352 |
| HF | Hydrogen fluoride | 42.440 | 42.534 |
| CF | Fluoromethylidyne radical | 31.773 | 3.386 |
| CH3F | Fluoromethane | 56.439 | 15.925 |
| C2HF | Fluoroacetylene | 12.129 | 35.863 |
| C2H3F | Fluoroethylene | 49.943 | 7.548 |
| C2H5F | Fluoroethane | 61.873 | 14.238 |
| C3H7F | 2-Fluoropropane | 65.938 | 12.780 |
| C6H5F | Fluorobenzene | 59.242 | 14.933 |
| CNF | Cyanogen fluoride | 4.332 | 47.266 |
| OF | Fluorine oxide | 146.425 | 10.348 |
| HOF | Hypofluorous acid | 139.415 | 26.606 |
| CHOF | HCOF | 89.492 | 25.404 |
| C2H3OF | Acetyl fluoride | 89.311 | 17.028 |
| NOF | Nitrosyl fluoride | 101.644 | 80.951 |
| O2F | Fluorine dioxide | 137.358 | 38.408 |
| NO3F | Fluorine nitrate | 154.290 | 59.658 |
| F2 | Fluorine molecule | 144.153 | 0.208 |
| CF2 | Difluoromethylene | 29.545 | 7.532 |
| CH2F2 | Difluoromethane | 41.805 | 10.761 |
| C2H2F2 | gem-Difluoroethylene | 43.293 | 10.860 |
| C2H4F2 | 1,1-Difluoroethane | 55.299 | 10.281 |
| C6H4F2 | 1,2-Difluorobenzene | 73.597 | 21.793 |
| C6H4F2 | 1,3-Difluorobenzene | 79.806 | 19.928 |
| C6H4F2 | 1,4-Difluorobenzene | 83.772 | 23.537 |
| N2F2 | cis-Difluorodiazene | 136.754 | 28.861 |
| N2F2 | trans-Difluorodiazene | 162.092 | 36.801 |
| OF2 | Difluorine oxide | 172.425 | 12.186 |
| CF3 | Trifluoromethyl | 42.882 | 7.120 |
| CHF3 | Trifluoromethane | 28.935 | 23.916 |
| C2HF3 | Trifluoroethylene | 58.367 | 18.977 |
| C2H3F3 | 1,1,1-Trifluoroethane | 44.162 | 23.641 |
| NF3 | Nitrogen trifluoride | 140.046 | 23.111 |
| C2NF3 | Trifluoroacetonitrile | 181.028 | 148.417 |
| NOF3 | F3NO | 126.938 | 38.928 |
| C2HO2F3 | Trifluoroacetic acid | 57.566 | 32.190 |
| CF4 | Carbon tetrafluoride | 39.994 | 24.187 |
| C2F6 | Hexafluoroethane | 64.367 | 30.206 |
| C4F6 | Perfluorobutadiene | 89.146 | 30.442 |
| C6F6 | Hexafluorobenzene | 149.749 | 78.901 |
| C3F8 | Perfluoropropane | 83.956 | 44.568 |
